# Covalent Templated Catenane Synthesis Requiring Hindered Ester Cleavage

**DOI:** 10.1021/acs.joc.6c01398

**Published:** 2026-08-04

**Authors:** Nick Westerveld, Martin J. Wanner, Jan H. van Maarseveen

**Affiliations:** Van ‘t Hoff Institute for Molecular Sciences, 1234 University of Amsterdam, Science Park 904, 1098 XH Amsterdam, The Netherlands

## Abstract

Previously, we developed efficient methodology toward hetero [n]­rotaxanes using 2,5-dihydroxyterephthalate as a covalent scaffold in the axis of a 30-membered all-carbon macrocycle. Herein we show that to arrive at the more challenging catenanes, proper preorganization provided by 2,6-dihydroxyterephthalate is required for efficient closure of the second macrocycle. Extreme steric hindrance of the specific terephthalate ester covalently linking both macrocycles prevented disconnection of the rings by saponification. To overcome this, to the endocyclic phenyl rings in the macrocycle spanning the templating terephthalate moiety 4-OMe groups were introduced allowing liberation of the two macrocycles by oxidation to the *para*-benzoquinones with concomitant cleavage of the esters. However, the strong electron-withdrawing properties of the central terephthalic template completely blocked oxidation. Eventually, aluminum hydride as a small and powerful nucleophile was able to cleave the highly shielded ester liberating the mechanically interlocked catenane architecture.

## Graphical abstract

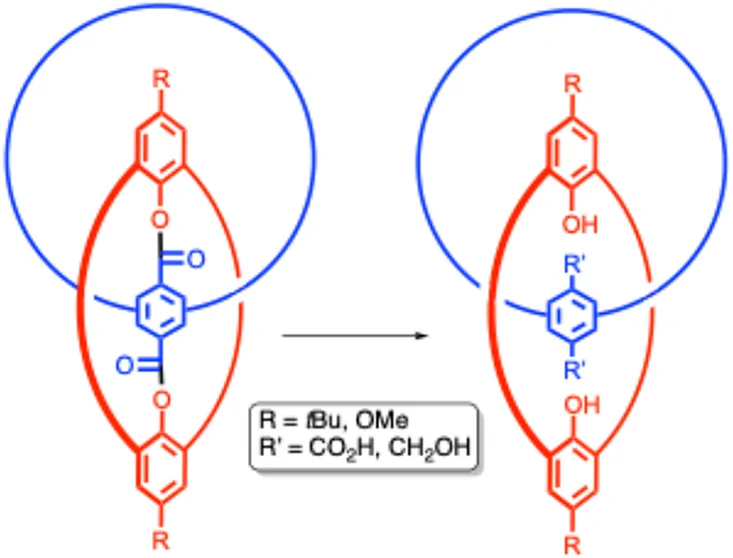

## Introduction

The vast majority of rotaxanes and catenanes are made by using supramolecular templating to preorganize the mechanically interlocked components.^1,2^ Self-assembly via supramolecular recognition stands out in its simplicity and generally high yields are obtained. As with every method, also supramolecular templating has its drawbacks such as the limited types of suitable moieties for the required noncovalent binding. For this reason, several groups embarked on the development of novel routes to synthesize “impossible” mechanically interlocked molecules that lack supramolecular recognition elements.^3^ Over the recent years, we have developed new methodology based on temporal covalent connection to preorganize the ring-precursor components toward mechanically interlocked molecules (MiMs) via clipping strategies (Scheme 1).^4–6^

**Scheme 1:**
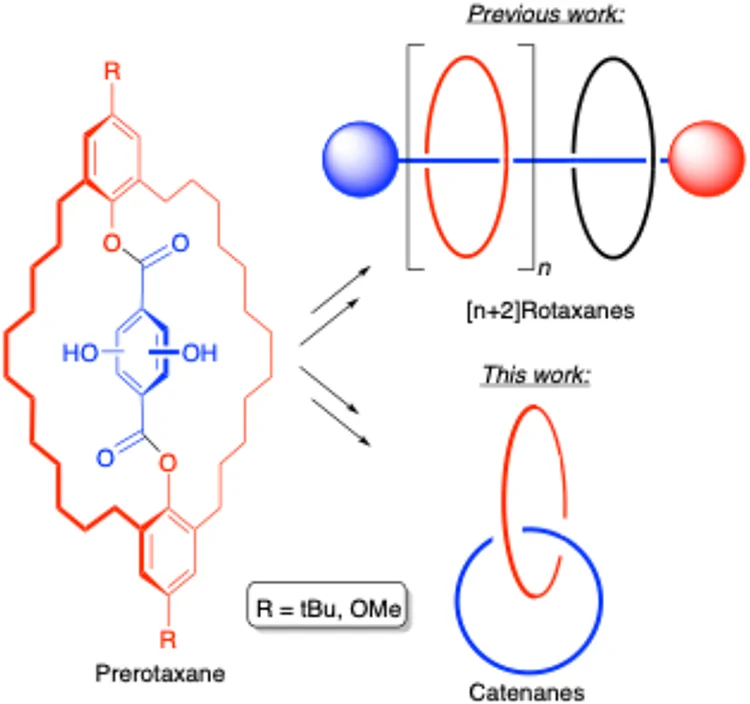
Outline of Previous Work towards Rotaxanes and the Current Expansion to Give Catenanes

By using 2,5-dihydroxyterephthalic acid as a templating thread-component we have recently disclosed efficient methodology for the synthesis of “impossible” homo- and hetero­[*n*]­rotaxanes.^5^ Key is the perpendicular arrangement of the central terephthalate phenyl group with respect to the plane of the macrocycle in a prerotaxane. As a result, the terephthalic 2,5-phenolic hydroxy groups each point to a different side of the plane of the macrocycle paving the way for further functionalization toward MiMs. Herein, we expanded this methodology toward catenanes. As will be detailed, in this journey we had to tackle extreme steric hindrance exerted by the so-called catenand effect.^7^

## Results and Discussion

To transform our previously described 30-membered prerotaxane 1,^5^ available on gram scale, into a catenane we also chose ring-closing metathesis (RCM) as the key reaction to introduce the second macrocycle (Scheme 2). For this, alkylation of the 2,5-phenolic hydroxyl groups in **1** with 13-bromotridec-1-ene in DMF, using K_2_CO_3_ as the base gave, after stirring for 3 days at 90 °C, RCM precursor **2** in 90% isolated yield. After extensive optimization, the best macrocyclization conditions were using Grubbs second-generation catalyst at high dilution (1 mM) in refluxing toluene. To prevent double bond migration leading to a truncated macrocycle, 1,4-quinone was added.^8^ However, precatenane **3a** was obtained in a low yield of 13% only, as a mixture of *E*/*Z*-isomers. Obviously, the 2,5-*para*-substitution pattern of the terephthalate linker tethered alkene results in an extended placement of the mutually reactive alkenes hampering macrocyclization. The mixture of *E*/*Z*-isomers was removed facilely by hydrogenolytic saturation of the alkenes to give precatenane **3b**. Liberation of the mechanically interlocked architecture by classical saponification went uneventful and catenane **4** was isolated in quantitative yield.

**Scheme 2:**
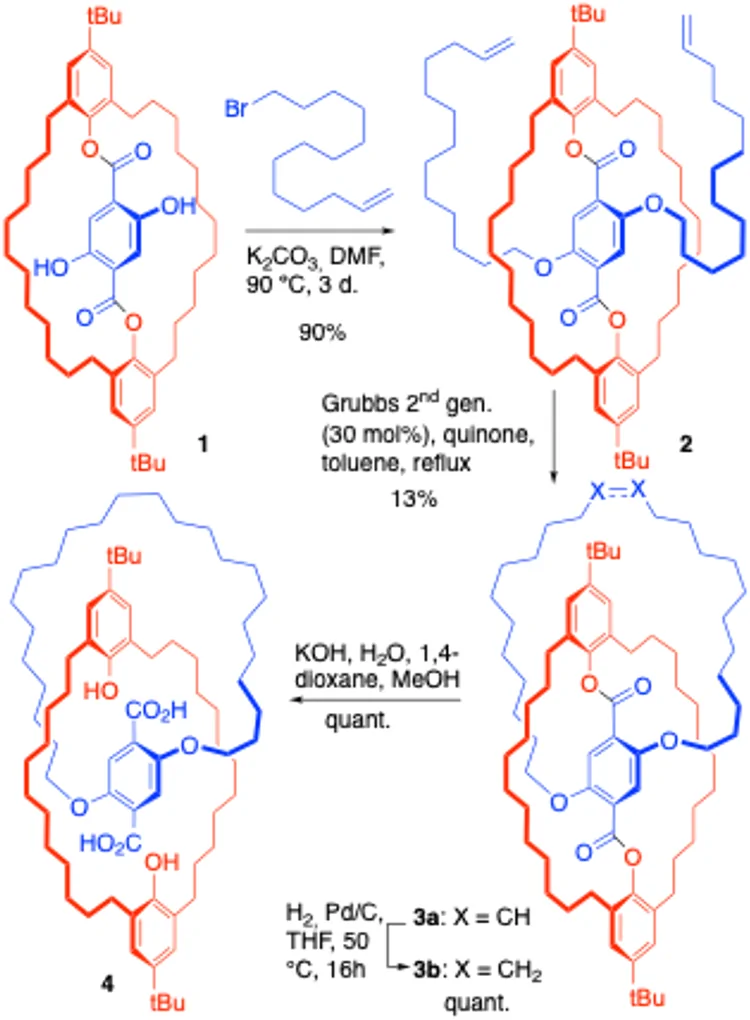
Precatenane Synthesis Starting from 2,5-Dihydroxyterephthalate Centered Prerotaxane 1

Despite of having the catenane in hand, we were not satisfied with the low yield of the second macrocyclization to give precatenane **3a**. To overcome the unfavored extended conformation of the macrocyclization precursor, we set out to prepare 2,6-dihydroxyterephthalic acid derived prerotaxane **12b**, mutually placing both hydroxyl groups in a meta-arrangement (Scheme 3). As a result, after installation of the second macrocycle precursors, the terminal alkenes will be directed in close proximity facilitating the RCM-mediated macrocyclization.

**Scheme 3:**
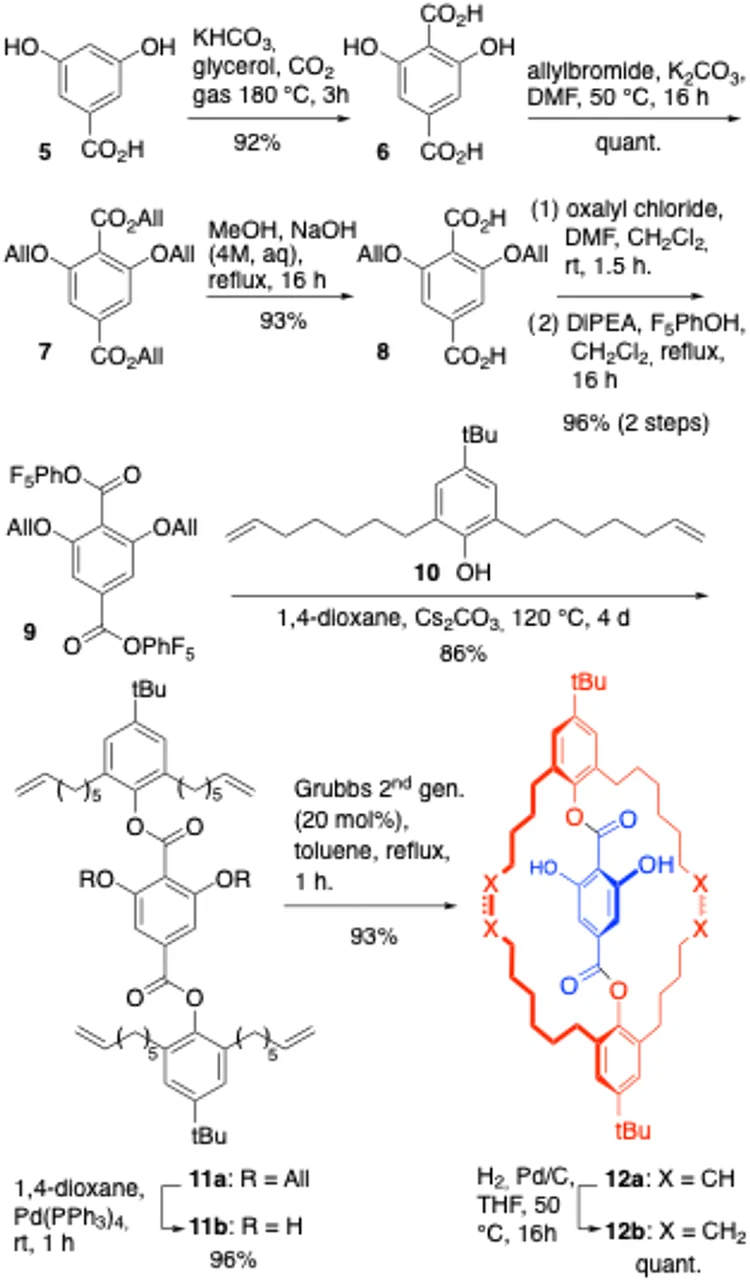
Synthesis of 2,6-Dihydroxyterephthalate Centered Prerotaxane **12b**

The synthesis of **12b** started with the Kolbe-Schmitt reaction of commercially available 3,5-dihydroxybenzoic acid (**5**) by treatment with CO_2_ dissolved in glycerol under slightly basic conditions at an elevated temperature giving 3,5-dihydroxyterephthalic acid (**6**) in excellent 92% yield.^9^ Exhaustive allylation, followed by saponification gave 3,5-diallyloxybenzoic acid (**8**) in 93% overall yield. For transformation of the carboxylic acids into pentafluorophenyl esters, the intermediate acid chlorides were reacted with pentafluorophenol in dichloromethane using DiPEA as the base, providing **9** in 96% overall yield. Displacement of the pentafluorophenols by 2,6-di­(hept-6-en-1yl)­phenol (**10**) gave **11a**, which was subjected to Pd(0)-catalyzed allyl protective group removal to give RCM precursor **11b** in 96% yield. Grubbs’ second-generation catalyst mediated RCM in refluxing toluene gave prerotaxane **12a** in 93% isolated yield as a mixture of *E*/*Z*-isomers. It should be noted that this RCM reaction was conducted without using 1,4-quinone as an additive and mass spectrometry indeed revealed that also some methylene-truncated prerotaxane was formed due to double bond migration preceding the RCM macrocyclization. Catalytic hydrogenation transformed this mixture into to the fully saturated prerotaxane **12b**. The NMR spectra of both **12a**,**b** showed a remarkable difference in the shift of both phenolic protons of more than 2 ppm due to the fact that one forms a hydrogen bond to the flanking ester carbonyl. This also desymmetrizes the axle phenyl group resulting in multiple peaks for the two protons. Different mutual orientations of the two ester carbonyl groups may also contribute to desymmetrizing the axle phenyl group.

Alkylation of the phenolic hydroxyl group in **12b** with 13-bromotridec-1-ene using K_2_CO_3_ as the base gave precursor **13**, setting the stage for the second RCM reaction (Scheme 4). Gratifyingly, subjection of **13** to Grubbs second-generation catalyst in refluxing toluene efficiently closed the 29-membered macrocycle to give precatenane **14a** as a mixture of *E*/*Z*-isomers in 53% yield after purification. This macrocyclization was performed in the absence of quinone too and HRMS-analysis revealed the extensive formation of truncated rings lacking one up to three methylene fragments. Surprisingly, batches made earlier using the same catalyst only suffered slightly from the formation of methylene truncated macrocycles. Catalytic hydrogenation of the internal alkene removed the *E*/*Z*-mixture yielding precatenane **14b** in 88% yield.

**Scheme 4:**
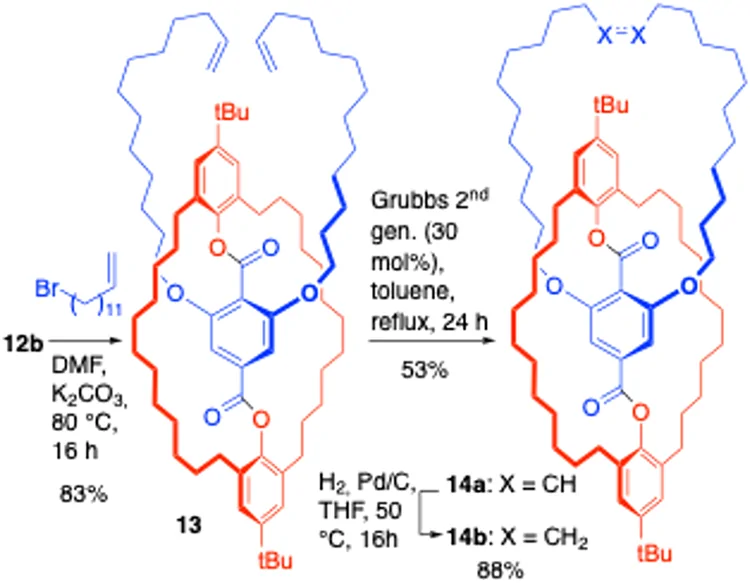
Synthesis of 2,6-Dihydroxyterephthalate Centered Precatenane **14b**

Liberation of the catenane by classic saponification seemed to proceed well according to TLC analysis showing only one clean product spot, as expected with higher polarity. However, the broad 1H-NMR signals of the Ar–CH_2_– and Ar–O–CH_2_– signals, but more convincingly the fact that the aromatic protons *ortho* with respect to the 4-*t*Bu-groups appeared separately in the spectra, made us suspicious if *both* esters were cleaved. High resolution mass spectrometric analysis revealed that indeed only the sterically less demanding ester was hydrolyzed giving **15a** (Scheme 5).

**Scheme 5:**
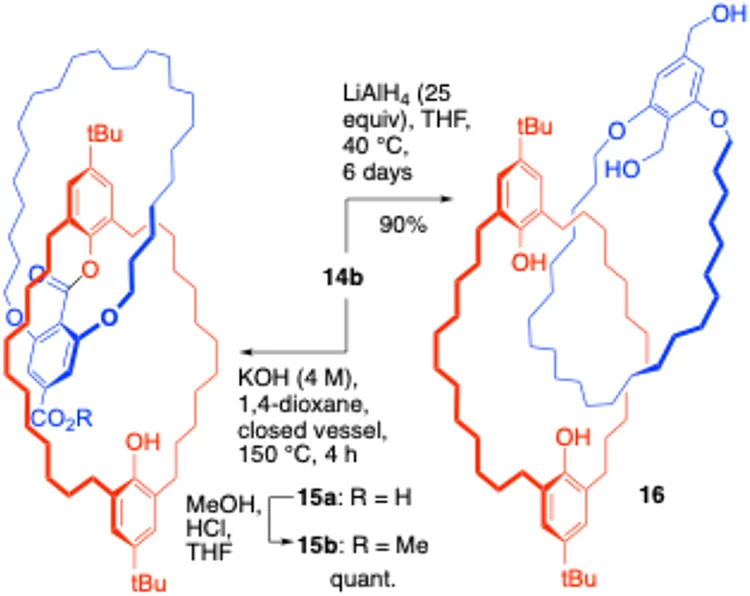
Catenane Release from Precatenane **13b**

Further proof was obtained after esterification of **15a** with methanol under HCl catalysis to give **15b** in a quantitative yield. It should be noted that even under these acidic conditions in the presence of an excess of methanol the single connecting phenolic ester bond showed to be completely inert to transesterification. Remarkably, all attempts to cleave the remaining phenolic ester by means of saponification or transesterification failed, even using very forcing conditions. As a method of last resort to cleave the sterically very congested ester bond, **14b** was treated with an excess of LiAlH_4_ at elevated temperature. Fortunately, indeed the starting precatenane was consumed but it needed stirring for 6 days, as was monitored by TLC analysis, before the reaction went to completion. ^1^H NMR and mass spectrometric analysis revealed that indeed both esters were cleaved providing catenane **16** in 90% yield, albeit slightly contaminated by some impurities that could not be removed. We realize at his stage that these drastic conditions limit the applicability of our covalent methodology.

Although steric factors are intrinsically connected to precatenanes, we never expected that the even somewhat activated phenolic esters would be so hard to cleave.^10^ This shows the strong influence of the so-called catenand effect, especially exerted by the fact that the ester bond is flanked by two 2,6-disubstituted phenyl rings. In earlier work, we also had to tackle the catenand effect in cases of ketals connecting the two macrocycles in a precatenane.^11,12^ This triggered us to search for alternative strategies to overcome sterically hindered ester cleavage. We reasoned that replacing the 4-*t*Bu groups by 4-OMe groups leads to hydroquinones that are amenable for oxidative transformation into benzoquinones with concomitant ester cleavage liberating the catenane (Scheme 6).

**Scheme 6:**
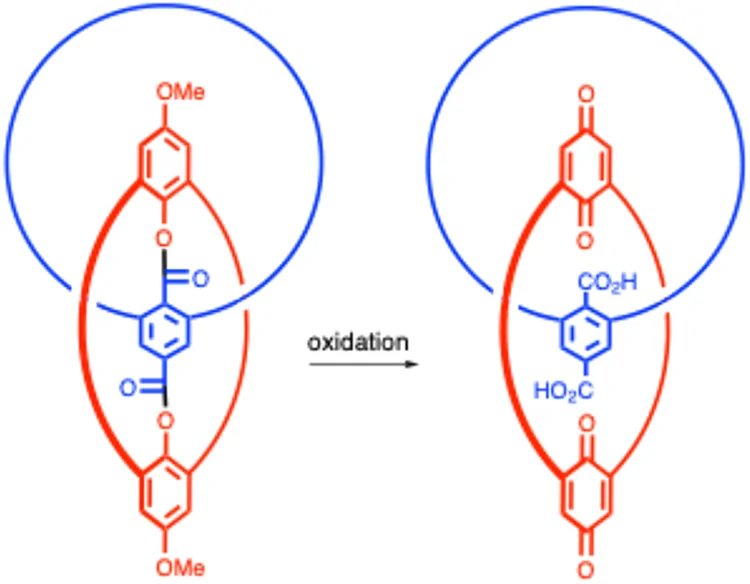
Proposed Oxidative Disconnection of the Macrocycles

Remarkably, a similar strategy was used by Schill in the first directed covalent synthesis of a catenane back in 1964.^13^ We were further encouraged by literature precedent by the Heathcock group who used esters derived from 2,6-di-*tert*-Bu-4-methoxyphenol in controlled aldol reactions.^14^ These esters also showed complete inertness toward saponification or transesterification conditions but could be cleaved in high yields using ceric ammonium nitrate (CAN) as an oxidant. Furthermore, looking ahead to future applications, benzoquinones are nature’s utmost redox mediators and may be used as such in artificial photosynthetic devices.^15^

The synthesis of the 4-methoxyphenol-centered prerotaxane started by reacting 4-methoxyphenol (**17**) with paraformaldehyde using NaOH as a base to give **18**,^16^ which was oxidized using MnO_2_ resulting in 2-hydroxy-5-methoxyisophthalaldehyde (**19**) in 88% overall yield (Scheme 7).^17^ Introduction of the tethered alkenes as RCM precursors was accomplished by hex-5-en-1-ylmagnesium bromide addition to **19** providing **20** as a mixture of diastereomers, followed by silane reduction of the resulting benzylic alcohols to give macrocycle precursor **21** in excellent overall yield.^4^ Transesterification of the pentafluorophenyl esters in **9** with phenol **21** required heating for 4 days to reach full consumption of the starting materials and gave RCM precursor **22a** in 86% yield after purification. Both removal of the allyl protective groups and the subsequent RCM reaction, followed by hydrogenolytic removal of the olefinic bonds, proceeded uneventfully and gave prerotaxane **23b** in 98% overall yield. As was found for prerotaxanes **12a**,**b**, the phenolic protons show a remarkable peak separation due to partial H-bonding desymmetrizing the macrocycle plane resulting in splitting of the central phenyl protons in multiple peaks.

**Scheme 7:**
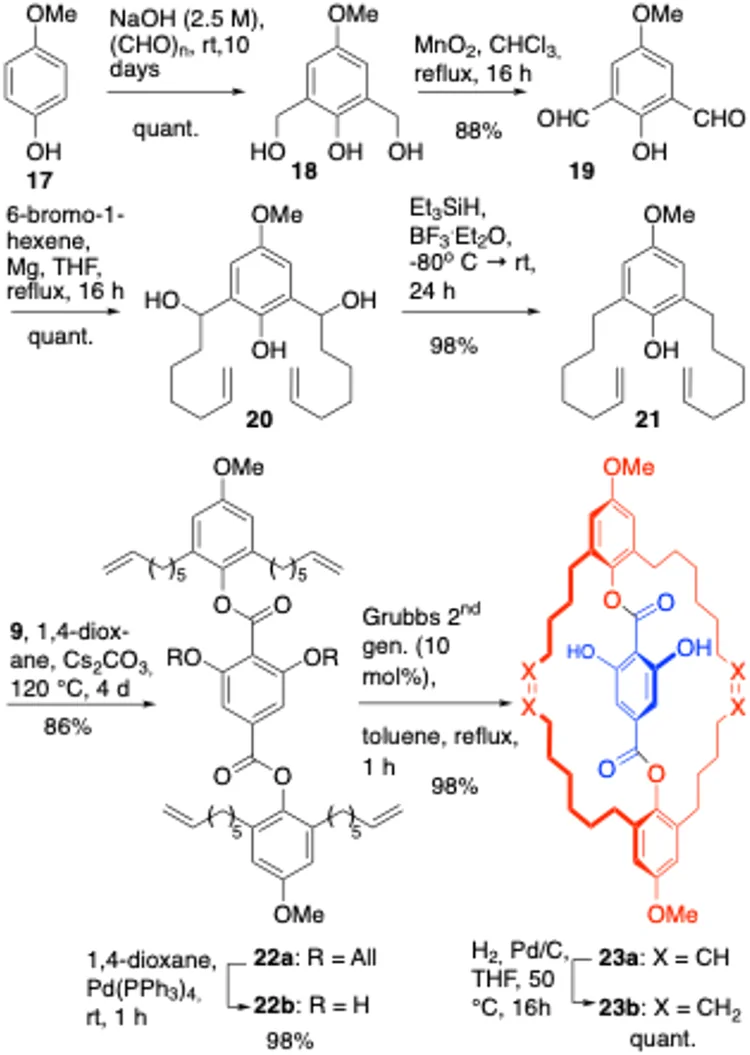
Synthesis of the Macrocycle *para*-Oxyquinone Centered Prerotaxane

Attachment of the tethered alkenes giving **24**, and the following RCM macrocyclization provided precatenane **25a** in a high overall yield of 72% (Scheme 8). Pd-catalyzed hydrogenation of the internal alkene resulted in removal of the *E*/*Z*-mixture to give precatenane **25b** in quantitative yield.

**Scheme 8:**
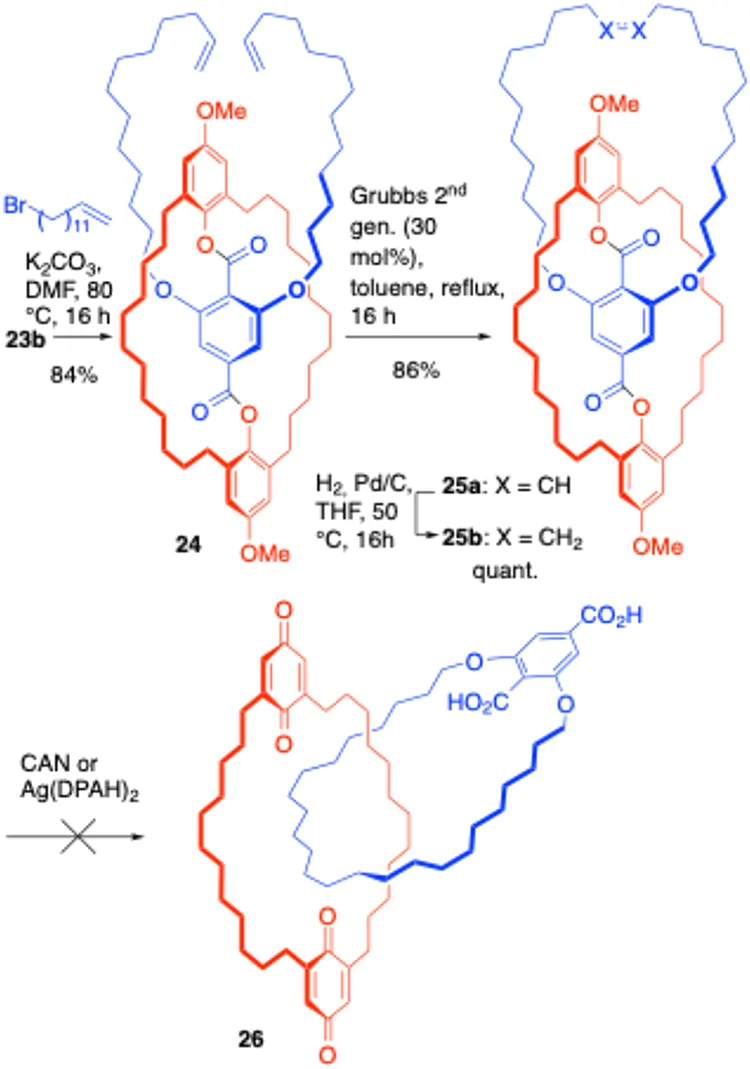
Synthesis of the Macrocycle *para*-Oxyquinone Centered Precatenane **23b** and Attempted Oxidative Cleavage

To our great surprise, but even more disappointment, precatenane **25b** showed complete inertness toward oxidation conditions using silica-supported CAN^18^ or the even very powerful oxidant silver­(II) bis­(hydrogen dipicotinate)^19^ (Ag­(DHAP)_2_).

To study the origin of this unexpected failure we conducted model oxidations of esters derived from 2,6-di­(hept-6-en-1-yl)-4-methoxyphenol (**21**) being the acetate **27**, benzoate **27**, 2,6-dimethoxybenzoate **29** together with terephthalate diester **31** and trans 1,4-cyclohexanedicarboxylate diester **32** (Scheme 9).

**Scheme 9:**
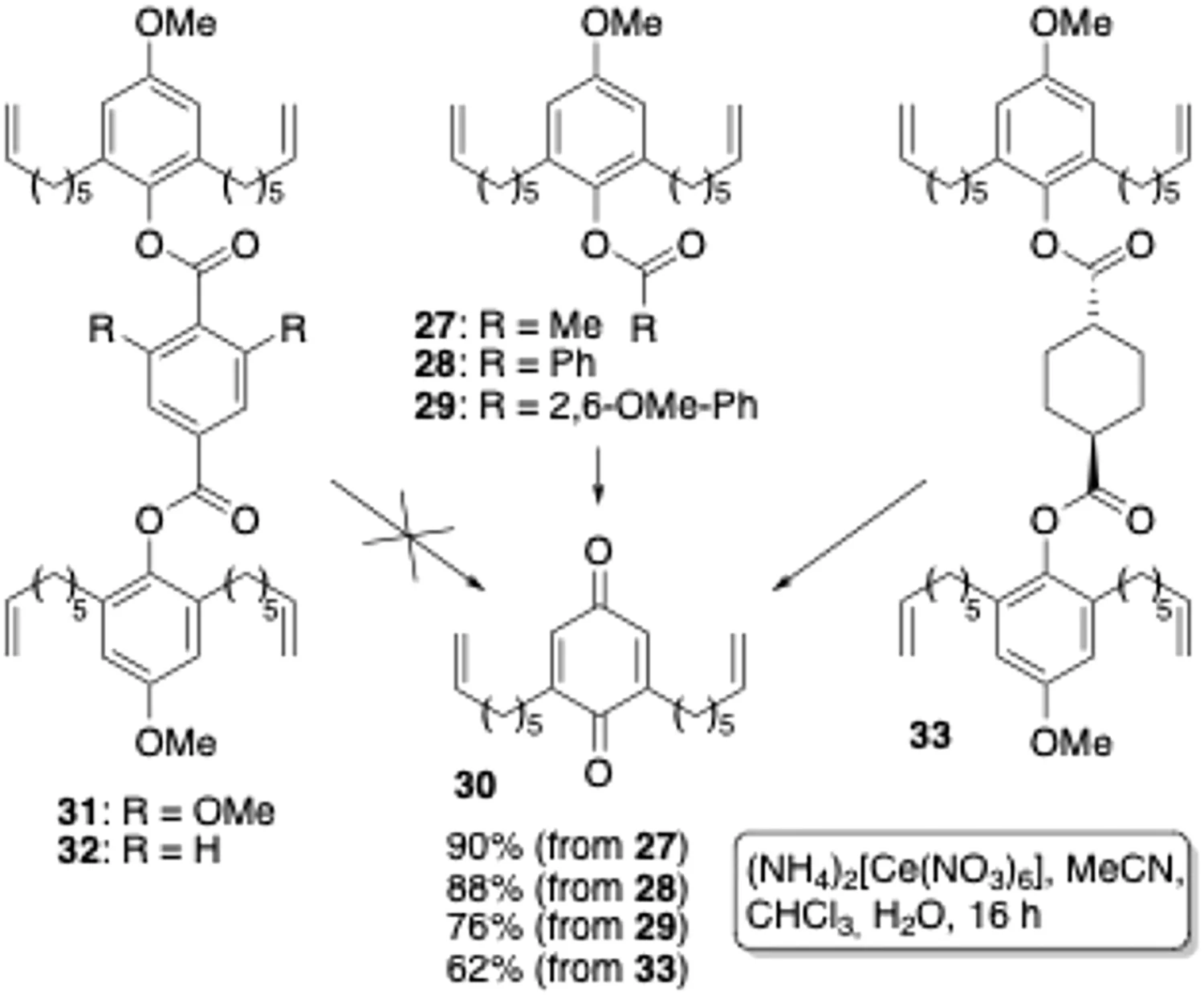
Oxidations of Model 2,6-di­(hept-6-en-1-yl) *para*-Oxyquinones Providing 2,6-di­(hept-6-en-1-yl)-*para*-Benzoquinone

Using CAN as the oxidant, alkoxyquinones **27–29** could be oxidized smoothly to give 2,6-di­(hept-6-en-1-yl)-*p*-benzoquinone (**30**) in yields ranging between 76 and 90%. In stark contrast, using the same the oxidation conditions, phthalates **31** and **32** remained unaffected. Even after prolonged treatment with Ag­(DHAP)_2_ as the oxidizing reagent these phthalates did not show any reactivity. After electronic decoupling of both esters by replacing the conjugated phthalate phenyl group by an aliphatic cyclohexane moiety to give **33**, CAN-mediated oxidation of both alkoxyquinones smoothly gave quinone **30** in 62% isolated yield. Apparently, not steric factors but the strong electron withdrawing properties of the phthalate diester completely blocks initial electron abstraction from the 2,6-dialkyl-4-methoxyphenoxy moiety. It must be noted, however, that although quinone **30** could be obtained in pure form after chromatography, it was prone to decomposition. Most probably, tautomerization of quinone **30** provides the quinone methide which is known to be highly reactive^20^ making such moieties useful for further synthetic applications.^21^

Finally, to make the benzoquinone-containing catenane, precatenane **25b** was treated with an excess of LiAlH_4_ to cleave both esters (Scheme 10). In line with the earlier reduction of precatenane **14b** incorporating 4-*t*Bu-phenols, heating was required for 5 days before all precatenane was consumed. After work up, the catenane was liberated to give the tetraol **34** in 90% isolated yield after purification. Surprisingly, CAN-mediated oxidation of the resulting electron-rich *para*-hydroquinone moieties aiming at **35** only gave a complex mixture of compounds that could not be purified further. As found for the oxidation of model *para*-oxyquinone esters **27–29** providing 2,6-dialkyl-*para*-benzoquinone **30** as an unstable compound, it must be concluded that also oxidation of **34** leads to decomposition.

**Scheme 10:**
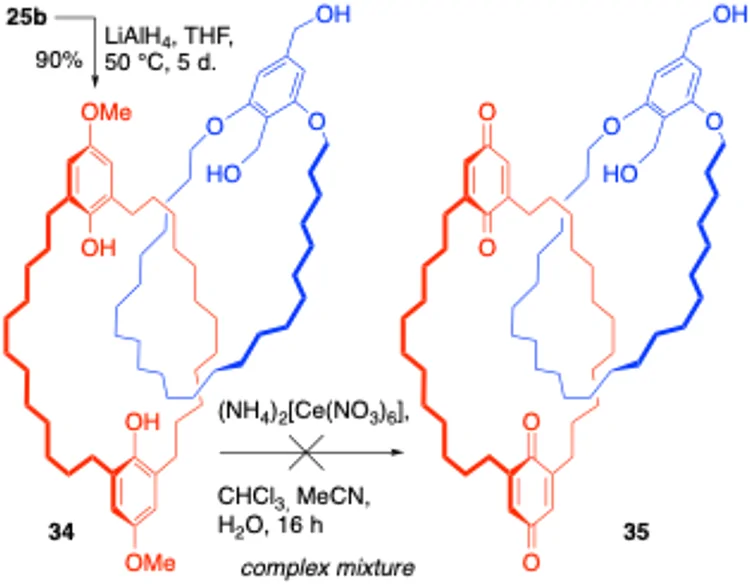
Reductive Cleavage of the Ring-Connecting Ester Linkage and Attempted Oxidation to a Benzoquinone-Containing Catenane

Although we could successfully expand our covalent methodology for the synthesis of MiMs toward catenanes, the harsh conditions required for the disconnection of the final ester bond hampers the eventual applicability to functional molecules. Work is in progress to understand the pathways of decomposition of the 2,6-dialkyl-*para*-benzoquinones to allow the design of alternative *para*-oxyquinones enabling not only mild oxidative liberation of the mechanically interlocked components circumventing the catenand effect but also further applications as redox mediators.

## Conclusions

By using 2,5-dihydroxyterephthalate as the central template, the two macrocycles giving the precatenane both could be closed using RCM as the key step. However, the second macrocyclization gave a low yield due to extended placement of the terminal alkenes at the precursor chain hampering cyclization. Indeed, by starting 2,6-dihydroxyterephthalate a high yield could be obtained also for the second macrocyclization. Unexpectedly, due to extreme steric hindrance effected by the catenand effect, cleavage by saponification of the specific terephthalate ester connecting both macrocycles could not be accomplished. An alternative approach employing two *para*-oxyquinone moieties in the second macrocycle to enable oxidation to the quinones with concomitant cleavage of the esters also failed. In this case, the strong electron-withdrawing properties of the central terephthalic template blocked oxidation. Ultimately, the small and powerful nucleophile aluminum hydride could cleave the highly shielded esters liberating the catenane. In the case of the catenane of which one of the macrocycles contains two *para*-hydroquinones, CAN oxidation gave a complex mixture of decomposed products that could not be identified. Further work will aim at substituting the central strong electron withdrawing terephthalic template by aliphatic analogs allowing liberation of the catenane by mild chemical or electrochemical oxidation in combination with introducing chemically stable 2,6-connected *para*-quinones to allow further application as redox mediators in mechanically interlocked molecular photocatalytic devices.

## Experimental Section

### General Methods and Materials

Reactions were carried out under air and without additional measures such as drying, unless stated otherwise. Heating and stirring was performed using an oil bath and standard thermostatized stirring plates and Teflon stirring beans. Thin layer chromatography (TLC) was performed on Merck TLC plates (0.25 mm) precoated with silica gel 60 F254. Flask column chromatography was performed using SilaFlash P60 (40–63 μm) under compressed air flow. Starting materials and reagents were used as supplied by commercial vendors. Anhydrous CH_2_Cl_2_ and CH_3_CN were freshly distilled from CaH_2_. Dried THF was obtained through distillation with sodium and dried solvents were stored under N_2_ atmosphere. A Bruker DRX-400 MHz instrument was used to record all NMR spectra. Chemical shifts (δ) are reported in ppm relative to residual undeuterated solvent peaks. Data of the recorded ^1^H NMR spectra are described as follows: chemical shift (multiplicity, coupling constant when applicable, number of H). The following abbreviations are used to report the multiplicities: s (singlet), d (doublet), t (triplet), q (quartet), quint (quintet), dd (doublet of doublet), m (multiplet), br m (broad multiplet). High resolution mass spectra (HRMS) were recorded on an AccuTOF GC v 4g, JMS-T100GCV Mass spectrometer (JEOL, Japan) and HR-ToF Bruker Daltonik GmbH (Bremen, Germany) Impact II, an ESI-ToF MS capable of resolution of at least 40,000 fwhm. FD/FI probe was equipped with FD Emitter, Carbotec, FD 10 μm. Current rate 51.2 mA/min over 1.2 min, using field desorption (FD) as ionization method.

#### 13-Bromotridec-1-ene

The synthesis was carried out according to a literature procedure with slight adaptations.^22^ An oven-dried 250 mL round-bottom flask was loaded with 1,10-dibromodecane (16 g, 1 equiv, 53 mmol) and dry diethyl ether (100 mL) under a nitrogen flow. Using a syringe, lithium tetrachlorocuprate­(II) (11 mL of a 0.1 molar in THF, 0.02 equiv, 1.1 mmol) was added. Via a dripping funnel was slowly added allyl magnesium bromide (7.7 g, 53 mL, 1.0 molar in THF, 1 equiv, 53 mmol) over the course of an hour. The resulting mixture was stirred for 16 h at room temperature and quenched with a saturated aqueous ammonium chloride solution (100 mL). The mixture was transferred to a separatory funnel and washed with water (100 mL) and pentane (100 mL), separated, washed with pentane (3 × 100 mL), dried over anhydrous magnesium sulfate, filtered and evaporated. The residue was purified by column chromatography over silica using pentane as the eluent to give 13-bromotridec-1-ene (5.3 g, 38%) as a clear liquid. ^1^H NMR (CDCl_3_, 400 MHz) δ 5.85–5.75 (m, 1H), 5.01–4.90 (m, 2H), 3.39 (t, 2H, *J* = 8 Hz), 2.07–2.01 (m, 2H), 1.88–1.81 (m, 2H), 1.44–1.26 (m, 16H) ppm. ^13^C­{^1^H} NMR (CDCl_3_, 101 MHz) δ 139.2, 114.2, 33.9, 33.9, 33.0, 29.7, 29.6, 29.6, 29.6, 29.3, 29.1, 28.9, 28.3 ppm.

#### Precatenane Precursor (**2**)

A dry 25 mL round-bottom flask was loaded with **2** (0.26 g, 1 equiv, 0.33 mmol), DMF (5 mL, dry) and potassium carbonate (0.14 g, 3.0 equiv, 0.98 mmol) and the resulting mixture was stirred for 15 min. 13-Bromotridec-1-ene (0.19 g, 2.2 equiv, 0.72 mmol) was added via syringe and the mixture was stirred at 90 °C for 3 days. After cooling to room temperature, the mixture was poured into water (50 mL) and the round-bottom flask was washed with water (2 × 25 mL) and DCM (2 × 25 mL). The mixture was separated and the water layer was washed with DCM (3 × 25 mL), dried over anhydrous magnesium sulfate, filtered and evaporated. The residue was purified by column chromatography over silica using a mixture of DCM and pentane (1:2) as the eluent to afford **2** as a white solid (0.34 g, 90%). ^1^H NMR (CDCl_3_, 400 MHz) δ 7.84 (br s, 1H), 7.73 (br s, 1H), 7.16 and 7.15 (2 × br s, 4H), 5.88–5.78 (m, 2H), 5.04–4.92 (m, 4H), 4.17–4.11 (m, 4H), 2.74–2.39 (m, 8H), 2.07 (q, 4H, *J* = 8.0 Hz), 1.90–1.85 (m, 4H), 1.71–0.88 (m, 94H) ppm. ^13^C­{^1^H} NMR (CDCl_3_, 101 MHz) δ 165.9, 165.8, 162.1, 161.2, 153.6, 153.3, 152.4, 152.2, 149.0, 148.7, 145.6, 144.9, 139.3, 134.6, 134.4, 134.3, 125.3, 125.1, 123.9, 123.5, 118.5, 118.1, 116.8, 116.3, 114.2, 114.2, 69.9, 69.7, 68.1, 64.2, 34.5, 33.9, 32.0, 31.6, 30.7, 30.4, 30.3, 30.0, 29.8, 29.7, 29.6, 29.6, 29.6, 29.3, 29.3, 29.3, 29.2, 29.1, 29.0, 28.8, 28.6, 28.5, 28.4, 28.2, 26.2, 25.9, 25.8, 22.8, 14.2. HRMS (FD+) *m*/*z*: M^•+^ calculated for C_78_H_122_O_6_ 1154.9236; found 1154.9250 (1 ppm).

#### Precatenane (**3a**)

Precatenane precursor **2** (0.10 g, 1 equiv, 87 μmol) and benzoquinone (3.7 mg, 0.4 equiv, 35 μmol) were dissolved in toluene (87 mL) and evacuated three times under a nitrogen atmosphere. Grubbs M204 catalyst (15 mg, 0.2 equiv, 17 μmol) was added and the mixture was refluxed for 16 h. After cooling to room temperature, the mixture was evaporated and the residue cpurified by column chromatography over silica using a mixture of DCM and pentane (1:2) as the eluent to afford **3a** (13 mg, 11 μmol, 13%) as a white solid. ^1^H NMR (CDCl_3_, 400 MHz) 7.86–7.69 (3 × br s, 2H), 7.12 (br s, 4H), 5.37 (m, 2H), 4.20–4.09 (2 × br m, 4H), 2.70–2.30 (br m, 8H), 1.95–0.86 (m, 120H) ppm.

#### Precatenane (**3b**)

Precatenane **3a** (60 mg, 1 equiv, 53 μmol) was dissolved in THF (5 mL) and evacuated. Palladium on carbon (2.8 mg, 10% Wt, 0.05 equiv, 2.7 μmol) was added and the mixture was stirred under a balloon of hydrogen at 50 °C for 16 h. After cooling to room temperature, the mixture was filtered over Celite and evaporated to afford precatenane **3b** (60 mg, 53 μmol, quantitative) as a white solid. ^1^H NMR (CDCl_3_, 400 MHz) 7.87–7.70 (4 × br s, 2H), 7.13 (s, 4H), 4.21–4.10 (m, 4H), 2.67–2.39 (m, 8H), 1.92–0.85 (m, 106H) ppm. HRMS (FD+) *m*/*z*: M^•+^ calculated for C_76_H_120_O_6_ 1128.9079; found 1128.9103 (2 ppm).

#### Catenane (**4**)

Precatenane **3** (60 mg, 1 equiv, 53 μmol) was dissolved 1,4-dioxane (4 mL). Sodium hydroxide (13 mg, 0.32 mL, 6 equiv, 0.32 mmol) and methanol (1.4 mL) were added and the mixture was stirred at 50 °C for 16 h. After cooling to room temperature, KHSO_4_ (1 molar) was added to bring the pH to 1. The mixture was extracted with DCM (4 × 5 mL), dried over anhydrous magnesium sulfate and evaporated to afford catenane **4** (62 mg, 53 μmol, quantitative) as a white solid. ^1^H NMR (CDCl_3_, 400 MHz) 7.94–7.90 (3 x br s, 2H), 6.95 (br s, 4H), 4.30–4.12 (m, 4H), 2.61–2.53 (m, 8H), 1.94–1.75 (m, 4H), 1.63–0.87 (m, 108H) ppm. ^13^C­{^1^H} NMR (CDCl_3_, 101 MHz) δ 164.5, 151.7, 149.4, 149.2, 142.9, 142.8, 128.5, 127.8, 127.6, 124.8, 124.5, 123.0, 118.1, 71.0, 34.1, 31.8, 31.8, 31.1, 31.1, 30.9, 30.8, 30.5, 30.2, 30.0, 30.0, 29.9, 29.8, 29.8, 29.8, 29.7, 29.7, 29.6, 29.6, 29.5, 29.5, 29.4, 29.4, 29.4, 29.3, 29.3, 29.2, 28.9, 28.4, 25.7 ppm. HRMS (FD+) *m*/*z*: M^•+^ calculated for C_76_H_124_O_8_ 1164.9291; found 1164.9297 (0.5 ppm).

#### 2,6-Dihydroxyterephthalic Acid (**6**)^9^

A 500 mL three-neck round-bottom flask filled with glycerol (25 mL) was added 3,5-dihydroxybenzoic acid **5** (25 g, 1 equiv, 0.16 mol) and under manual stirring the mixture was heated to 100 °C for 1 h. After cooling to room temperature, a gas inlet was inserted into the mixture and connected to a CO_2_ cylinder. After flushing the apparatus with CO_2_ gas, potassium hydrogen carbonate (75 g, 4.6 equiv, 0.75 mol) was added in portions with manual stirring. After complete addition the mixture was heated to 185 °C for 3 h. After cooling to room temperature, a stirring bar and water (150 mL) were added. Concentrated aqueous HCl (37%, 150 mL) was added slowly and once everything was added, the reaction mixture was stirred until no more gas evolution was observed. The resulting precipitate was filtered off, washed with water (50 mL) and diethyl ether (50 mL). After drying, 2,6-dihydroxyterephthalic acid **6** (30 g, 92%) was obtained as a white solid. ^1^H NMR (CDCl_3_, 400 MHz) δ 10.48 (br s, 4H), 6.78 (s, 2H) ppm. ^13^C­{^1^H} NMR (CDCl_3_, 101 MHz) δ 171.6, 166.9, 159.7, 134.0, 108.8, 106.8 ppm.

#### Diallyl 2,6-Bis­(allyloxy)­terephthalate (**7**)

To dry DMF (200 mL) was added 2,6-dihydroxyterephthalic acid **6** (10 g, 1 equiv, 51 mmol) and potassium carbonate (70 g, 10 equiv, 0.51 mol) under vigorous stirring. Allyl bromide (27 mL, 6 equiv, 0.31 mol) was added slowly and after complete addition the mixture was warmed to 50 °C for 16 h. After cooling to room temperature, the crude reaction mixture was poured into water (1 L) and extracted with diethyl ether (3 × 500 mL). The combined organic layers were dried over anhydrous magnesium sulfate, filtered and evaporated. Diallyl 2,6-bis­(allyloxy)­terephthalate **7** (18 g, quantitative) was obtained as a thick brown oil which was used without further purification. ^1^H NMR (CDCl_3_, 400 MHz) δ7.26 (s, 2H), 6.09–5.95 (m, 4H), 5.45–5.37 (m, 4H), 5.32–5.25 (m, 4H), 4.86–4.82 (m, 4H), 4.64–4.61 (m, 4H) ppm. ^13^C­{^1^H} NMR (CDCl_3_, 101 MHz) δ 165.5, 165.3, 156.3, 132.6, 132.4, 132.1, 132.0, 118.6, 118.5, 118.1, 117.8, 106.8, 69.6, 66.1 ppm. HRMS (EI+) *m*/*z*: M^•+^ calculated for C_20_H_22_O_6_ 358.1411; found 358.1410 (0.3 ppm).

#### 2,6-Bis­(allyloxy)­terephthalic Acid (**8**)

Diallyl 2,6-bis­(allyloxy)­terephthalate **7** (18 g, 1 equiv, 50 mmol) was dissolved in methanol (500 mL) and 4 M aqueous NaOH (100 mL) was added under vigorous stirring. The mixture was stirred at 80 °C for 16 h. After cooling to room temperature, the solvent was evaporated and the dried residue was dissolved in 50 mL water and was brought to a pH of 1 with concentrated aqueous HCl (37%). The precipitate was filtered and washed with water to give 2,6-bis­(allyloxy)­terephthalic acid **8** (13 g, 93%) as a white solid after drying under vacuum. ^1^H NMR (DMSO, 400 MHz) δ 13.15 (br s, 2H), 7.20 (s, 2H), 6.04–5.95 (m, 2H), 5.40–5.35 (dq, 2H), 5.26–5.23 (dq, 2H), 4.66 (dt, 4H) ppm. ^13^C­{^1^H} NMR (DMSO, 101 MHz) δ 166.6, 166.0, 154.9, 133.1, 132.6, 118.9, 117.1, 106.2, 68.7 ppm. HRMS (FD+) *m*/*z*: M^•+^ calculated for C_14_H_14_O_6_ 278.0785; found 278.0794 (3 ppm).

#### Bis­(perfluorophenyl) 2,6-Bis­(allyloxy)­terephthalate (**9**)

A flame-dried 250 mL round-bottom flask charged with dry dichloromethane (100 mL) under a nitrogen flow and 2,6-bis­(allyloxy)­terephthalic acid **8** (10 g, 1 equiv, 36 mmol) was added under stirring followed by oxalyl chloride (16 mL, 5 equiv, 0.18 mol). One drop of DMF was added and the mixture started bubbling vigorously and was stirred for and additional hour after all solids had dissolved. The mixture was evaporated to dryness and the residue was redissolved in dry dichloromethane (50 mL) and transferred to a flame-dried 500 mL round-bottom flask containing dry dichloromethane (250 mL), DiPEA (19 mL, 3 equiv, 0.11 mol) and pentafluorophenol (17 g, 2.5 equiv, 90 mmol). The resulting mixture was stirred for 16 h at reflux. After cooling to room temperature, the mixture was poured in pentane (350 mL) and stirred. The solution was filtered over a plug of silica and Celite, washed with a 1:1 mixture of dichloromethane and pentane and evaporated. The resulting white solid was recrystallized from boiling heptane to obtain bis­(perfluorophenyl) 2,6-bis­(allyloxy)­terephthalate **9** (21 g, 35 mmol, 96%) as a white solid. ^1^H NMR (CDCl_3_, 400 MHz) δ 7.43 (s, 2H), 6.10–6.00 (m, 2H), 5.48–5.42 (dq, 2H), 5.36–5.32 (dq, 2H), 4.72 (dt, 4H) ppm. ^13^C­{^1^H} NMR (CDCl_3_, 101 MHz) δ 161.9, 161.1, 157.2, 131.8, 130.6, 118.7, 116.5, 107.5, 70.3 ppm. ^19^F NMR (CDCl_3_, 376 MHz) δ −151.2–151.3 (dt), −152.2–152.3 (dt), −157.1 (t), −157.7 (t), −161.7–161.9 (m), −162.1–162.3 (m) ppm. HRMS (FD+) *m*/*z*: M^•+^ calculated for C_26_H_12_F_10_O_6_ 610.0469; found 610.0466 (0.5 ppm).

#### Bis­(2,6-di­(hept-6-en-1-yl)-4-*tert*-butylphenyl) 2,6-Bis­(allyloxy)­terephthalate (**11a**)

A flame-dried round-bottom flask was loaded with 4-(*tert*-butyl)-2,6-di­(hept-6-en-1-yl)­phenol **10** (2.0 g, 2 equiv, 5.8 mmol), dry 1,4-dioxane (45 mL) and cesium carbonate (3.8 g, 4 equiv, 12 mmol, dried in an oven at 150 °C for 16 h). The resulting solution was stirred for 30 min at room temperature under nitrogen. Bis­(perfluorophenyl) 2,6-bis­(allyloxy)­terephthalate **9** (1.8 g, 1 equiv, 2.9 mmol) was added as a solid and the mixture was stirred at reflux for 4 days. After cooling to room temperature, cyclohexane was added (twice the reaction volume; 90 mL) and stirred for 5 min. The mixture was filtered over a mixture of Celite and silica and washed with cyclohexane. After evaporated to dryness **11a** was obtained as a yellow thick oil (2.3 g, 86%). In the case that still some starting phenol **10** is present, further purification by column chromatography can be performed using 2.5% EtOAc in pentanes as the eluent. ^1^H NMR (CDCl_3_, 400 MHz) δ 7.54 (s, 2H), 7.19 (s, 2H), 7.18 (s, 2H), 6.15–6.05 (m, 2H), 5.88–5.75 (m, 4H), 5.51–5.46 (m, 2H), 5.37–5.34 (m, 2H), 5.03–4.94 (m, 8H), 4.82–4.79 (m, 4H), 2.78 (br s, 4H), 2.54 (t, 4H, *J* = 8 Hz), 2.07 (q, 8H), 1.71–1.62 (m, 8H), 1.50–1.32 (m, 36H) ppm. ^13^C­{^1^H} NMR (CDCl_3_, 101 MHz) δ 164.3, 164.2, 157.2, 156.8, 149.2, 148.9, 148.6, 145.2, 145.0, 142.8, 139.2, 139.1, 139.1, 138.9, 138.9, 138.9, 134.3, 133.9, 133.8, 132.4, 132.0, 132.0, 127.4, 125.0, 124.7, 124.7, 118.7, 118.4, 114.4, 114.4, 114.3, 114.3, 107.2, 106.9, 70.1, 34.5, 34.5, 34.1, 33.9, 33.9, 33.8, 31.7, 31.6, 31.0, 30.7, 30.2, 30.2, 30.1, 29.9, 29.2, 29.1, 29.0, 29.0, 28.9, 28.8 ppm. HRMS (FD+) *m*/*z*: M^•+^ calculated for C_62_H_86_O_6_ 926.6419; found 926.6436 (2 ppm).

#### Bis­(4-(*tert*-butyl)-2,6-di­(hept-6-en-1-yl)­phenyl) 2,6-dihydroxyterephthalate (**11b**)

A dry round-bottom flask containing dry 1,4-dioxane (20 mL) was charged with bis­(4-(tert-butyl)-2,6-di­(hept-6-en-1-yl)­phenyl) 2,6-bis­(allyloxy)­terephthalate **11a** (2.3 g, 1 equiv, 2.5 mmol), diethylamine (1.0 mL, 4 equiv, 10 mmol) and tetrakis­(triphenylphosphine)­palladium(0) (0.15 g, 0.05 equiv, 0.13 mmol) under a nitrogen flow. The resulting reaction mixture was stirred at room temperature and after complete conversion (1 h), a saturated aqueous ammonium chloride solution (20 mL) was added and stirred for 5 min. The solution was transferred to a separatory funnel and washed with water (100 mL) and ethyl acetate (100 mL). The water layer was extracted with ethyl acetate (3 × 100 mL) and the combined organic layers were washed with water (2 × 100 mL) and brine (1 × 100 mL), dried over anhydrous magnesium sulfate, filtered and evaporated. The residue was purified by column chromatography over a silica plug with a mixture of pentane and ethyl acetate (4:1) to obtain bis­(4-(tert-butyl)-2,6-di­(hept-6-en-1-yl)­phenyl) 2,6-dihydroxyterephthalate **11b** (2.1 g, 96%) as a clear liquid. ^1^H NMR (CDCl_3_, 400 MHz) δ 9.91 (br s, 2H), 7.50 (s, 2H), 7.29 (s, 2H), 7.22 (s, 2H), 5.90–5.80 (m, 4H), 5.07–4.97 (m, 8H), 2.68–2.57 (m, 8H), 2.16–2.08 (m, 8H), 1.74–1.33 (m, 34H) ppm. ^13^C­{^1^H} NMR (CDCl_3_, 101 MHz) δ 168.4, 163.8, 161.6, 150.5, 149.2, 148.9, 145.2, 143.3, 142.7, 139.1, 139.0, 139.0, 138.8, 138.7, 137.3, 133.9, 133.8, 127.3, 125.4, 125.0, 124.6, 114.5, 114.5, 114.4, 114.3, 110.0, 102.7, 34.7, 34.5, 34.1, 33.9, 33.7, 33.6, 31.7, 31.6, 31.5, 30.9, 30.7, 30.7, 30.2, 30.1, 29.9, 29.2, 29.1, 29.0, 28.9, 28.7, 28.7 ppm. HRMS (FD+) *m*/*z*: M^•+^ calculated for C_56_H_78_O_6_ 846.5793; found 846.5793 (0 ppm).

#### Precatenane Precursor (**12a**)

A flame-dried round-bottom flask was charged with bis­(4-(*tert*-butyl)-2,6-di­(hept-6-en-1-yl)­phenyl) 2,6-dihydroxyterephthalate **11b** (1.5 g, 1 equiv, 1.8 mmol) and toluene (0.89 L). The mixture was degassed by three nitrogen/vacuum cycles. Grubbs second generation catalyst M204 (0.15 g, 0.1 equiv, 0.18 mmol) was added followed by three nitrogen/vacuum cycles before the mixture was brought to reflux. After one hour at reflux the mixture was cooled to room temperature and filtered over a silica plug and washed with chloroform. After evaporation, NMR showed the presence of a small amount of the ligand of Grubbs II catalyst. This could be removed by washing by adding methanol and crushing the solids, filtration and drying to obtain **12a** (1.3 g, 93%) as a white solid that was used without further purification. ^1^H NMR (CDCl_3_, 400 MHz) δ 10.96 (br s, 1H), 8.75 (br s, 1H), 7.47–7.40 (3 × br s, 2H), 7.19 (s, 2H), 7.12 (s, 2H), 5.22–5.10 (m, 4H), 2.52–2.42 (m, 8H), 1.84 (br s, 8H), 1.59 (m, 8H), 1.36–1.22 (m, 34H) ppm. ^13^C­{^1^H} NMR (CDCl_3_, 101 MHz) δ 168.6, 168.5, 163.9, 163.8, 163.6, 159.4, 150.9, 149.3, 149.2, 145.2, 145.1, 143.1, 143.1, 137.3, 137.1, 134.6, 134.5, 134.5, 134.3, 130.2, 130.0, 129.6, 129.5, 126.0, 125.9, 125.5, 125.4, 110.2, 109.9, 102.5, 102.5, 34.7, 34.5, 32.4, 32.3, 32.3, 32.2, 32.1, 31.7, 31.6, 31.5, 30.9, 30.8, 30.7, 30.6, 30.6, 30.6, 30.5, 29.0, 28.8, 28.8, 28.7, 28.6, 28.5, 28.4, 28.3, 28.2 ppm. HRMS (FD+) *m*/*z*: M^•+^ calculated for C_52_H_70_O_6_ 790.5167; found 790.5173 (1 ppm).

#### Precatenane Precursor (**12b**)

**12a** (1.3 g,1 equiv, 1.7 mmol) was dissolved in THF (20 mL) and the mixture was purged with nitrogen. Palladium on carbon (10% by weight Pd, 0.20 g) was added and the mixture was heated to 50 °C for 16 h under a dihydrogen atmosphere (balloon). After cooling to room temperature, the mixture was filtered over Celite and washed with THF. The obtained solid **12b** (1.3 g, quantitative) after evaporation was pure according to ^1^H NMR clean and no further purification was needed. In some batches a highly fluorescent, yellow impurity was present that could be washing with some methanol. ^1^H NMR (CDCl_3_, 400 MHz) δ 10.99 (br s, 1H), 8.78 (br s, 1H), 7.50–7.41 (3 × br s, 2H), 7.21 (s, 2H), 7.14 (s, 2H), 2.57–2.45 (m, 8H), 1.68–1.53 (m, 8H), 1.40–1.02 (m, 50H) ppm. ^13^C­{^1^H} NMR (CDCl_3_, 101 MHz) δ 168.6, 168.4, 163.8, 163.7, 163.6, 159.4, 150.9, 149.2, 149.2, 145.2, 145.1, 143.2, 143.1, 137.4, 137.3, 134.6, 134.5, 134.4, 125.9, 125.8, 125.4, 125.3, 110.2, 109.9, 102.6, 102.6, 34.7, 34.5, 31.6, 31.6, 30.9, 30.7, 30.6, 30.5, 29.9, 29.9, 29.5, 29.4, 29.3, 29.2, 29.0, 29.0, 28.9, 28.3, 28.2, 28.2, 28.1 ppm. HRMS (FD+) *m*/*z*: M^•+^ calculated for C_52_H_74_O_6_ 794.5478; found 794.5476 (0.3 ppm).

#### Precatenane Precursor (**13**)

An oven-dried 25 mL round-bottom flask was loaded with **12b** (0.52 g, 1 equiv, 0.65 mmol), dry DMF (5 mL) and potassium carbonate (0.23 g, 2.5 equiv, 1.6 mmol) and the resulting mixture was stirred for 15 min to give the yellow colored phenolate. 13-Bromotridec-1-ene (0.38 g, 2.2 equiv, 1.4 mmol) was added using a syringe and the mixture was stirred at 80 °C for 3 days. After cooling to room temperature, the mixture was poured into water (50 mL) and the round-bottom flask was rinsed with water (2 × 25 mL) and DCM (2 × 25 mL). The mixture was separated and the water layer was washed with DCM (3 × 25 mL), dried over anhydrous magnesium sulfate, filtered and evaporated. The residue was chromatographed over silica using a mixture of DCM and pentane (1:1) as the eluent to afford the pure product **13** (0.63 g, 83%) as a thick colorless oil. ^1^H NMR (CDCl_3_, 400 MHz) δ 8.07, 7.53, 7.45 (3 x br s, 2H), 7.15 (s, 2H), 7.15 (s, 2H), 5.88–5.77 (m, 2H), 5.02–4.93 (m, 4H), 4.15 (br s, 4H), 2.78–2.38 (m, 8H), 2.08–2.02 (m, 4H), 1.86 (brm, 4H), 1.68–1.56 (m, 8H), 1.49–0.86 (m, 88H) ppm. ^13^C­{^1^H} NMR (CDCl_3_, 101 MHz) δ 149.2, 148.6, 145.1, 145.0, 139.4, 139.3, 134.7, 134.6, 125.4, 125.0, 114.2, 106.9, 69.6, 34.5, 34.5, 34.0, 31.7, 31.6, 30.8, 30.6, 30.2, 30.0, 29.7, 29.7, 29.6, 29.6, 29.5, 29.3, 29.3, 29.2, 29.1, 28.9, 28.0, 26.0, 25.9 ppm. HRMS (FD+) *m*/*z*: M^•+^ calculated for C_78_H_122_O_6_ 1154.9236; found 1154.9208 (2 ppm).

#### Precatenane (**14a**)

**13** (0.25 g, 0.22 mmol, 1 equiv) was dissolved in toluene (0.11 L) and degassed for 30 min with argon. Grubbs catalyst M204 (93 mg, 0.11 mmol, 0.5 equiv) was added and the solution was stirred at reflux for 1 day. After evaporation of the solvent the residue was purified by column chromatography over silica using a mixture of pentane and dichloromethane (2:1) as the eluent to afford **14a** as a white solid (0.13 g, 53%). ^1^H NMR (CDCl_3_, 400 MHz) δ 7.49 (s, 1H), 7.43 (s, 1H), 7.17–7.08 (m, 4H), 5.41–5.34 (m, 2H), 4.29–4.13 (m, 4H), 3.14–0.83 (m, 118H) ppm. ^13^C­{^1^H} NMR (CDCl_3_, 101 MHz) δ 164.6, 149.2, 148.6, 145.1, 145.0, 135.0, 134.7, 134.6, 134.4, 134.2, 130.4, 130.0, 125.4, 125.3, 125.0, 69.2, 41.5, 36.2, 34.5, 34.5, 33.9, 32.6, 32.6, 31.7, 31.6, 30.7, 30.4, 30.1, 30.0, 29.8, 29.8, 29.5, 29.4, 29.3, 29.3, 29.2, 29.2, 28.9, 28.1, 28.0, 27.8, 26.5, 26.3, 26.0, 22.7, 20.6, 19.6, 18.9, 14.4, 11.6 ppm. HRMS (FD+) *m*/*z*: M^•+^ calculated for C_76_H_118_O_6_ 1126.8923; found 1126.8935 (1 ppm). Compound contaminated with –(CH_2_)*_n_*– truncated macrocycles.

#### Precatenane (**14b**)

**14a** (0.13 g, 0.115 mmol, 1 equiv) was dissolved in tetrahydrofuran (12 mL), and degassed for 30 min. Palladium on carbon (10% by weight Pd, 0.11 g) was added and the mixture was placed under a hydrogen atmosphere (balloon) and stirred at 50 °C for 1 day. The reaction mixture was filtrated over Celite and evaporated. The residue was purified by column chromatography over silica using a mixture of pentane and dichloromethane (2:1) to afford **14b** (0.11 g, 88%) as a white solid. ^1^H NMR (CDCl_3_, 400 MHz) δ 7.49 (s, 1H), 7.43 (s, 1H), 7.17–7.09 (4*s*, 4H), 4.41–3.92 (m, 4H), 3.27–0.57 (m, 130H) ppm. ^13^C­{^1^H} NMR (CDCl_3_, 101 MHz) δ 164.4, 149.0, 148.4, 144.9, 144.9, 134.7, 134.5, 134.4, 134.2, 134.0, 125.2, 125.1, 124.7, 106.7, 105.6, 69.1, 69.0, 34.3, 34.3, 31.9, 31.5, 31.4, 30.6, 30.2, 29.9, 29.7, 29.6, 29.5, 29.4, 29.3, 29.2, 29.2, 29.1, 29.0, 28.7, 28.1, 27.8, 27.7, 27.6, 26.3, 26.1, 22.6 ppm. HRMS (FD+) *m*/*z*: M^•+^ calculated for C_76_H_120_O_6_ 1128.9079; found 1128.9058 (2 ppm).

#### Precatenane (**15a**)

**14b** (20 mg, 0.018 mmol, 1 equiv) was dissolved in 1,4-dioxane (2 mL), aqueous KOH (4M, 0.25 mL) and methanol (0.5 mL). The solution was heated to 110 °C to evaporate the methanol and the vessel was closed and heated to 150 °C for 4 h. After cooling to room temperature, the mixture was evaporated. The residue was acidified with aqueous KHSO_4_ (1M) and extracted with ethyl acetate, dried over anhydrous magnesium sulfate, filtered and evaporated to dryness affording **15a** in quantitative yield (20 mg) and used in the next step without further purification. ^1^H NMR (CDCl_3_, 400 MHz) δ 7.29 (s, 2H), 7.14 (S, 1H), 7.06 (S, 1H), 7.01 (S, 2H), 4.15 (s, 2H), 4.07 (s, 2H), 2.63 (s, 8H), 1.79–0.71 (m, 126H) ppm. No ^13^C NMR was measured due to the low solubility. HRMS (FD+) calculated for C_76_H_122_O_7_ M^•+^ 1146.9186, found 1146.9230 (4 ppm).

#### Precatenane (**15b**)

**15a** (20 mg, 0.018 mmol, 1 equiv) was dissolved in tetrahydrofuran (1 mL). A freshly prepared solution of HCl was obtained by adding acetyl chloride (0.3 mL, 4 mmol) to methanol (2 mL) that was subsequently added to the THF solution followed by stirring for 1 day at room temperature. After evaporation, **15b** was obtained in quantitative yield (20 mg) as a white solid. ^1^H NMR (CDCl_3_, 400 MHz) δ 7.37 (br s, 2H), 7.17 (s, 1H), 7.08 (s, 1H), 7.02 (s, 1H), 7.00 (s, 1H), 4.17 (br s, 4H), 3.94 (s, 3H), 2.67 (s, 8H), 1.98–0.74 (m, 122H) ppm. No ^13^C NMR was measured due to low solubility. HRMS (FD+) *m*/*z*: M^•+^ calculated for C_77_H_124_O_7_ 1160.9342; found 1160.9360 (2 ppm).

#### Catenane (**16**)

Precatenane **14b** (10 mg, 1 equiv, 8.9 μmol) was dissolved in THF (2 mL) and lithium aluminum hydride (8.4 mg, 25 equiv, 0.22 mmol) was added and the mixture was stirred at 50 °C for 3 days in a closed vessel. After cooling to room temperature, the mixture was poured in ice water (20 mL) and extracted with DCM (3 × 10 mL), dried over anhydrous magnesium sulfate, filtered and evaporated. The residue was purified by column chromatography over silica using a mixture of DCM and pentane (1:1) as the eluent. Catenane **16** was obtained as a colorless oil (90%, 9 mg). ^1^H NMR (CDCl_3_, 400 MHz) δ 6.94 (br s, 2H), 6.92 (br s, 2H), 6.54 (br s, 2H), 5.26 (br s, 2H), 4.83 (br m, 2H), 4.59 (br s, 2H), 4.13–3.99 (br m, 8H), 3.65–3.60 (br m, 4H), 2.56–2.47 (m, 8H), 1.79–0.88 (m, 132H). No ^13^C­{^1^H} NMR was measured due to low solubility. HRMS (FD+) *m*/*z*: M^•+^ calculated for C_76_H_120_O_6_ 1128.9079; found 1128.9034 (4 ppm). Compound contaminated with -(CH_2_)_n_- truncated macrocycles.

#### (2-Hydroxy-5-methoxy-1,3-phenylene)­dimethanol (**18**)

A 1L round-bottom flask was charged with water (400 mL), NaOH (40 g, 1.6 equiv, 1.0 mol), 4-methoxyphenol (**17**) (80 g, 1 equiv, 64 mmol) and paraformaldehyde (70 g, 3.6 equiv, 2.3 mol) and stirred vigorously for 5 days at room temperature. The resulting reaction mixture showed an orange color. The pH was adjusted to 5–6 with concentrated hydrochloric acid (37% aq.). The mixture was poured in a separatory funnel and an additional 600 mL of water was added and was washed with ethyl acetate (5 × 1 L). The combined organic layers were dried over anhydrous magnesium sulfate, filtered over a silica plug and washed with ethyl acetate. The solvent was evaporated and (2-hydroxy-5-methoxy-1,3-phenylene)­dimethanol **18** (118 g, 0.64 mol, quantitative) was obtained as a white solid. The product can be recrystallized from boiling ethyl acetate if impurities are present. ^1^H NMR (DMSO, 400 MHz) δ 8.01 (br s, 1H), 6.74 (s, 2H), 5.19 (s, 2H), 4.51 (s, 4H), 3.67 (s, 3H) ppm. The spectral data were in accordance with literature data.^16^

#### 2-Hydroxy-5-methoxyisophthalaldehyde (**19**)

A 2L round-bottom flask was charged with (2-hydroxy-5-methoxy-1,3-phenylene)­dimethanol **18** (80 g, 1 equiv, 0.43 mol) and chloroform (1,5 L) and the mixture was stirred vigorously. Manganese dioxide (320 g, 8.5 equiv, 3.7 mol) was added in portions to prevent boiling of the mixture and once everything was added the mixture was heated at reflux for 16 h. After cooling to room temperature, Celite (500 gr) and anhydrous magnesium sulfate (100 gr) were added to the mixture and stirred for 10 min and filtered over a Celite plug. The black residue was washed with acetone until no yellow color ran off the plug. The solvent was evaporated and the residue was purified by column chromatography using silica and chloroform with 1% THF as the eluent to obtain 2-hydroxy-5-methoxyisophthalaldehyde **19** (69 g, 0.38 mol, 88%) as a bright yellow fluffy solid. The product can be recrystallized from boiling chloroform to obtain yellow needles. ^1^H NMR (CDCl_3_, 400 MHz) δ 11.12 (s, 1H), 10.22 (s, 2H), 7.51 (s, 2H), 3.86 (s, 3H) ppm. Spectral data were in accordance with literature data.^17^

#### 1,1′-(2-Hydroxy-5-methoxy-1,3-phenylene)­bis­(hept-6-en-1-ol) (**20**)

To a 1L three-necked round-bottom flask was added magnesium (12 g, 4.5 equiv, 0.50 mol) and the flask was flame-dried under vacuum while stirring. After cooling to room temperature, dry THF (500 mL) was added and the dripping funnel was loaded with 6-bromohex-1-ene (82 g, 67 mL, 4.5 equiv, 0.50 mol) and dry THF (80 mL). A first batch of 10–15 mL of the halide was added to start the reaction. After the Grignard reaction started, the rest of the mixture was slowly added over the course of half an hour. After complete addition, the mixture was warmed to 50 °C for 16 h. After cooling down in an ice bath, 2-hydroxy-5-methoxyisophthalaldehyde **19** (20 g, 1 equiv, 0.11 mol) was added as a solid to the mixture and after complete addition the mixture was heated to reflux for 24 h. After cooling to room temperature, the mixture was quenched with 100 mL of a saturated ammonium chloride solution and diluted with 1L of water. The product was extracted with ethyl acetate (4 × 750 mL), dried over anhydrous magnesium sulfate and filtered over a small layer of silica. The solvent was evaporated to obtain 1,1’-(2-hydroxy-5-methoxy-1,3-phenylene)­bis­(hept-6-en-1-ol) **20** (39 g, 0.11 mol, quantitative) as a mixture of diastereomers as a thick oil. The product was used without any further purification in the next step. ^1^H NMR (CDCl_3_, 400 MHz) δ 8.37 (s, 0.5H), 8.29 (s, 0.5H), 6.48 (d, 2H, *J* = 12 Hz), 5.84–5.73 (m, 2H), 5.01–4.90 (m, 4H), 4.73–4.64 (m, 2H), 3.72 (s, 3H), 3.67 (br s, 1H), 3.52 (br s, 1H), 2.03 (q, 4H), 1.85–1.70 (m, 4H), 1.50–1.28 (m, 8H) ppm. ^13^C­{^1^H} NMR (CDCl_3_, 101 MHz) δ 152.4, 152.3, 147.0, 147.0, 139.0, 130.6, 130.5, 114.5, 111.7, 111.5, 74.5, 73.6, 55.9, 55.9, 37.05, 36.9, 33.8, 33.8, 28.8, 28.8, 25.6 ppm. HRMS (FD+) *m*/*z*: M^•+^ calculated for C_21_H_32_O_4_ 348.2295; found 348.2312 (5 ppm).

#### 2,6-Di­(hept-6-en-1-yl)-4-methoxyphenol (**21**)

A flame-dried 1L round-bottom flask was charged with 1,1’-(2-hydroxy-5-methoxy-1,3-phenylene)­bis­(hept-6-en-1-ol) **20** (39 g, 1 equiv, 0.11 mol) and dry DCM (600 mL) and the mixture was cooled down to −80 °C with dry ice and acetone. Triethylsilane (58 g, 80 mL, 4.5 equiv, 0.50 mol) was added under vigorously stirring, followed by the slow addition of boron trifluoride etherate (71 g, 62 mL, 4.5 equiv, 0.50 mol) over the course of 10 min. The reaction was stirred overnight, meanwhile allowing the mixture to warm to room temperature. Water (250 mL) was added to the reaction mixture followed by stirring for 30 min. The mixture was added to a separation funnel and washed with DCM (2 × 250 mL). The combined organic fractions were dried over anhydrous magnesium sulfate, filtered over a thick silica plug and evaporated. The residue was purified by chromatography over silica with a mixture of pentane and ethyl acetate (95:5) as the eluent to obtain 2,6-di­(hept-6-en-1-yl)-4-methoxyphenol **21** (34 g, 0.11 mol, 98%) as a yellow oil. ^1^H NMR (CDCl_3_, 400 MHz) δ 6.58 (s, 2H), 5.90–5.80 (m, 2H), 5.06–4.96 (m, 4H), 4.43 (s, 1H), 3.78 (s, 3H), 2.60 (t, 4H, *J* = 8 Hz), 2.10 (q, 4H, *J* = 8 Hz), 1.69–1.62 (m, 4H), 1.50–1.40 (m, 8H) ppm. ^13^C­{^1^H} NMR (CDCl_3_, 101 MHz) δ 153.2, 145.4, 139.1, 129.4, 114.4, 112.9, 55.7, 33.8, 30.5, 29.7, 29.1, 28.9 ppm. HRMS (FD+) *m*/*z*: M^•+^ calculated for C_21_H_32_O_2_ 316.2397; found 316.2391 (2 ppm).

#### Bis­(2,6-di­(hept-6-en-1-yl)-4-methoxyphenyl) 2,6-bis­(allyloxy)­terephthalate (**22a**)

A flame-dried round-bottom flask was charged with 2,6-di­(hept-6-en-1-yl)-4-methoxyphenol **21** (6.3 g, 2 equiv, 20 mmol), dry 1,4-dioxane (150 mL) and cesium carbonate (13 g, 4 equiv, 40 mmol) (dried in an oven at 150 °C overnight). The resulting solution was stirred for 30 min at room temperature under nitrogen. Bis­(perfluorophenyl) 2,6-bis­(allyloxy)­terephthalate **9** (6.1 g, 1 equiv, 10 mmol) was added as a solid to the solution and the mixture was stirred at reflux (set for 110 °C) for 4 days. After cooling to room temperature, cyclohexane was added (twice the reaction volume; 300 mL) and stirred for 5 min. The mixture was filtered over a mixture of Celite and silica and washed with cyclohexane. Evaporation to dryness gave a brown thick oil (8.7 g, 86%) that appeared to be pure according to NMR analysis and was used without further purification. ^1^H NMR (CDCl_3_, 400 MHz) δ 7.46 (s, 2H), 6.67 (s, 4H), 6.09–5.99 (m, 2H), 5.84–5.68 (m, 4H), 5.45–5.39 (m, 2H), 5.32–5.28 (m, 2H), 4.98–4.88 (m, 4H), 4.75–4.72 (m, 4H), 3.81 (2*s*, 3H), 3.81 (s, 3H), 2.69 (br s, 4H), 2.45 (t, 4 H, *J* = 12 Hz), 2.02 (br m, 8H), 1.59 (m, 8H), 1.36 (m, 16H) ppm. ^13^C­{^1^H} NMR (CDCl_3_, 101 MHz) δ 164.4, 164.4, 157.4, 157.4, 156.7, 141.1, 140.9, 139.1, 138.9, 136.2, 135.8, 132.3, 131.9, 118.7, 118.3, 114.4, 114.3, 112.9, 112.6, 107.1, 70.0, 60.4, 55.5, 55.4, 53.5, 33.8, 33.7, 30.8, 30.1, 29.9, 29.0, 29.0, 28.9, 28.8 ppm. HRMS (FD+) *m*/*z*: M^•+^ calculated for C_56_H_74_O_8_ 874.5378; found 874.5380 (0.2 ppm).

#### Bis­(2,6-di­(hept-6-en-1-yl)-4-methoxyphenyl) 2,6-dihydroxyterephthalate (**22b**)

To a flame-dried round-bottom flask containing dry 1,4-dioxane (50 mL) was added bis­(2,6-di­(hept-6-en-1-yl)-4-methoxyphenyl) 2,6-bis­(allyloxy)­terephthalate **22a** (5.0 g, 1 equiv, 5.7 mmol), diethylamine (1.7 g, 2.4 mL, 4 equiv, 23 mmol) and tetrakis­(triphenylphosphine)­palladium(0) (0.33 g, 0.05 equiv, 0.29 mmol) under a nitrogen atmosphere. The reaction was stirred at room temperature and after complete conversion (1 h), a saturated ammonium chloride solution (20 mL, aq.) was added and stirred for 5 min. The solution was transferred to a separation funnel and washed with water (100 mL) and ethyl acetate (3 × 100 mL). The combined organic layers were dried over anhydrous magnesium sulfate and evaporated. The residue was purified by column chromatography over a silica plug using pentane and ethyl acetate (4:1) as the eluent to obtain bis­(2,6-di­(hept-6-en-1-yl)-4-methoxyphenyl) 2,6-dihydroxyterephthalate **22b** (4.5 g, 5.6 mmol, 98%) as a clear liquid. ^1^H NMR (CDCl_3_, 400 MHz) δ 9.79 (br s, 2H), 7.38 (s, 2H), 6.71 (s, 2H), 6.66 (s, 2H), 5.83–5.68 (m, 4H), 4.99–4.88 (m, 8H), 3.83 (s, 3H), 3.81 (s, 3H), 2.45 (q, 8H, *J* = 12 Hz), 2.00 (q, 8H, *J* = 12 Hz), 1.60 (quint, 8H, *J* = 8 Hz), 1.34 (m, 16H) ppm. ^13^C­{^1^H} NMR (CDCl_3_, 101 MHz) δ 168.6, 164.0, 161.6, 158.5, 157.5, 141.1, 139.1, 139.0, 138.8, 137.2, 135.9, 135.8, 114.5, 114.3, 113.2, 112.9, 110.0, 102.7, 55.7, 55.6, 33.8, 33.7, 33.7, 30.7, 30.6, 30.6, 29.9, 29.9, 29.0, 29.0, 28.8, 28.7 ppm. HRMS (FD+) *m*/*z*: M^•+^ calculated for C_50_H_66_O_8_ 794.4752; found 794.4763 (1 ppm).

#### Precatenane Precursor (**23a**)

A flame-dried round-bottom flask was charged with bis­(2,6-di­(hept-6-en-1-yl)-4-methoxyphenyl) 2,6-dihydroxyterephthalate **22b** (5.3 g, 1 equiv, 6.7 mmol) and toluene (0.67 L). The mixture was degassed by three N_2_/vacuum cycles. Grubbs second generation catalyst (0.57 g, 0.1 equiv, 0.67 mmol) was added followed by an additional three N_2_/vacuum cycles before the mixture was brought to reflux. After 2 h at reflux, the mixture was cooled down to room temperature, filtered over a silica plug and washed with chloroform. After evaporation, some ligand of Grubbs II catalyst was present that could be removed by adding some methanol and crushing the solids, followed by filtration and drying to obtain **23a** as a white solid (4.8 g, 6.5 mmol, 98%) that was used without further purification. ^1^H NMR (CDCl_3_, 400 MHz) δ 10.95 (s, 1H), 8.77 (s, 1H), 7.43–7.41 (3*s*, 2H), 6.72 (s, 2H), 6.67 (s, 2H), 5.32–5.07 (m, 4H), 3.82 (s, 3H), 3.80 (s, 3H), 2.54–2.38 (m, 8H), 1.88–1.78 (m, 8H), 1.66–1.54 (m, 8H), 1.41–1.19 (m, 16H) ppm. ^13^C­{^1^H} NMR (CDCl_3_, 101 MHz) δ 168.8, 168.7, 164.1, 164.0, 163.6, 159.4, 158.6, 157.6, 141.0, 140.9, 138.8, 138.8, 137.1, 137.0, 136.5, 136.4, 136.4, 136.3, 130.1, 129.9, 129.6, 129.4, 113.8, 113.7, 113.4, 113.3, 110.1, 109.9, 102.5, 102.5, 55.6, 55.5, 32.4, 32.3, 32.1, 30.7, 30.6, 30.5, 30.4, 30.4, 30.3, 28.9, 28.7, 28.7, 28.5, 28.4, 28.3, 28.1 ppm. HRMS (ESI+) *m*/*z*: [M + H]^+^ calculated for C_46_H_59_O_8_ 739.4205; found 739.4212 (1 ppm).

#### Precatenane Precursor (**23b**)

A round-bottom flask was loaded with **23a** (2.9 g, 1 equiv, 3.9 mmol) and tetrahydrofuran (270 mL). The mixture was put under a nitrogen flow after three N_2_/vacuum cycles before palladium on carbon (42 mg, 10% Wt, 0.01 equiv, 39 μmol) was added. An additional three N_2_/vacuum cycles were applied, and the mixture was connected to a balloon of hydrogen. The mixture was stirred at 50 °C overnight. After cooling to room temperature, three N_2_/vacuum cycles before the mixture was filtered over Celite and washed with THF followed by evaporation of the volatiles. Sometimes a bright fluorescent impurity was present in the crude that could be washed away with some methanol and grinding of the solids, followed by filtration and drying. No further purification was required obtaining **23b** (2.9 g, 3.9 mmol, quantitative) as a white solid. ^1^H NMR (CDCl_3_, 400 MHz) δ 10.93 (s, 1H), 8.75 (s, 1H), 7.48–7.38 (3*s*, 2H), 6.71 (s, 2H), 6.66 (s, 2H), 3.82 (s, 3H), 3.80 (s, 3H), 2.53–2.36 (m, 8H), 1.65–1.51 (m, 8H), 1.37–1.27 (m, 8H), 1.22–0.98 (m, 16H) ppm. ^13^C­{^1^H} NMR (CDCl_3_, 101 MHz) δ 168.8, 168.7, 164.1, 163.6, 159.4, 158.6, 157.6, 140.9, 138.9, 137.3, 137.2, 136.6, 136.5, 136.5, 136.3, 113.7, 113.6, 113.3, 113.2, 110.1, 109.9, 102.6, 55.7, 55.6, 30.7, 30.6, 30.5, 30.4, 30.4, 30.0, 29.6, 29.5, 29.3, 29.2, 29.1, 29.0, 28.9, 28.9, 28.3, 28.2, 28.2, 28.0 ppm. HRMS (FD+) *m*/*z*: M^•+^ calculated for C_46_H_62_O_8_ 742.4439; found 742.4454 (2 ppm).

#### Precatenane Precursor (**24**)

A flame-dried 25 mL round-bottom flask was charged with **23b** (1.1 g, 1 equiv, 1.5 mmol), DMF (5 mL, dry) and potassium carbonate (0.51 g, 2.5 equiv, 3.7 mmol, dried in an oven at 150 °C overnight) and the resulting mixture was stirred for 15 min to give the yellow colored phenolate. 13-Bromotridec-1-ene 16 (0.85 g, 2.2 equiv, 3.3 mmol) was added via syringe and the mixture was stirred at 80 °C for 3 days. After cooling to room temperature, the mixture was poured into water (50 mL) and the round-bottom flask was rinsed with water (2 × 25 mL) and DCM (2 × 25 mL). The layers were separated and the water layer was washed with DCM (3 × 25 mL), dried over anhydrous magnesium sulfate, filtered and evaporated. The residue was purified by column chromatography over silica using a mixture of DCM and pentane (1:1) as the eluent to afford compound **24** as a thick oil (1.4 g, 1.2 mmol, 84%). ^1^H NMR (CDCl_3_, 400 MHz) δ 7.52 (br s, 1H), 7.44 (s, 1H), 6.69 (s, 2H), 6.68 (s, 2H), 5.87–5.77 (m, 2H), 5.03–4.91 (m, 4H), 4.19–4.10 (m, 4H), 3.81 (s, 3H), 3.80 (s, 3H), 2.76 (br s, 2H), 2.56–2.49 (m, 4H), 2.43–2.35 (m, 2H), 2.07–2.01 (m, 4H), 1.86 (br m, 4H), 1.68–1.54 (m, 8H), 1.48–1.42 (m, 4H), 1.38–1.24 (m, 44H), 1.12 (br s, 8H), 1.01 (br s, 8H) ppm. ^13^C­{^1^H} NMR (CDCl_3_, 101 MHz) δ 157.6, 157.5, 141.0, 139.4, 139.3, 136.7, 136.5, 114.2, 113.4, 113.0, 106.9, 69.9, 55.5, 55.5, 33.9, 30.6, 30.5, 30.3, 30.1, 29.9, 29.7, 29.7, 29.7, 29.6, 29.6, 29.5, 29.5, 29.3, 29.2, 29.1, 29.0, 28.9, 27.9, 26.0 ppm. HRMS (FD+) *m*/*z*: M^•+^ calculated for C_72_H_110_O_8_ 1102.8195; found 1102.8218 (2 ppm).

#### Precatenane (**25a**)

An oven-dried 1L round-bottom flask was charged with **24** (0.73 g, 1 equiv, 0.66 mmol) and toluene (0.66 L). The mixture was degassed by three N_2_/vacuum cycles before Grubbs second generation catalyst (0.11 g, 0.2 equiv, 0.13 mmol) was added, followed by an additional three N_2_/vacuum cycles. The mixture was refluxed for 1 day under a nitrogen atmosphere. After cooling to room temperature, the mixture was filtered over a silica plug, washed with chloroform and evaporated. The crude was purified by column chromatography using silica and chloroform as the eluent to obtain **25a** (0.61 g, 0.57 mmol, 86%) as a white solid. ^1^H NMR (CDCl_3_, 400 MHz) δ 7.52 (s, 1H), 7.46 (s, 1H), 6.74–6.67 (m, 4H), 5.46–5.36 (m, 2H), 4.34–4.17 (m, 4H), 3.83 (s, 3H), 3.82 (s, 3H), 3.17–0.89 (m, 88H) ppm. ^13^C­{^1^H} NMR (CDCl_3_, 101 MHz) δ 157.5, 157.4, 140.9, 136.5, 130.3, 113.3, 106.8, 69.1, 55.4, 55.3, 32.5, 30.0, 29.7, 29.7, 29.5, 29.4, 29.2, 29.1, 27.8, 26.4, 26.2 ppm. HRMS (FD+) *m*/*z*: M^•+^ calculated for C_70_H_106_O_8_ 1074.7882; found 1074.7903 (2 ppm).

#### Precatenane (**25b**)

To a round-bottom flask was added **25a** (0.33 g, 1 equiv, 0.30 mmol) and THF (10 mL). The mixture was put under nitrogen flow by three N_2_/vacuum cycles before palladium on carbon (15 mg, 10% Wt, 0.05 equiv, 14 μmol) was added. An additional three N_2_/vacuum cycles were applied and the mixture was connected to a balloon of hydrogen. The mixture was stirred at 50 °C overnight. After cooling to room temperature, three N_2_/vacuum cycles were applied to remove all dihydrogen gas before the mixture was filtered over Celite and washed with THF, followed by evaporation of the volatiles. The bright fluorescent impurity could be washed away with some methanol and grinding of the solids, followed by filtration and drying. No further purification was required and **25b** (0.33 g, 0.30 mmol, quant.) was obtained as a white solid. ^1^H NMR (CDCl_3_, 400 MHz) δ 7.50 (s, 1H), 7.44 (s, 1H), 6.72–6.65 (m, 4H), 4.31–4.14 (m, 4H), 3.81 (s, 3H), 3.80 (s, 3H), 3.15–0.85 (m, 92H) ppm. ^13^C­{^1^H} NMR (CDCl_3_, 101 MHz) δ 164.9, 157.6, 157.5, 141.0, 137.0, 136.6, 136.4, 136.2, 113.4, 113.3, 113.1, 106.9, 105.7, 77.4, 69.3, 55.5, 55.5, 30.5, 30.2, 29.8, 29.8, 29.8, 29.7, 29.5, 29.5, 29.4, 29.3, 29.3, 29.2, 28.0, 27.9, 26.5, 26.3 ppm. HRMS (FD+) *m*/*z*: M^•+^ calculated for C_70_H_108_O_8_ 1076.8039; found 1076.8037 (0.2 ppm).

### The Synthesis Protocols and Spectroscopic Data of the Model Compounds **27**, **28**, **29**, **31**, **32**, **33** and the Oxidation Reactions toward Quinone **30** Can Be Found in the Supporting Information

#### Catenane (**34**)

A pressure tube was loaded with **25b** (21 mg, 0.019 mmol, 1 equiv) and THF (dry, 0.5 mL). To this solution, lithium aluminum hydride (18 mg, 0.49 mmol, 25 equiv) was added and the pressure tube was closed. The contents of tube were stirred and heated to 50 °C for 5 days. After cooling to room temperature, the mixture was poured into water (25 mL) and extracted with DCM (3 × 25 mL), dried over anhydrous magnesium sulfate, filtered and evaporated. The residue was purified by chromatography over silica using a mixture of ethyl acetate and pentane (1:1) as the eluent to afford **34** (19 mg, 90%) as a liquid. ^1^H NMR (CDCl_3_, 400 MHz) δ 6.49 (br s, 6H), 5.13 (br s, 2H), 4.80 (s, 2H), 4.60 (s, 2H), 3.92 (t, 4H, *J* = 8 Hz), 3.73 (s, 6H), 2.50 (br m, 8H), 1.68 (m, 4H), 1.52 (br m, 8H), 1.40–1.10 (m, 78H) ppm. ^13^C­{^1^H} NMR (CDCl_3_, 101 MHz) δ 157.9, 153.0, 145.6, 142.2, 116.4, 112.7, 103.3, 68.1, 65.6, 55.6, 55.5, 30.8, 30.1, 30.0, 29.7, 29.6, 29.6, 29.5, 29.5, 29.4, 29.4, 29.3, 29.2, 26.3 ppm. HRMS (ESI+) *m*/*z*: [M + Na]^+^ calculated for C_70_H_116_O_8_Na 1107.8562; found 1107.8666 (9 ppm).

## Supplementary Material



## Data Availability

The data underlying this study are available in the published article and its Supporting Information.

## Notes

Synthesis protocols and spectroscopic data of the model compounds **27**, **28**, **29**, **31**, **32**, **33** and **30** together with copies of the ^1^H-, ^13^C NMR and mass spectra of these compounds and all others (PDF)
