# Supplementary material for: Covalent Templated Catenane Synthesis Requiring Hindered Ester Cleavage
Source: J Org Chem. 2026 Aug 4;91(32):11116–26. doi: 10.1021/acs.joc.6c01398 (PMC13488437; doi:10.1021/acs.joc.6c01398)
Supplement: Supplementary file 1 [file jo6c01398_si_001.pdf]

Supporting Information

Covalent templated catenane synthesis requiring hindered ester cleavage

Nick Westerveld, Martin J. Wanner and Jan H. van Maarseveen*

Van ‘t Hoff Institute for Molecular Sciences, University of Amsterdam, Science Park 904, 1098 XH Amsterdam, The Netherlands

Table of contents

	Page
**Synthesis protocols for model compounds 27, 28, 29, 31, 32, 33 and their oxidation reactions towards quinone 30.**	**S3-S5**
**Synthesis of compound 27**	**S3**
**Synthesis of compound 28**	**S3**
**Synthesis of compound 29**	**S3**
**Synthesis of compound 31**	**S4**
**Synthesis of compound 32**	**S4**
**Synthesis of compound 33**	**S5**
**Synthesis of compound 30**	**S5**
**Copies of the ^{1}H-, ^{13}C and high-resolution mass spectra**	**S6-S58**
**13-Bromotridec-1-ene**	**S6**
**Precatenane precursor 2**	**S7**
**Precatenane 3a**	**S8**
**Precatenane 3b**	**S9**
**Catenane 4**	**S10**
**2,6-Dihydroxyterephthalic acid (6)**	**S11**
**Diallyl 2,6-bis(allyloxy)terephthalate (7)**	**S12**
**2,6-Bis(allyloxy)terephthalate (8)**	**S14**
**Bis(perfluorophenyl) 2,6-bis(allyloxy)terephthalate (9)**	**S15**
**Bis(2,6-di(hept-6-en-1-yl)-4-tert-butylphenyl) 2,6-bis(allyloxy)terephthalate (11a)**	**S17**
**Bis(4-tert-butyl)-2,6-di(hept-6-en-1-yl) 2,6-dihydroxyterephthalate (11b)**	**S19**
**Precatenane precursor 12a**	**S20**
**Precatenane precursor 12b**	**S22**
**Precatenane precursor 13**	**S23**
**Precatenane 14a**	**S25**
**Precatenane 14b**	**S26**
**Precatenane 15a**	**S28**
**Precatenane 15b**	**S29**
**Catenane 16**	**S30**
**(2-Hydroxy-5-methoxy-1,3-phenylene)dimethanol (18)**	**S32**
**2-Hydroxy-5-methoxyisophthalaldehyde (19)**	**S32**
**1,1’-(2-Hydroxy-5-methoxy-1,3-phenylene)bis(hept-6-en-1-ol) (20)**	**S33**
**2,6-di(hept-6-en-1-yl)-4-methoxyphenol (21)**	**S34**
**Compound 22a**	**S36**
**Compound 22b**	**S37**
**Precatenane precursor 23a**	**S39**
**Precatenane precursor 23b**	**S40**
**Precatenane precursor 24**	**S42**
**Precatenane 25a**	**S43**
**Precatenane 25b**	**S45**
**2,6-Di(hept-6-en-1-yl)-4-methoxyphenyl acetate (27)**	**S46**
**2,6-Di(hept-6-en-1-yl)-4-methoxyphenyl benzoate (28)**	**S48**
**Bis(2,6-di(hept-6-en-1-yl)-4-methoxyphenyl) 2,6-dimethoxyterephthalate (31)**	**S51**
**Bis(2,6-di(hept-6-en-1-yl)-4-methoxyphenyl) terephthalate (32)**	**S52**
**bis(2,6-di(hept-6-en-1-yl)-4-methoxyphenyl) cyclohexane-1,4-dicarboxylate (33)**	**S54**
**2,6-di(hept-6-en-1-yl)cyclohexa-2,5-diene-1,4-dione (30)**	**S55**
**Catenane 34**	**S57**

Synthesis protocols for model compounds 27, 28, 29, 31, 32, 33 and their oxidation reactions towards quinone 30.

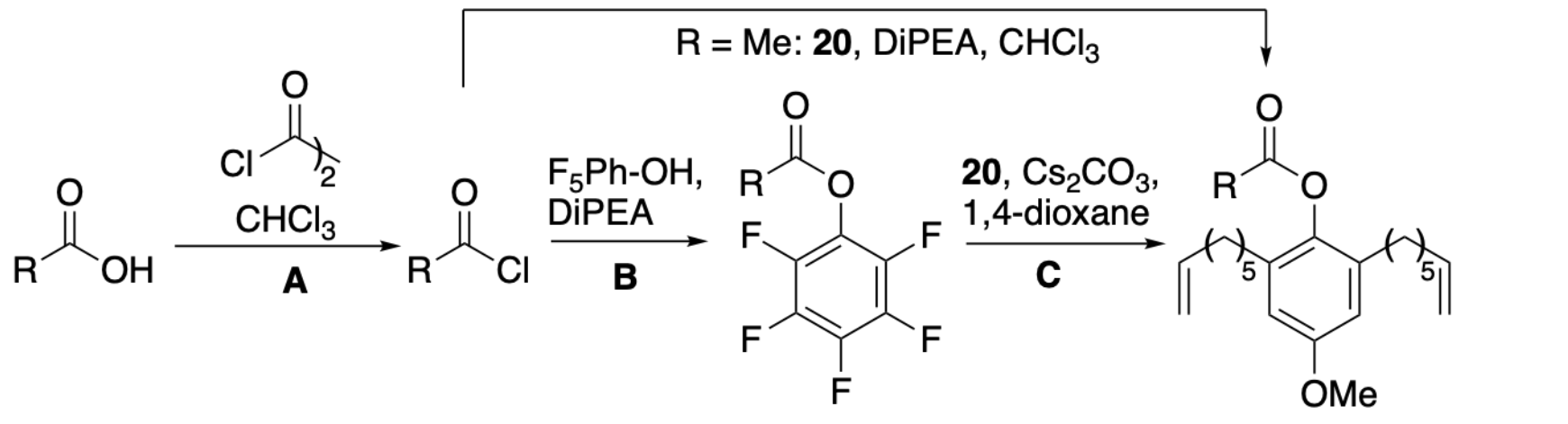

27: R = Me
28: R = Ph
29: R = 2,6-OMe-Ph

*General procedure **A** for the synthesis of the acyl chloride.* A roundbottom flask was charged with the carboxylic acid (1 mmol, 1.0 equiv) and chloroform (2.5 mL). Oxalyl chloride (1.2 equiv per carboxylic acid moiety) was added under stirring and a drop of DMF was added as a catalyst. The mixture was stirred for 16 hours at room temperature. The mixture was evaporated and the residue was dissolved in DCM and used without further purification in the next step.

*General procedure **B** for the synthesis of the pentafluorophenolic esters.* To a mixture of pentafluorophenol (1.2 equiv per acyl chloride moiety), DIPEA (1.5 equiv per acyl chloride moiety) in chloroform (1 M) was added the acyl chloride (1 mmol, 1 equiv), dissolved in chloroform. The mixture was stirred at reflux overnight. After cooling to room temperature, the mixture was filtered over silica and washed with a 1:1 mixture of chloroform and heptane. The mixture was evaporated and the obtained solid was recrystallized from heptane to obtain the pentafluorophenol esters that were used without further purification.

*General procedure **C** for the transesterification of the pentafluorophenol esters to the methoxyphenol esters **27**, **28** and **29**.* A flame dried roundbottom flask was loaded with Cs_2CO_3 (1.5 equiv per pentafluorophenolic ester moiety) and dry 1,4-dioxane (15 mL). 2,6-Di(hept-6-en-1-yl)-4-methoxyphenol (**20**) (1 equiv per pentafluorophenolic ester moiety) was added and the mixture was stirred for 15 minutes. The pentafluorophenolic ester (1 mmol, 1 equiv) was added and the mixture was stirred for 16 hours at reflux. After allowing the reaction mixture to cool to room temperature, heptane (30 mL) was added, and the mixture was filtered over celite to remove insoluble salts and washed with a 1:2 mixture of 1,4-dioxane and heptane. After evaporation of the solvent, the residue was purified by column chromatography over silica using EtOAc/heptane mixtures to elute the compound.

Synthesis of 27. 2,6-Di(hept-6-en-1-yl)-4-methoxyphenol (**20**) (0.50 g, 1 equiv, 1.6 mmol) was dissolved in chloroform (2 mL). DIPEA (0.55 mL, 2.0 equiv, 3.2 mmol) was added and stirred. Acetyl chloride (0.17 mL, 1.5 equiv, 2.4 mmol) was added and the reaction was stirred for two hours at reflux. After cooling to room temperature, the mixture was transferred to a separatory funnel, and the flask was rinsed with chloroform (2 mL). The organic layer was washed with NaOH (1M in water, 3 x 10 mL), dried over anhydrous magnesium sulphate, filtered and evaporated. The residue was purified by column chromatography using a 1:1 chloroform/pentane mixture to obtain 2,6-di(hept-6-en-1-yl)-4-methoxyphenyl acetate (**27**) as a colorless liquid (0.40 g, 70%).^{1}H NMR ($CDCl_3$ 400MHz) δ 6.66 (s, 2H), 5.90-5.80 (m, 2H), 5.07-4.96 (m, 4H), 3.79 (s, 3H), 2.47 (t, 4H, J = 8.0 Hz), 2.34 (s, 3H), 2.10 (q, 4H, J = 8.0 Hz), 1.63 (q, 4H, J = 8.0 Hz), 1.51-1.38 (m, 8H) ppm. ^{13}C{^{1}H} NMR ($CDCl_3$ 101 MHz) δ 169.5, 157.1, 141.0, 138.8, 135.5, 114.3, 112.6, 55.2, 33.6, 30.6, 29.6, 28.9, 28.6, 20.5 ppm.

ppm. HRMS (FD+) m/z: $M^{\bullet+}$ calculated for $C_{23}H_{34}O_3$ 358.2502; found 358.2520 (5 ppm).

Synthesis of 28. Synthesized from commercial benzoyl chloride following general procedures **B** and **C**. With **B**: pentafluorophenol (0.50 g, 1.2 equiv, 2.7 mmol), benzoyl chloride (0.26 mL, 1.0 equiv, 2.3 mmol) DIPEA (1.0 mL, 2.5 equiv, 5.7 mmol) and chloroform (6 mL). After recrystallizing the pentafluorophenolic ester was obtained as a white solid (0.61 g, 94%). **C:** 2,6-di(hept-6-en-1-yl)-4-methoxyphenol (**20**) (0.50 g, 1 equiv, 1.6 mmol), pentafluorophenyl benzoate (0.46 g, 1 equiv, 1.6 mmol), cesium carbonate (0.62 g, 1.2 equiv, 1.9 mmol). After chromatography over silica using 2.5% EtOAc in heptane, 2,6-di(hept-6-en-1-yl)-4-methoxyphenyl benzoate (**28**) (0.43 g, 65 %) was afforded as a white solid. ^{1}H NMR ($CDCl_3$ 400MHz) δ 8.32 (d, 2H), 7.71-7.66 (m, 1H), 7.57 (t, 2H), 6.75 (s, 2H), 5.84-5.74 (m, 2H), 5.02-4.93 (m, 4H), 3.84 (s, 3H), 2.55 (t, 4H), 2.08-2.02 (m, 4H), 1.72-1.64 (m, 4H), 1.44-1.35 (m, 8H) ppm. ^{13}C{^{1}H} NMR ($CDCl_3$ 101 MHz) δ 165.2, 157.3, 141.2, 138.9, 135.9, 133.5, 130.1, 129.5, 128.7, 114.2, 112.8, 112.7, 55.3, 33.6, 30.7, 29.8, 28.9, 28.6 ppm. HRMS (EI+) m/z: $M^{\bullet+}$ calculated for $C_{28}H_{36}O_3$ 420.2659; found 420.2669 (2 ppm).

Synthesis of 29. Synthesized following general procedures **A**, **B** and **C**. (**A/B**: 3 mmol 2,6-dimethoxybenzoic acid, 84% yield after recrystallization. **C**: 2,6-di(hept-6-en-1-yl)-4-methoxyphenol (**20**) (0.50 g, 1 equiv, 1.6 mmol), perfluorophenyl 2,6-dimethoxybenzoate (0.55 g, 1 equiv, 1.6 mmol), cesium carbonate (0.62 g, 1.2 equiv, 1.9 mmol). After purification by column chromatography using 2.5% EtOAc in heptane, 2,6-di(hept-6-en-1-yl)-4-methoxyphenyl

2,6-dimethoxybenzoate (**29**) (0.46 g, 0.96 mmol, 61 %) was obtained as a white solid. ^{1}H NMR ($CDCl_3$ 400MHz) δ 7.35 (t, 2H), 6.67 (s, 2H), 6.64 (d, 2H), 5.85-5.75 (m, 2H), 5.01-4.91 (m, 4H), 3.88 (s, 6H), 3.81 (s, 3H), 2.70 (brs, 4H), 2.08-2.02 (m, 4H), 1.66-1.62 (m, 4H), 1.45-1.39 (m, 8H) ppm. ^{13}C{^{1}H} NMR ($CDCl_3$ 101 MHz) δ 165.4, 157.6, 157.2, 141.0, 139.2, 139.1, 136.3, 131.3, 114.3, 114.3, 113.0, 112.6, 104.0, 55.9, 55.5, 33.9, 30.3, 29.9, 29.2, 28.9 ppm. HRMS (EI+) m/z: $M^{•+}$ calculated for $C_{30}H_{40}O_5$ 480.2875, found 480.2928 (error 12 ppm).

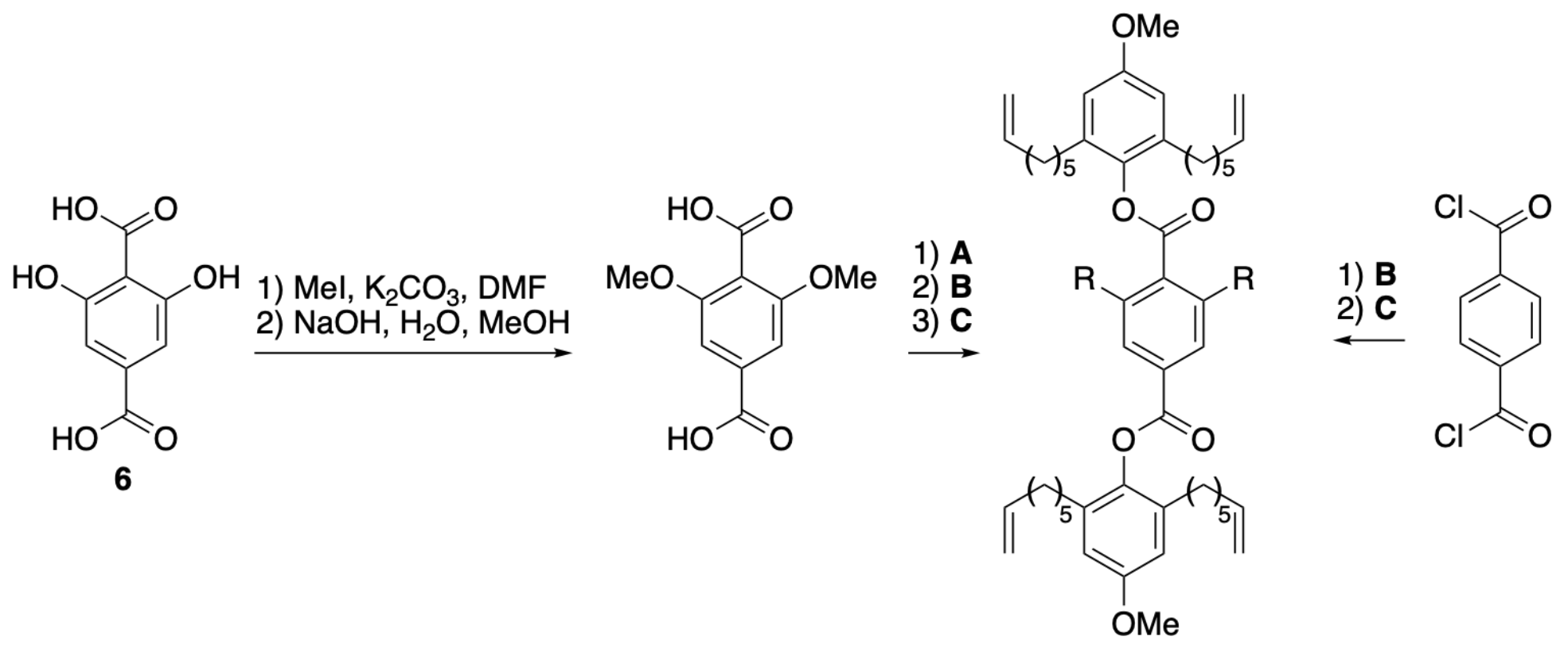

R = OMe: **31**
R = H: **32**

Synthesis of 31. 2,6-Dihydroxyterephthalic acid **6** (2.0 g, 10 mmol) and K_2CO_3 (6.9 g, 50 mmol, 5 equiv) were dissolved in dry DMF (25 mL) and methyl iodide (3.1 mL, 50 mmol, 5 equiv) was added to the mixture and stirred for 16 hours at 50° C. The reaction mixture was poured into water (200 mL) and extracted using DCM (5 x 50 mL). The combined organic layer was washed with water (3 x 50 mL), dried over anhydrous magnesium sulphate, filtered and evaporated. The obtained residue was dissolved in methanol (50 mL) and a solution of 4M sodium hydroxide in water (25 mL). The resulting mixture was stirred for 16 hours at 80° C. After allowing the reaction mixture to cool to room temperature, the solvents were evaporated and the obtained slurry was dissolved in water (50 mL) and acidified with hydrochloric acid (37% in water) to a pH of 1. Meanwhile, the product precipitated from the solution as a white solid. After filtration and drying 2,6-dimethoxyterephthalic acid (2.2 g, quantitative) was obtained as a white solid and used for the synthesis of **31** following general procedure **A**, **B** and **C**. With **A/B**: 5 mmol terephthalic acid, 74% yield after recrystallization. **C**: 2,6-di(hept-6-en-1-yl)-4-methoxyphenol (**20**) (2.3 g, 2.0 equiv, 7.2 mmol), bis(perfluorophenyl) 2,6-dimethoxyterephthalate (2.0 g, 1 equiv, 3.6 mmol), cesium carbonate (2.9 g, 2.5 equiv, 9.0 mmol). After chromatography over silica using 2.5% EtOAc in heptane, bis(2,6-di(hept-6-en-1-yl)-4-methoxyphenyl) 2,6-dimethoxyterephthalate **31** (1.7 g, 58 %) was obtained as a white solid. ^{1}H NMR ($CDCl_3$, 400MHz) δ 7.52 (s, 1H), 7.51 (s, 1H), 6.71 (s, 2H), 6.70 (s, 2H), 5.84-5.72 (m, 4H), 5.02-4.91 (m, 8H), 4.00 (d, 6H), 3.83 (d, 6H), 2.72 (br m, 4H), 2.52-2.47 (m, 4H), 2.10-2.01 (m, 8H), 1.69-1.60 (m, 8H), 1.46-1.35 (m, 16H) ppm. ^{13}C{^{1}H} NMR ($CDCl_3$ 101 MHz) δ 164.5, 164.5, 158.3, 157.6, 157.5, 157.4, 141.1, 140.8, 139.0, 138.9, 138.9, 136.3, 135.8, 132.2, 117.7, 114.4, 114.4, 112.9, 112.9, 112.7, 105.8, 105.7, 56.6, 56.2, 55.5, 55.5, 33.8, 33.7, 30.8, 30.7, 30.3, 29.9, 29.9, 29.2, 29.0, 29.0, 28.9, 28.8, 28.8 ppm. HRMS (FD+) m/z: $M^{•+}$ calculated for $C_{52}H_{70}O_8$ 822.5056; found 822.5066 (1 ppm).

Synthesis of 32. Compound **32** was synthesized from commercial terephthaloyl chloride following general procedure **B** and **C**. **B**: 5 mmol terephthaloyl chloride, 58%. **C**: 2,6-di(hept-6-en-1-yl)-4-methoxyphenol (**20**) (1.3 g, 2.0 equiv, 4.0 mmol), bis(perfluorophenyl) terephthalate (1.0 g, 1 equiv, 2.0 mmol), cesium carbonate (1.6 g, 2.5 equiv, 5.0 mmol). After purification by column chromatography over silica using 2.5% EtOAc in heptane as the eluent, bis(2,6-di(hept-6-en-1-yl)-4-methoxyphenyl) terephthalate (**32**) (1.2 g, 78 %) was obtained as a white solid. ^{1}H NMR ($CDCl_3$ 400MHz) δ 8.43 (s, 4H), 6.74 (s, 4H), 5.83-5.73 (m, 4H), 5.00-4.92 (m, 8H), 3.84 (s, 6H), 2.54 (t, 8H, J = 8.0 Hz), 2.06 (q, 8H, J = 8.0 Hz), 1.71-1.64 (m, 8H), 1.44-1.35 (m, 16H) ppm. ^{13}C{^{1}H} NMR ($CDCl_3$, 101 MHz) δ 164.4, 157.5, 141.0, 138.8, 135.7, 133.8, 130.4, 114.3, 112.9, 55.4, 33.6, 30.7, 29.8, 28.9, 28.7 ppm. HRMS (FD+) m/z: $M^{•+}$ calculated for $C_{50}H_{66}O_6$ 762.4854; found 762.4857 (0.4 ppm).

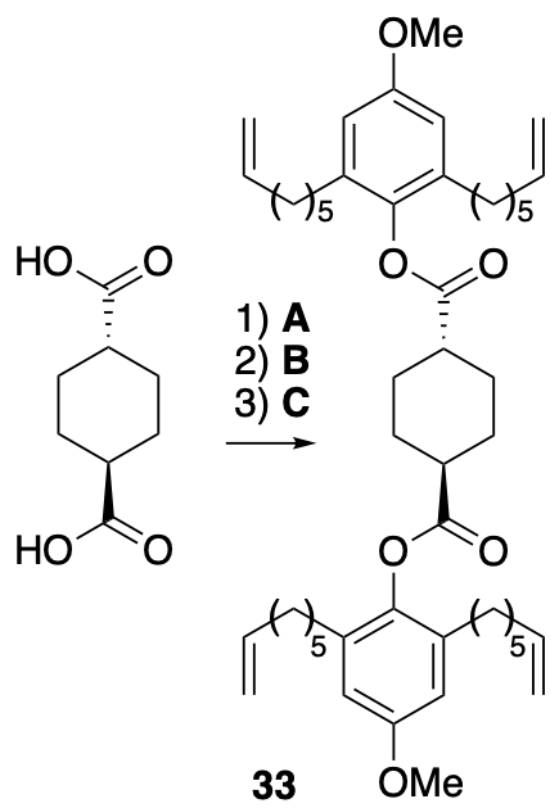

Synthesis of bis(2,6-di(hept-6-en-1-yl)-4-methoxyphenyl) cyclohexane-1,4-dicarboxylate (33). Compound **33** was synthesized from commercial trans cyclohexane-1,4-dicarboxylic acid following general procedures **A**, **B** and **C**. **A** and **B**: 10 mmol cyclohexane-1,4-dicarboxylic acid, 93% after recrystallizing from heptane. **C**: After purification by column chromatography over silica using 2.5% EtOAc in heptane as the eluent, **33** was obtained in 67% yield as a white solid. ^{1}H NMR ($CDCl_3$ 400MHz) δ 6.66 (d, 4H), 5.90-4.79 (m, 4H), 5.07-4.96 (m, 8H), 3.80 (s, 6H), 2.72-2.67 (brs, 2H), 2.58 (t, 2H, J = 8.0 Hz), 2.44 (t, 6H, J = 8.0 Hz), 2.37 (d, 4H, J = 8.0 Hz), 2.12-2.06 (m, 8H), 1.81-1.76 (m, 4H), 1.66-1.57 (m, 8H), 1.491.37 (m, 16H) ppm. ^{13}C{^{1}H} NMR ($CDCl_3$, 101 MHz) δ 173.9, 157.2, 153.2, 145.4, 140.8, 139.0, 139.0, 138.9, 135.6, 129.4, 114.4, 114.3, 114.3, 112.8, 55.6, 55.4, 42.7, 33.8, 33.7, 30.7, 30.5, 29.8, 29.7, 29.1, 28.8, 28.8, 28.8, 28.2 ppm. HRMS (FD+) m/z: $M^{\bullet+}$ calculated for $C_{50}H_{72}O_6$ 768.5323; found 768.5348 (3 ppm).

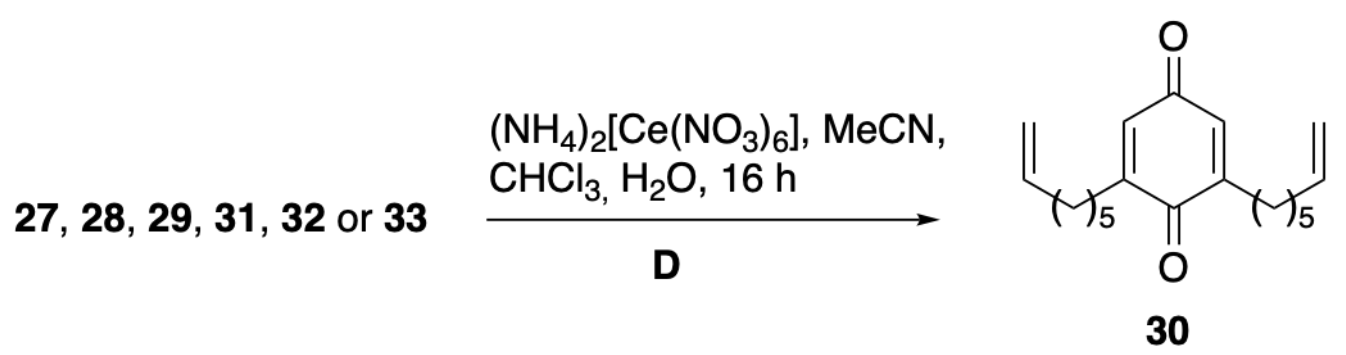

*General procedure **D** for the oxidative cleavage of the methoxyphenolic esters providing 2,6-di(hept-6-en-1-yl)cyclohexa-2,5-diene-1,4-dione* (**30**). The methoxyphenolic ester (1 mmol, 1 equiv) was dissolved in chloroform (10 mL). A solution of ceric ammonium nitrate (1.5 equiv per methoxyphenolic ester moiety) in water (5 mL) was added under vigorous stirring. After complete consumption of the starting material, the two layers were separated. The water layer was washed with chloroform (3 times) and the combined organic layers were washed with water (2 times) and brine (once). After drying over anhydrous magnesium sulphate and filtration over a small layer of silica to remove salts, quinone **30** was obtained as a yellow liquid. Compounds **27**, **28**, **29**, **31**, **32** and **33** were subjected to oxidation following general procedure **D**. From **27**: 90% yield. From **28**: 88% yield. From **29**: 76% yield. From **31**: No reaction. From **32**: No reaction. From **33**: 62% yield. ^{1}H NMR ($CDCl_3$ 400MHz) δ 6.49 (s, 2H), 5.84-5.74 (m, 2H), 5.02-4.92 (m, 4H), 1.59-1.25 (m, 16H) ppm. ^{13}C{^{1}H} NMR ($CDCl_3$, 101 MHz) δ 188.3, 187.8, 149.9, 138.9, 132.3, 114.6, 33.7, 29.3, 28.9, 28.7, 27.8 ppm. HRMS (FD+) m/z: $[M]^+$ calculated for $C_{20}H_{28}O_2$ 300.2084; found 300.2063 (7 ppm).

Copies of the ^{1}H-NMR, ^{13}C-NMR and high-resolution mass spectra.

13-bromotridec-1-ene. ^{1}H-NMR (400 MHz, $CDCl_3$)

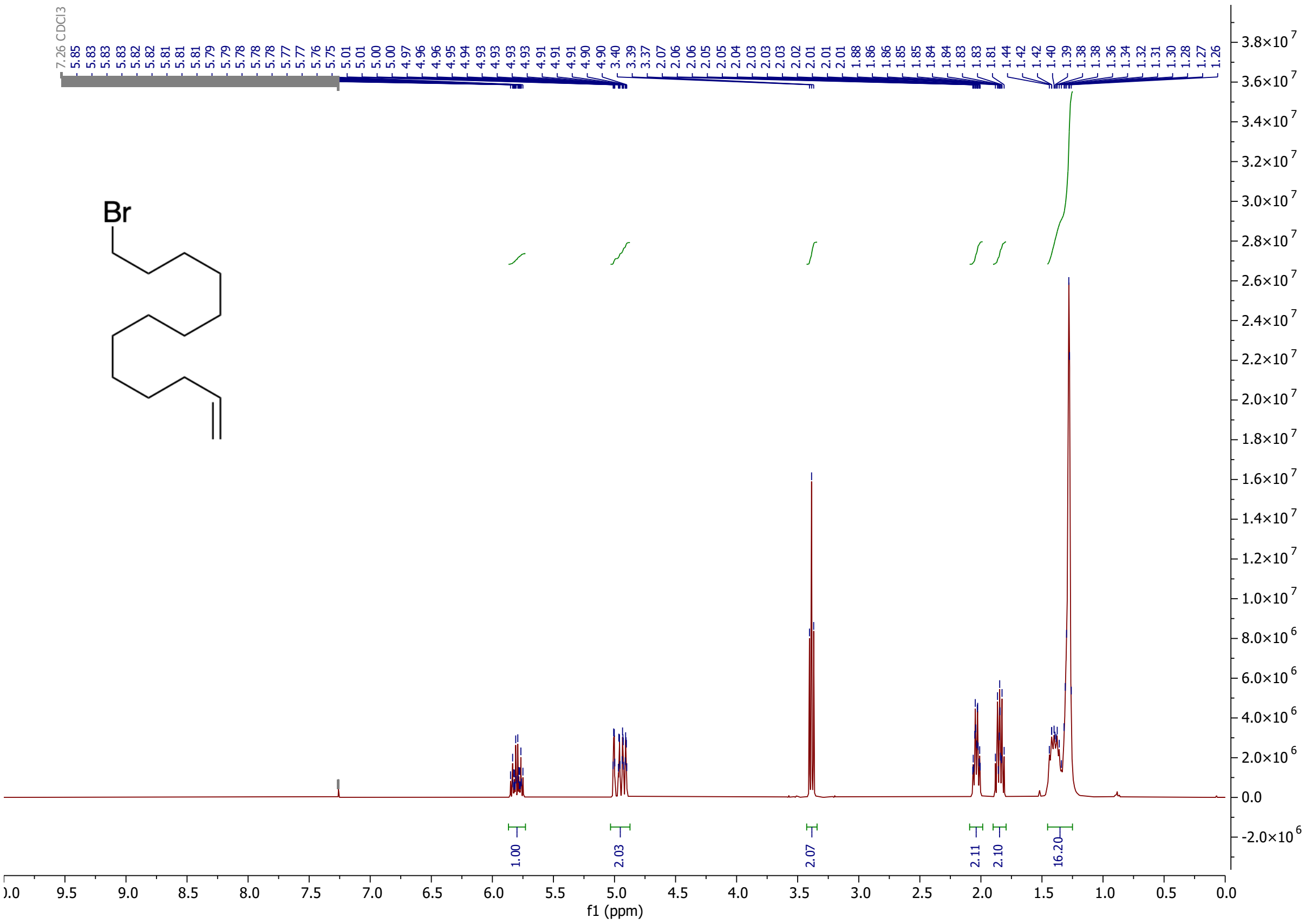

^{13}C{^{1}H}-NMR (101 MHz, $CDCl_3$)

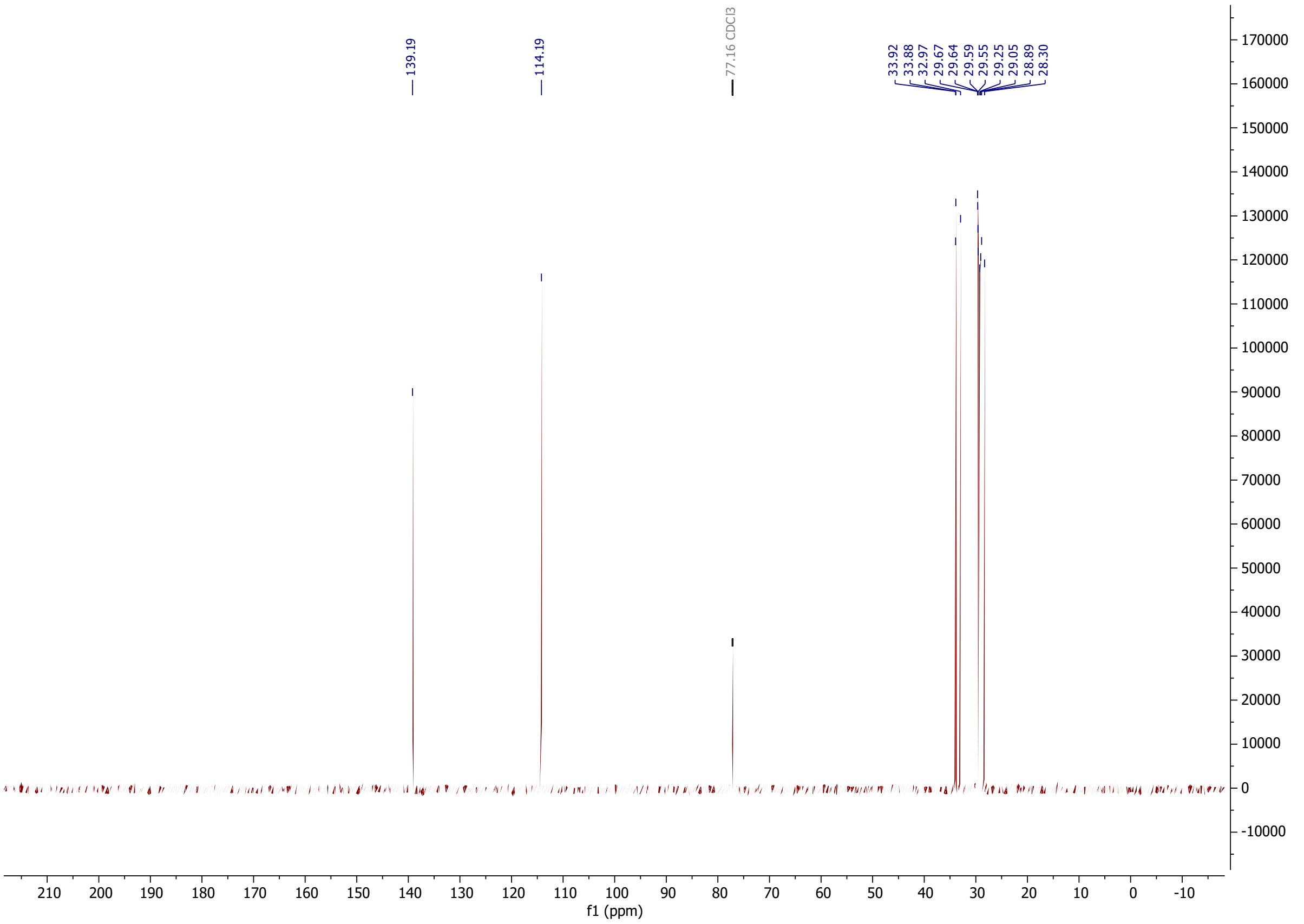

Precatenane precursor (2). ^{1}H-NMR (400 MHz, $CDCl_3$)

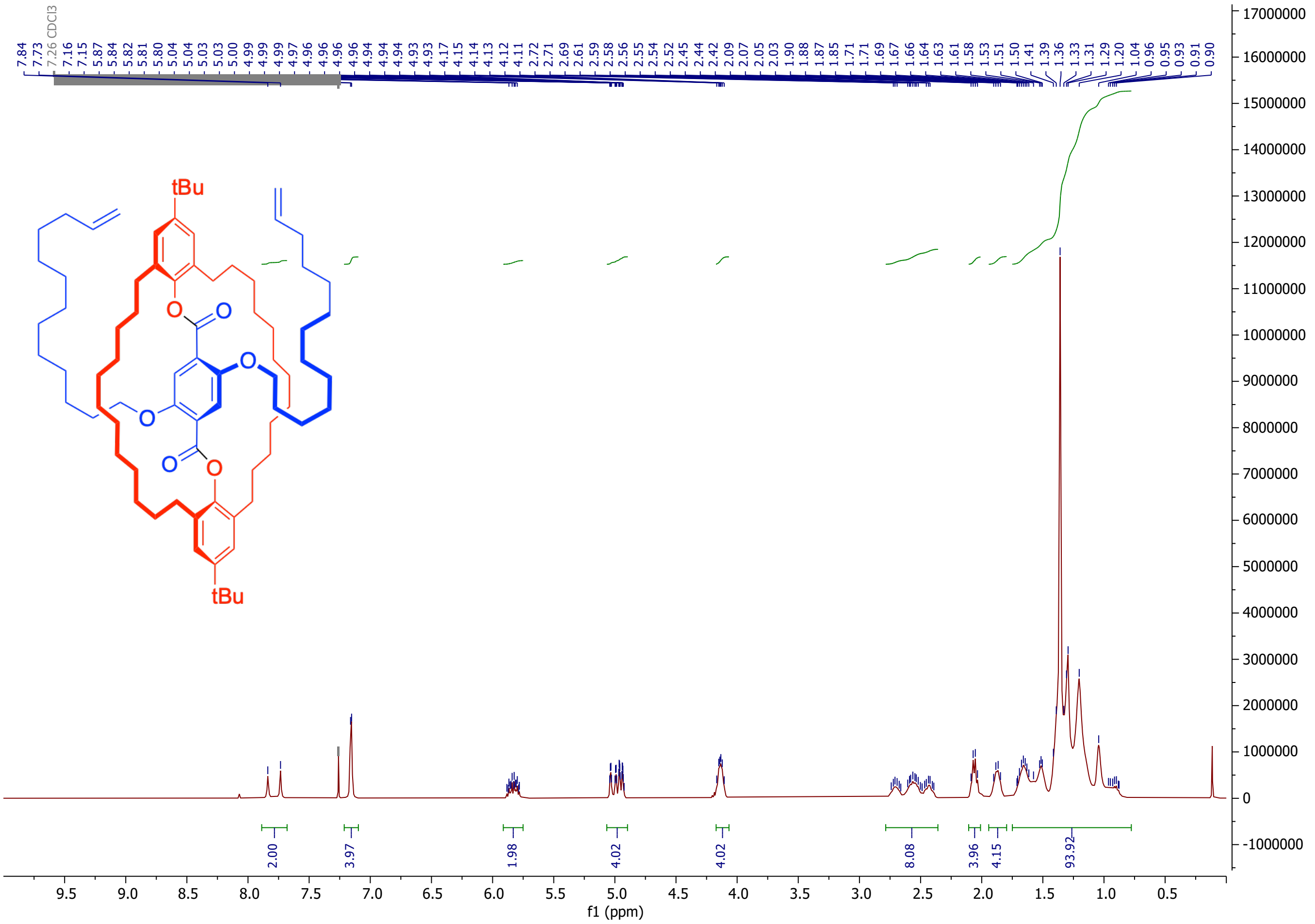

^{13}C{^{1}H}-NMR (101 MHz, $CDCl_3$)

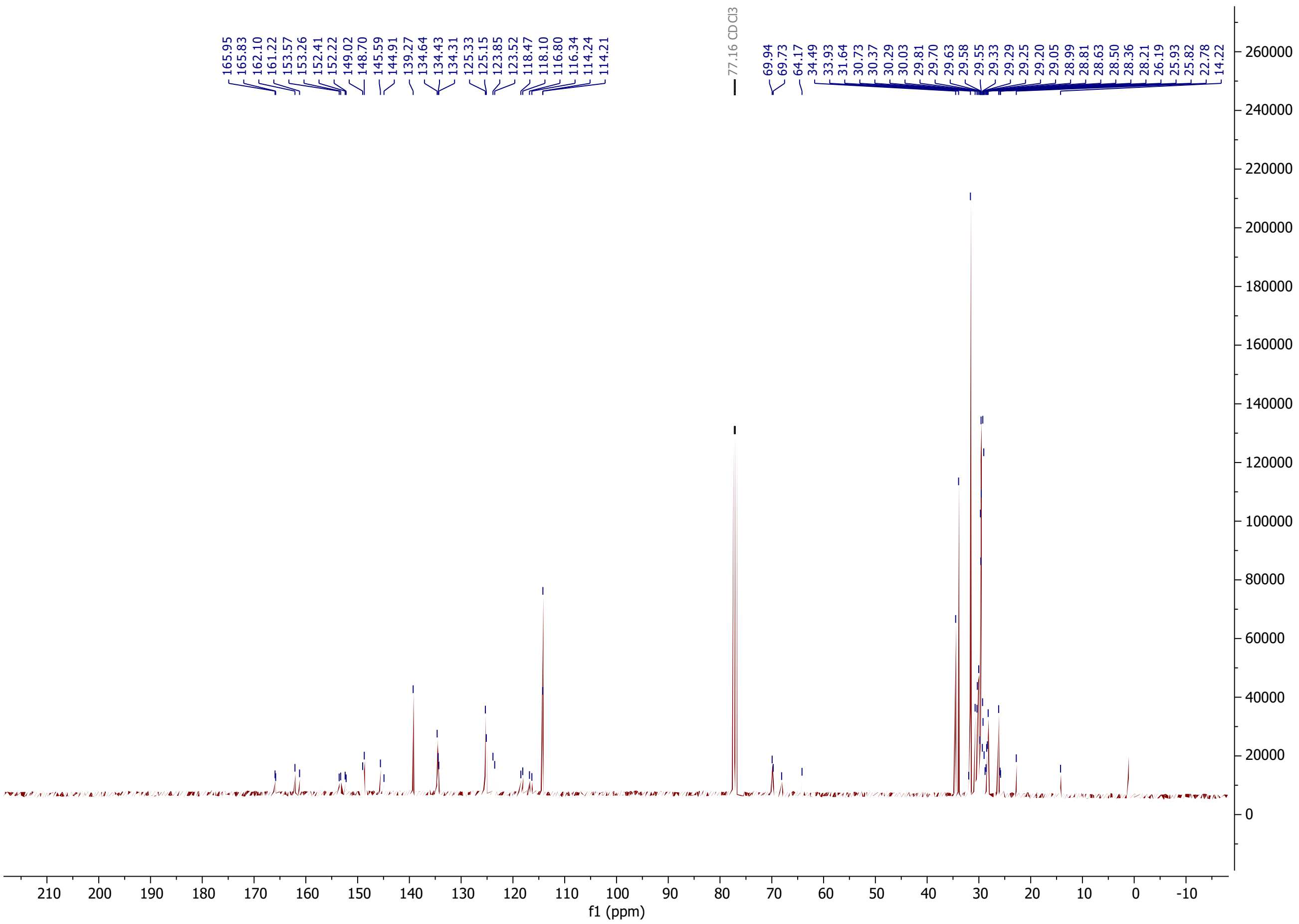

HRMS

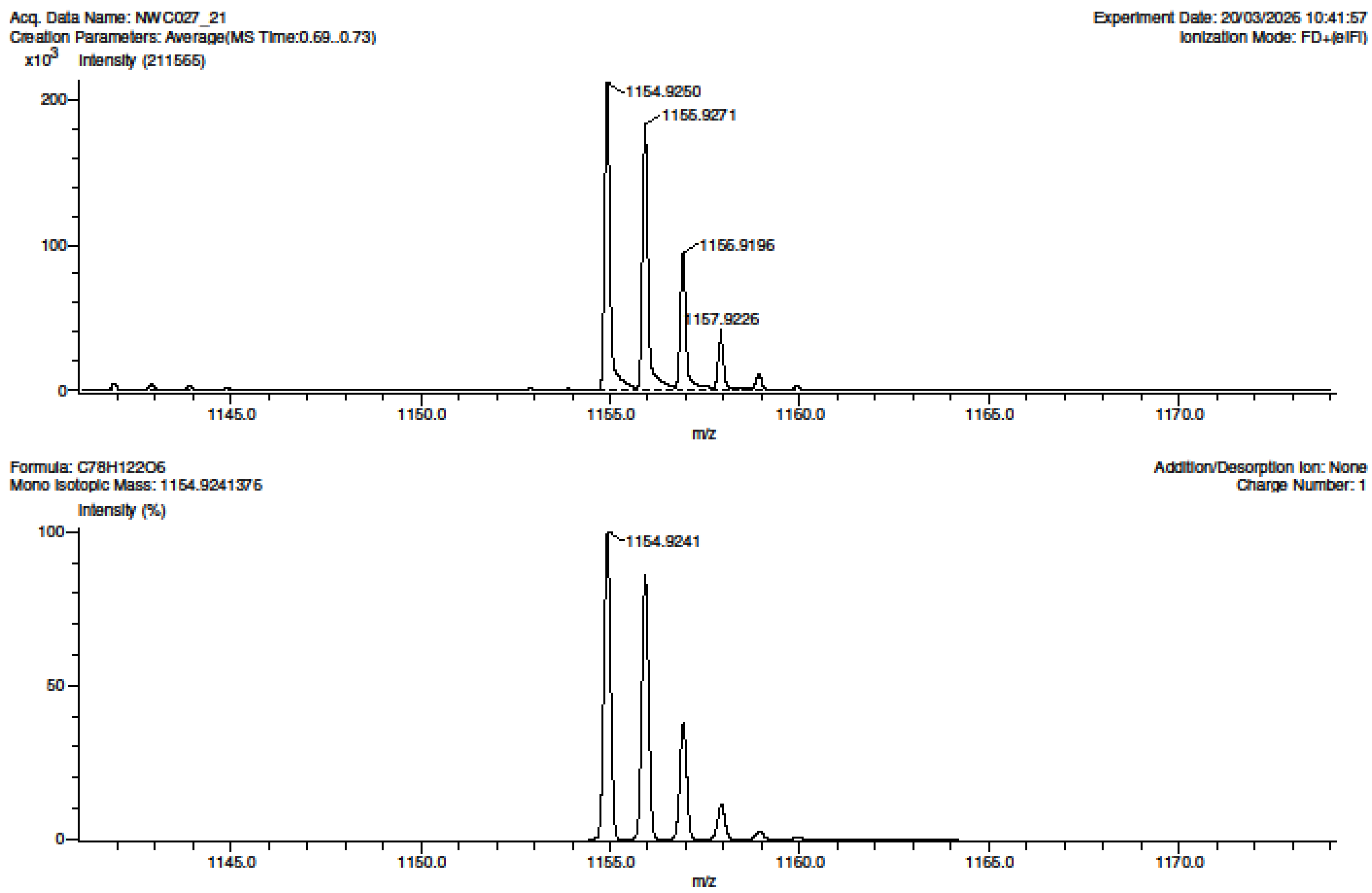

Precatenane 3a. ^{1}H-NMR (400 MHz, CDCl$_3$)

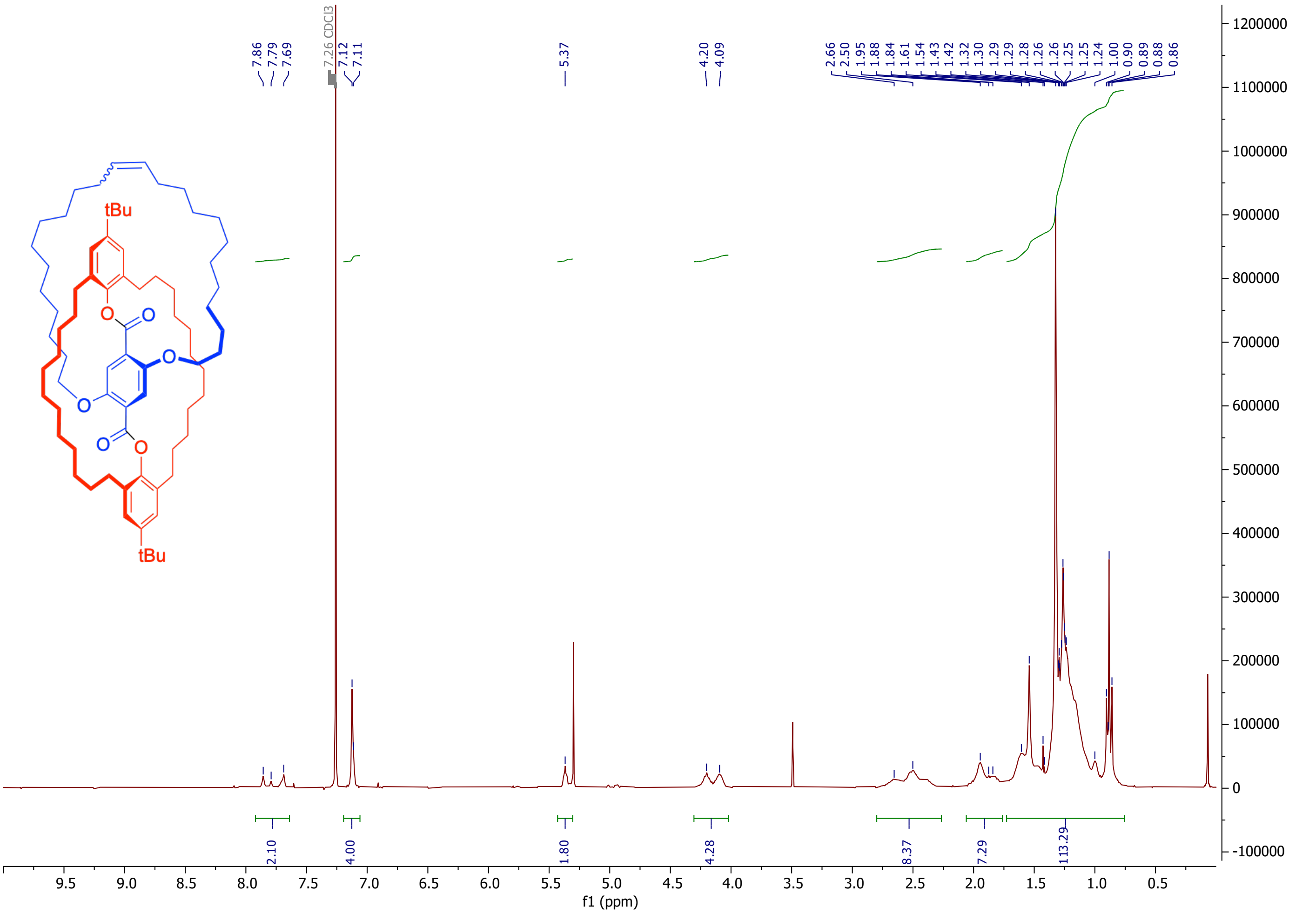

Precatenane 3b. ^{1}**H-NMR (400 MHz, CDCl$_3$)**

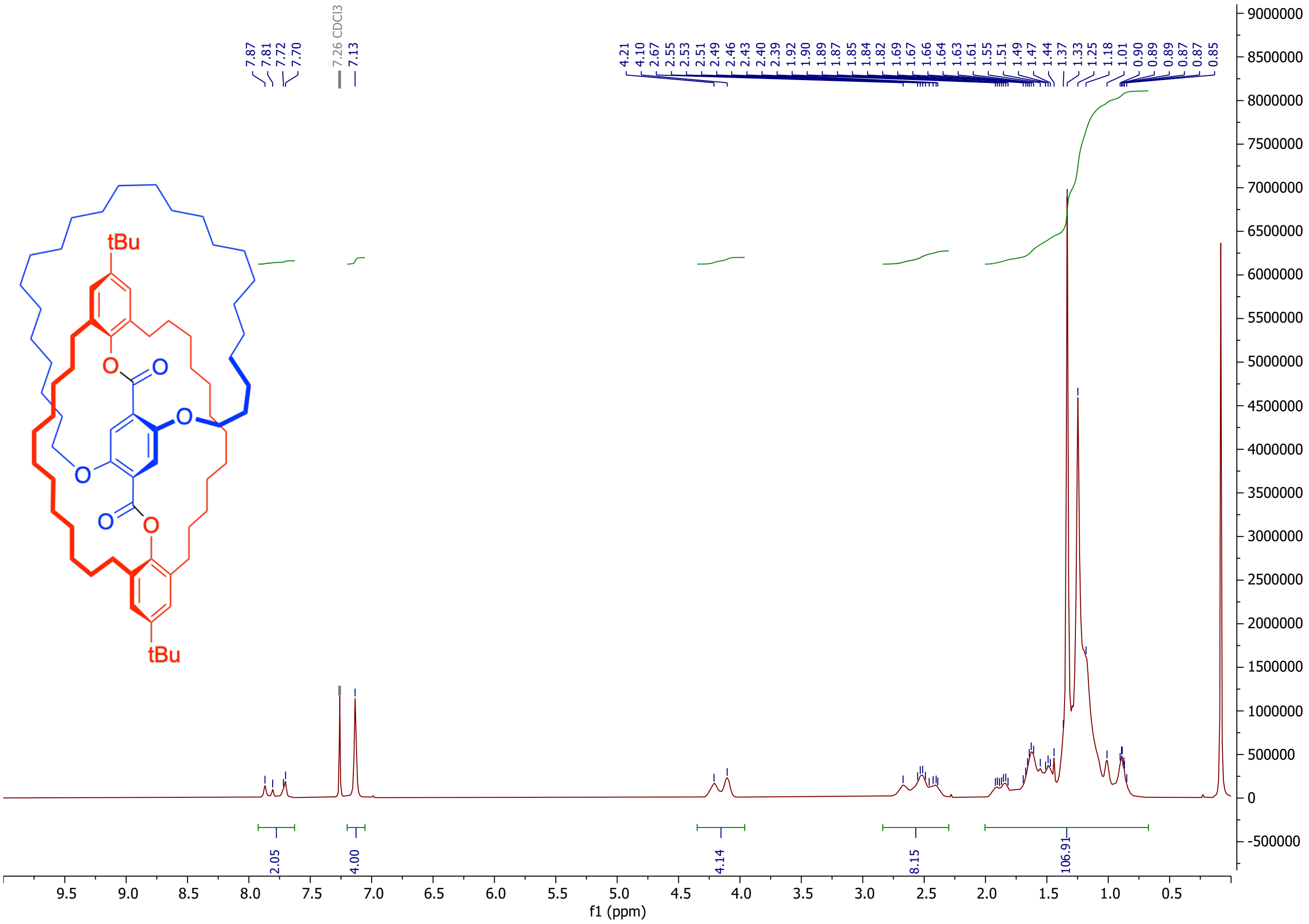

HRMS

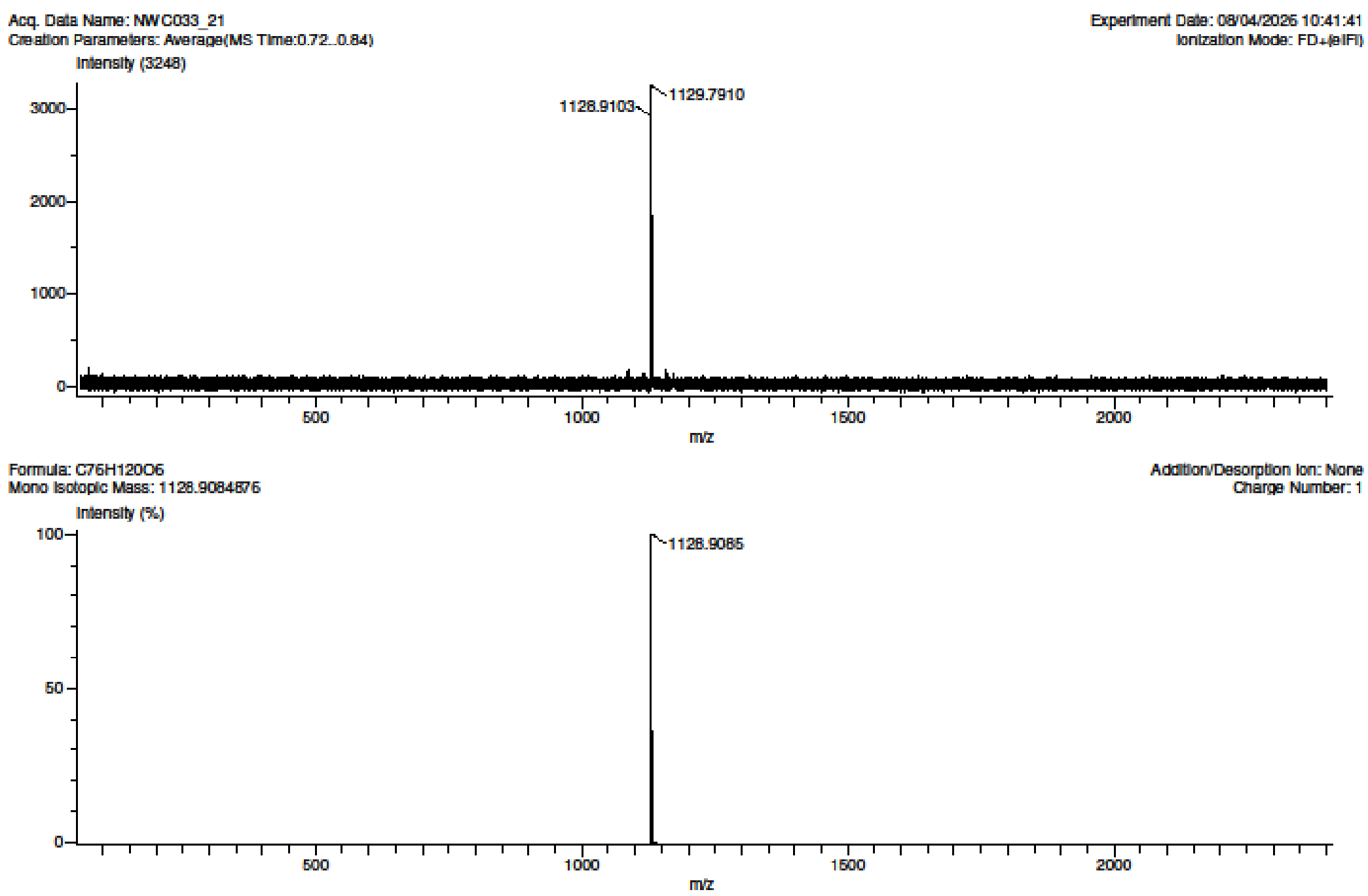

Catenane 4. ^{1}H-NMR (400 MHz, $CDCl_3$)

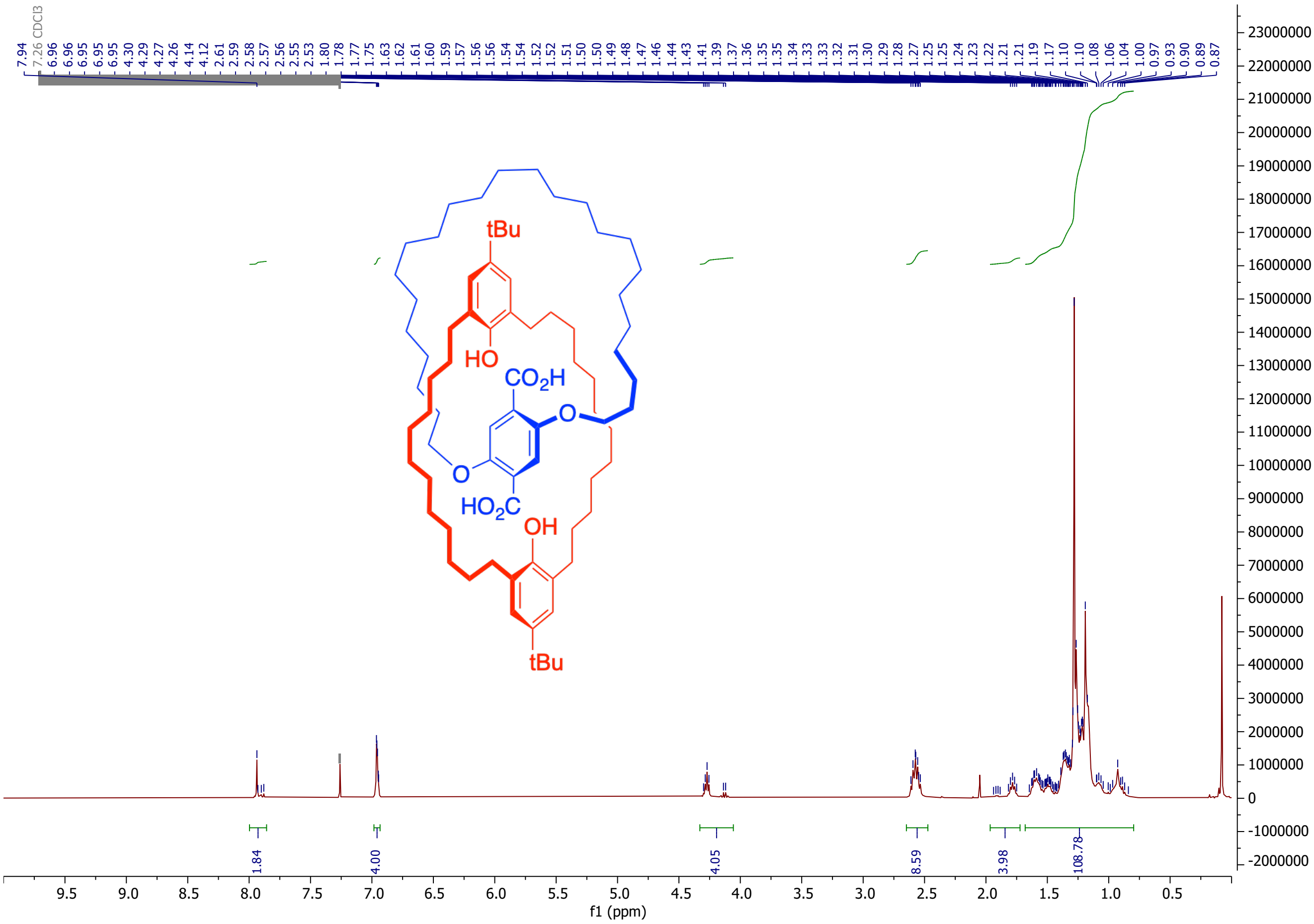

^{13}C{^{1}H}-NMR (101 MHz, $CDCl_3$)

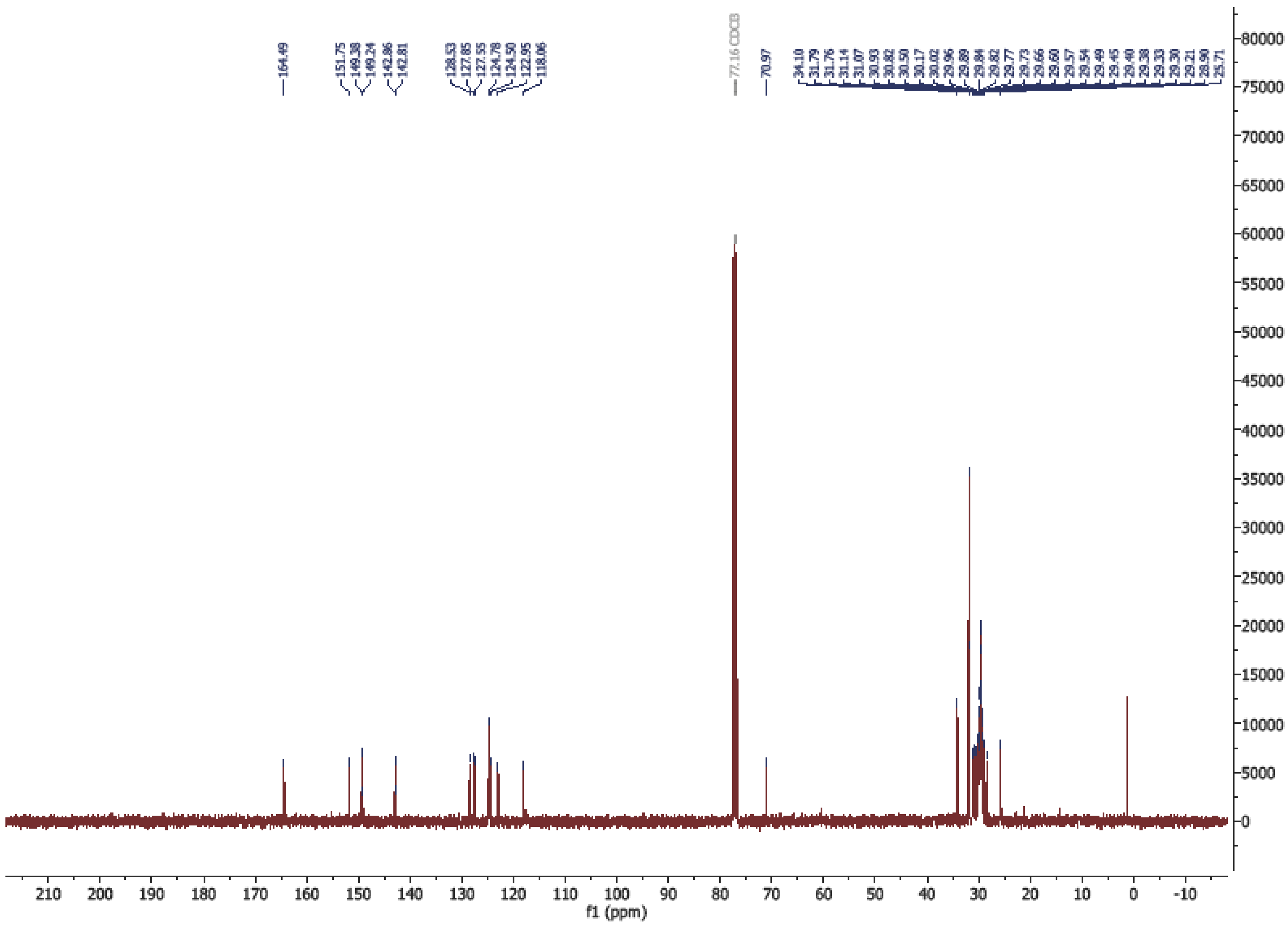

HRMS

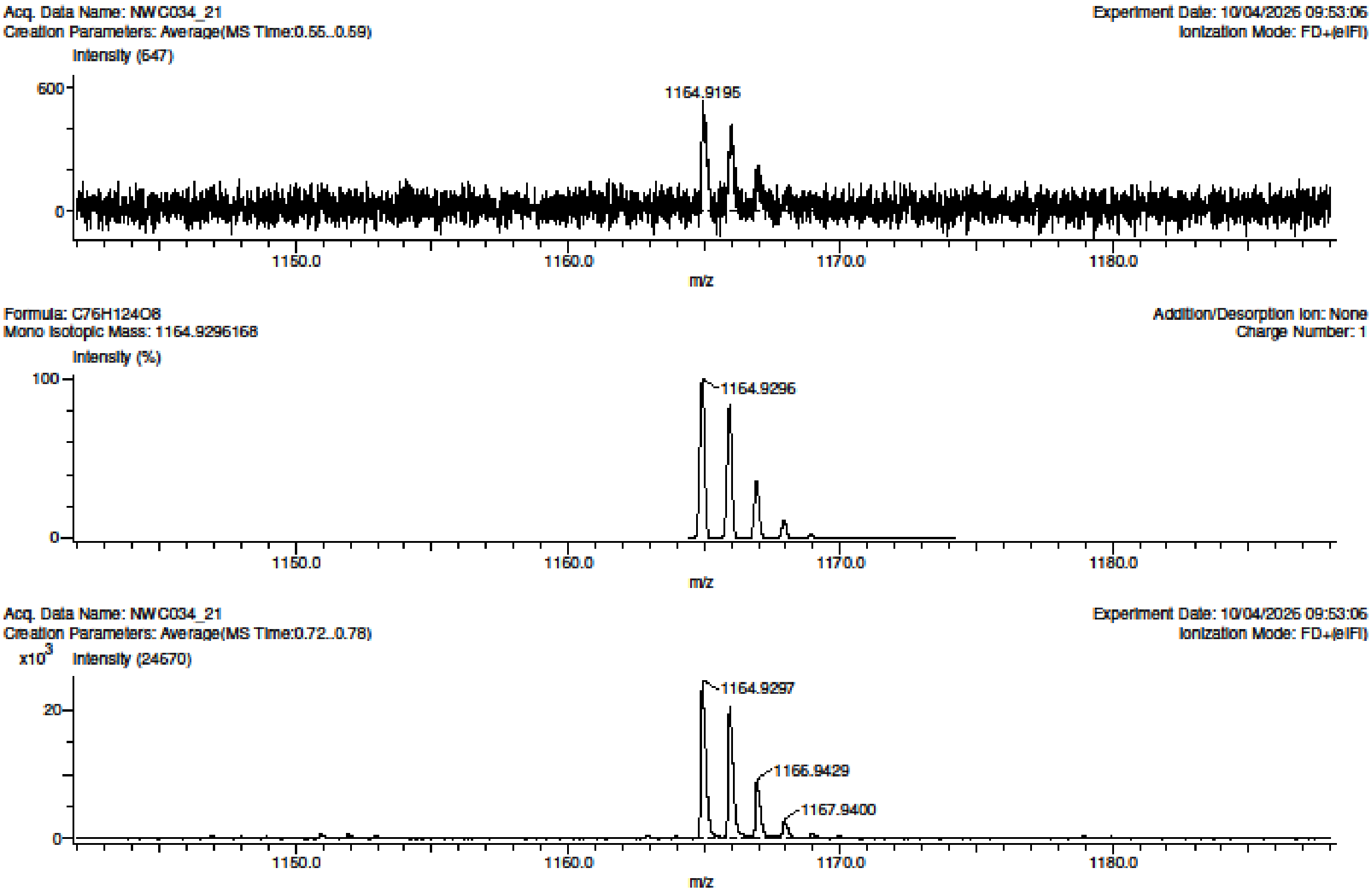

2,6-Dihydroxyterephthalic acid (6). ^{1}H-NMR (400 MHz, DMSO-d_6)

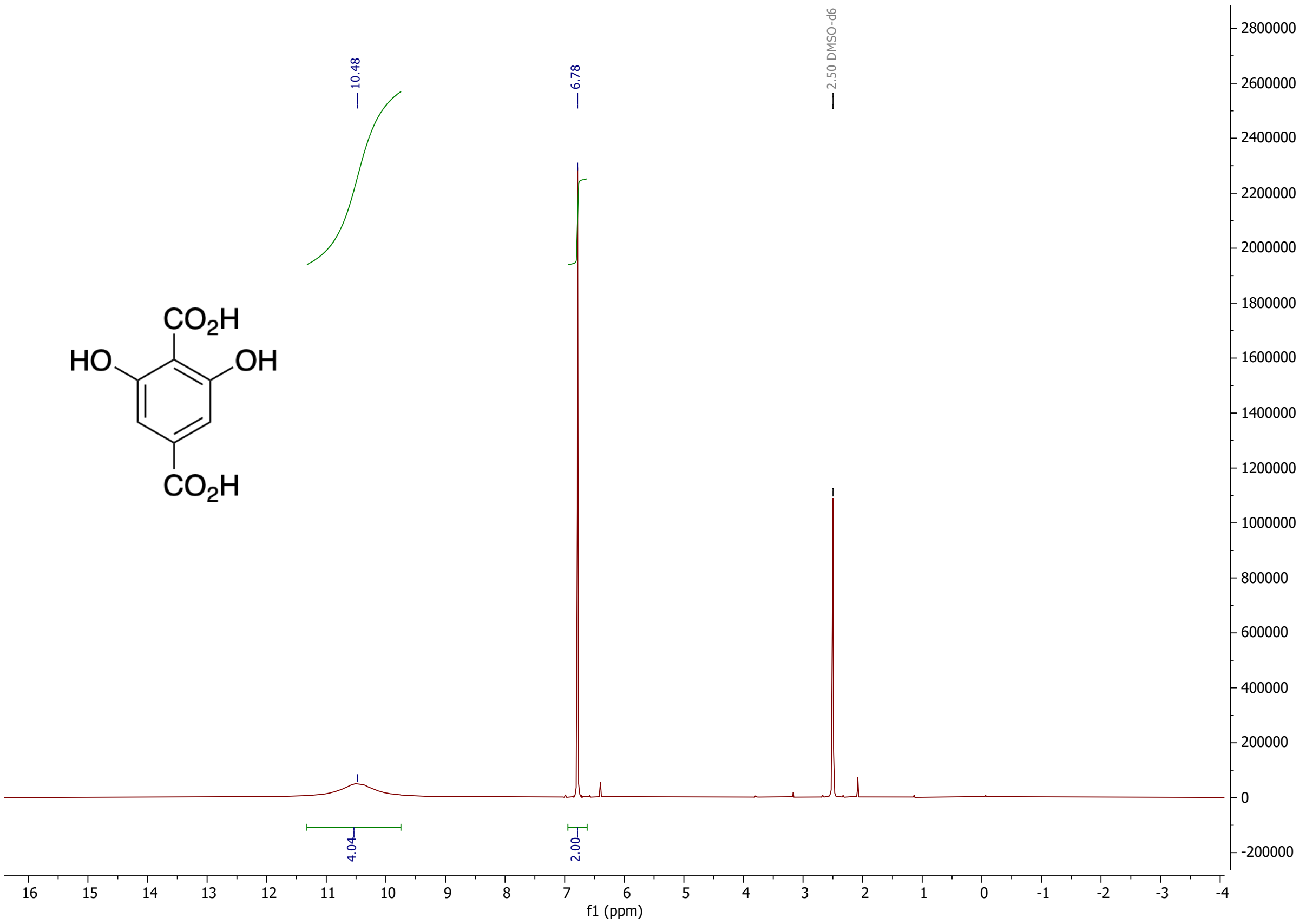

$^{13}C\{^1H\}$-NMR (101 MHz, DMSO-d$_6$)

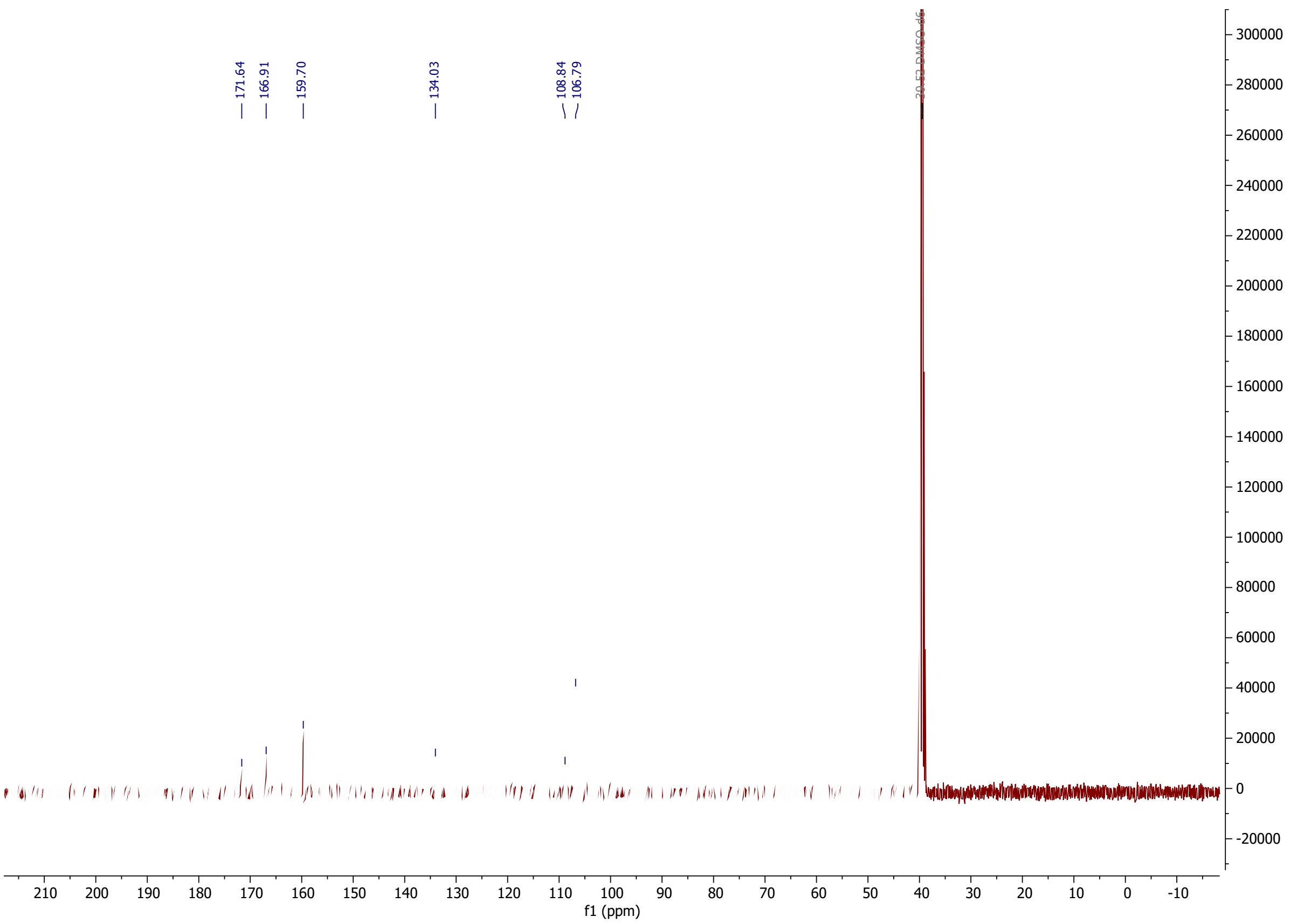

Diallyl 2,6-bis(allyloxy)terephthalate (7). ^1H-NMR (400 MHz, $CDCl_3$)

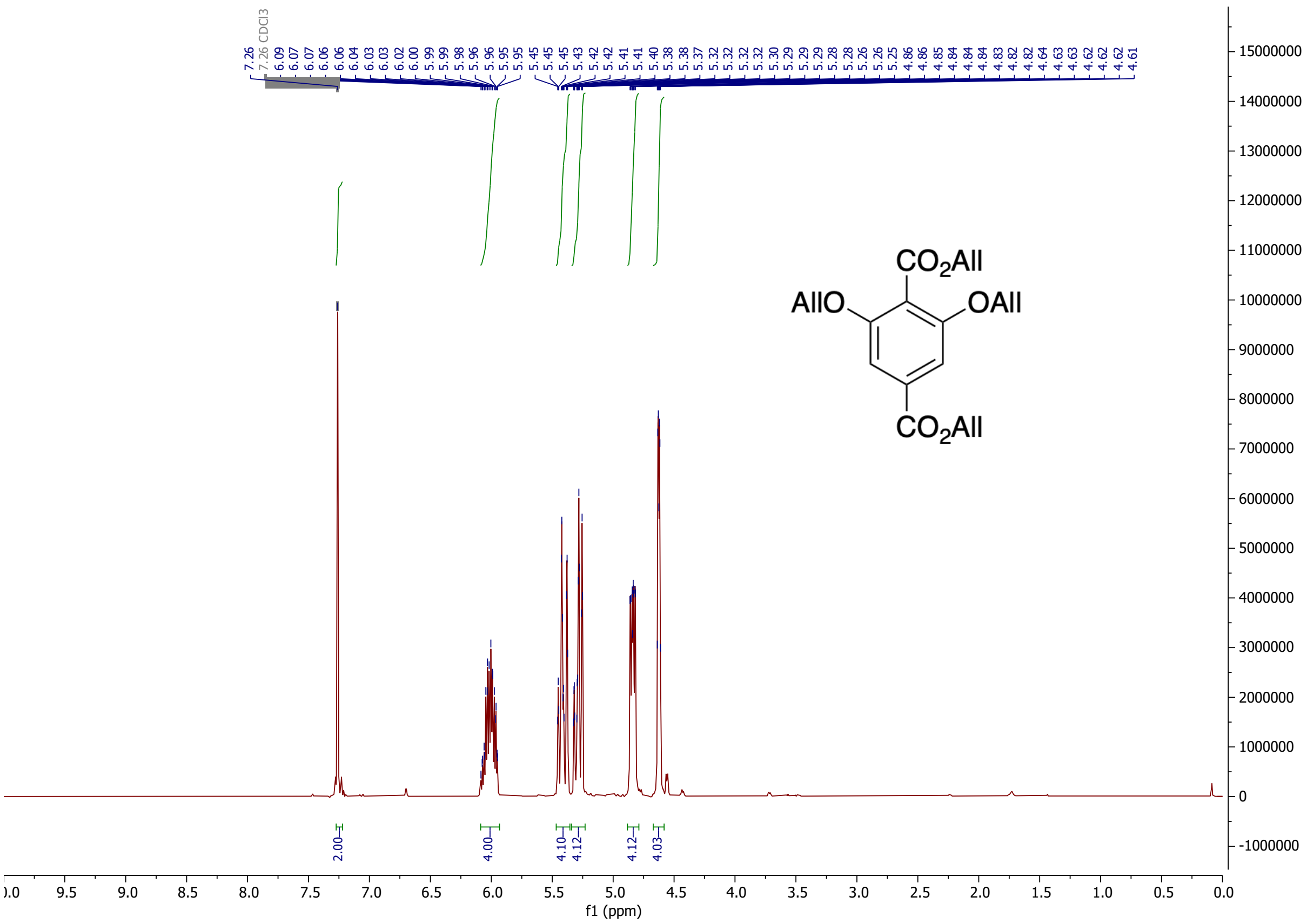

$^{13}C\{^1H\}$-NMR (101 MHz, $CDCl_3$)

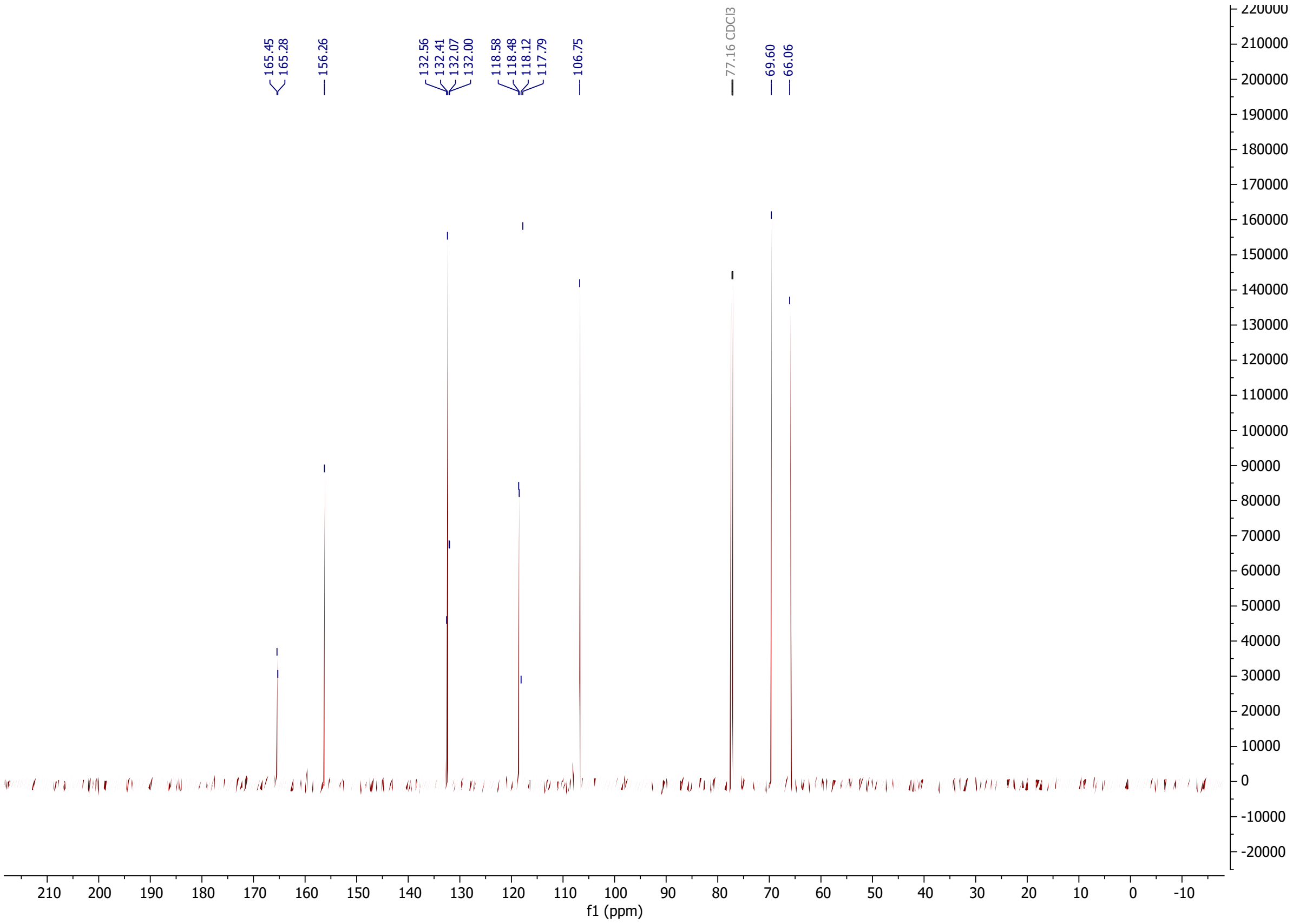

HRMS

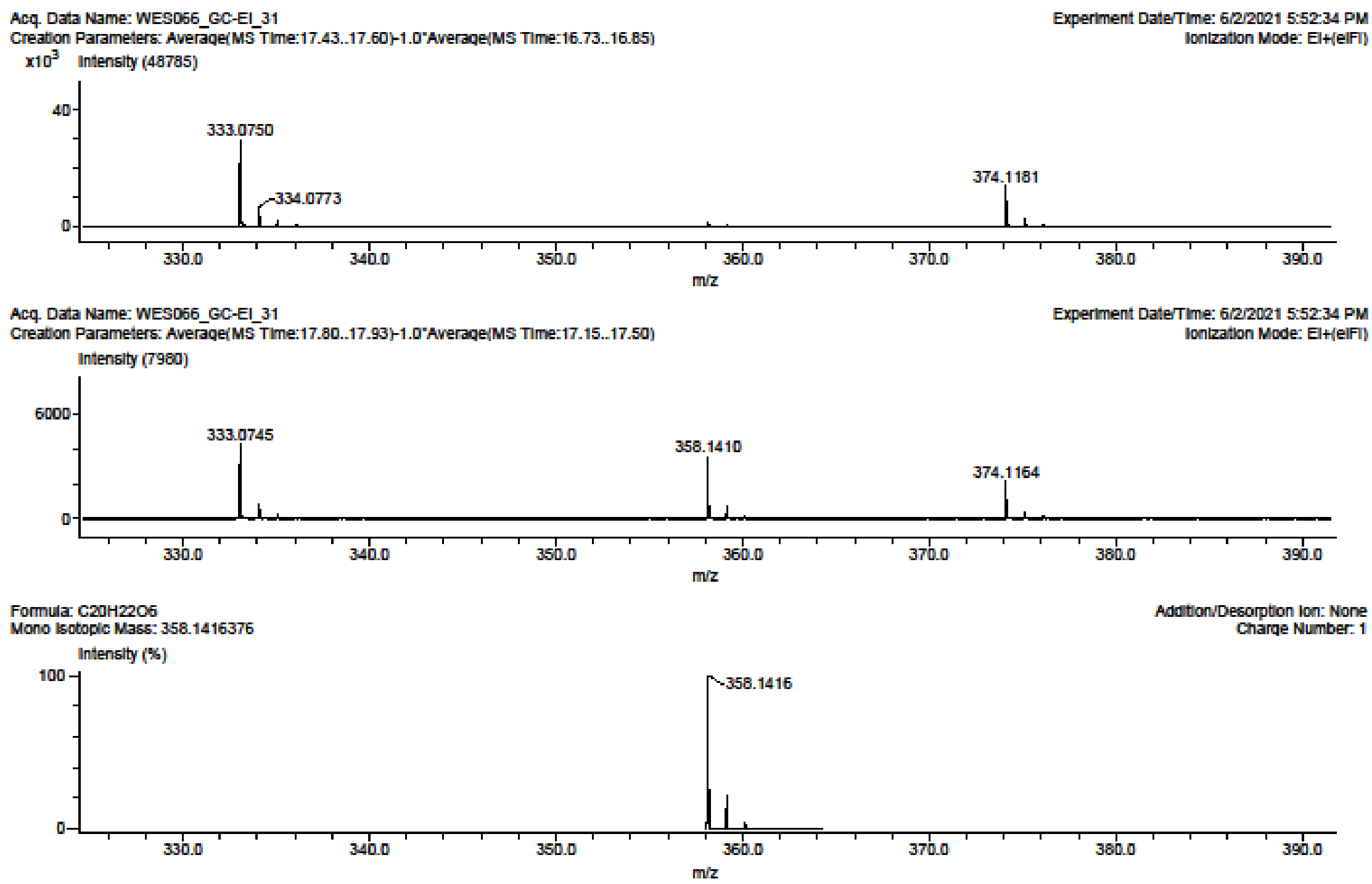

2,6-Bis(allyloxy)terephthalic acid (8). ^{1}H-NMR (400 MHz, DMSO-d_6)

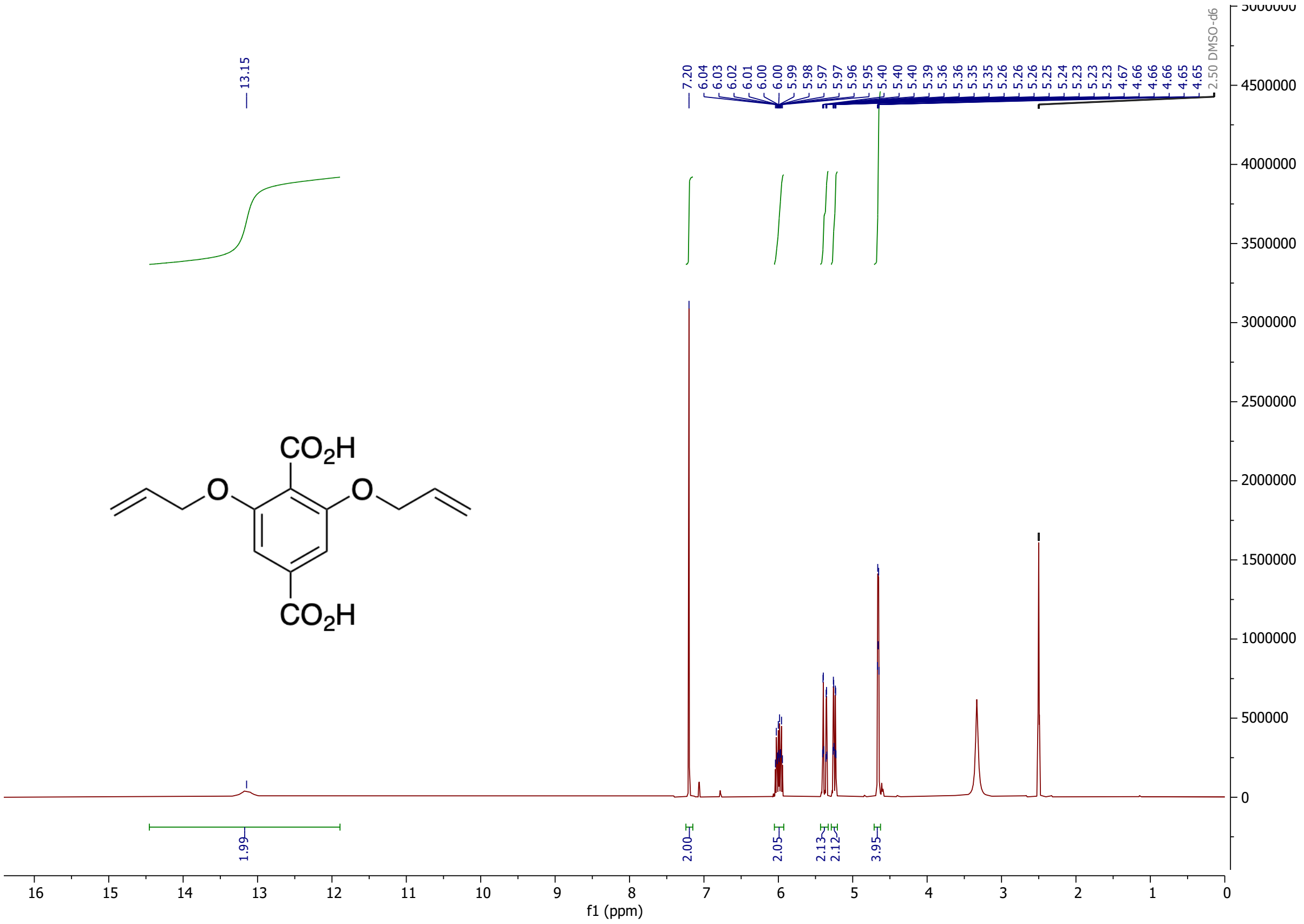

$^{13}C\{^{1}H\}$-NMR (101 MHz, DMSO-d_6)

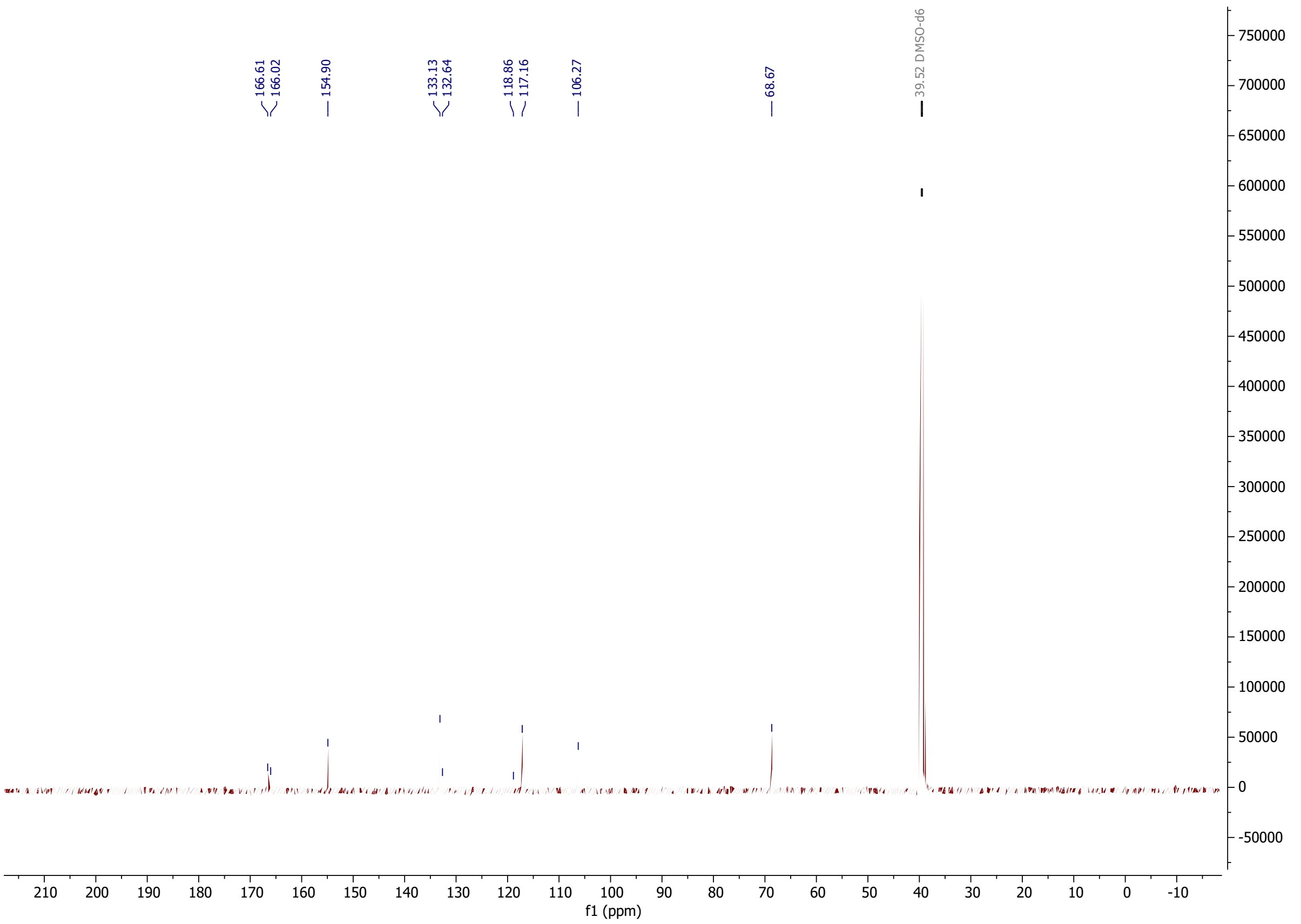

HRMS

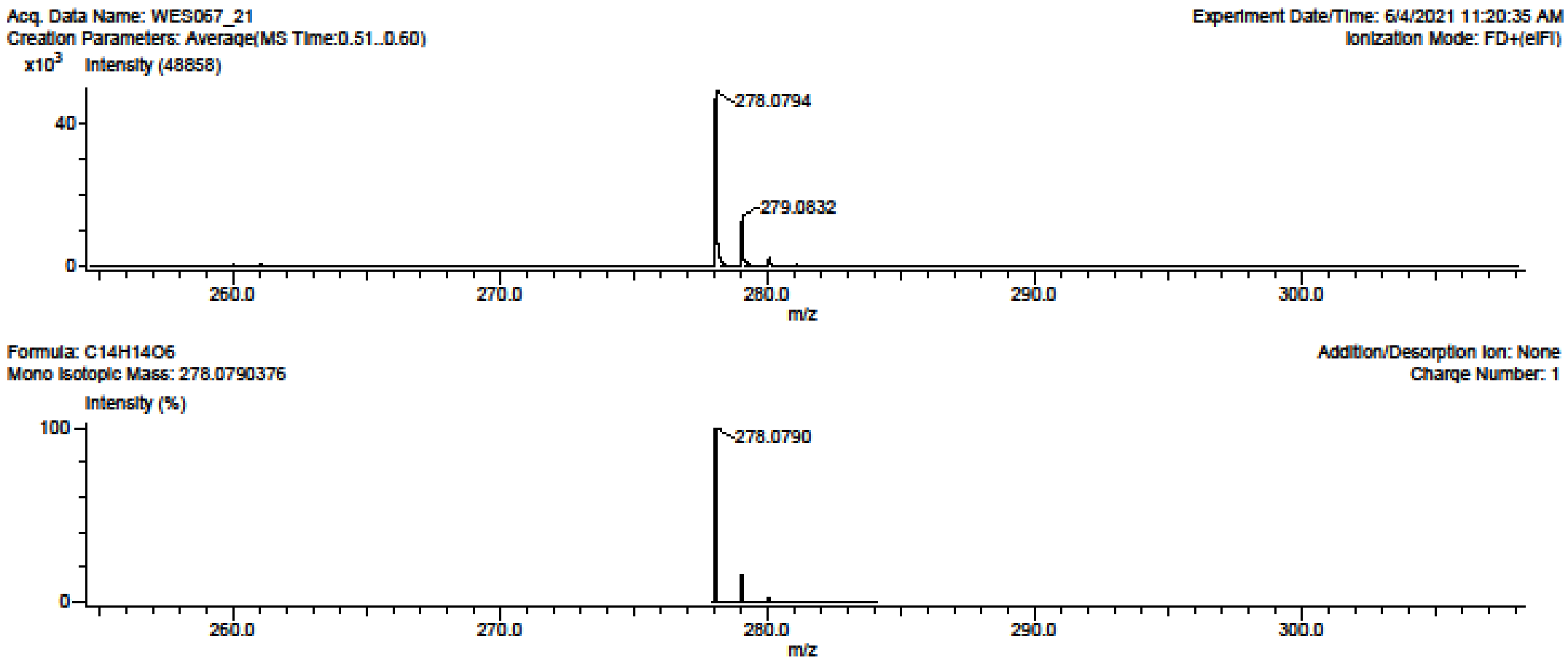

Bis(perfluorophenyl) 2,6-bis(allyloxy)terephthalate (9). ^{1}H-NMR (400 MHz, $CDCl_3$)

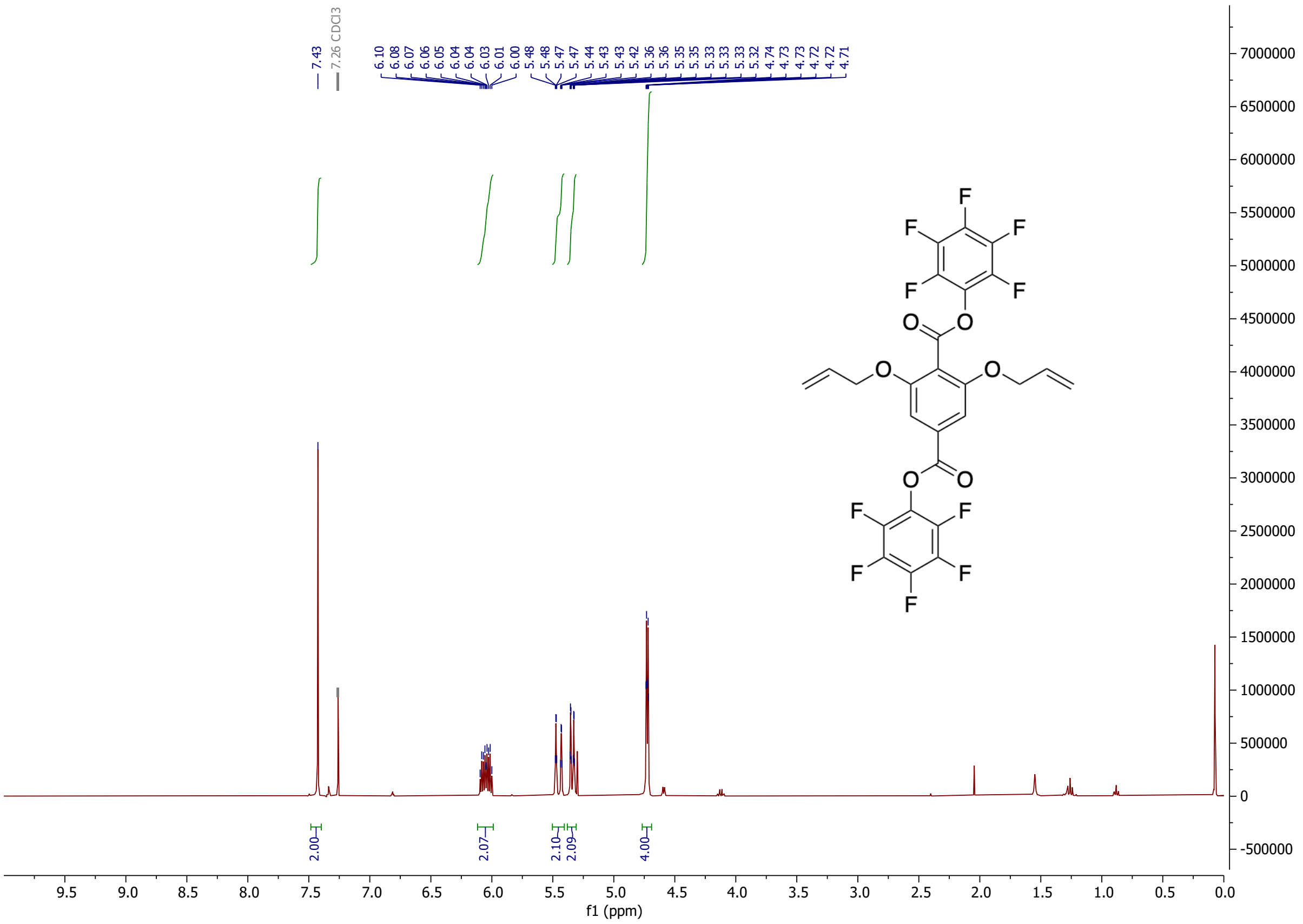

$^{13}C\{^1H\}$-NMR (101 MHz, $CDCl_3$)

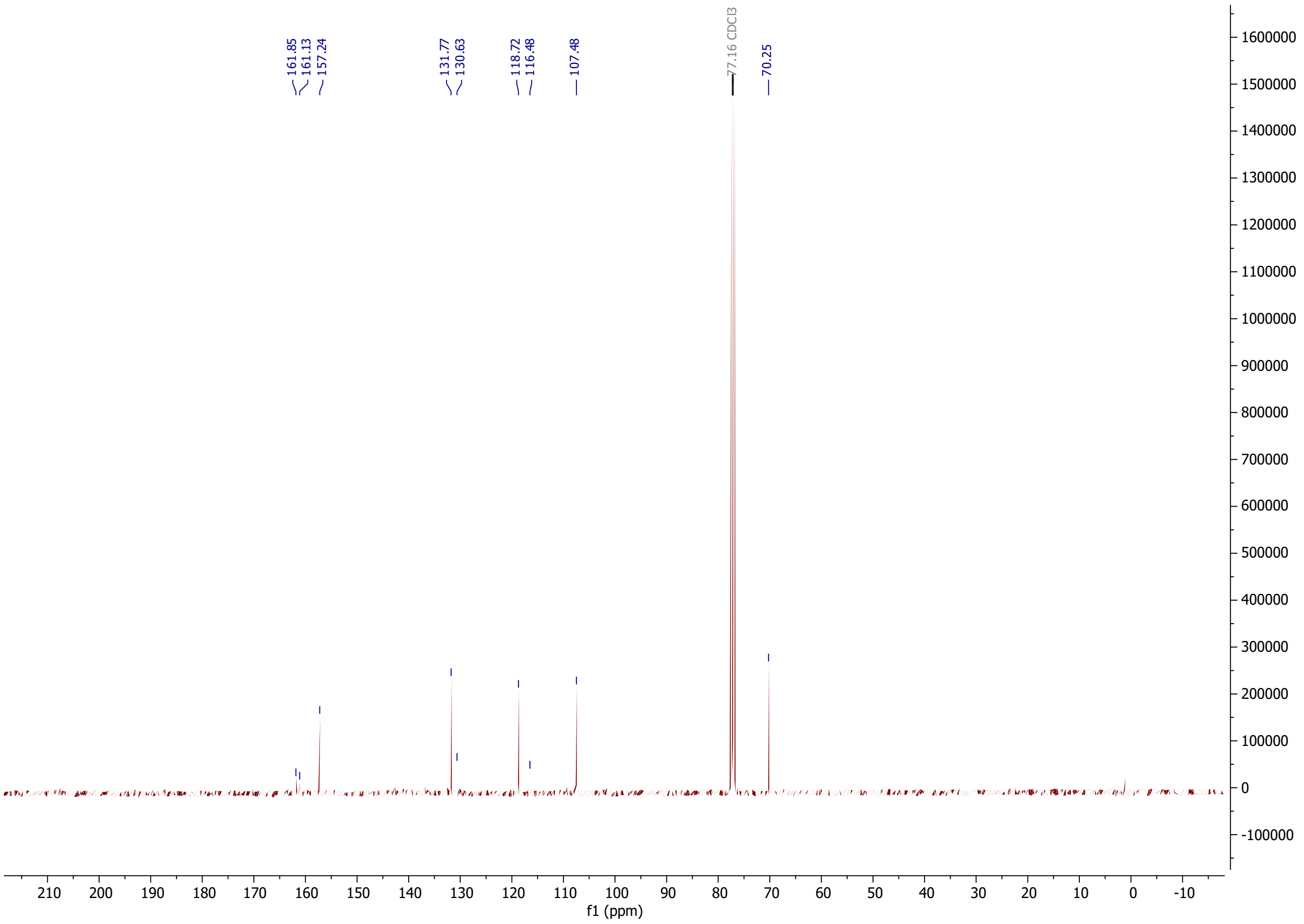

^{19}F-NMR (376 MHz, $CDCl_3$)

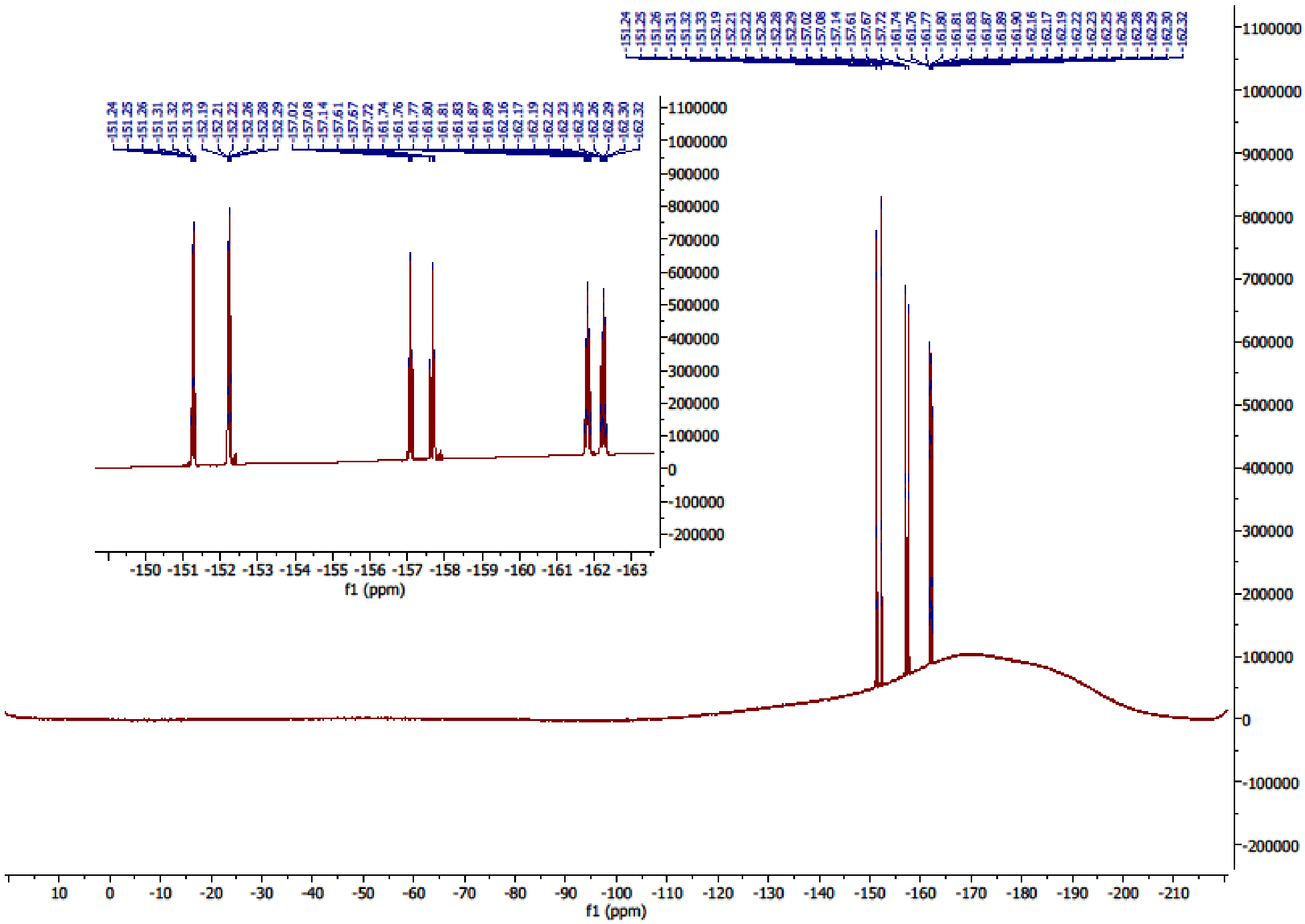

HRMS

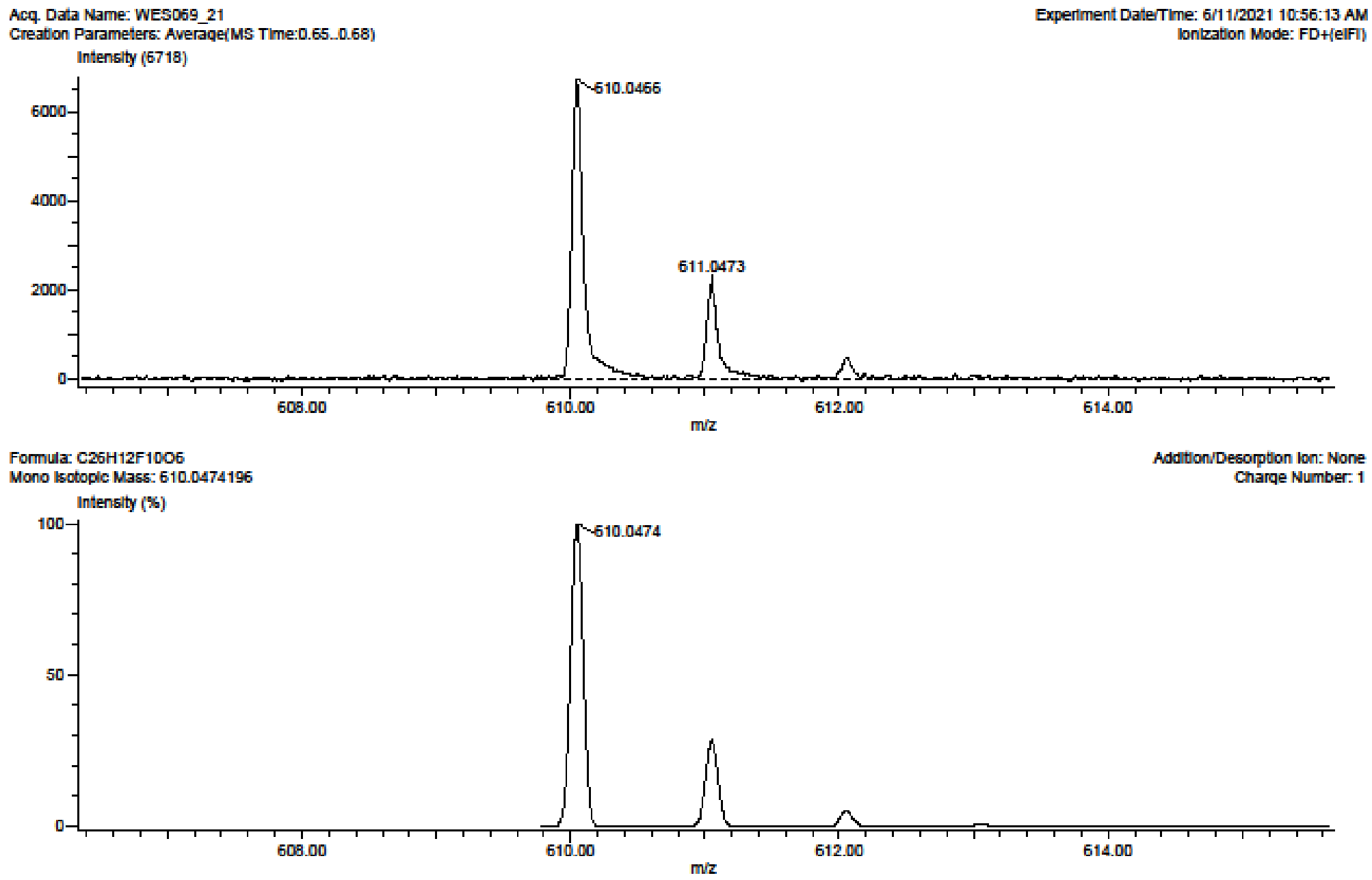

Bis(2,6-di(hept-6-en-1-yl)-4-tert-butylphenyl) 2,6-bis(allyloxy)terephthalate (11a). ^{1}**H-NMR (400 MHz, CDCl**$_3$**)**

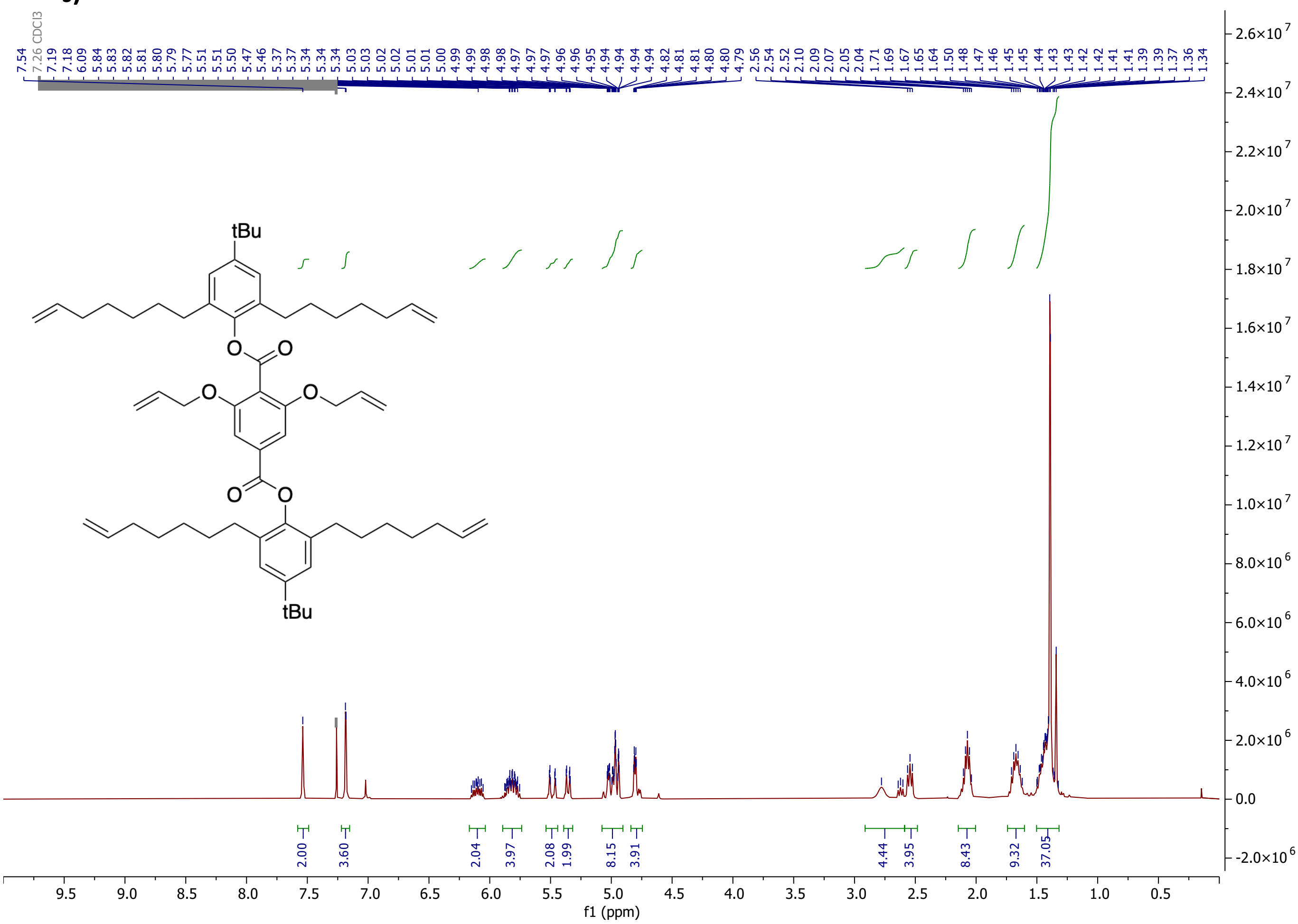

$^{13}C\{^{1}H\}$-NMR (101 MHz, $CDCl_3$)

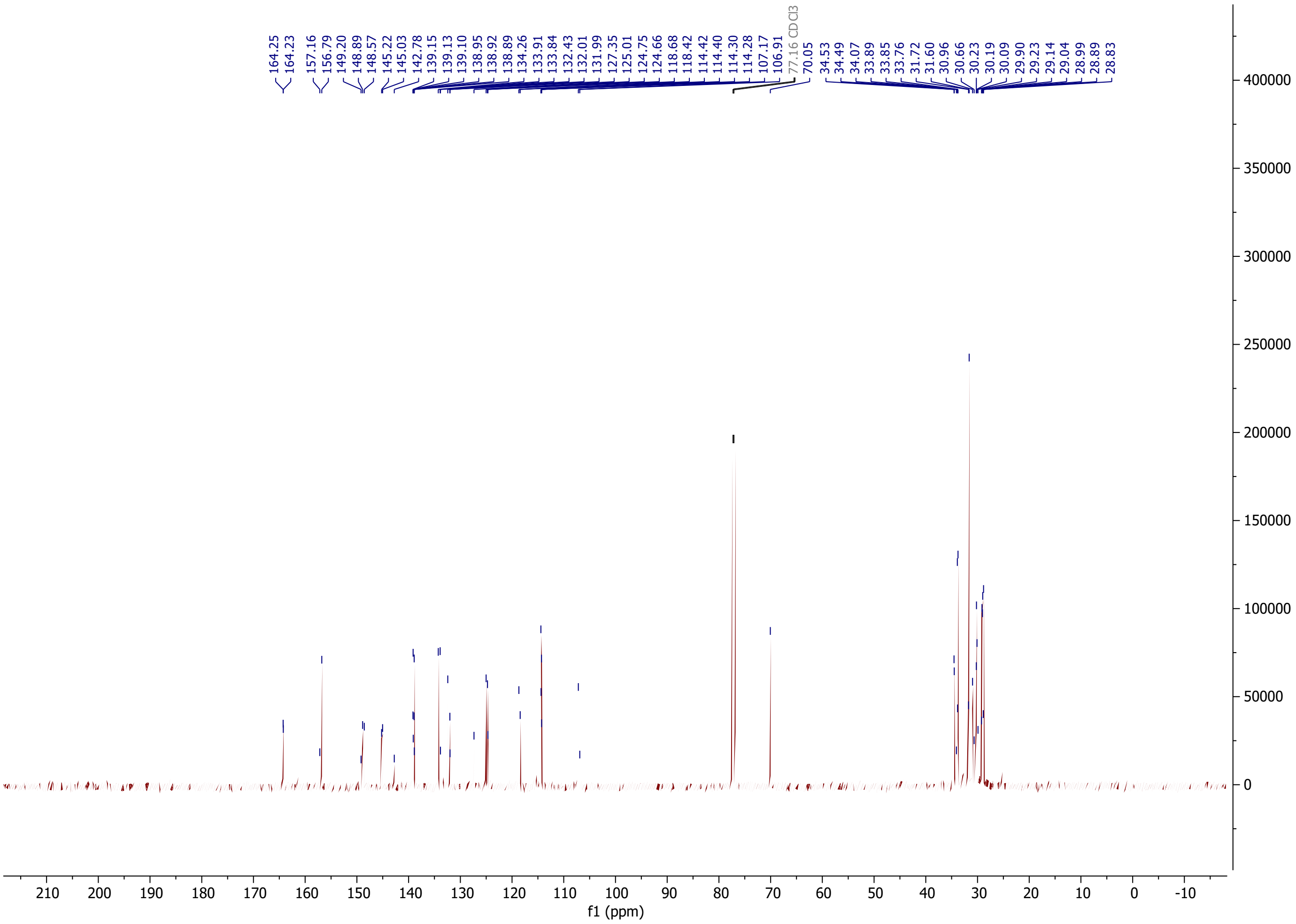

HRMS

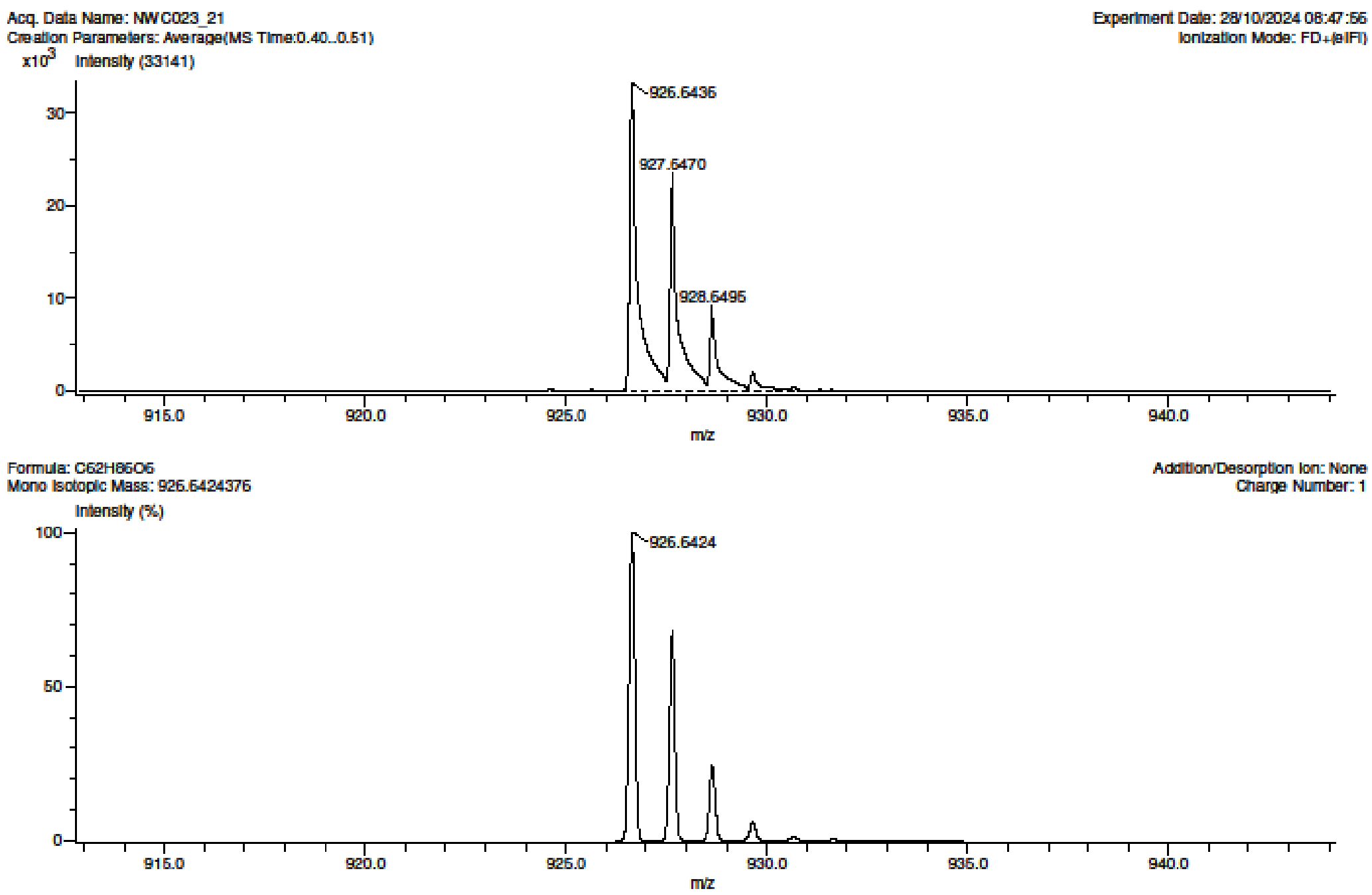

Bis(4-(tert-butyl)-2,6-di(hept-6-en-1-yl)phenyl) 2,6-dihydroxyterephthalate (11b). ^{1}H-NMR (400 MHz, CDCl$_3$)

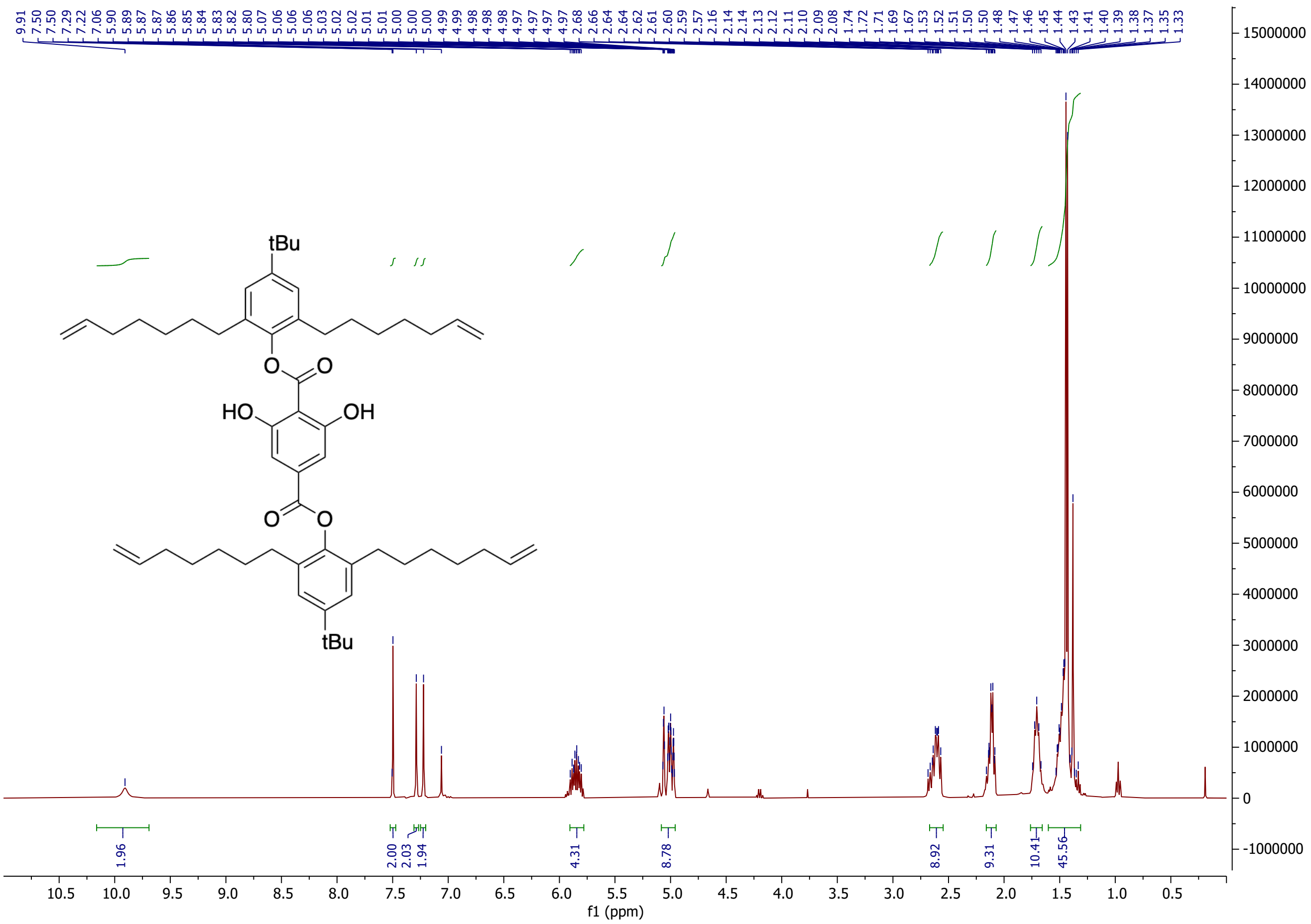

^{13}C{^{1}H}-NMR (101 MHz, CDCl$_3$)

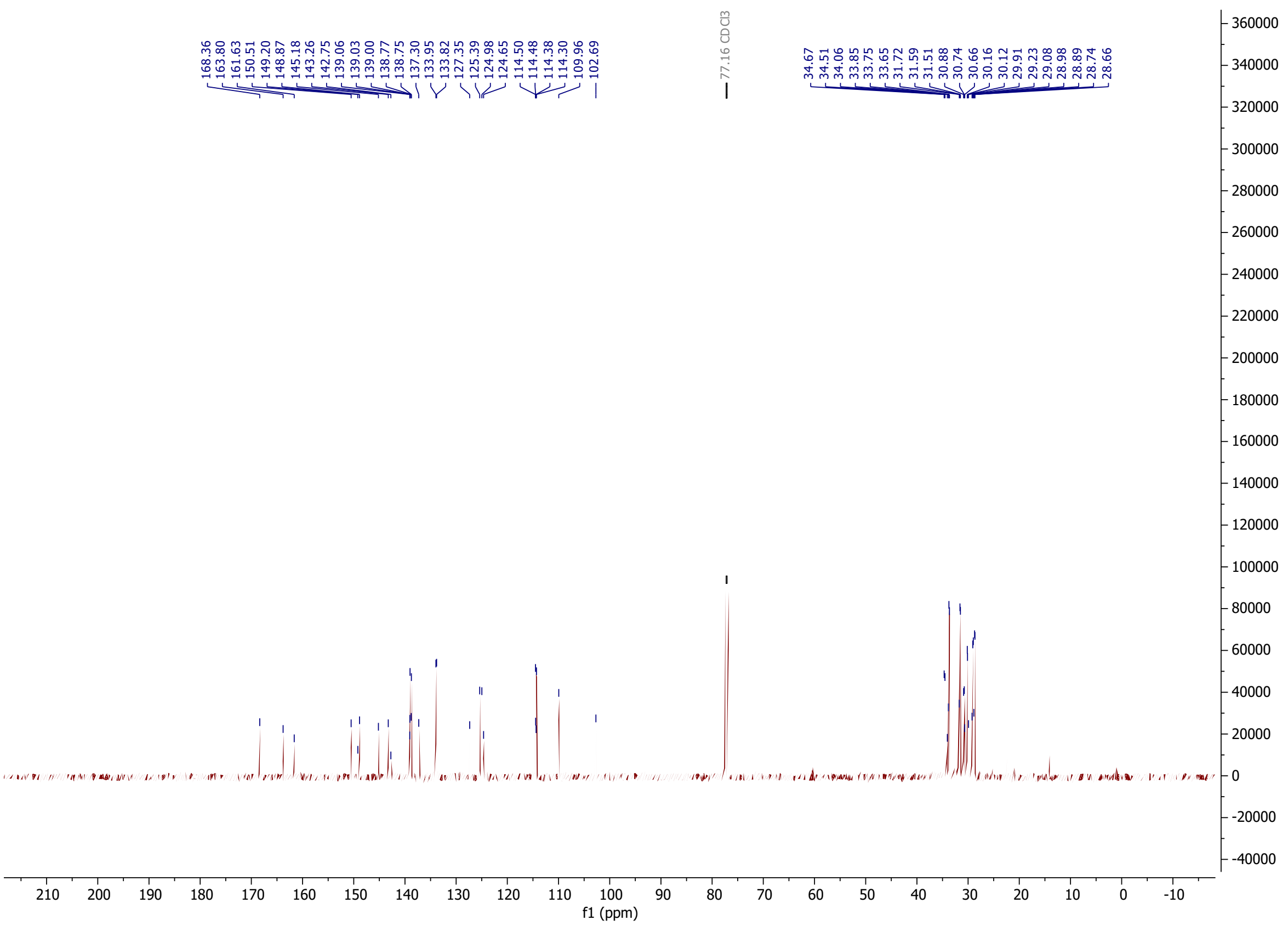

HRMS

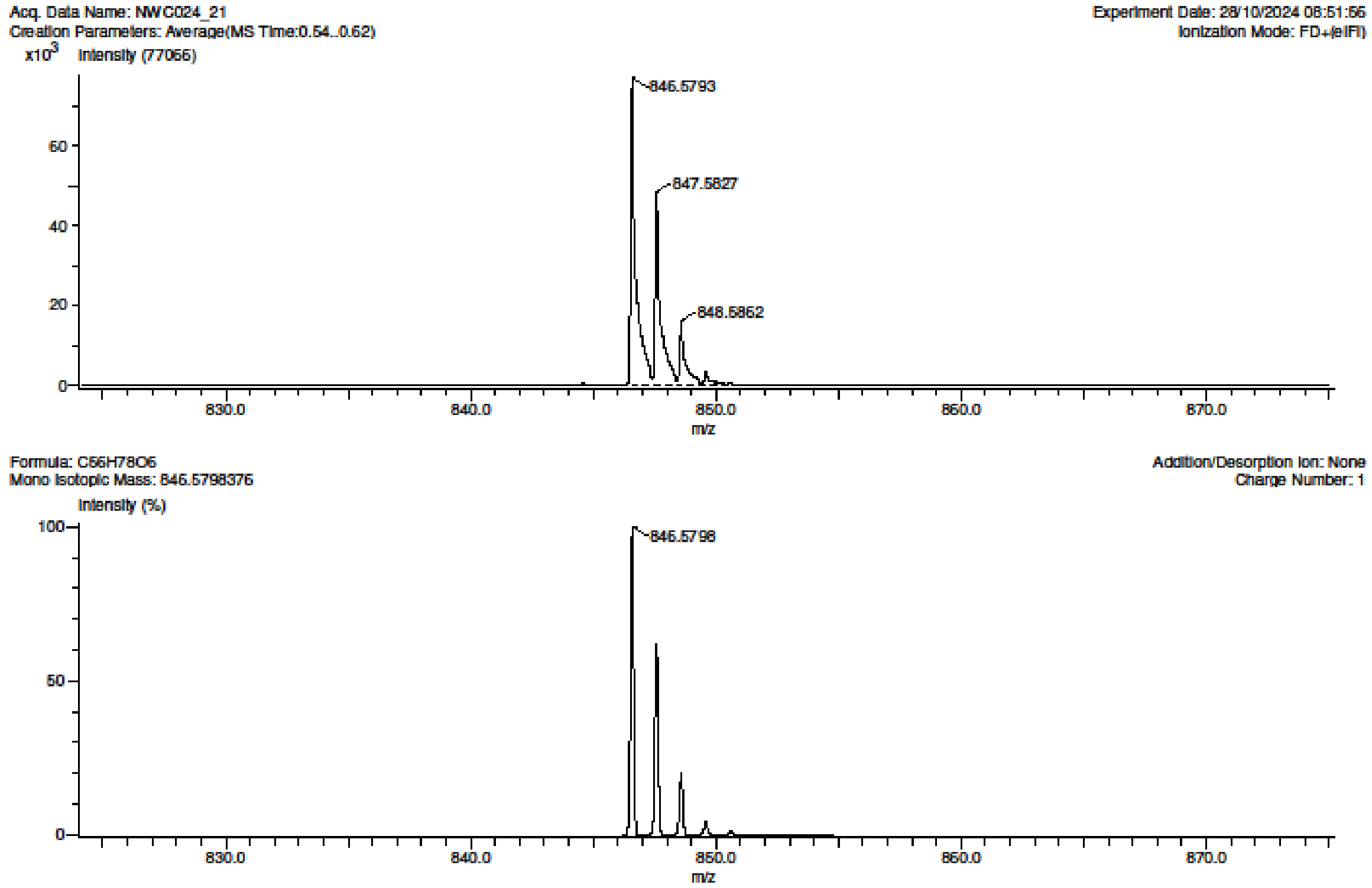

Precatenane precursor (12a). ^{1}H-NMR (400 MHz, $CDCl_3$)

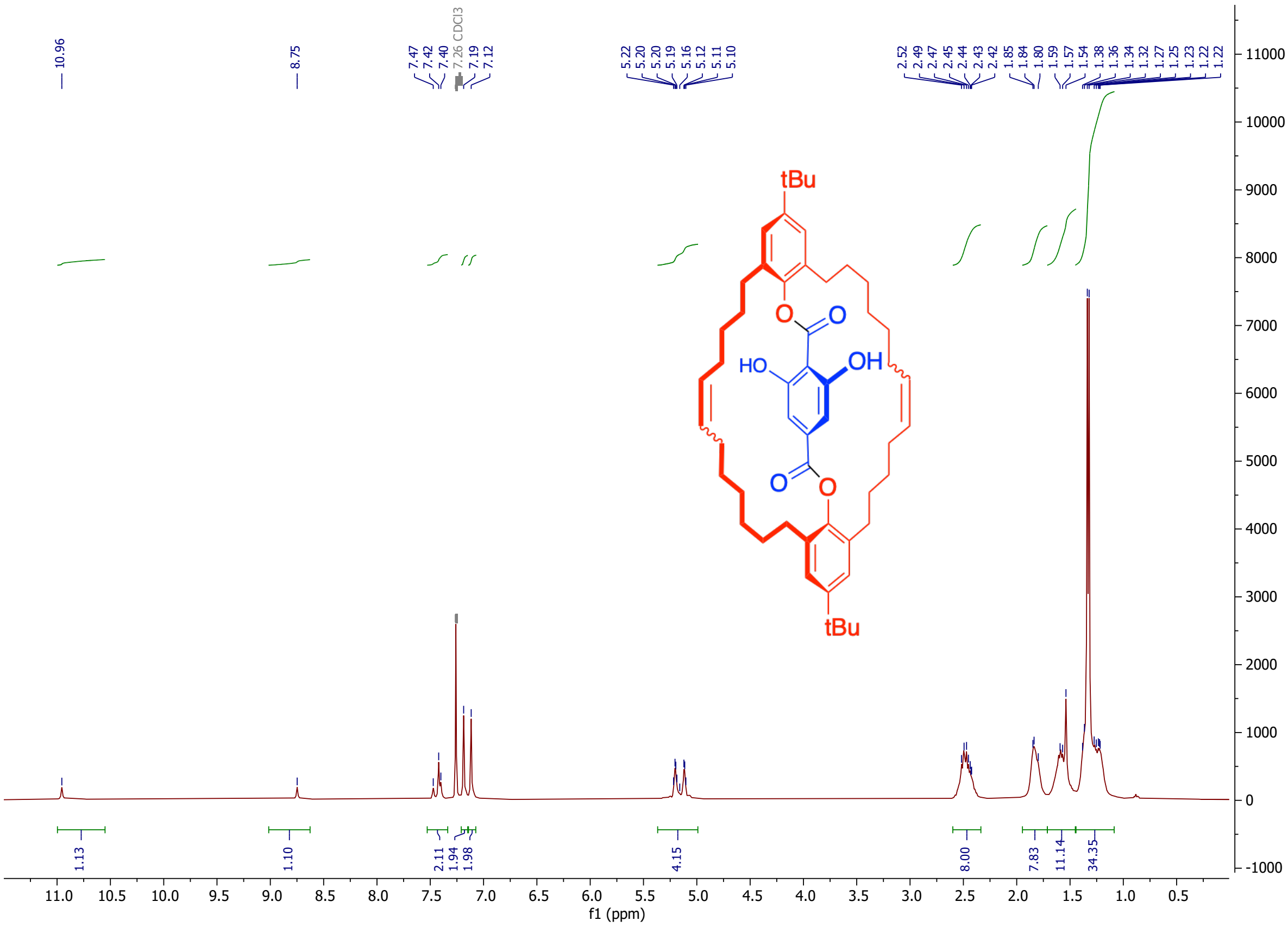

$^{13}C\{^{1}H\}$-NMR (101 MHz, $CDCl_3$)

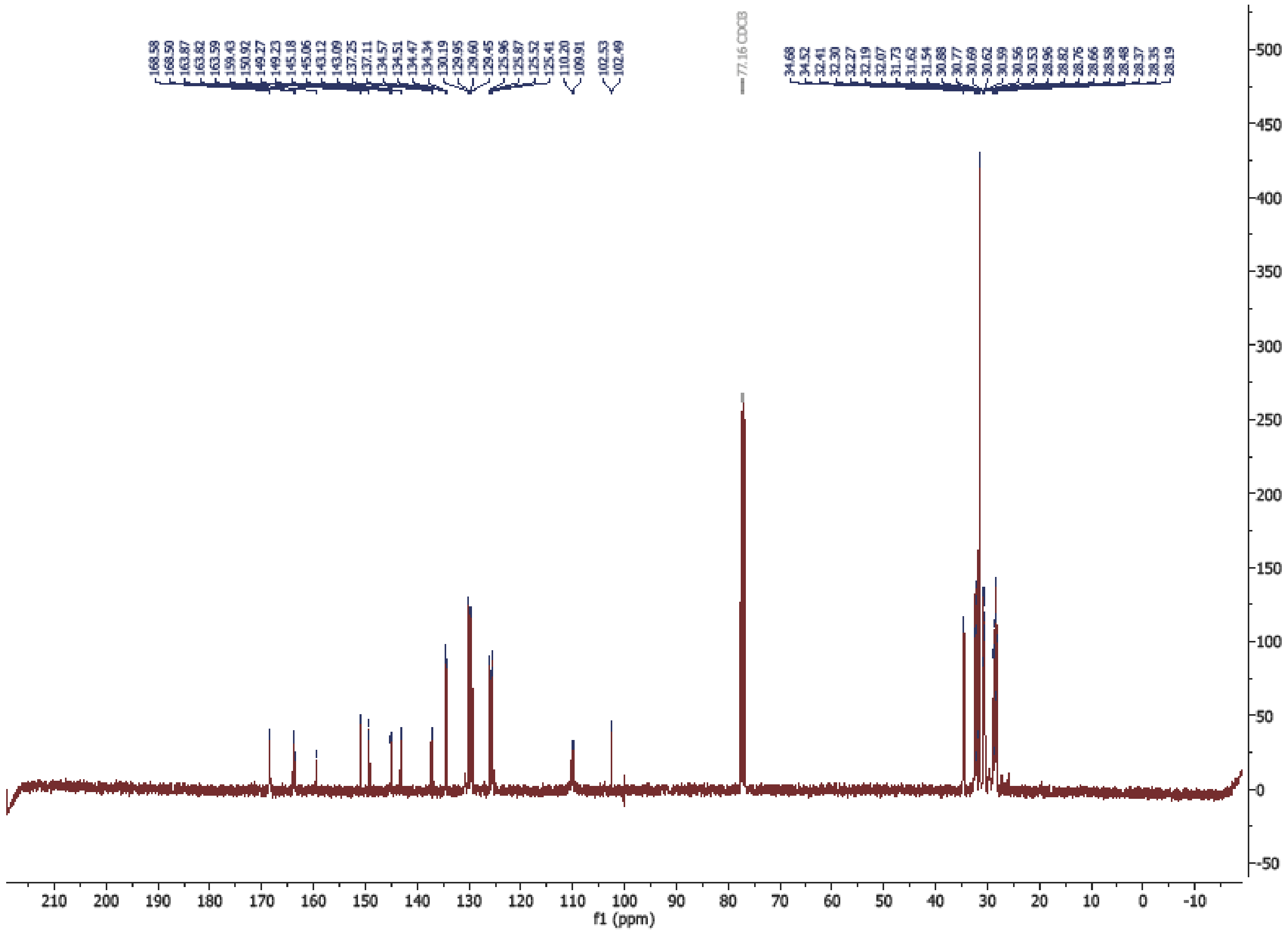

HRMS

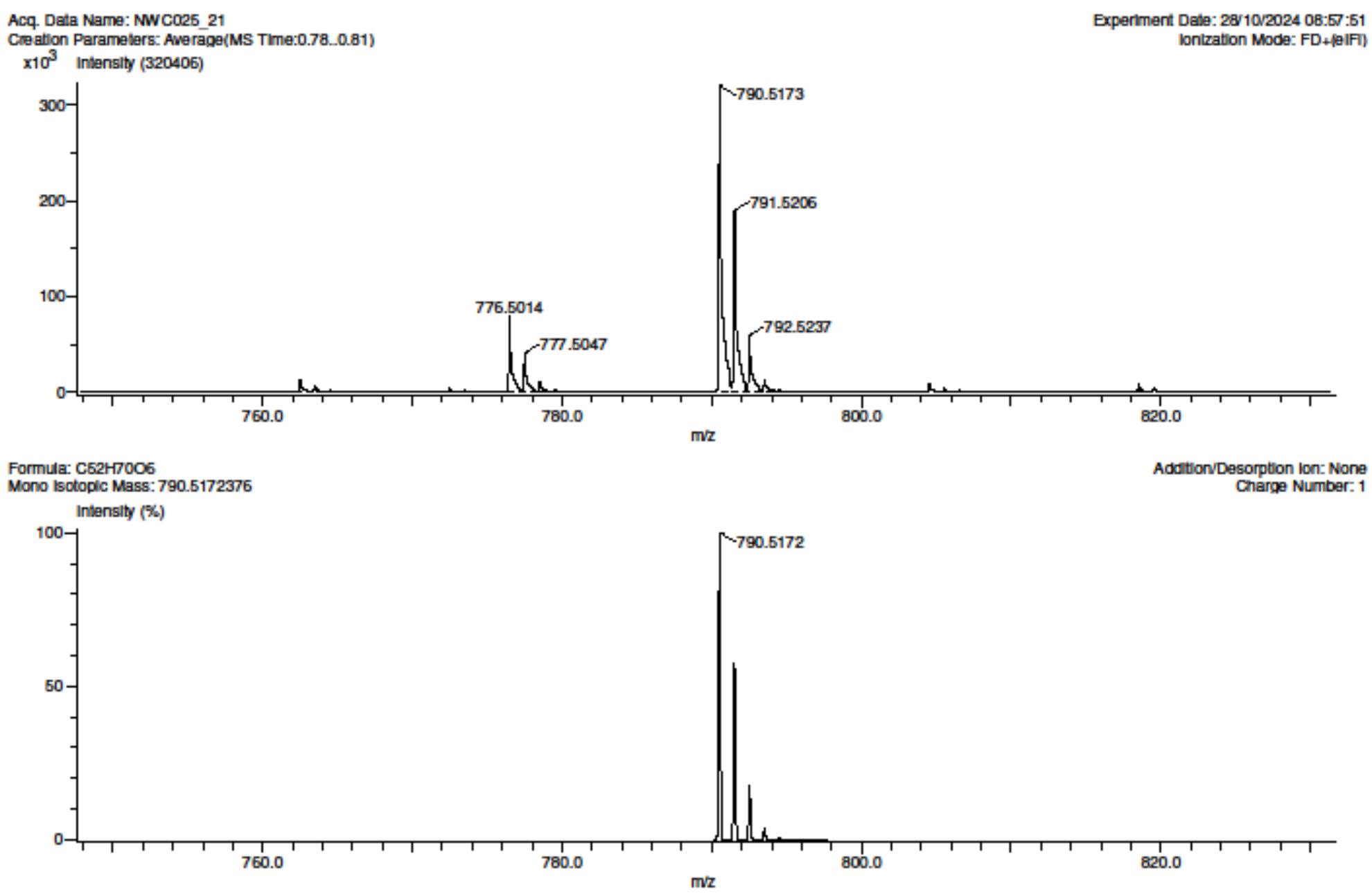

Precatenane precursor 12b. ^{1}H-NMR (400 MHz, $CDCl_3$)

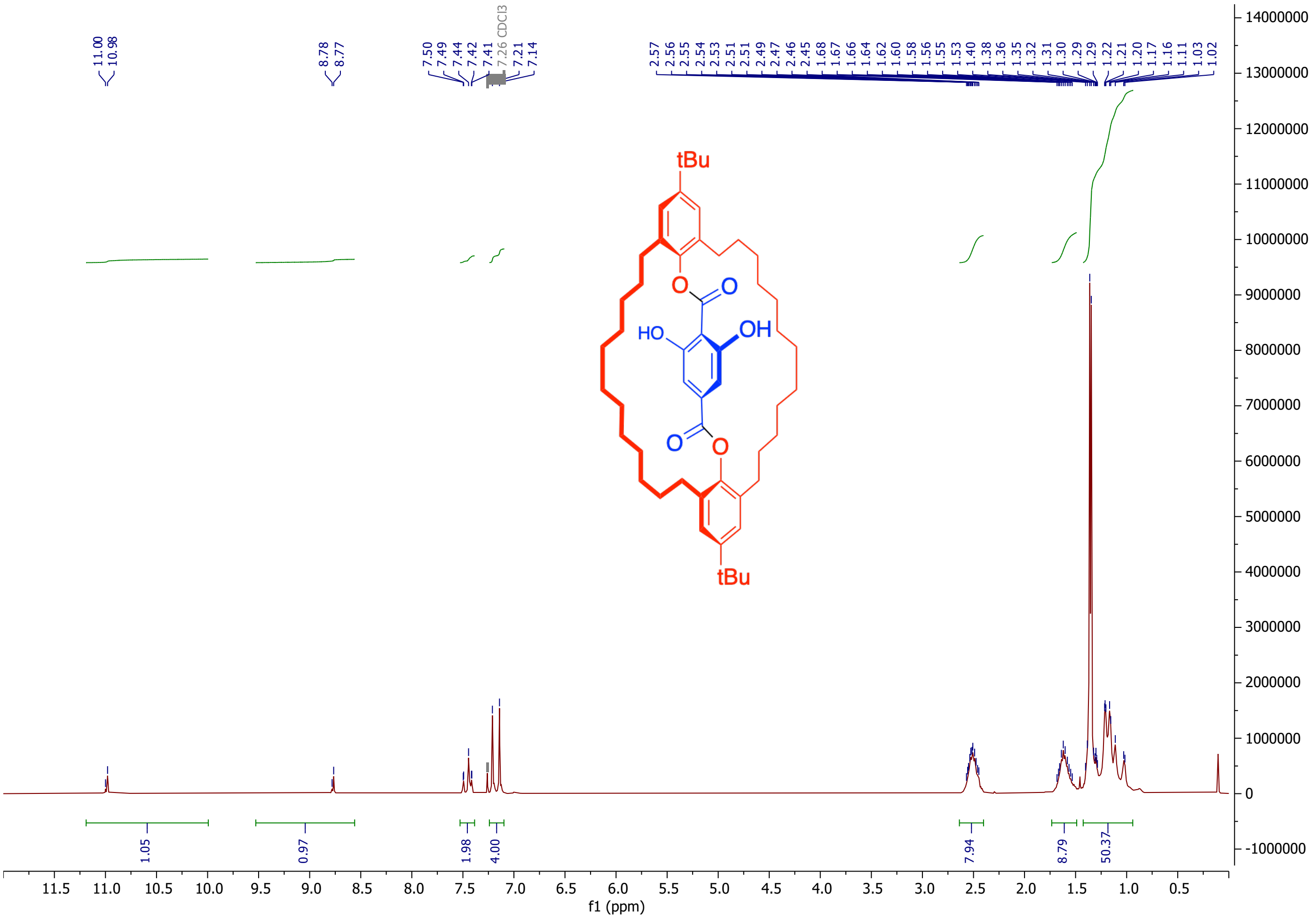

$^{13}C\{^{1}H\}$-NMR (101 MHz, $CDCl_3$)

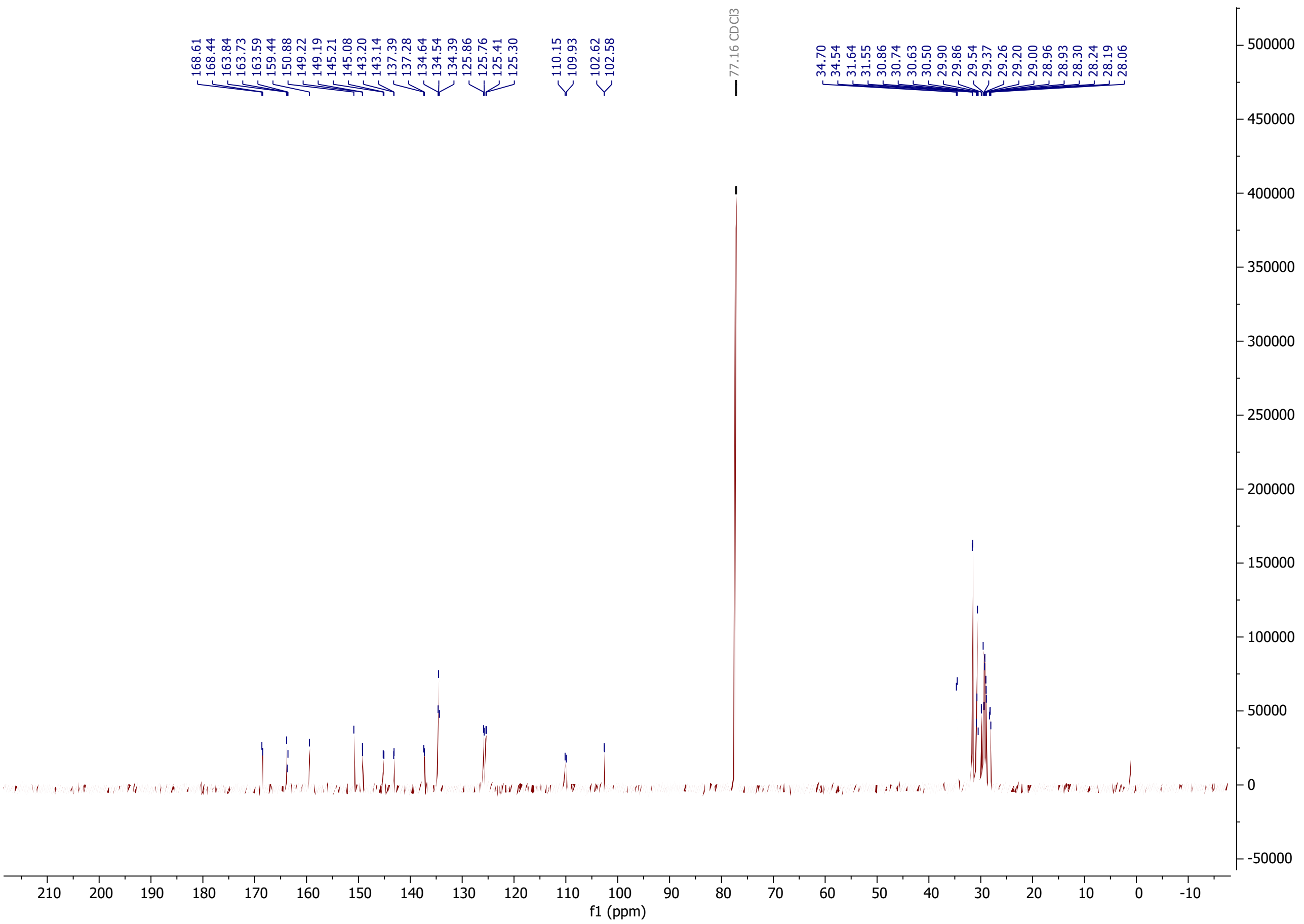

HRMS

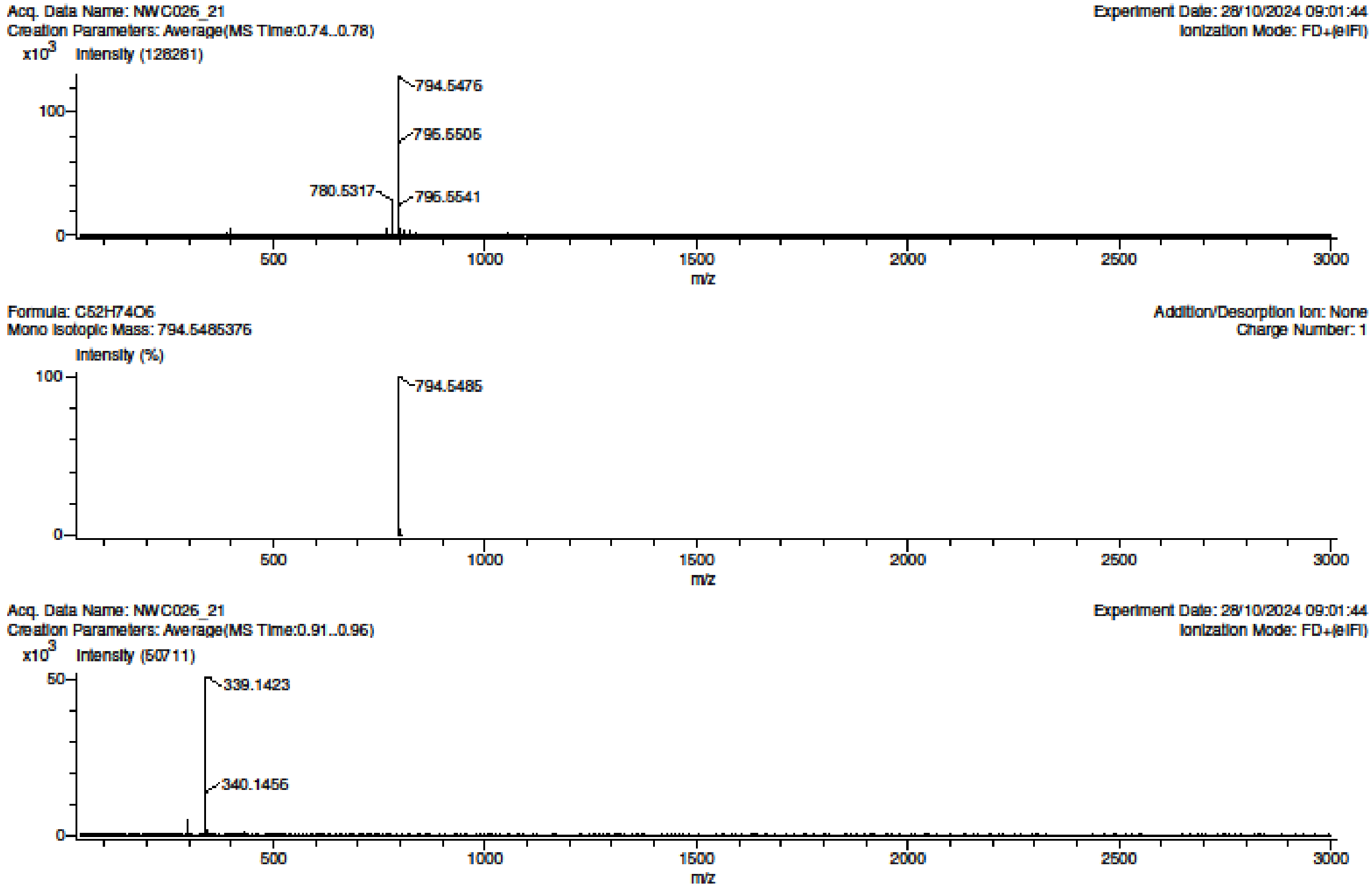

Precatenane precursor 13. ^{1}H-NMR (400 MHz, $CDCl_3$)

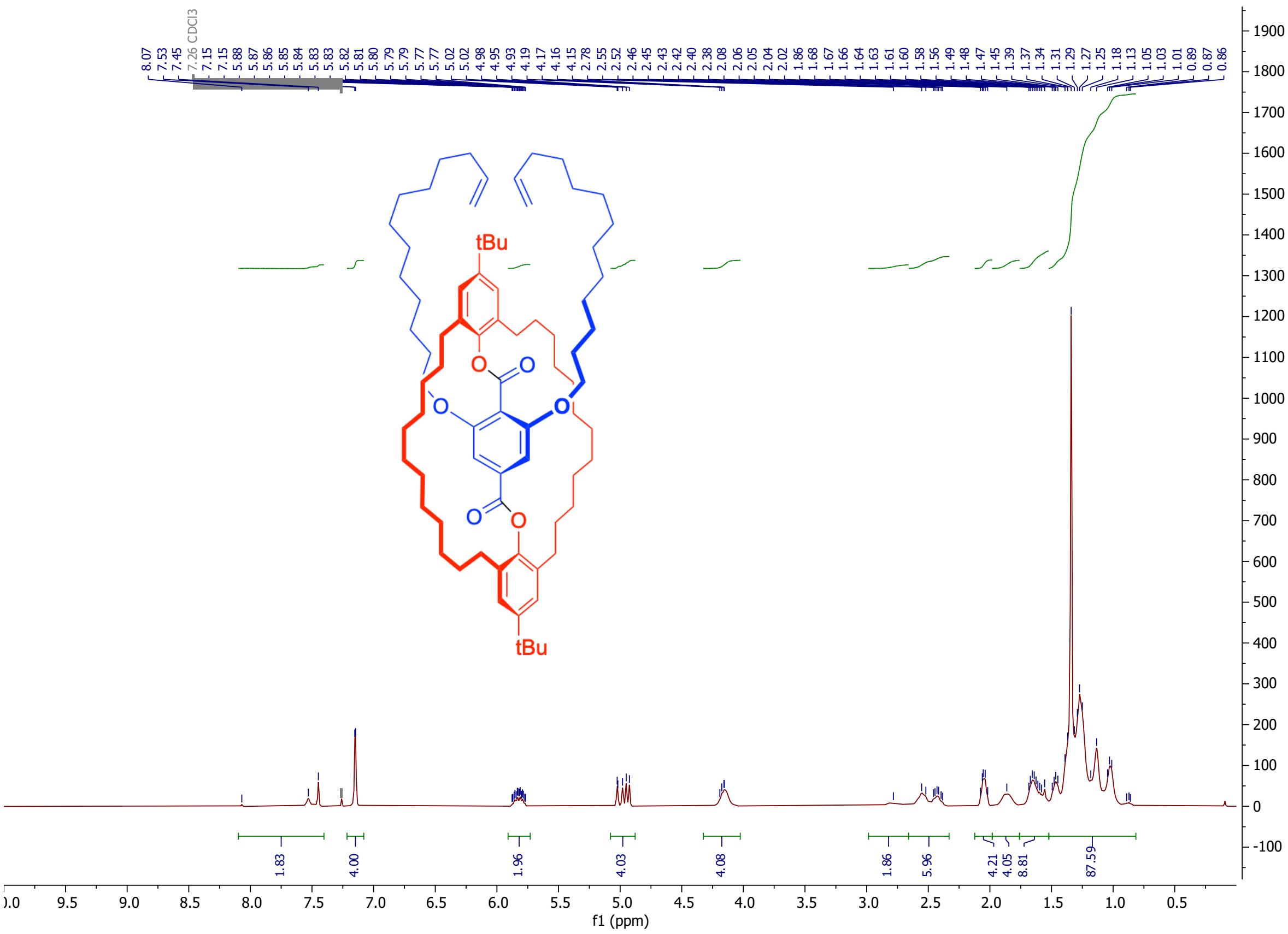

$^{13}C\{^{1}H\}$-NMR (101 MHz, $CDCl_3$)

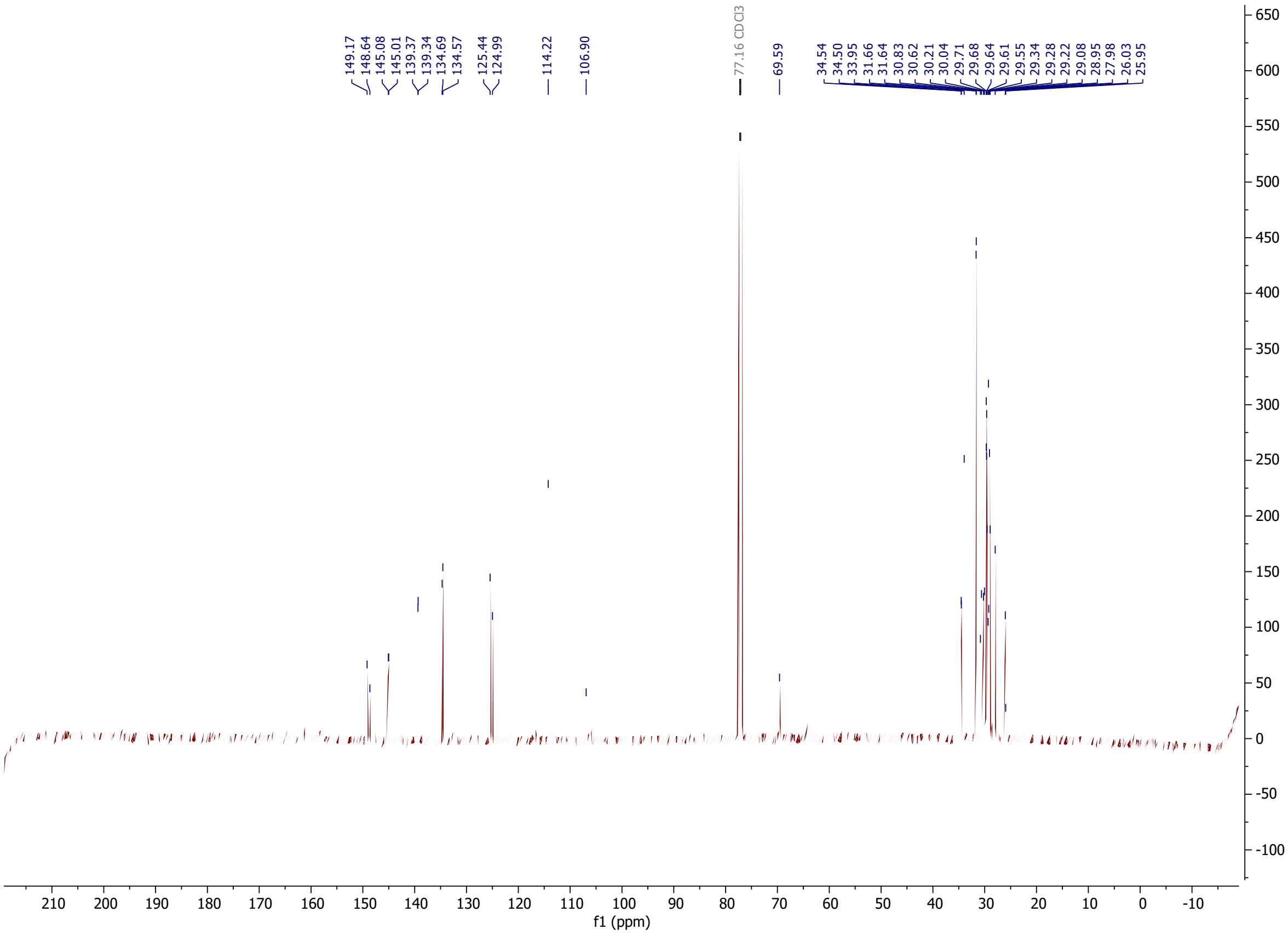

HRMS

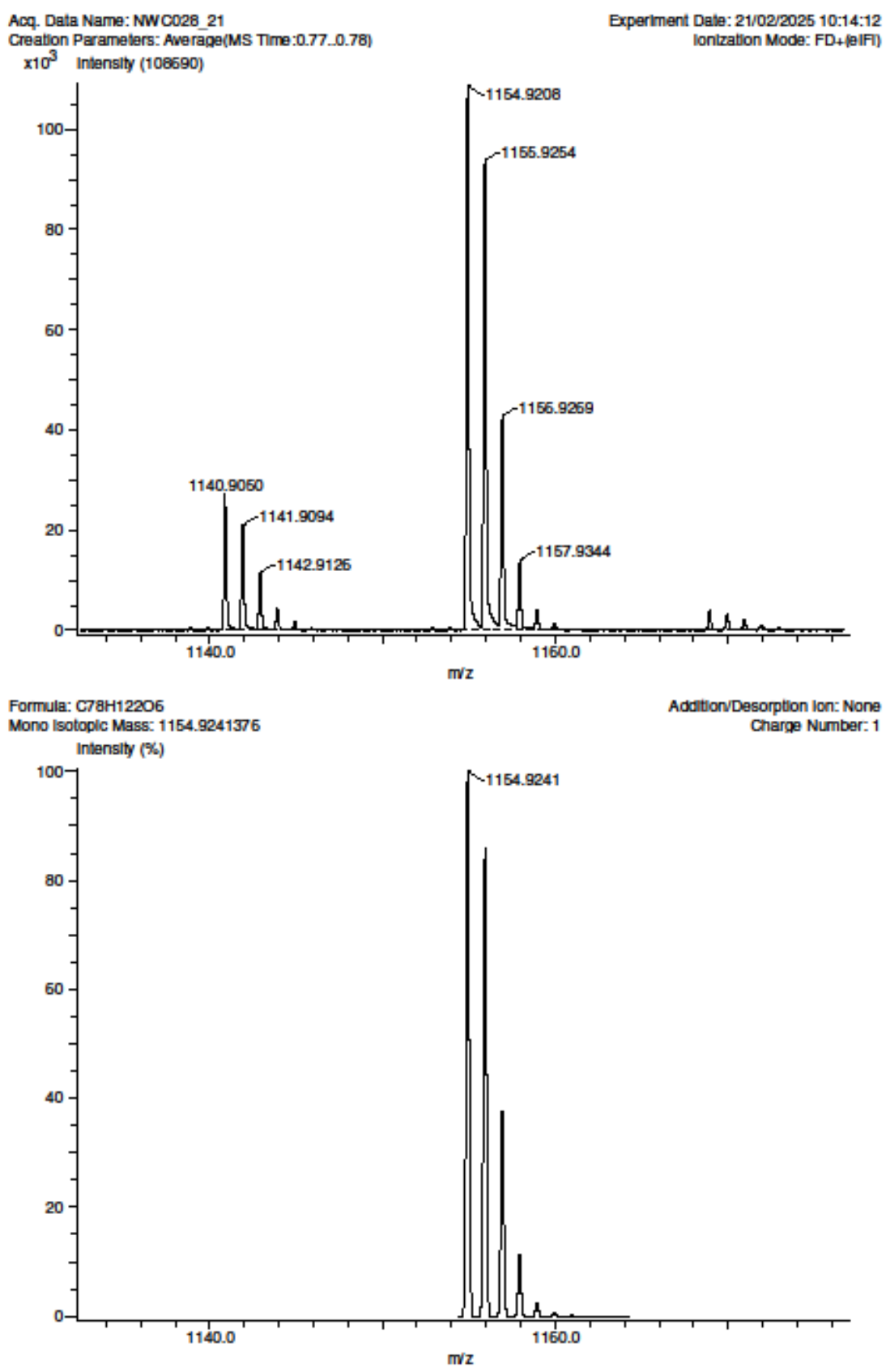

Precatenane 14a. ¹H-NMR (400 MHz, CDCl₃)

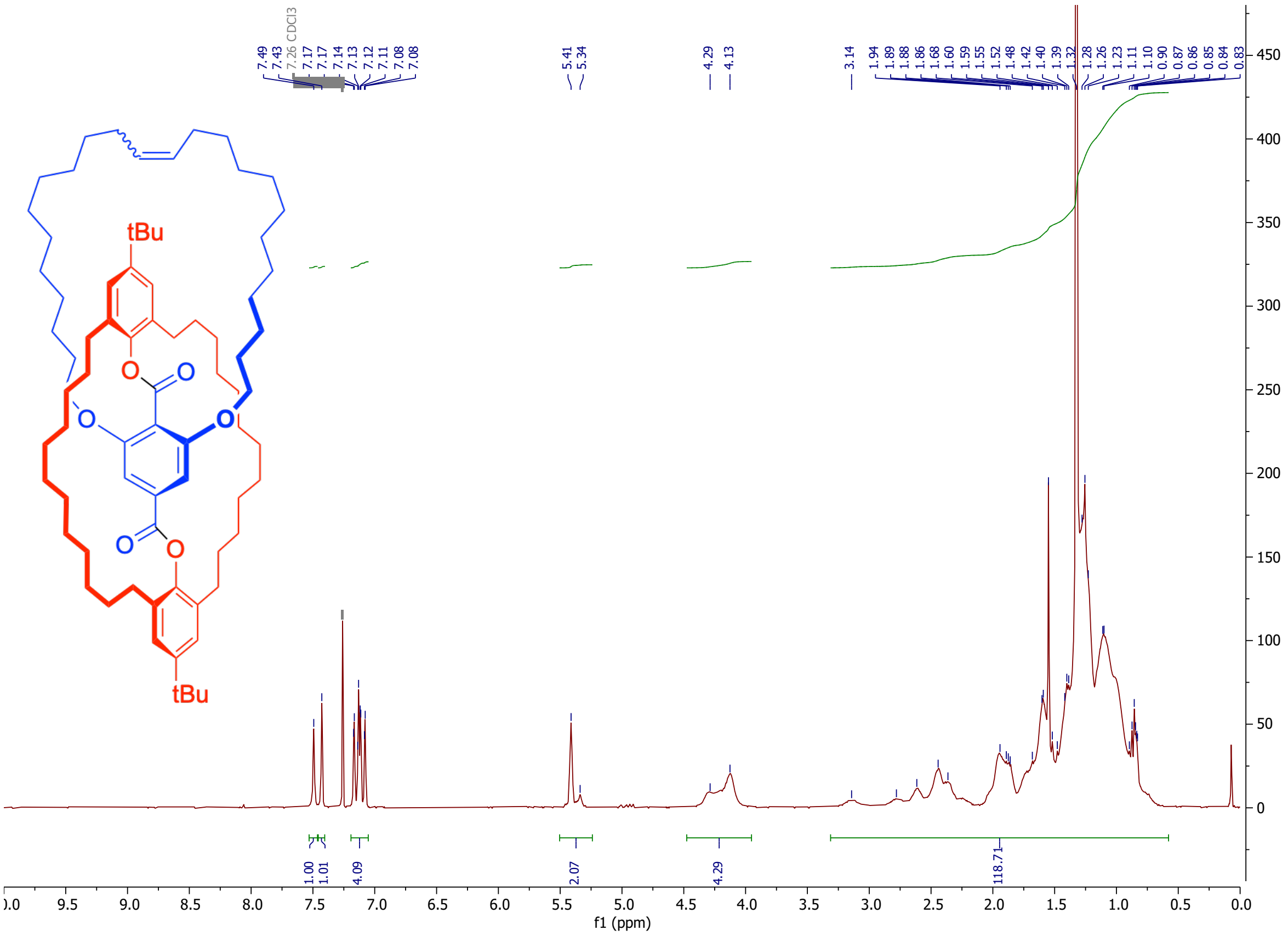

¹³C{¹H}-NMR (101 MHz, CDCl₃)

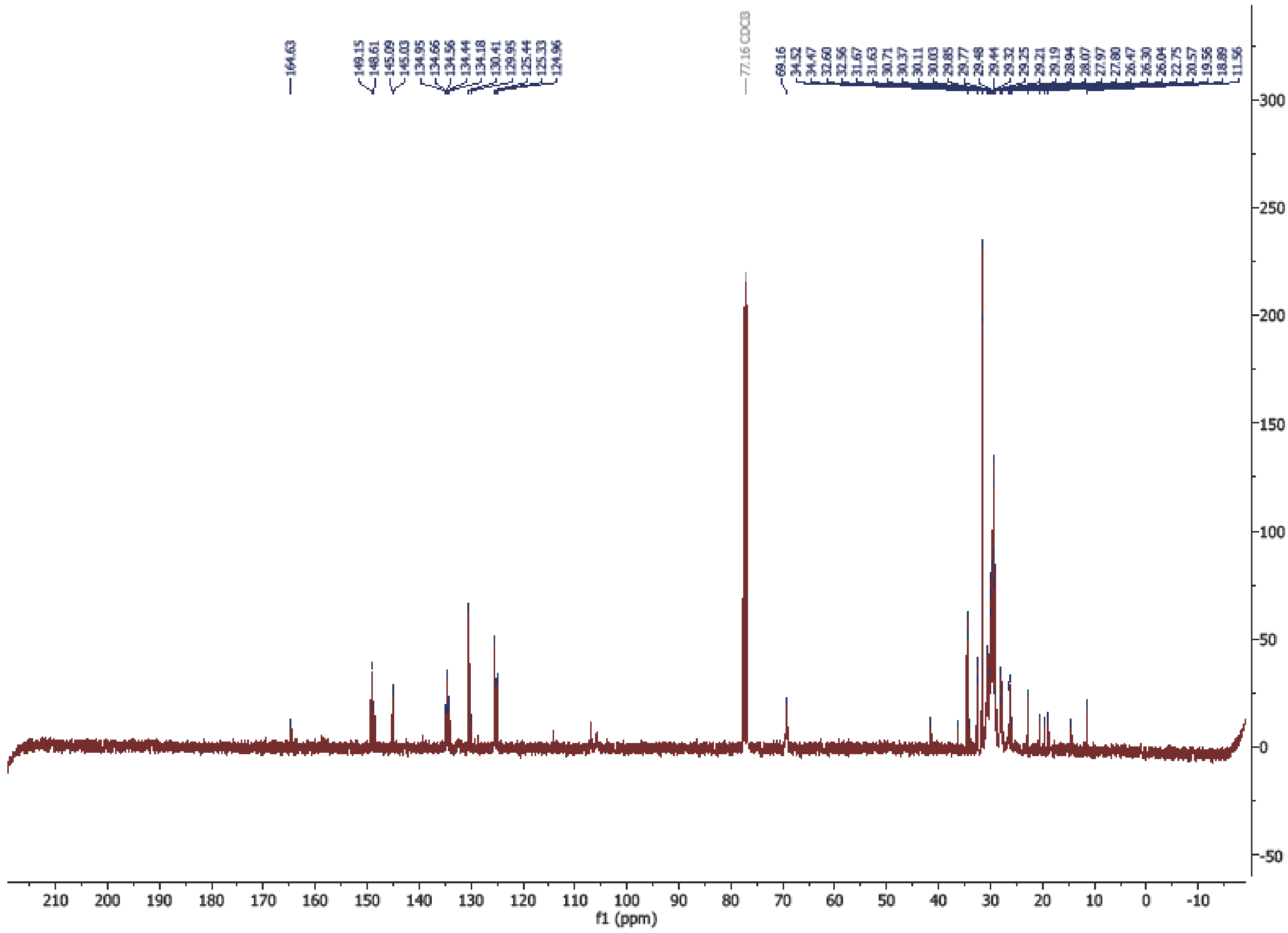

HRMS

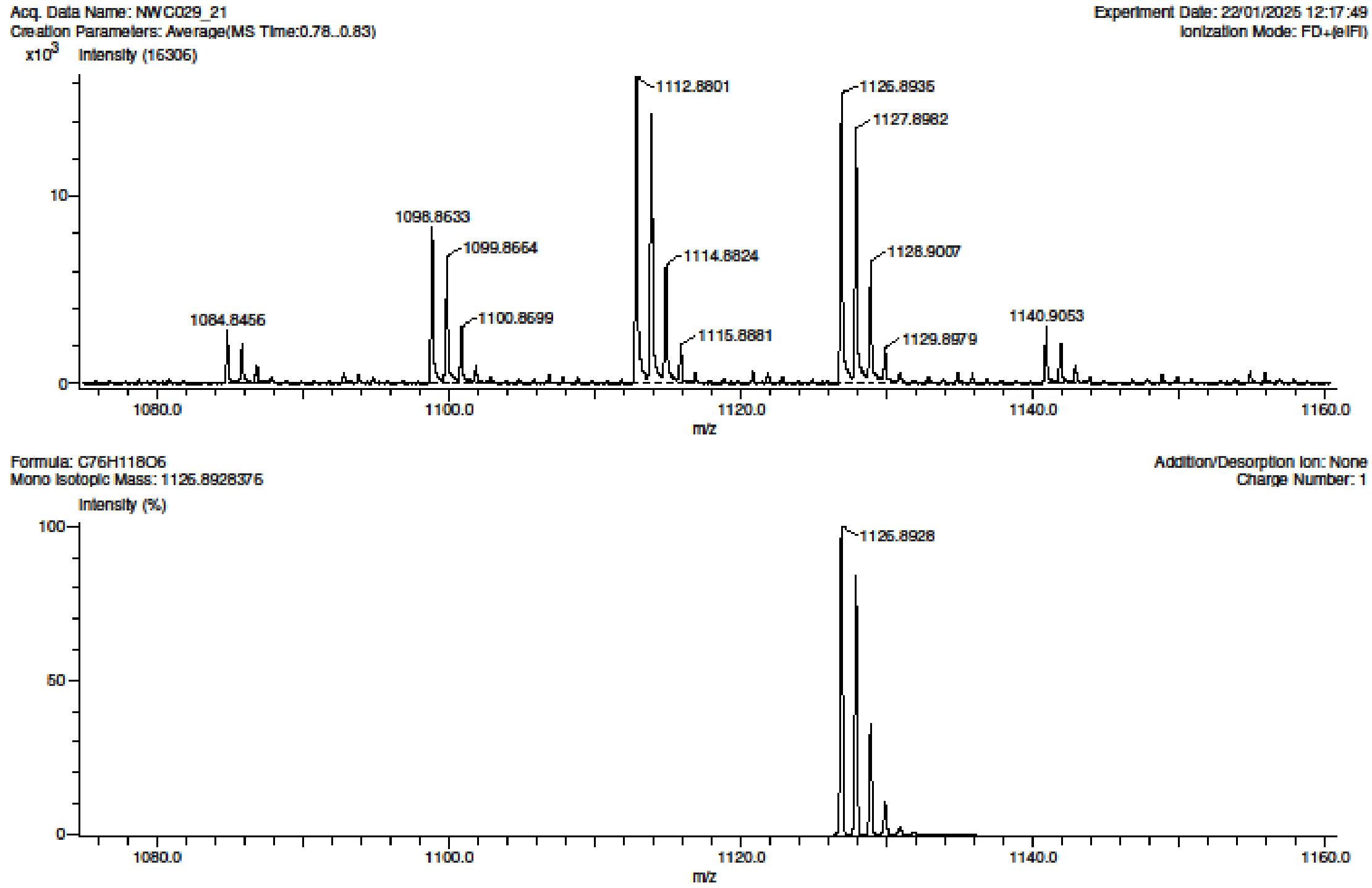

Precatenane 14b. ^{1}H-NMR (400 MHz, $CDCl_3$)

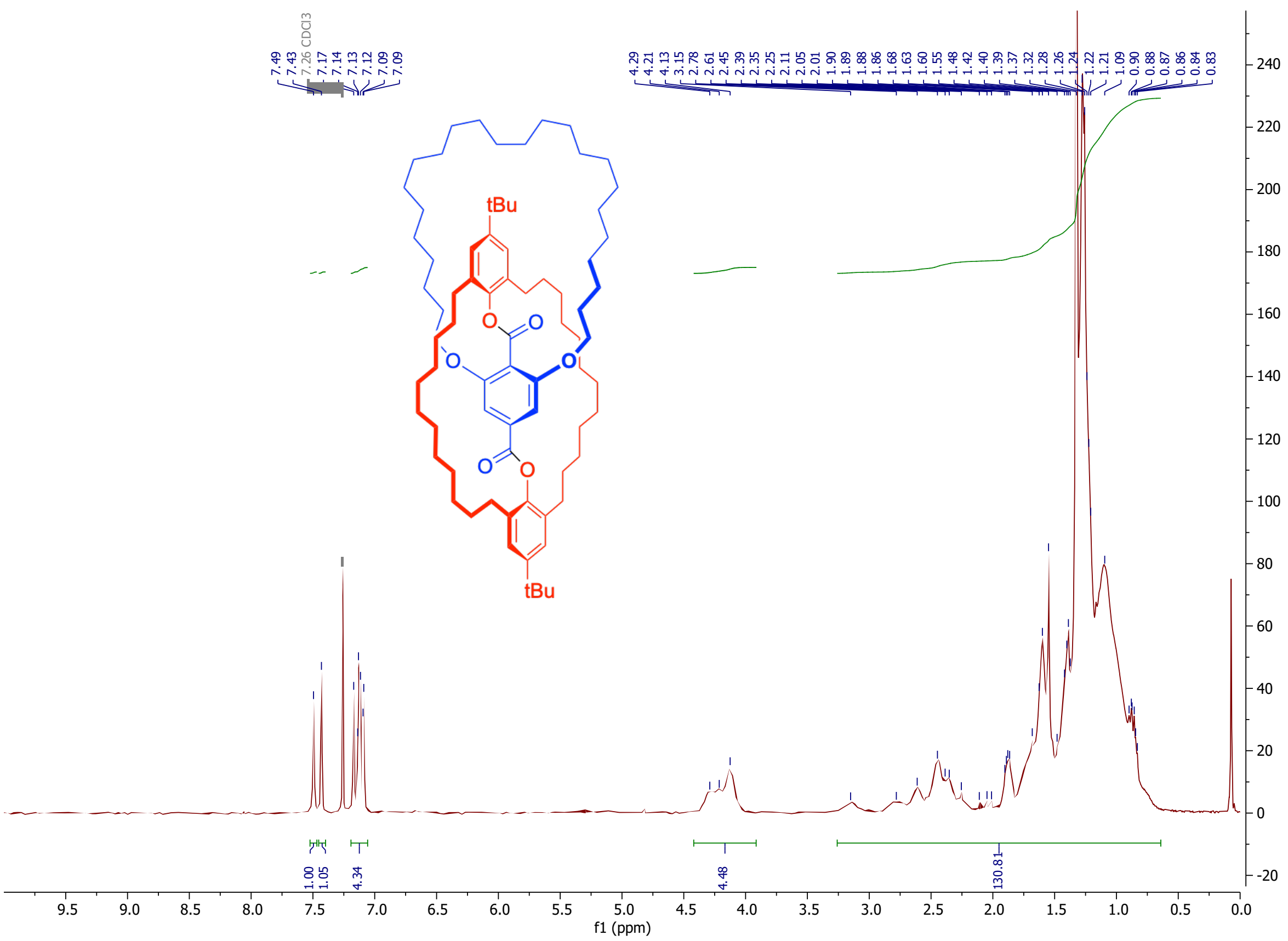

$^{13}C\{^1H\}$-NMR (101 MHz, $CDCl_3$)

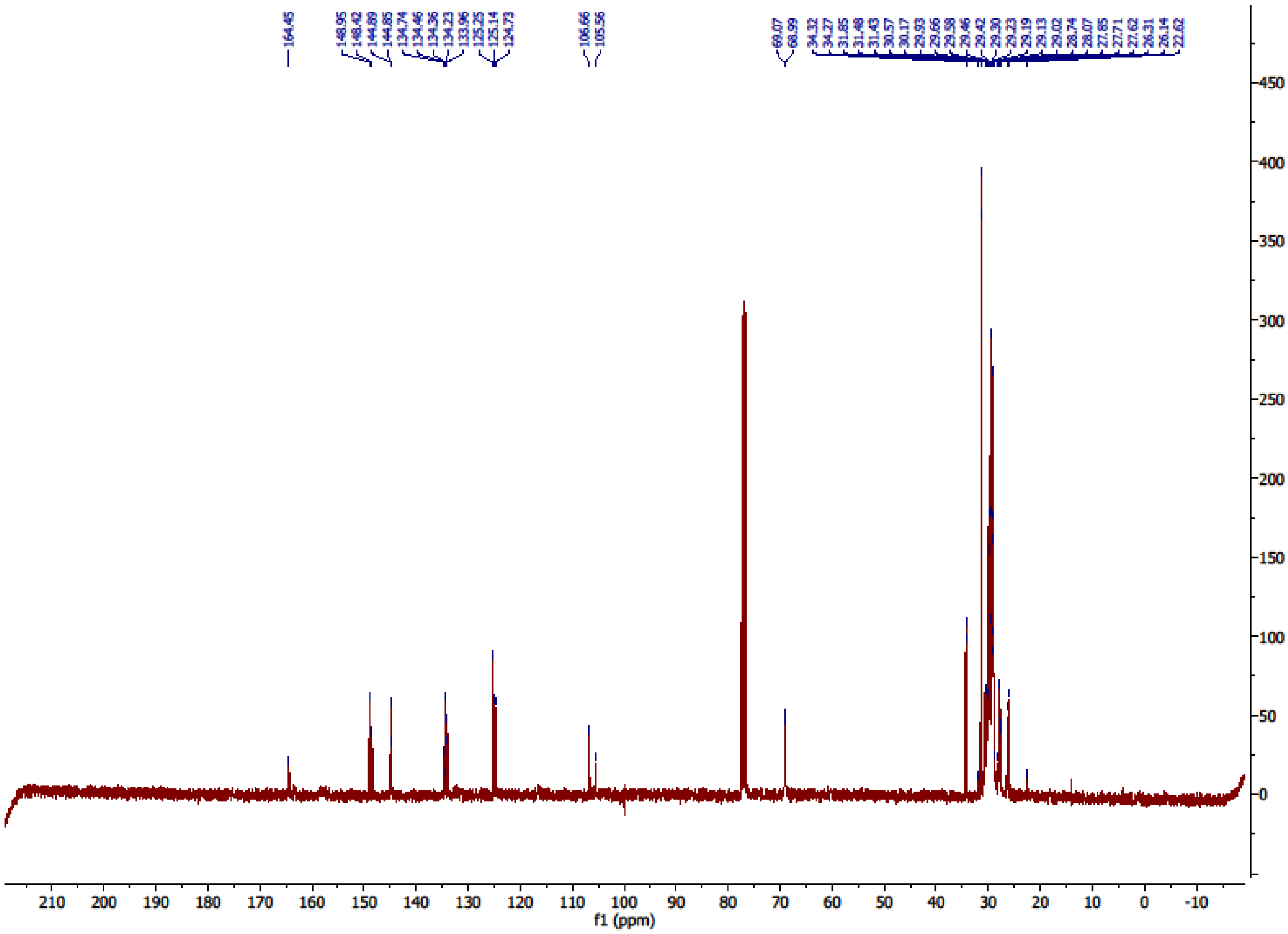

HRMS

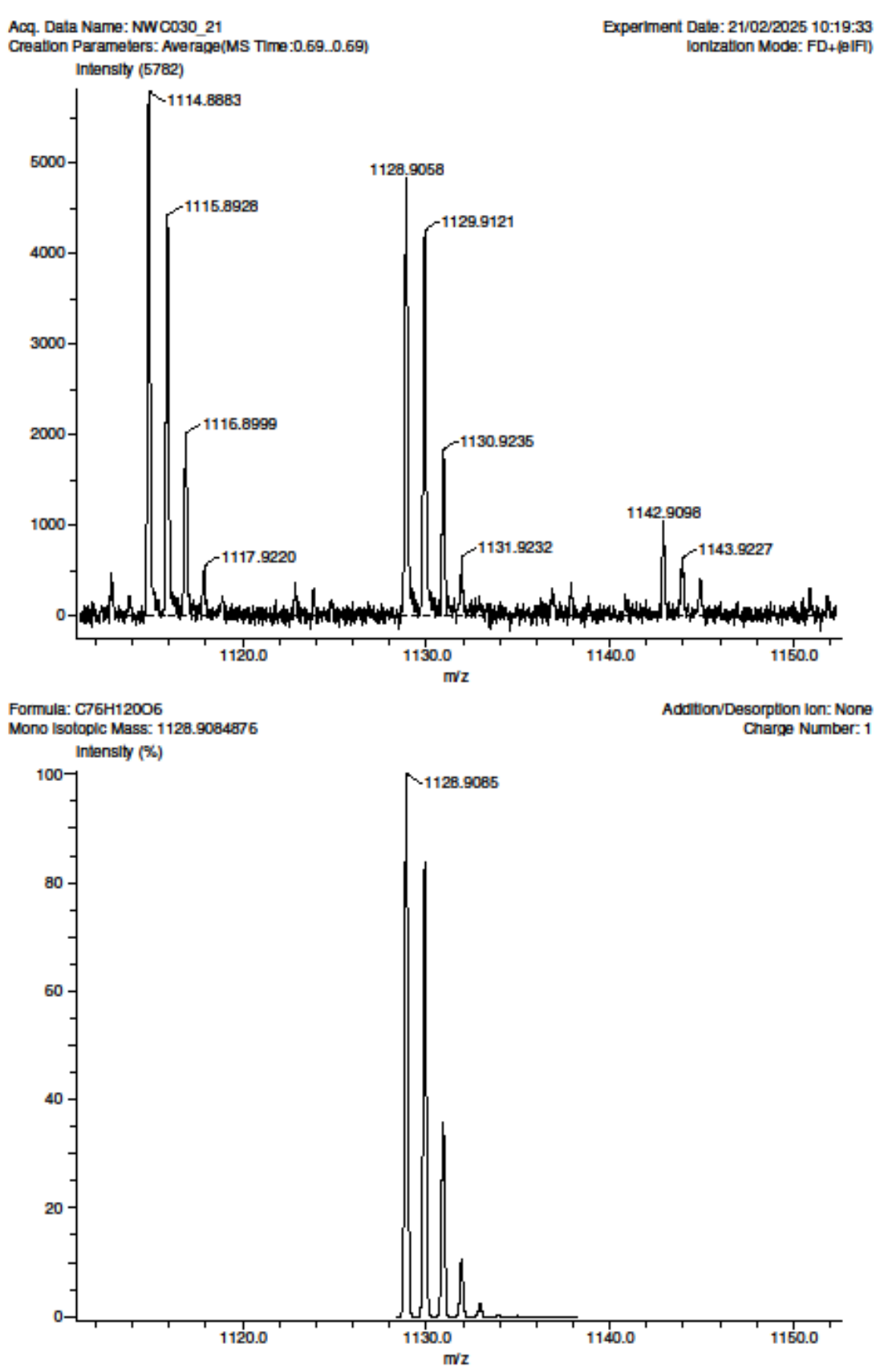

Precatenane 15a. ^{1}H-NMR (400 MHz, $CDCl_3$)

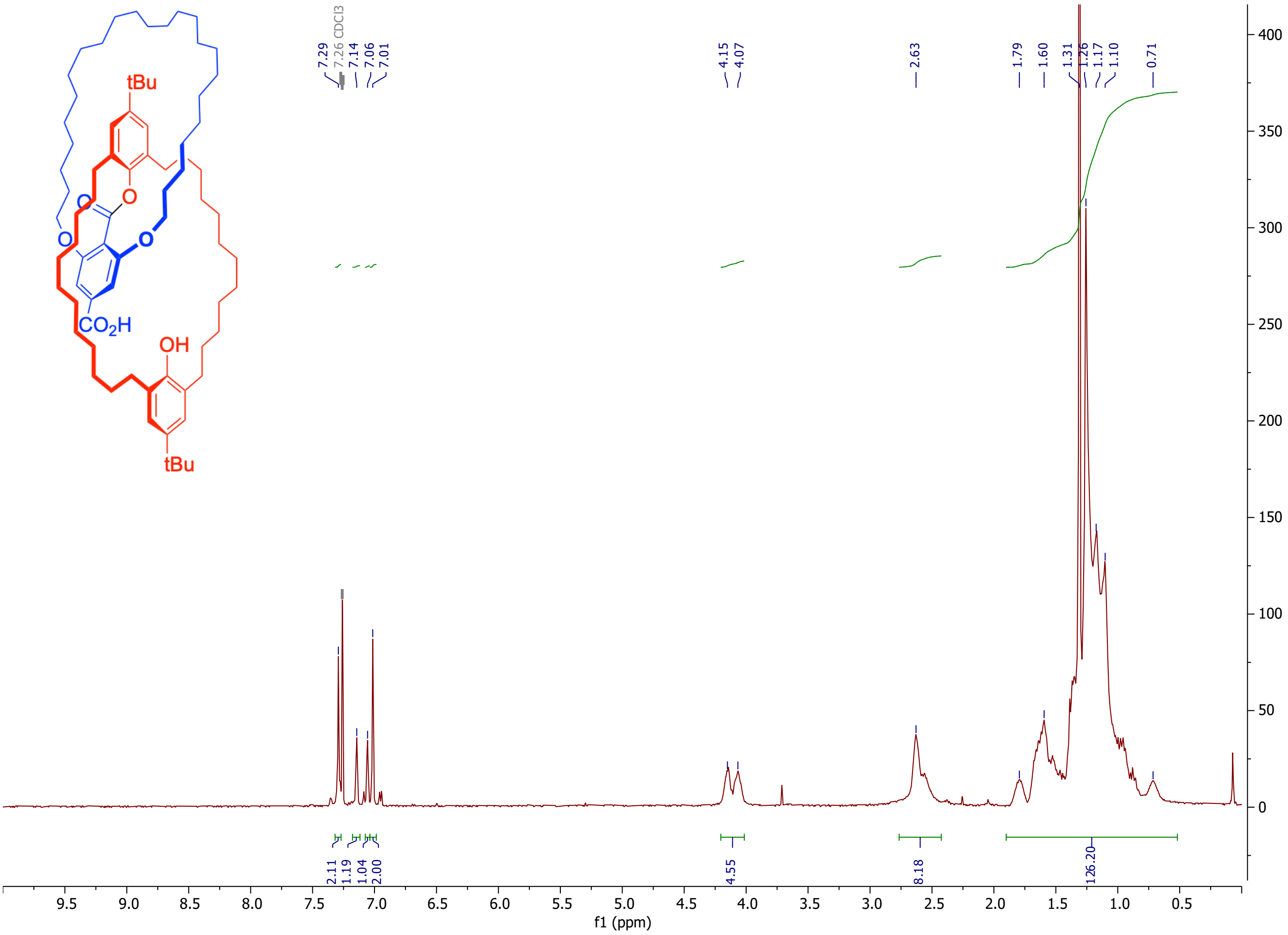

HRMS

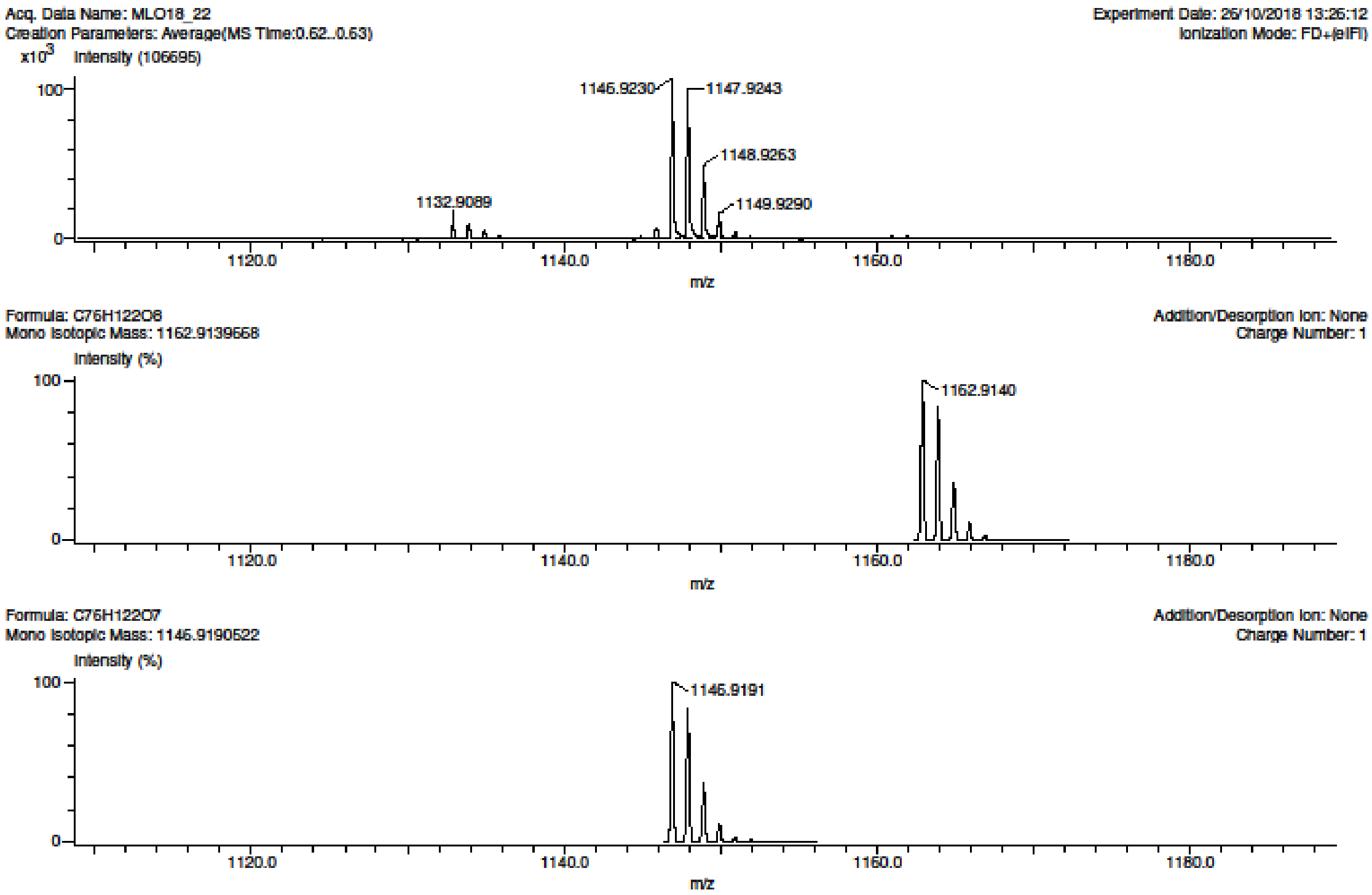

Precatenane 15b. ^{1}H-NMR (400 MHz, $CDCl_3$)

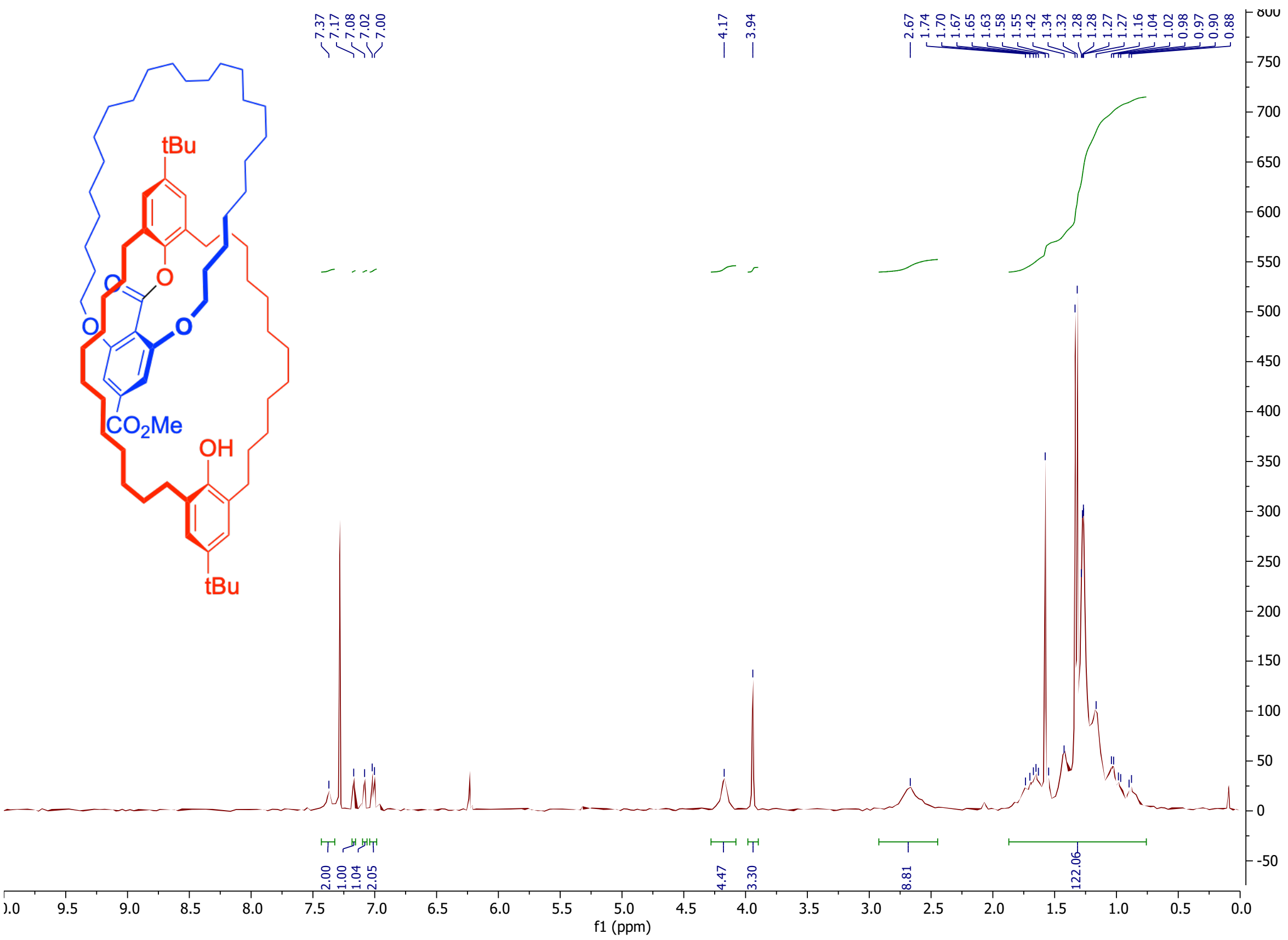

HRMS

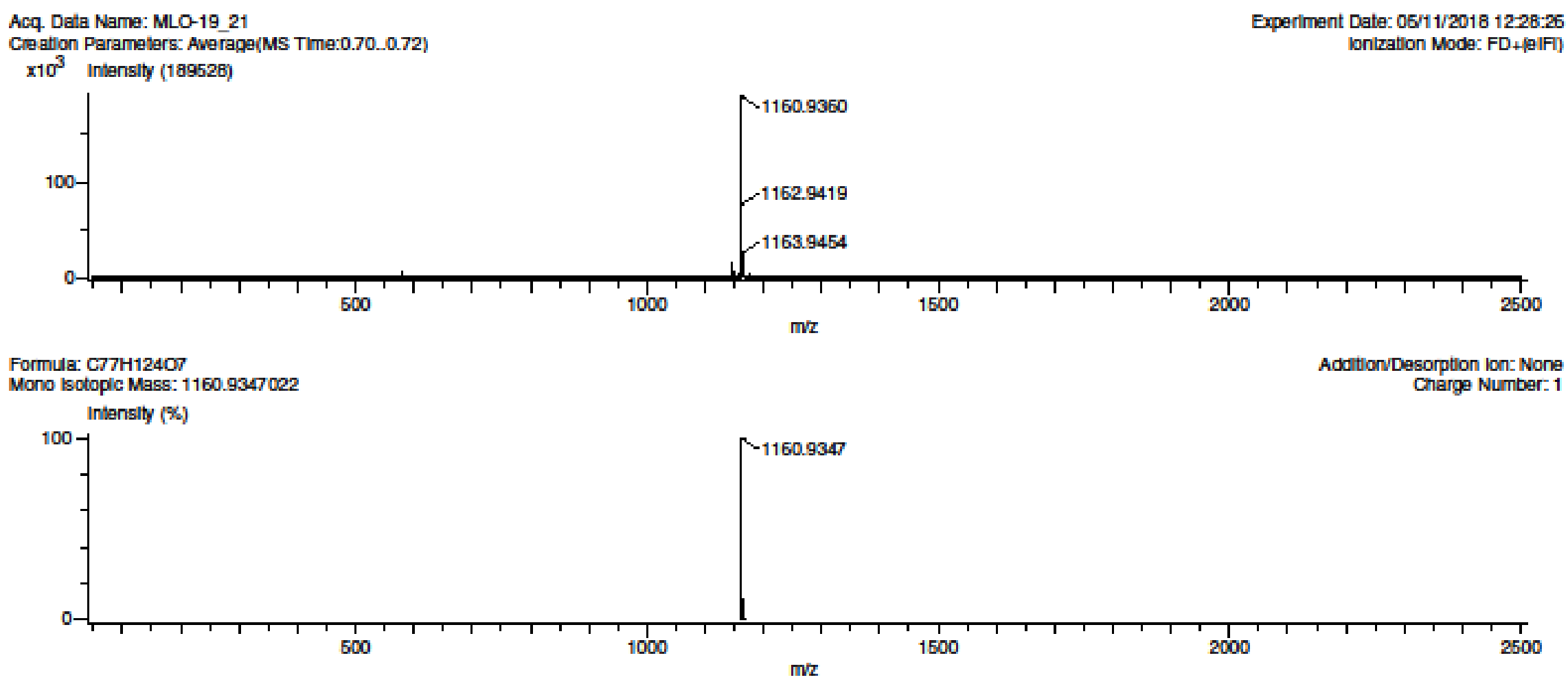

Catenane 16. ^{1}**H-NMR (400 MHz,** $CDCl_3$**)**

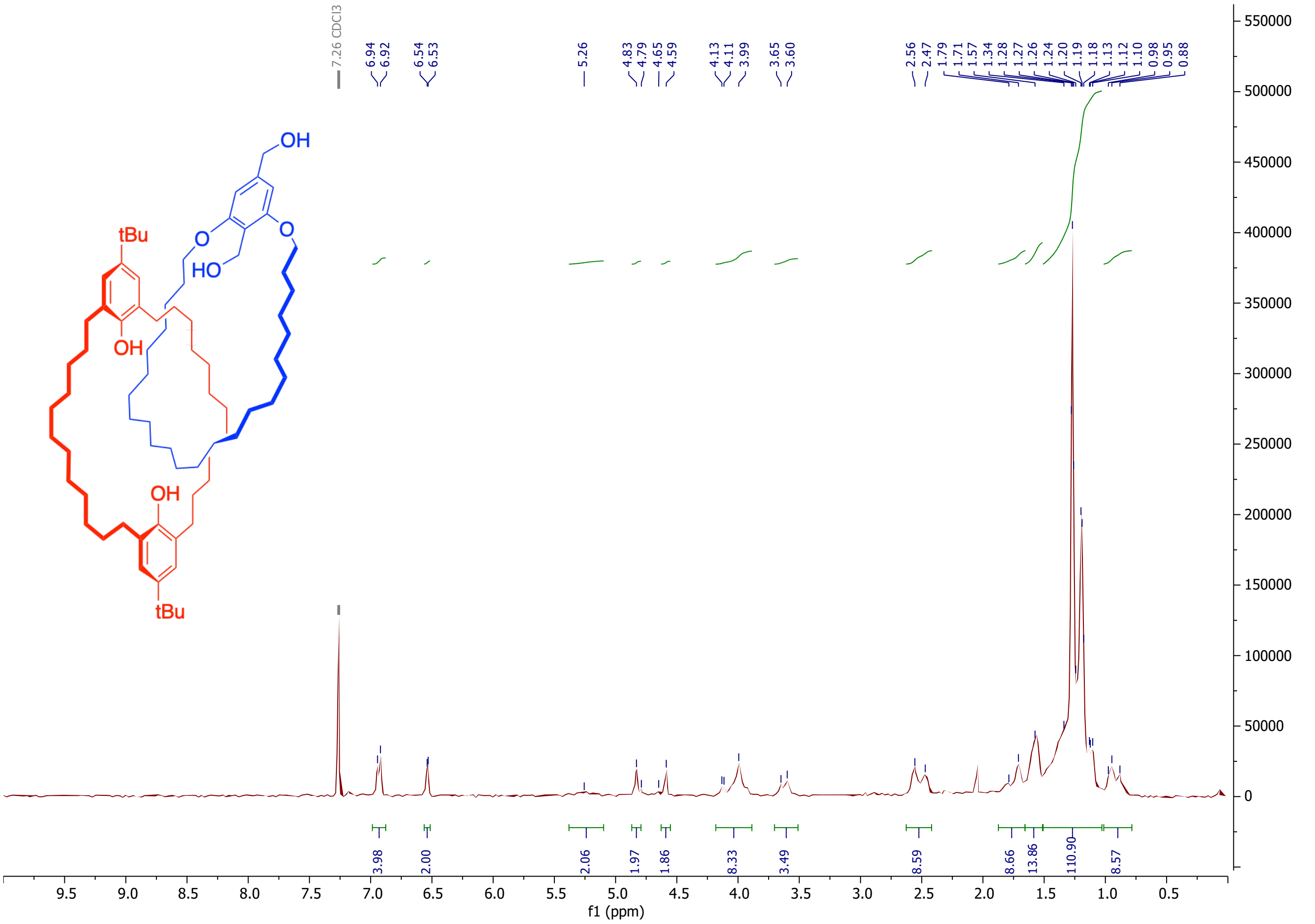

HRMS

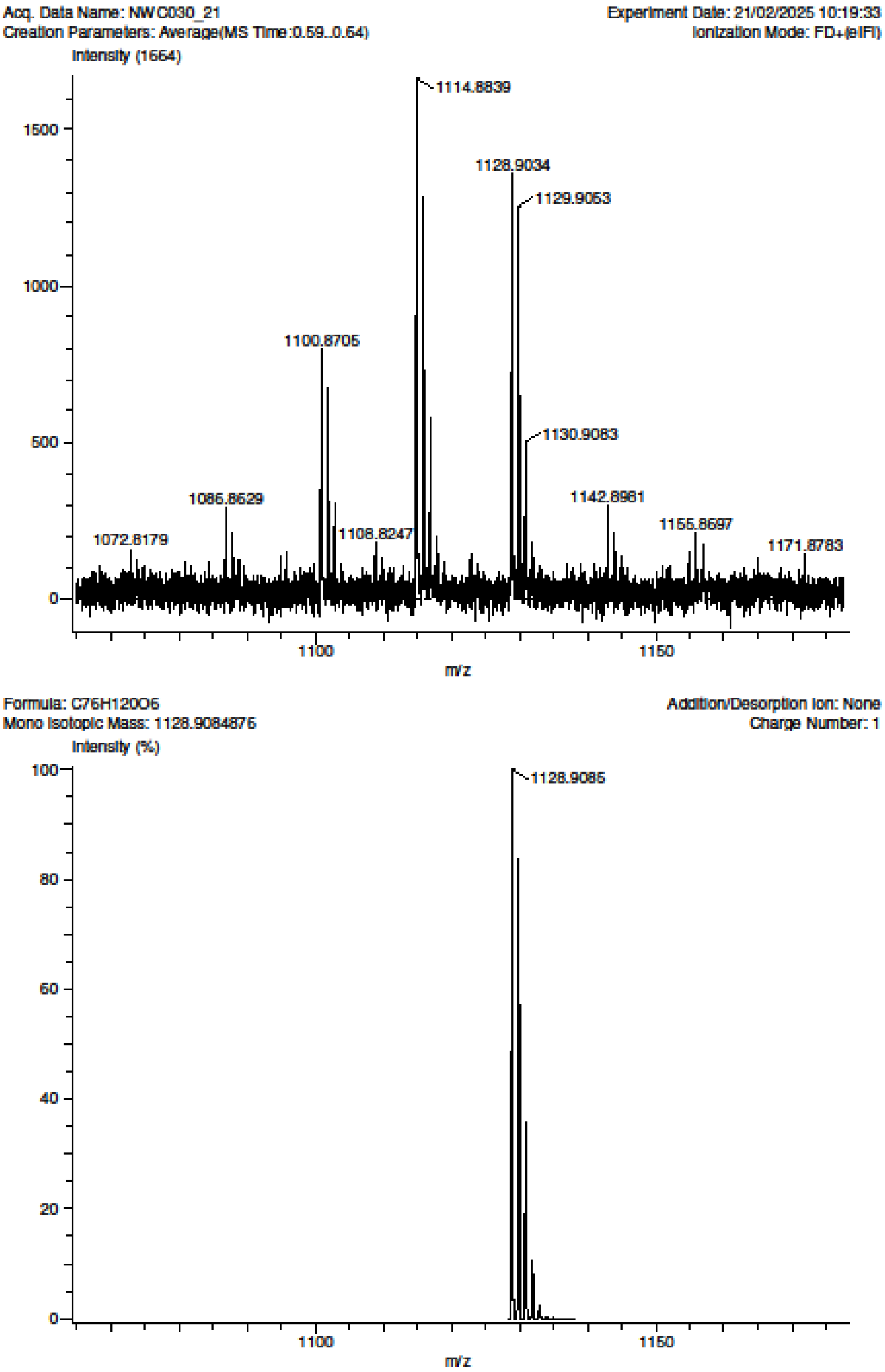

Acq. Data Name: NWC030_21
Creation Parameters: Average(MS Time:0.59..0.64)
Experiment Date: 21/02/2025 10:19:33
Ionization Mode: FD+(eIFI)
Intensity (1654)
1114.8839
1128.9034
1129.9053
1100.8705
1130.9083
1086.8629
1142.8981
1155.8697
1072.8179
1108.8247
1171.8783
m/z
Formula: C76H120O6
Mono Isotopic Mass: 1128.9084876
Addition/Desorption Ion: None
Charge Number: 1
Intensity (%)
1128.9085
m/z

(2-Hydroxy-5-methoxy-1,3-phenylene)dimethanol (18). ^{1}**H-NMR (400 MHz, DMSO-d$_6$)**

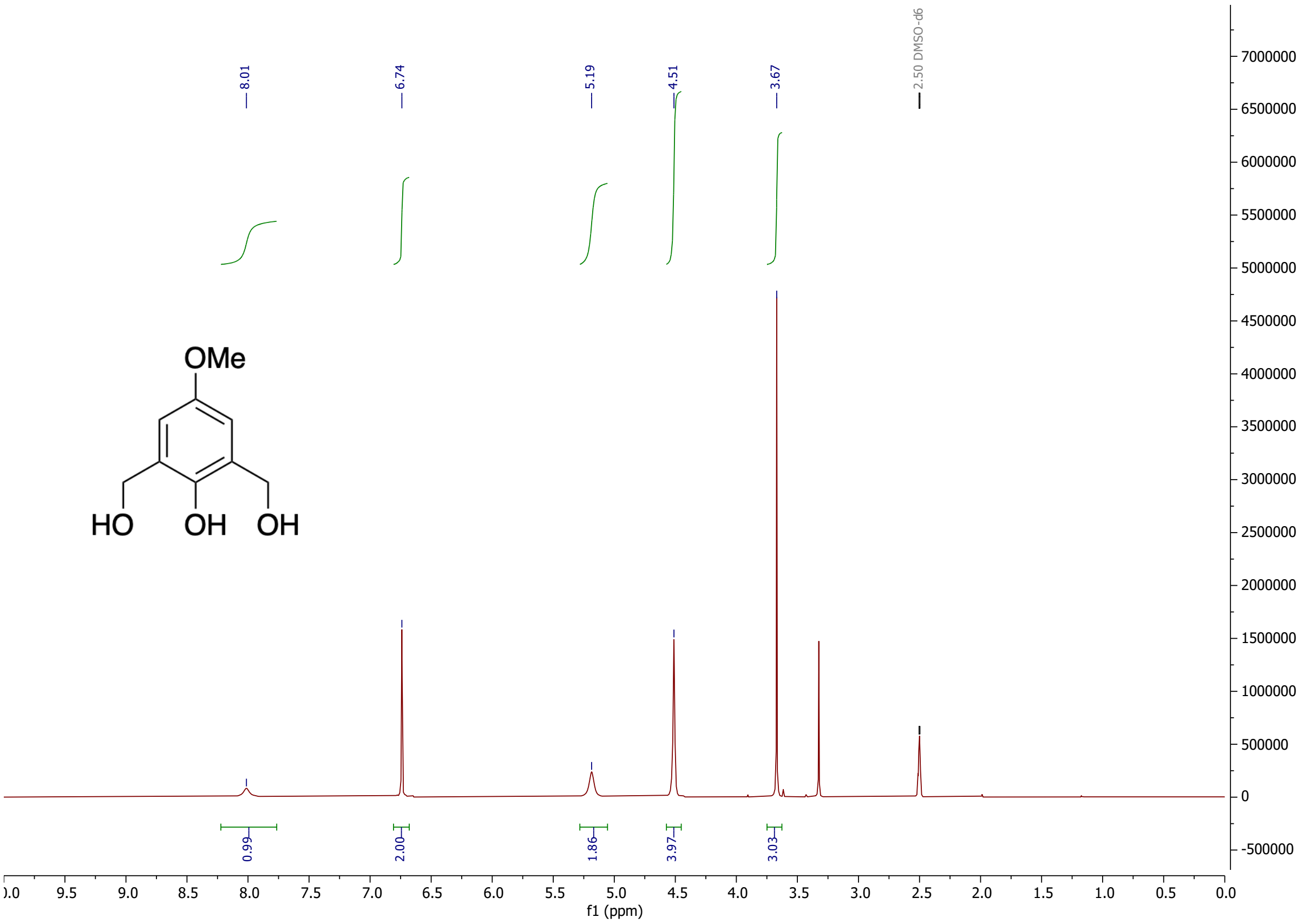

2-Hydroxy-5-methoxyisophthalaldehyde (19). ^{1}**H-NMR (400 MHz, CDCl$_3$)**

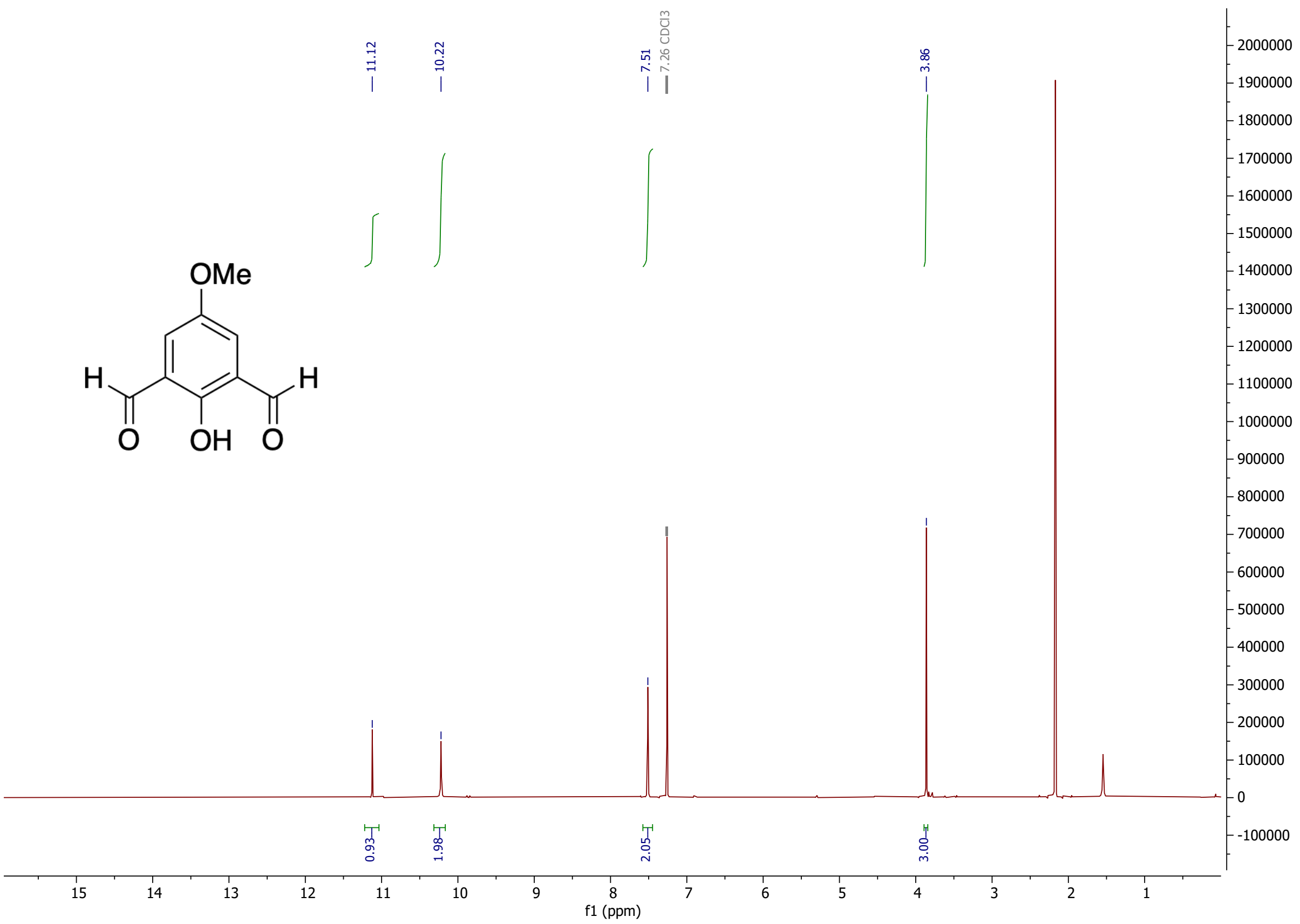

1,1'-(2-Hydroxy-5-methoxy-1,3-phenylene)bis(hept-6-en-1-ol) (20). ^{1}H-NMR (400 MHz, $CDCl_3$)

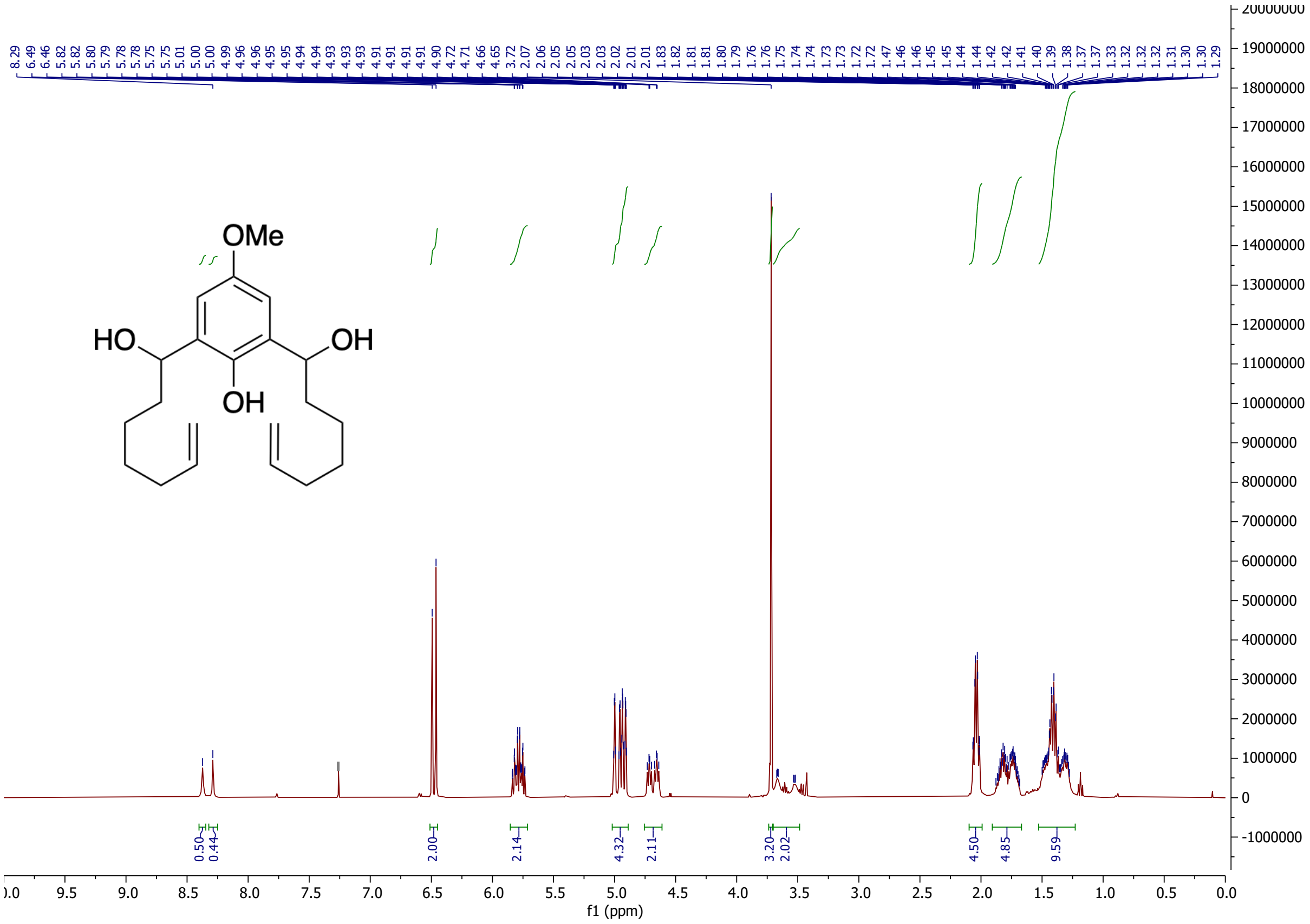

^{13}C{^{1}H}-NMR (101 MHz, $CDCl_3$)

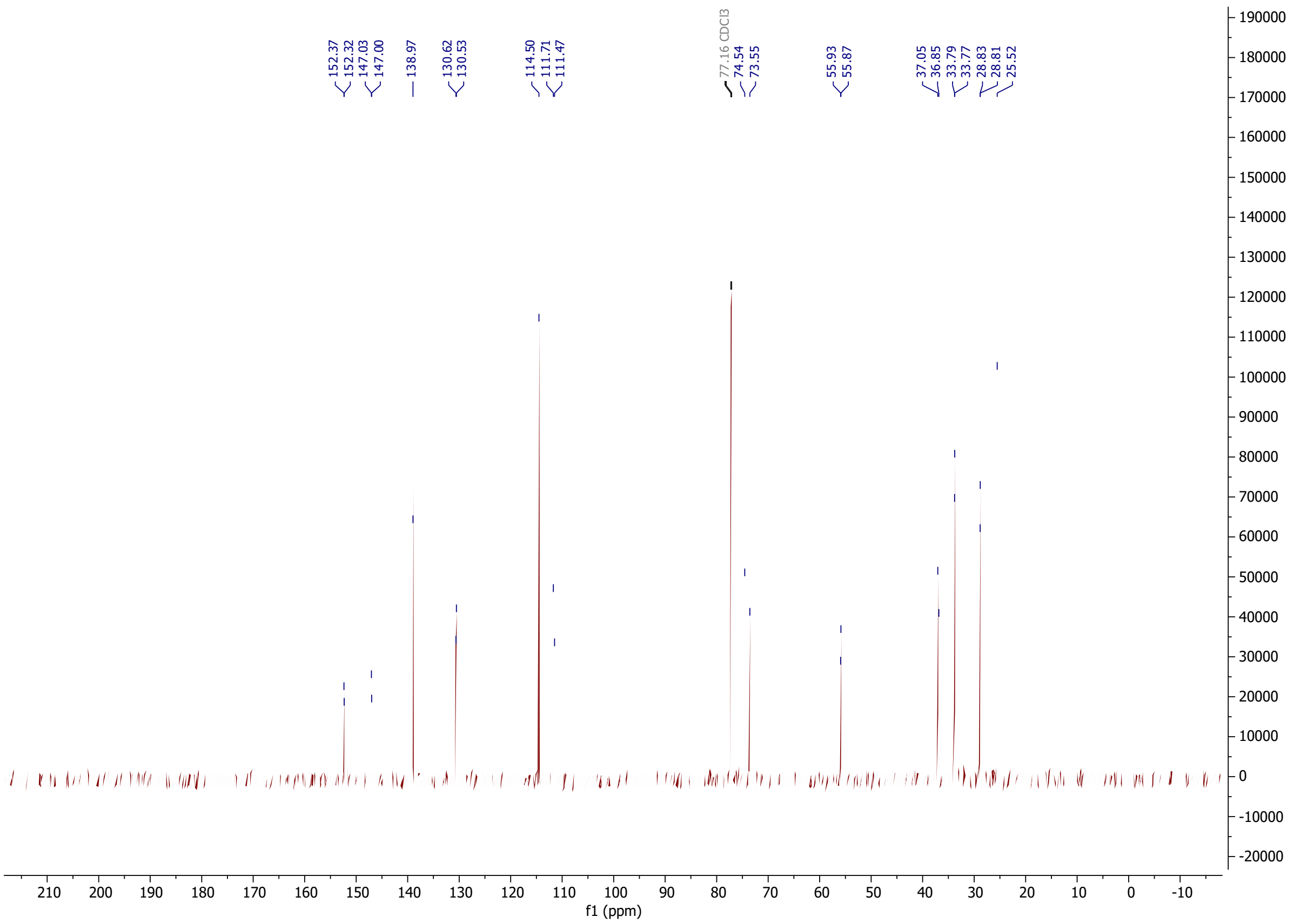

HRMS

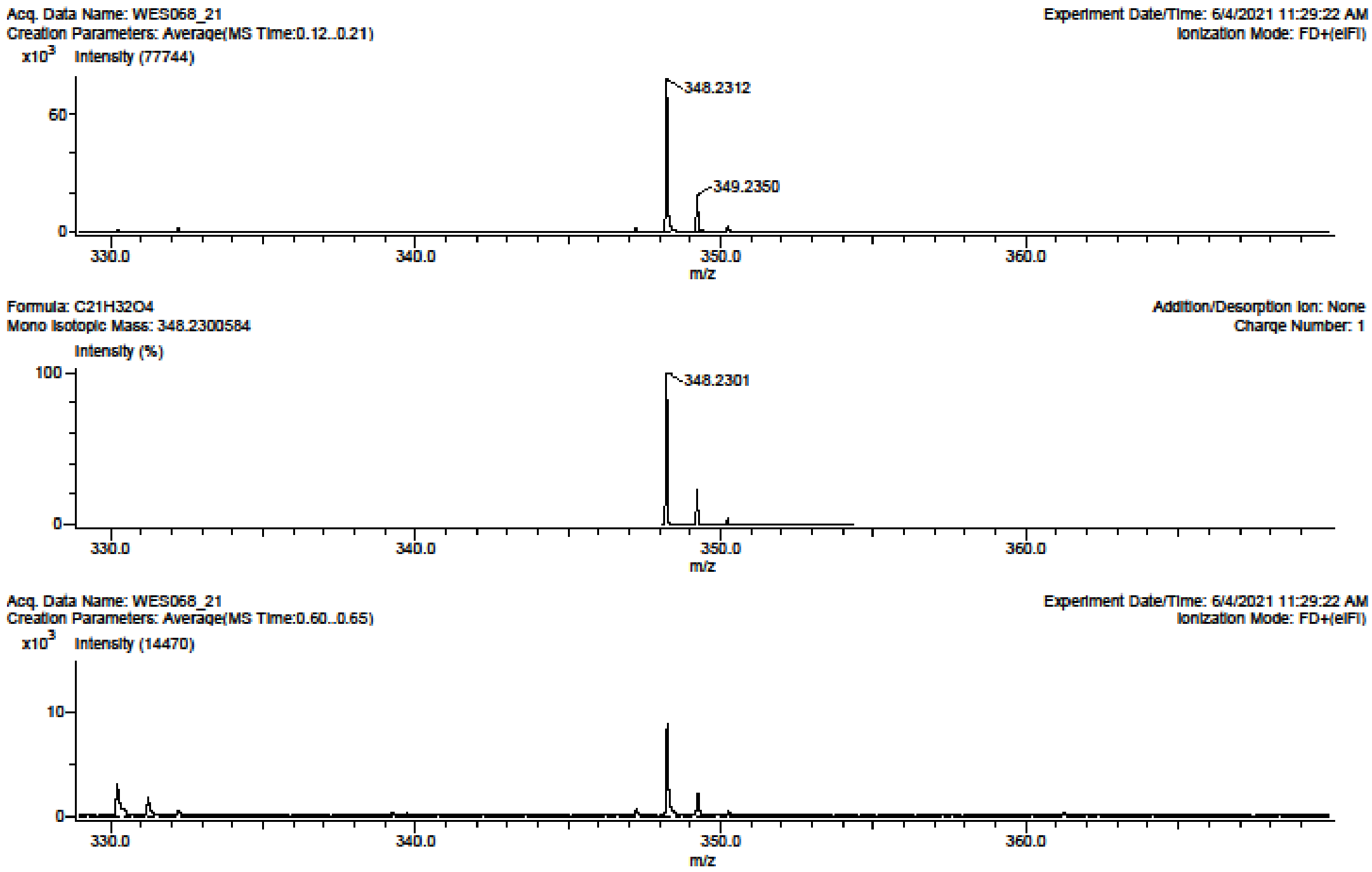

2,6-di(hept-6-en-1-yl)-4-methoxyphenol (21). ^{1}H-NMR (400 MHz, $CDCl_3$)

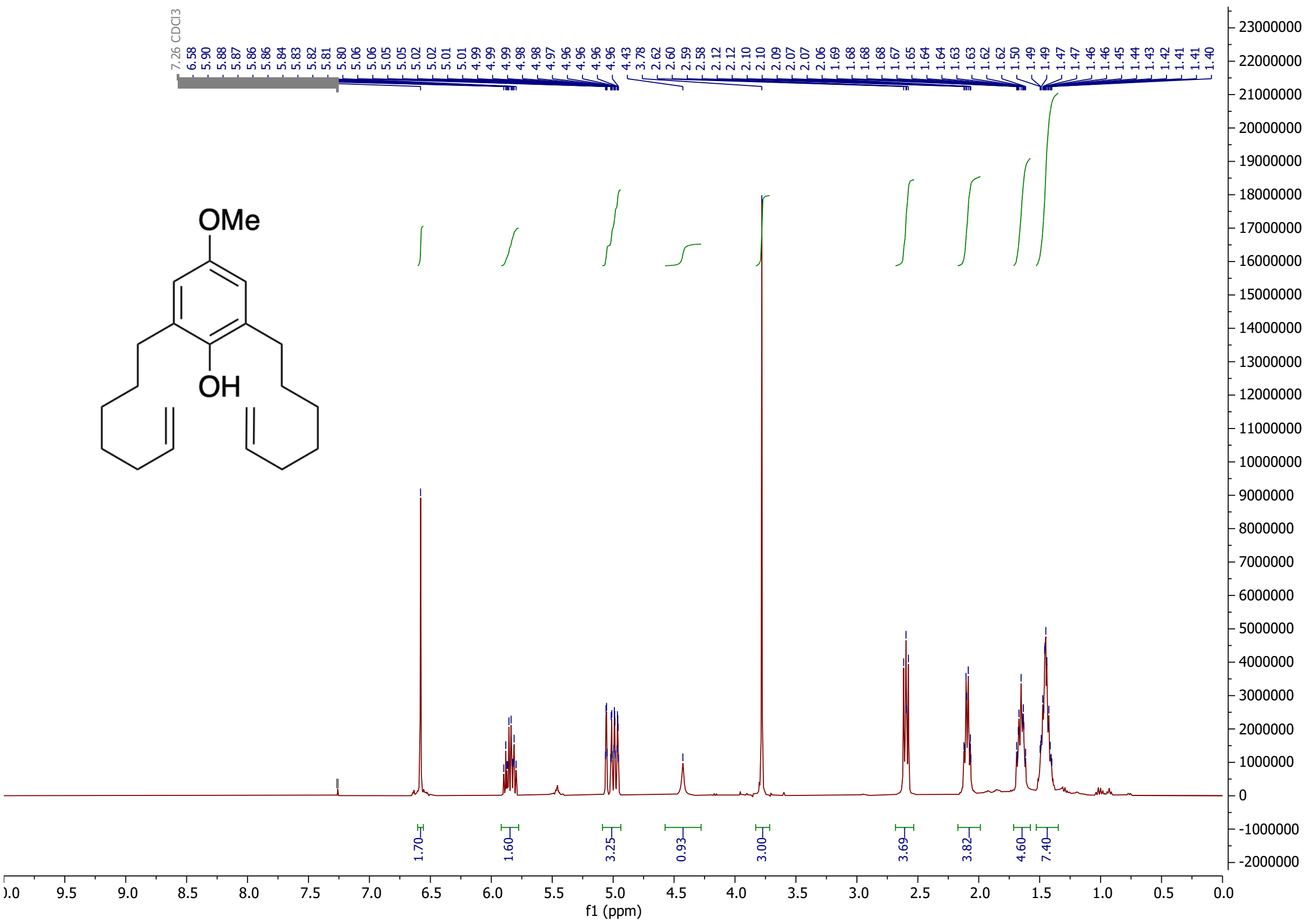

$^{13}C\{^{1}H\}$-NMR (101 MHz, $CDCl_3$)

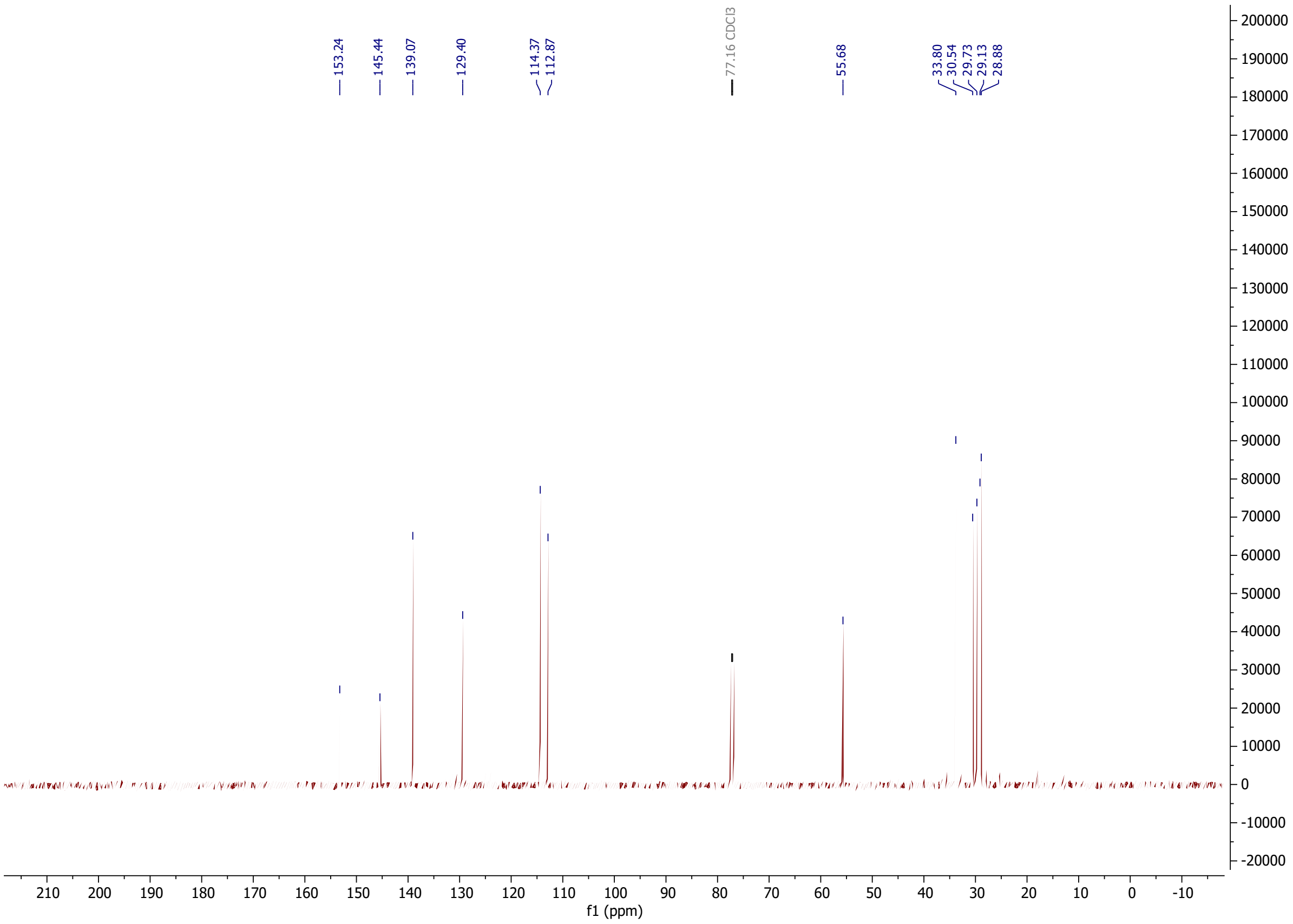

HRMS

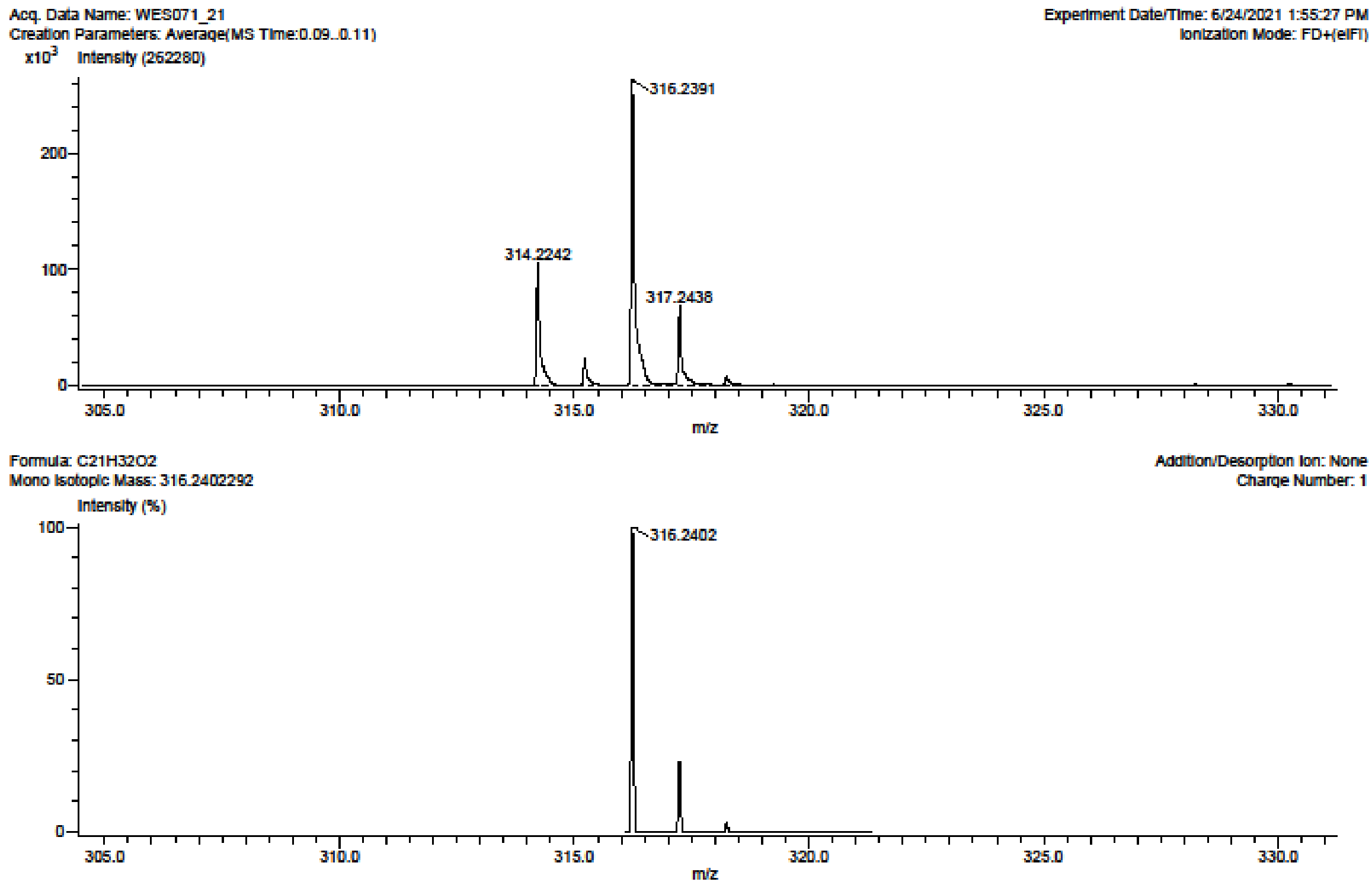

Compound 22a. ^{1}H-NMR (400 MHz, $CDCl_3$)

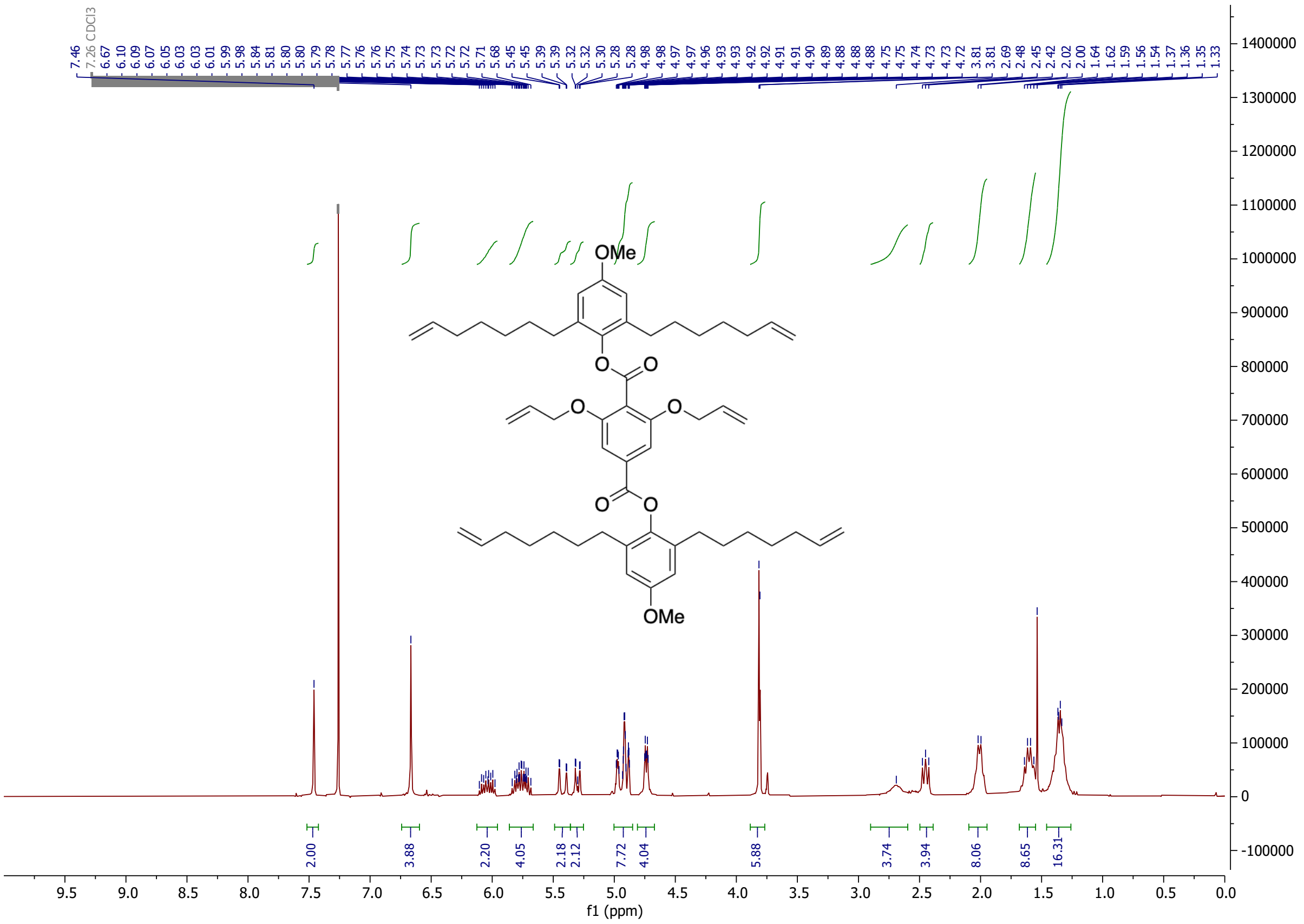

^{13}C{^{1}H}-NMR (101 MHz, $CDCl_3$)

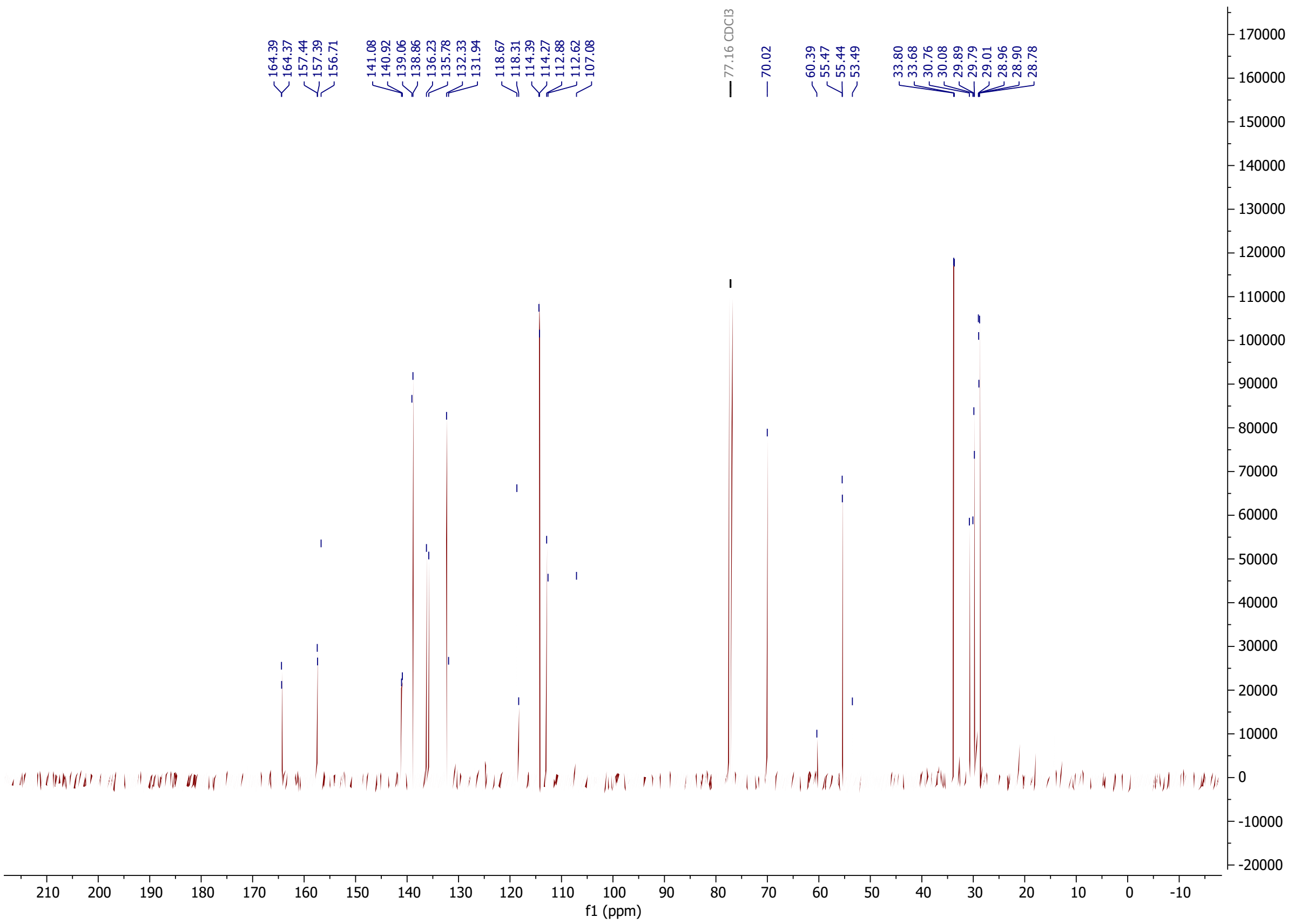

HRMS

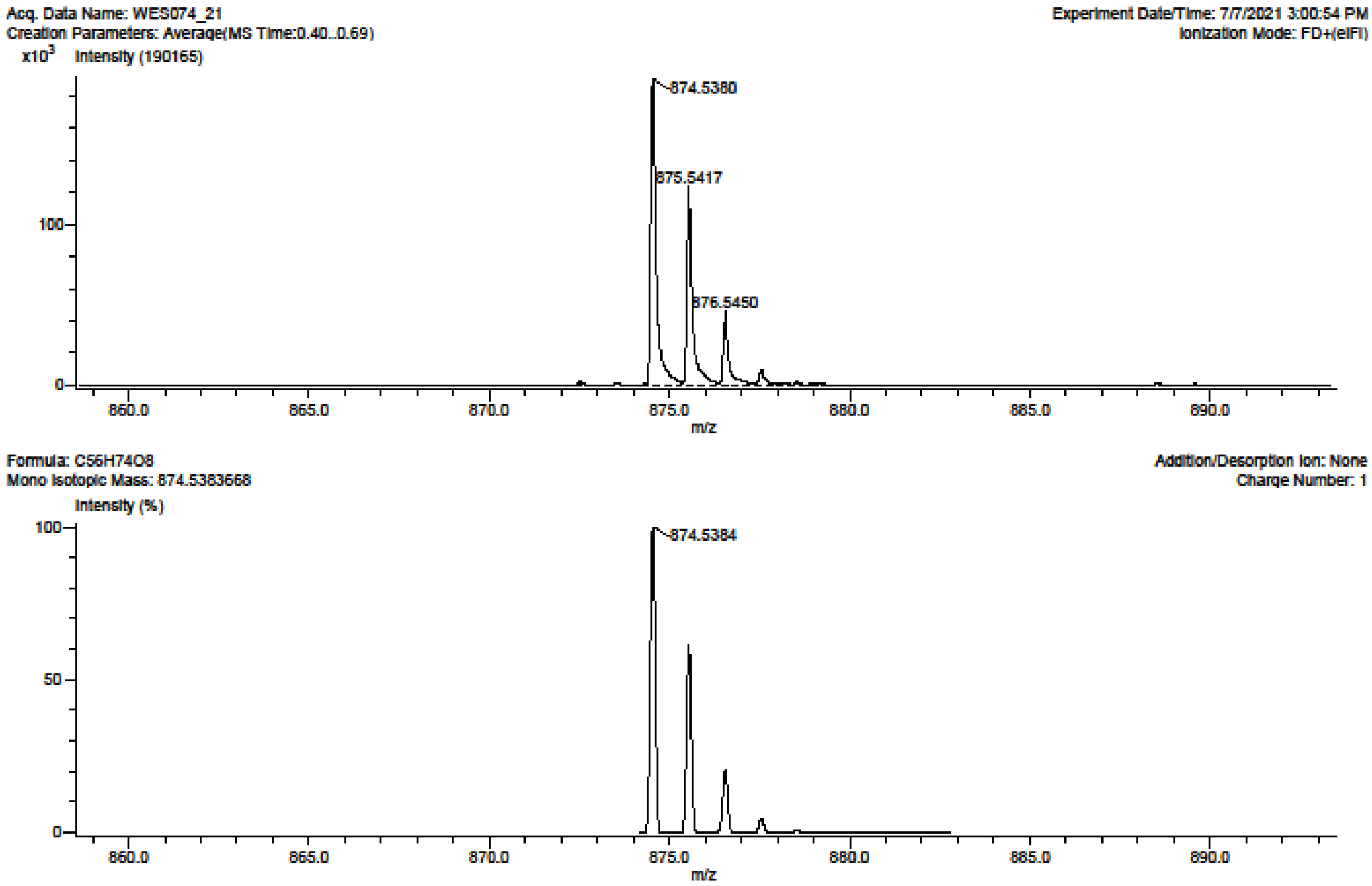

Compound 22b. ^{1}H-NMR (400 MHz, $CDCl_3$)

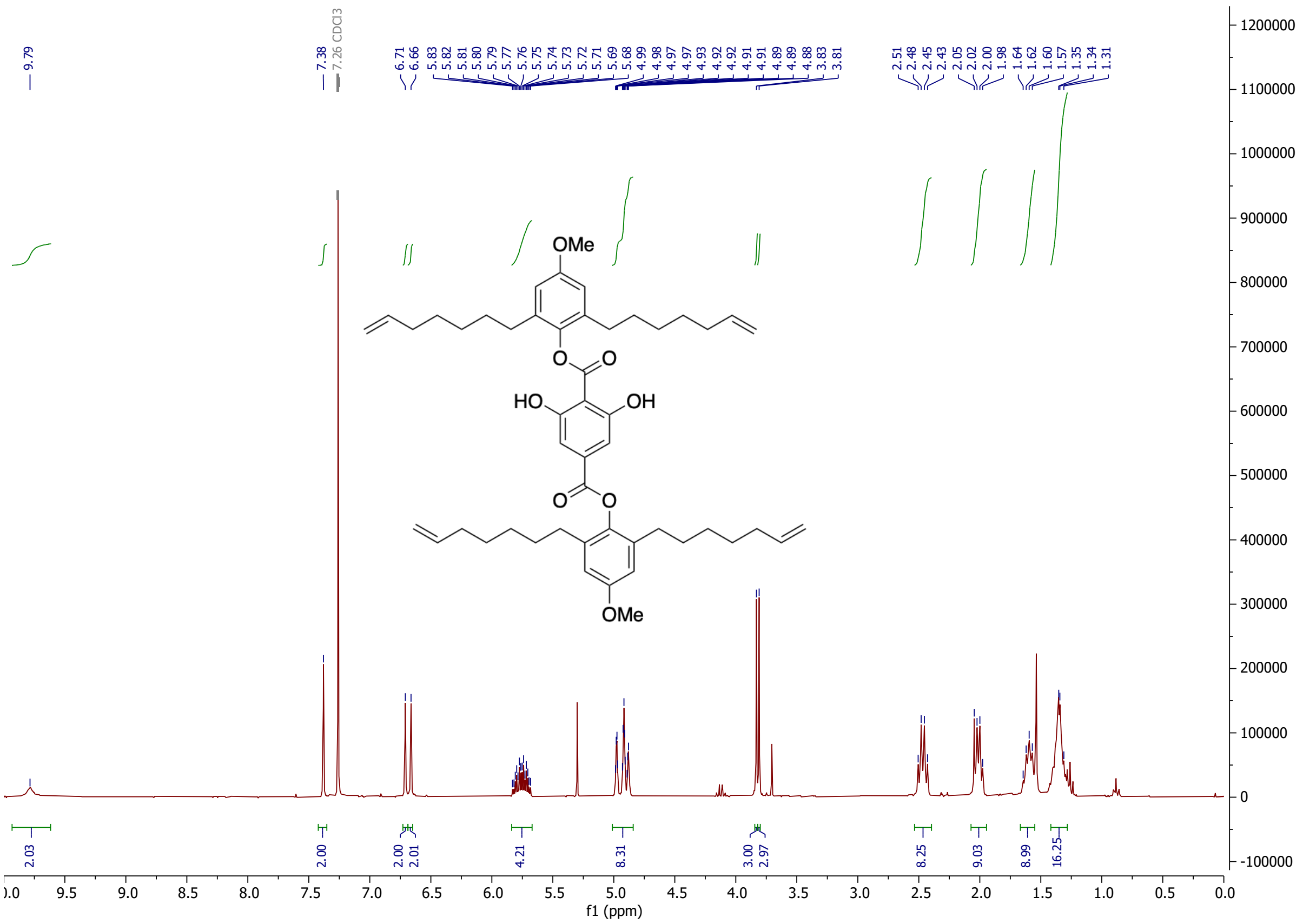

$^{13}C\{^1H\}$-NMR (101 MHz, $CDCl_3$)

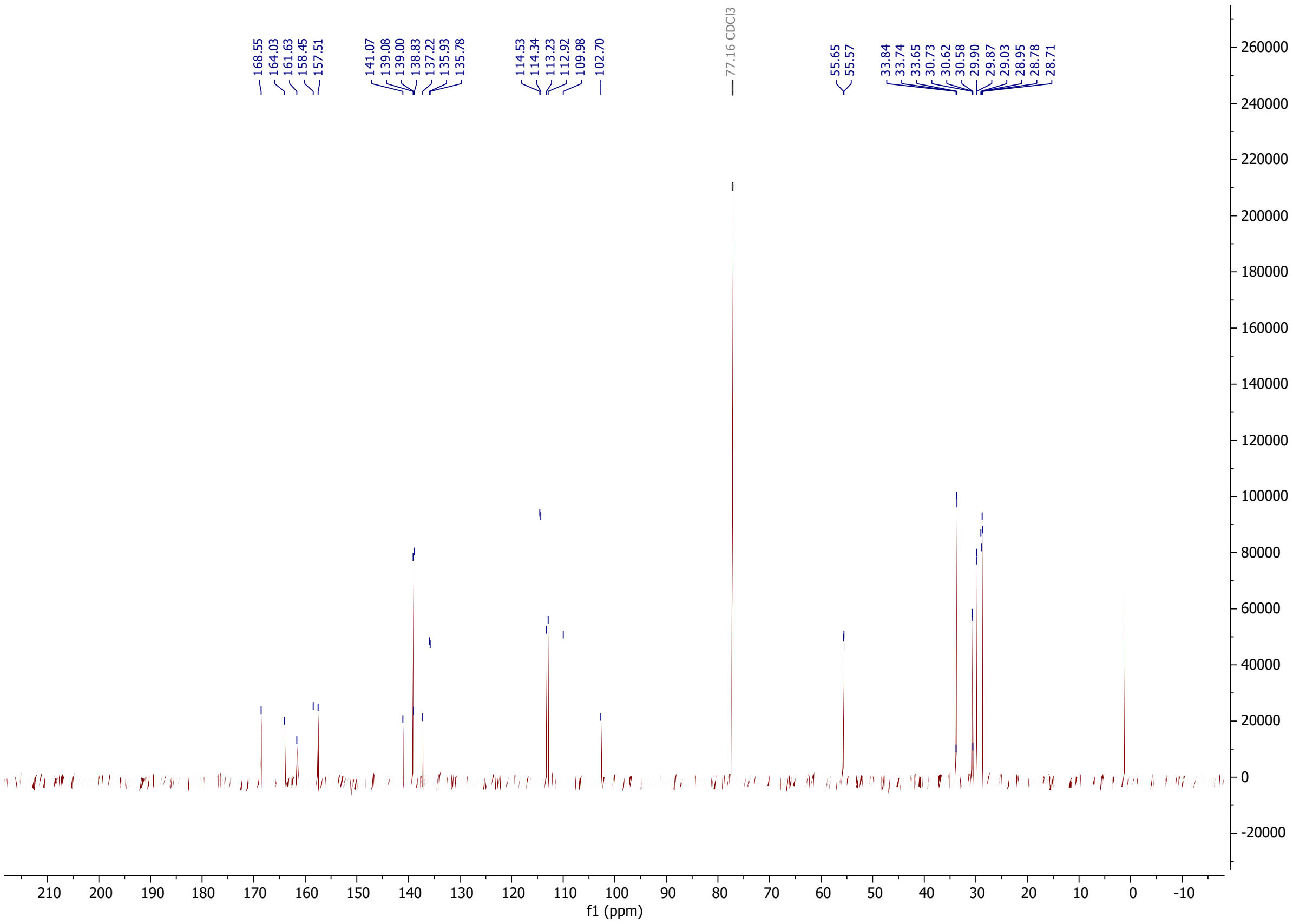

HRMS

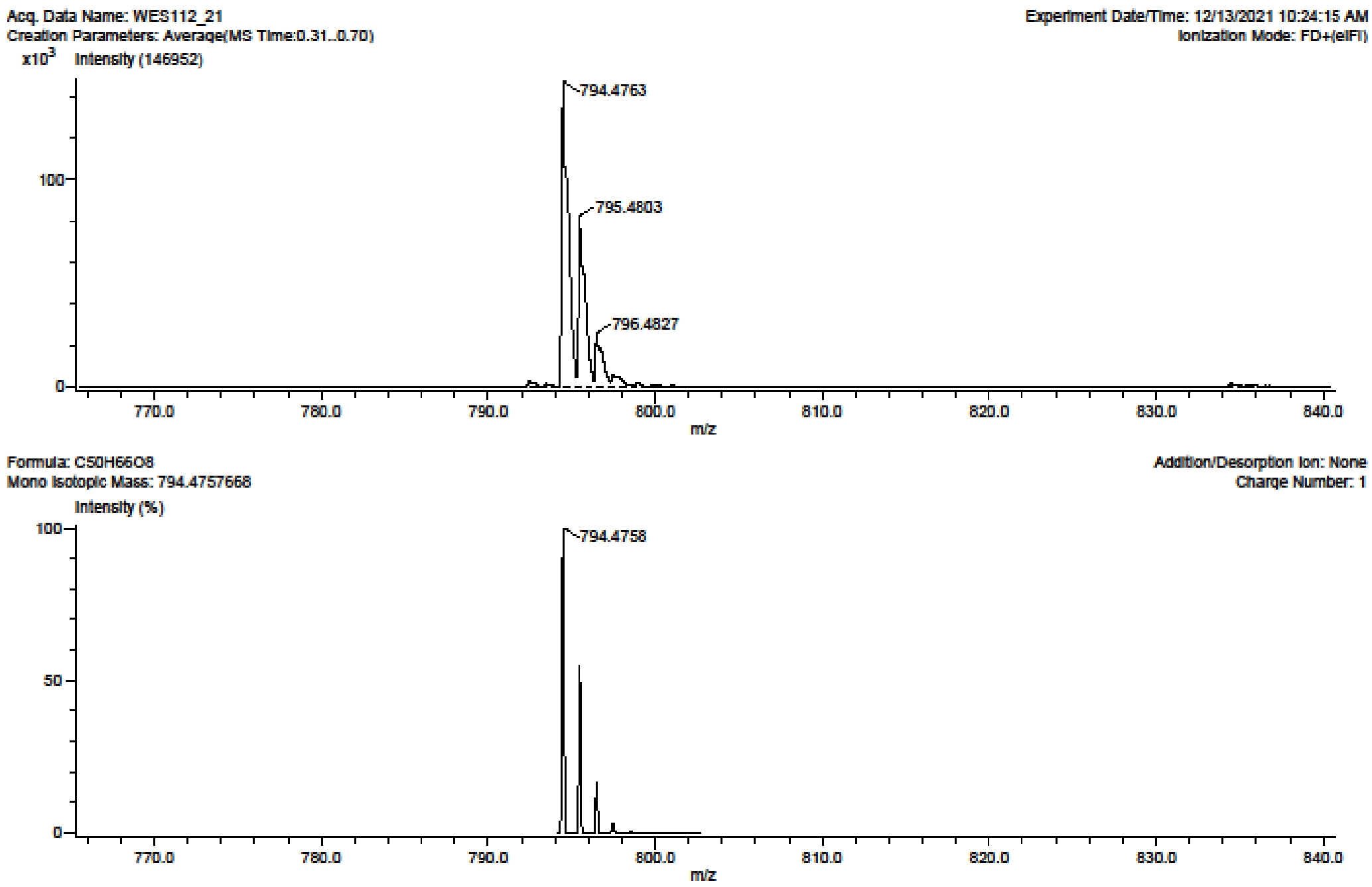

Precatenane precursor 23a. ¹H-NMR (400 MHz, $CDCl_3$)

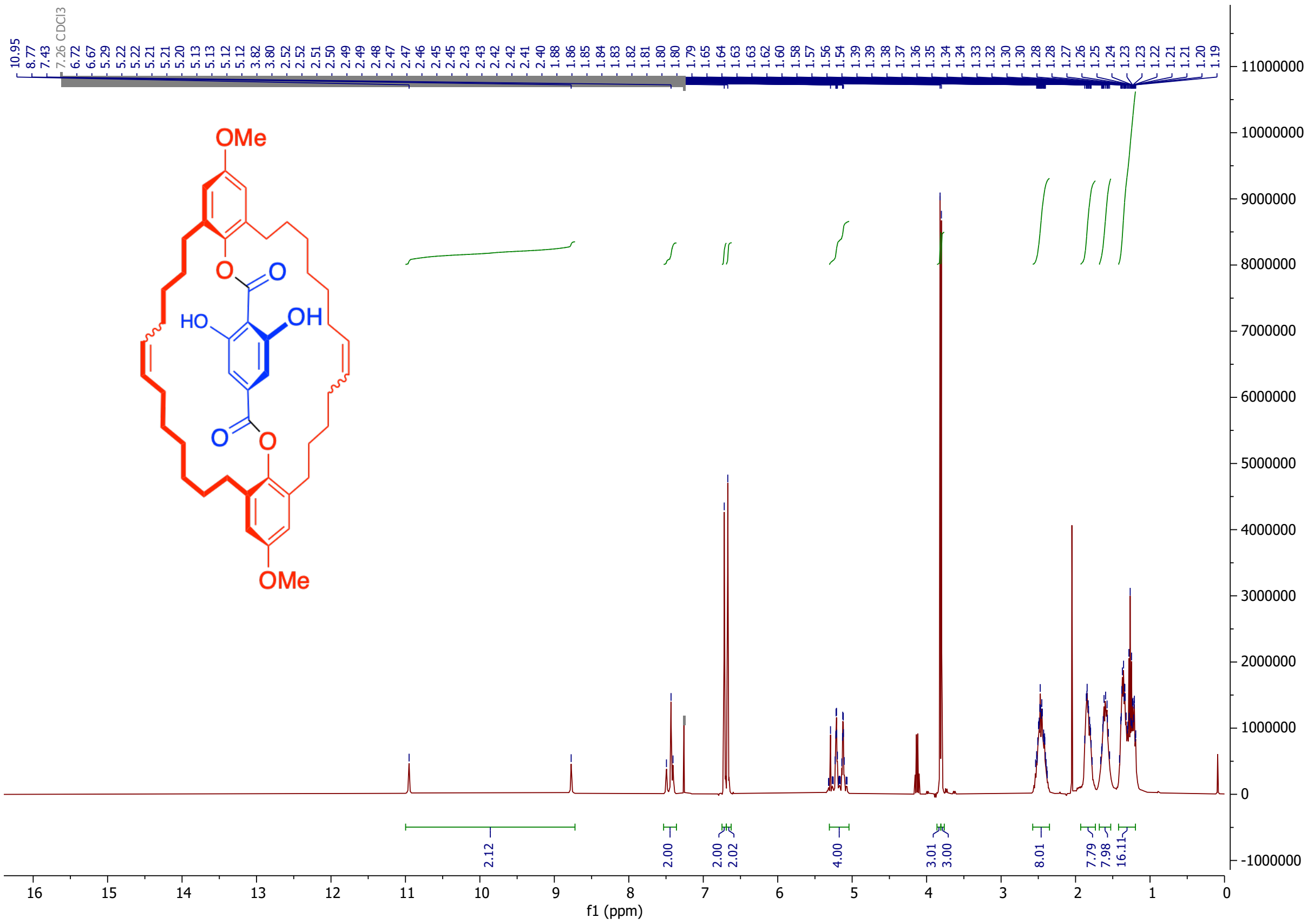

¹³C{¹H}-NMR (101 MHz, $CDCl_3$)

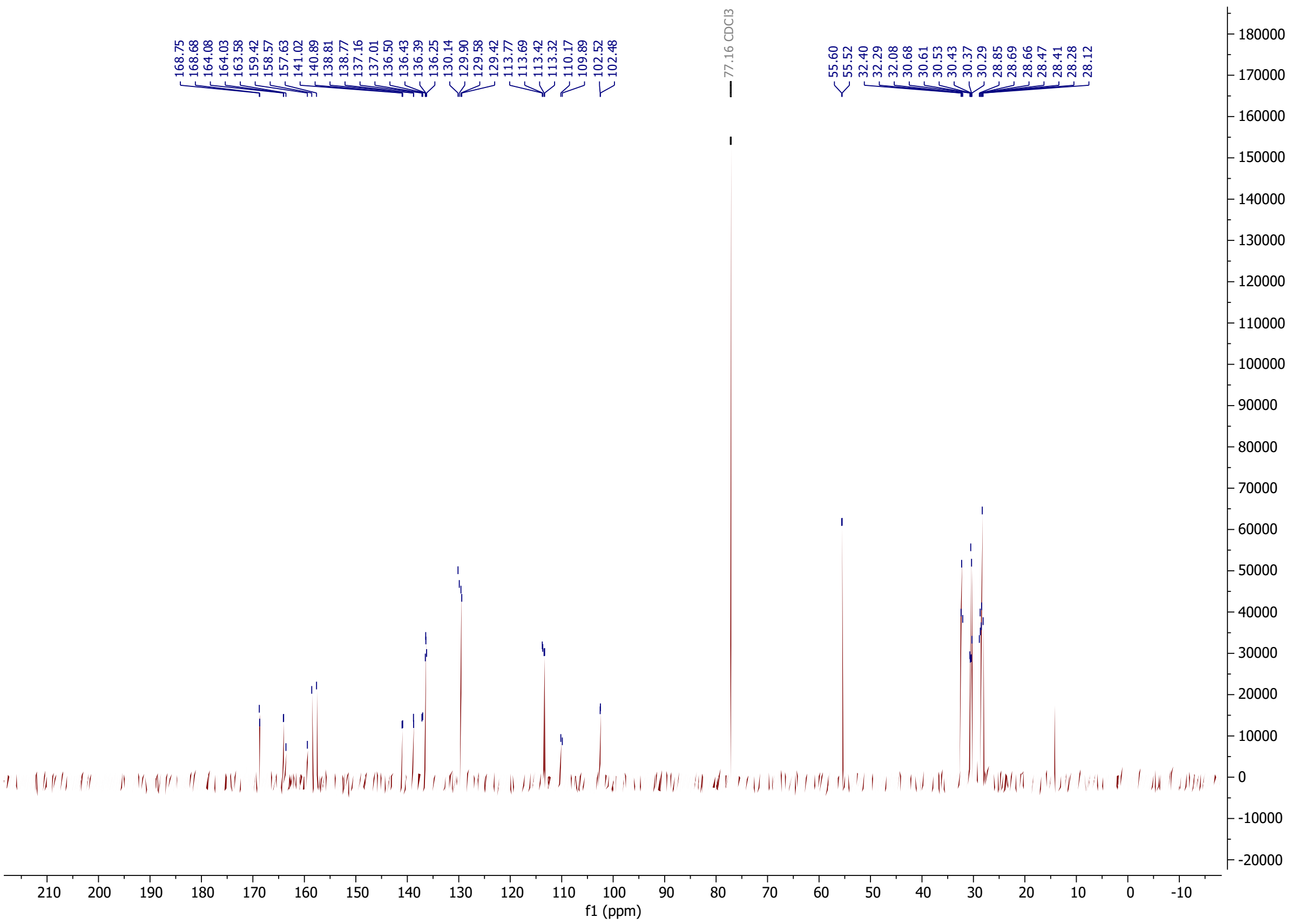

HRMS

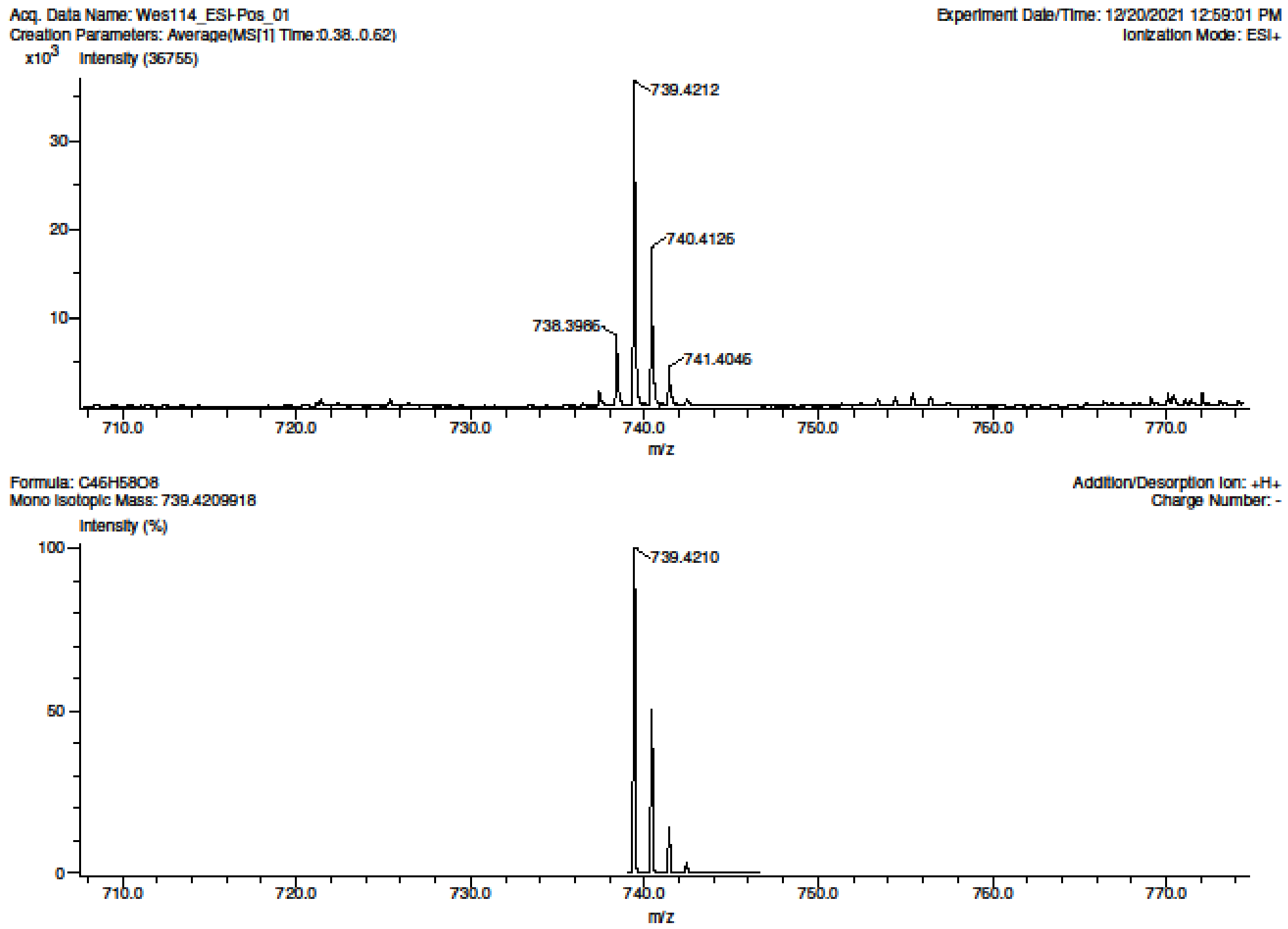

Prerotaxane precursor 23b. ^{1}H-NMR (400 MHz, $CDCl_3$)

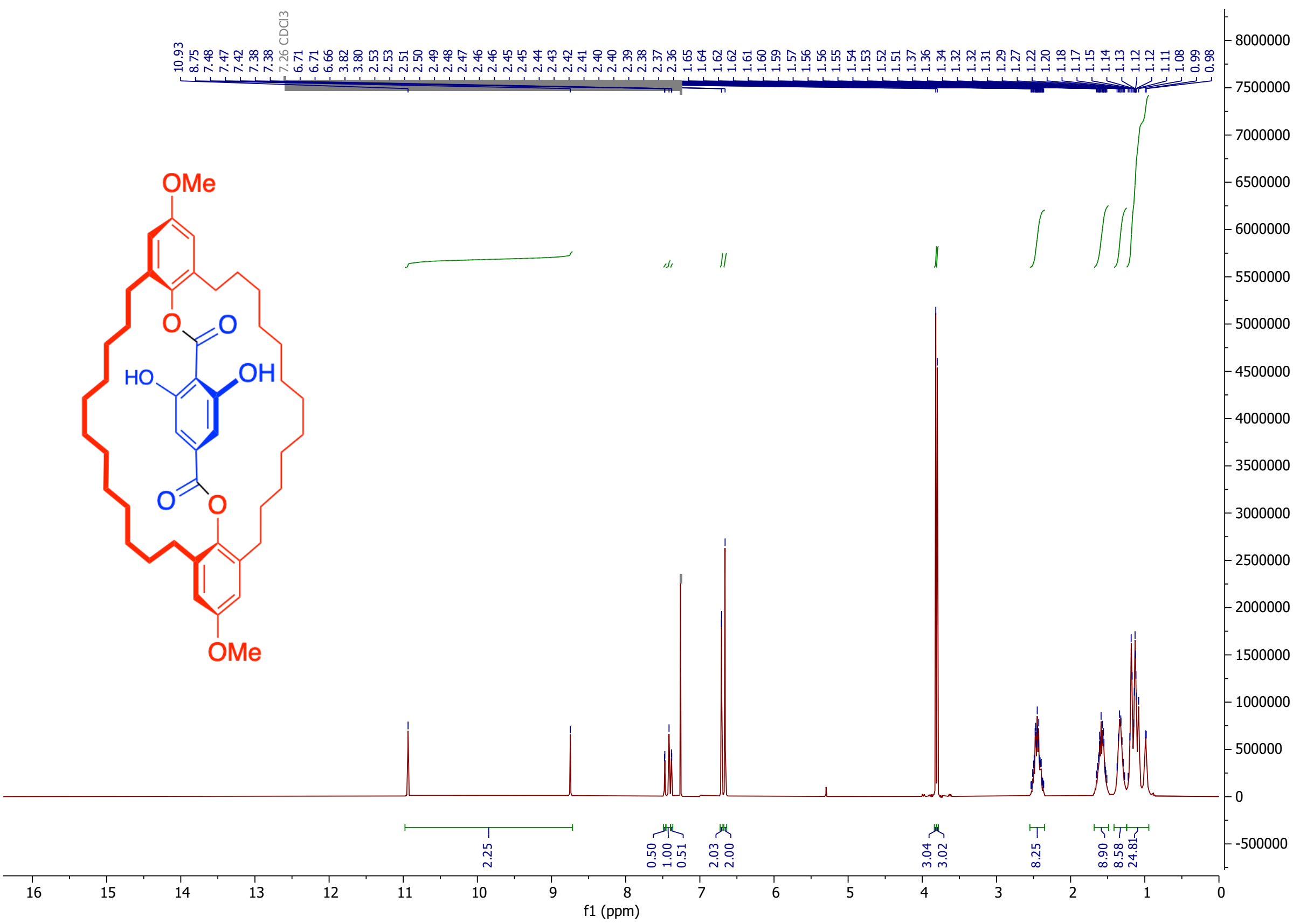

$^{13}C\{^{1}H\}$-NMR (101 MHz, $CDCl_3$)

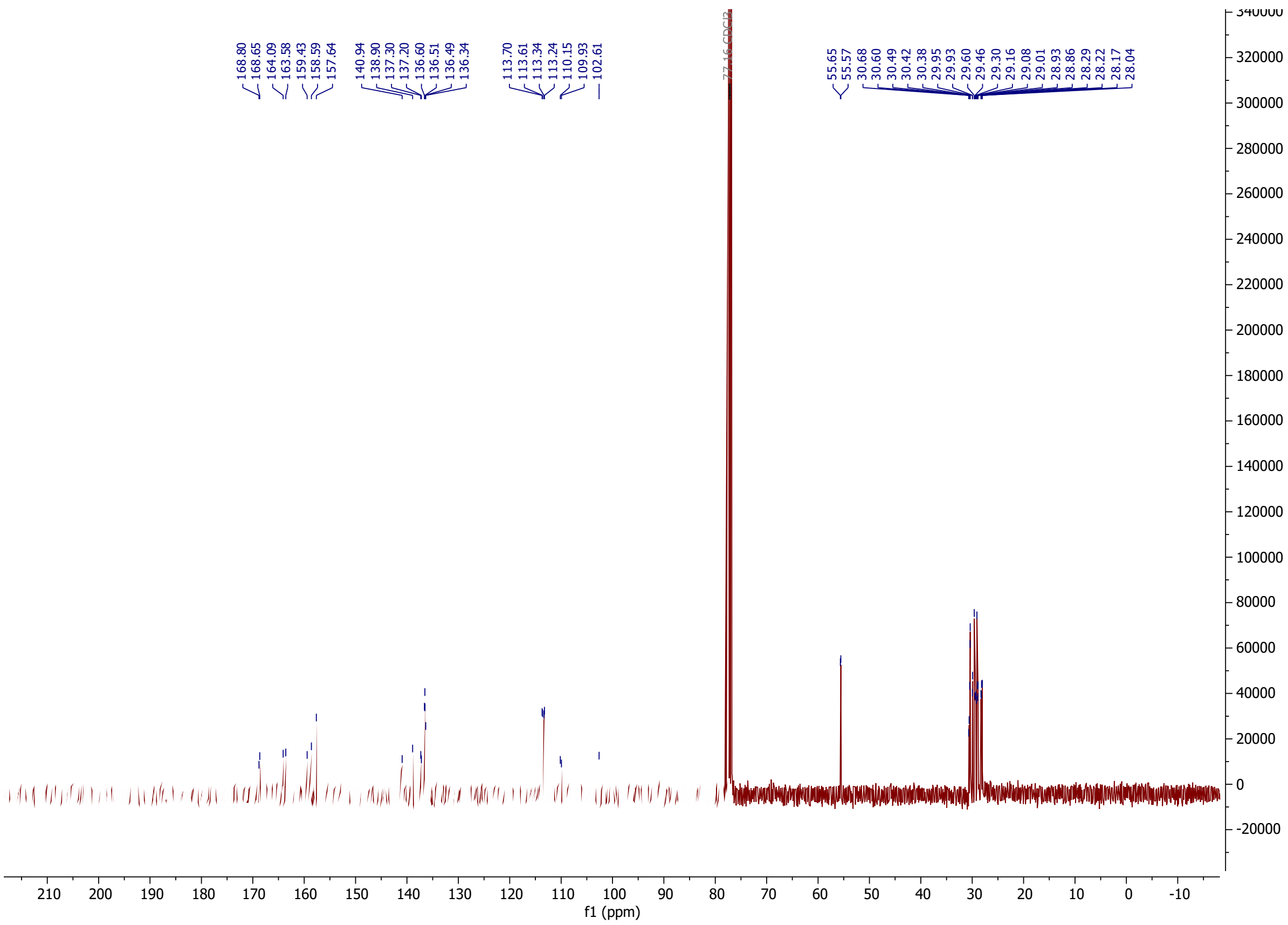

HRMS

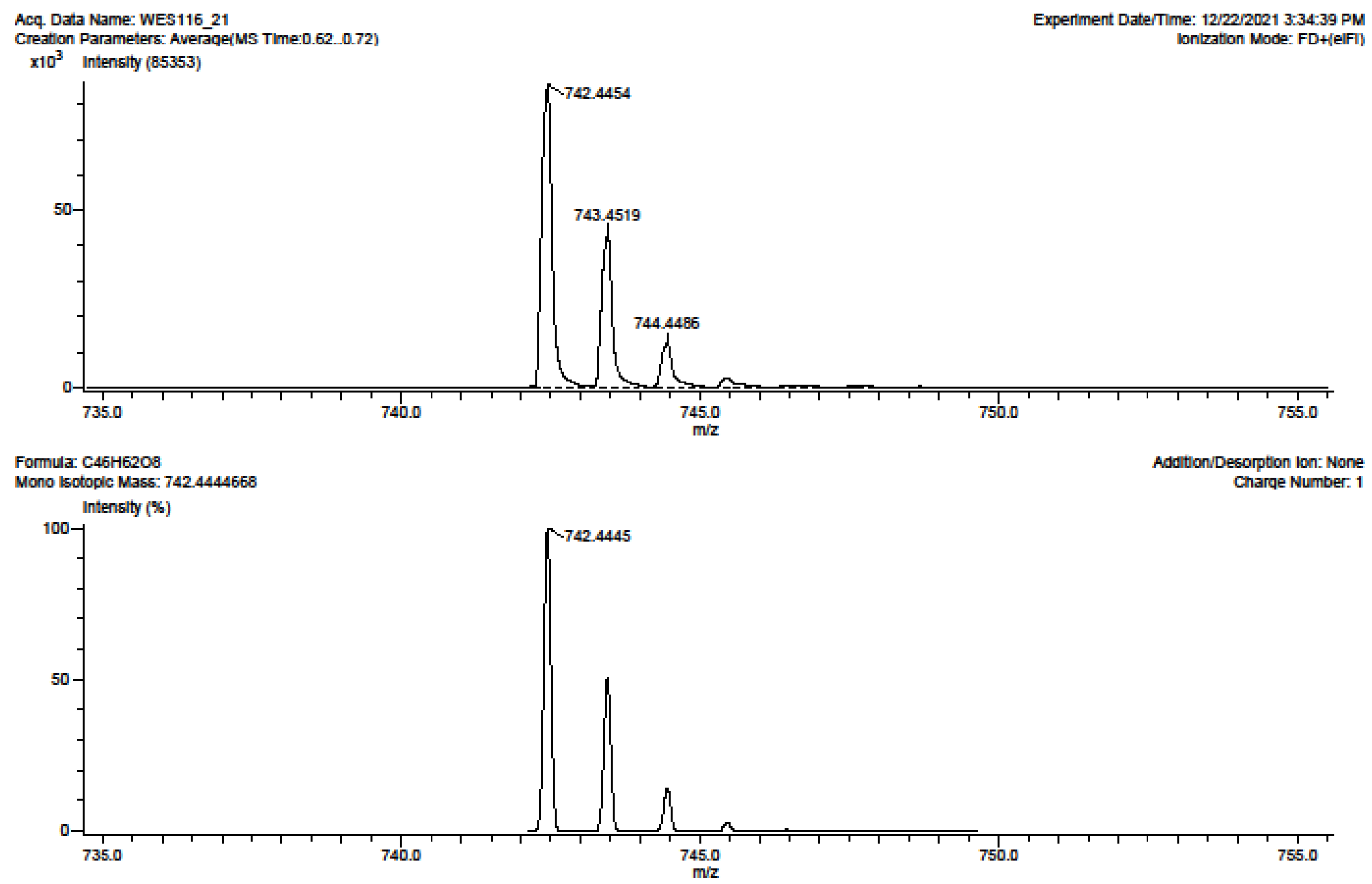

Precatenane precursor 24. ¹H-NMR (400 MHz, CDCl₃)

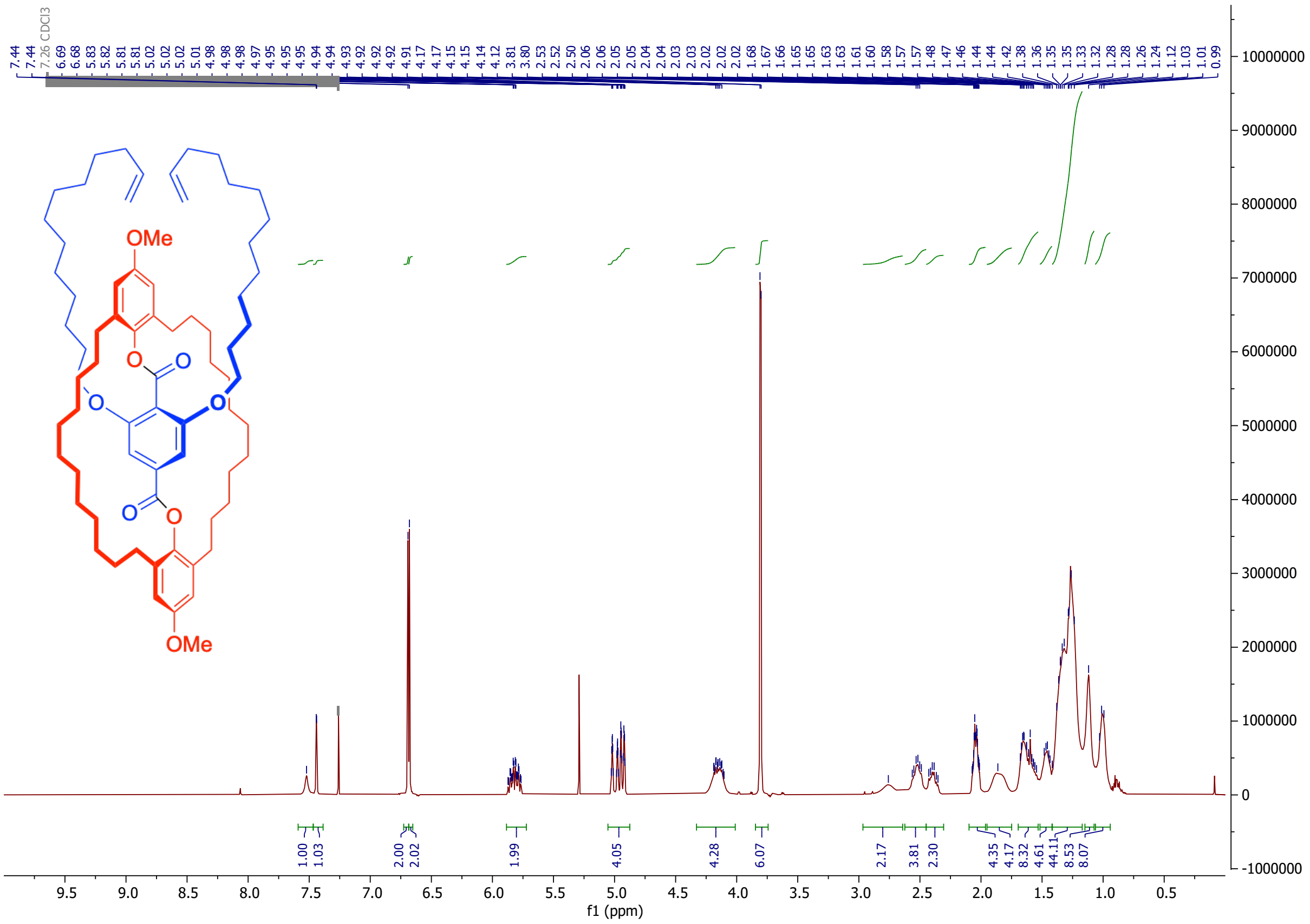

¹³C{¹H}-NMR (101 MHz, CDCl₃)

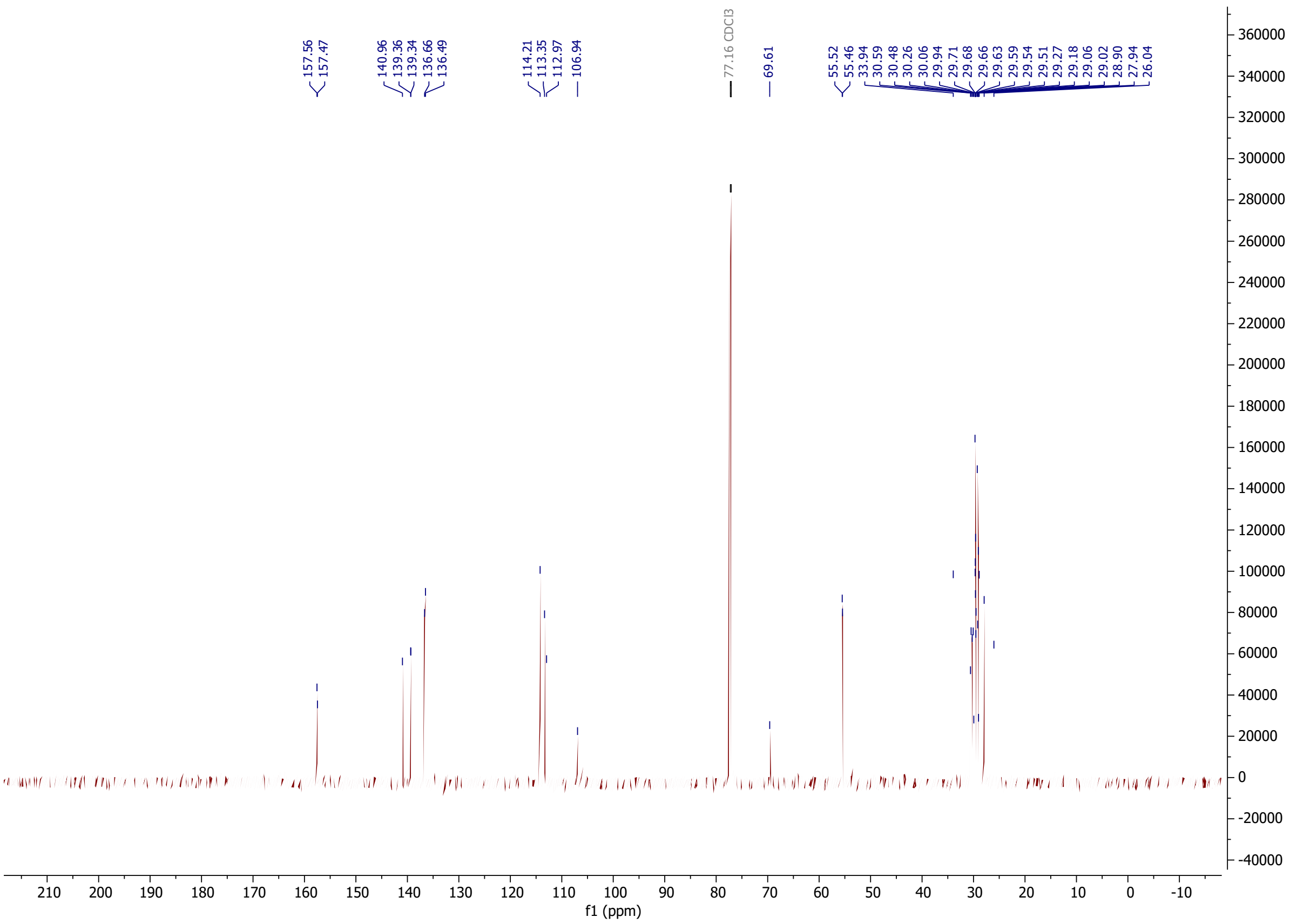

HRMS

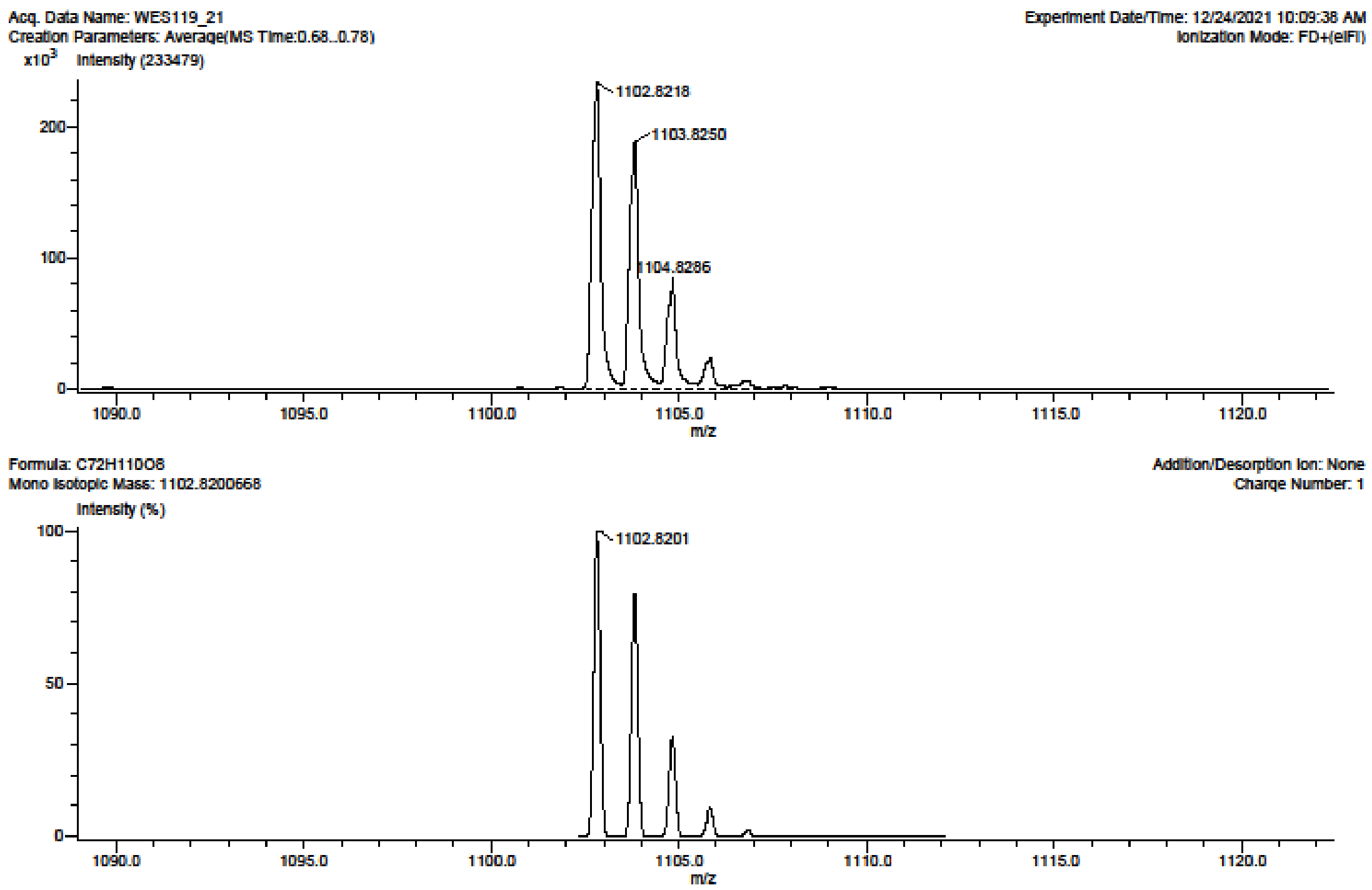

Precatenane 25a. ^{1}H-NMR (400 MHz, $CDCl_3$)

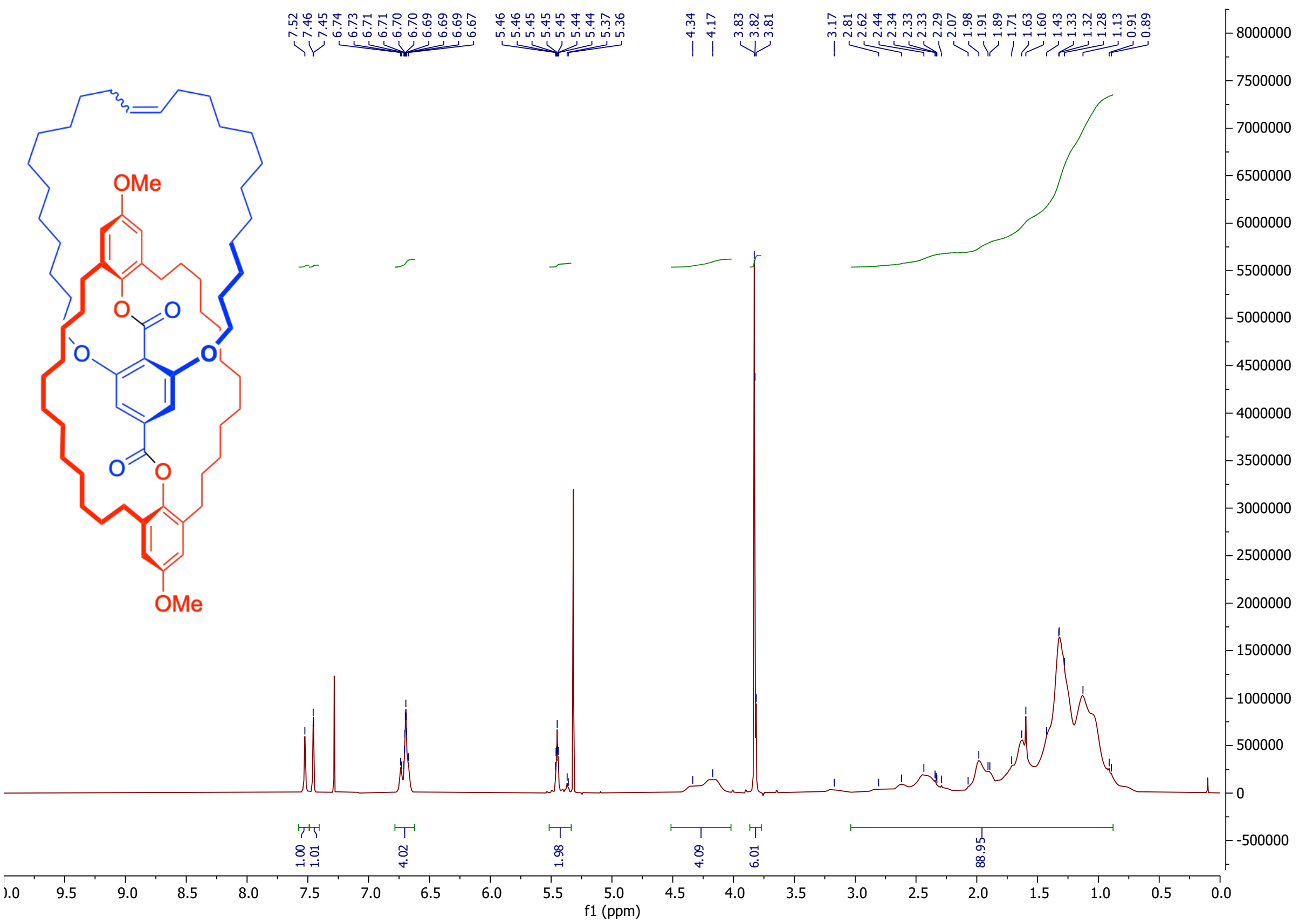

$^{13}C\{^1H\}$-NMR (101 MHz, $CDCl_3$)

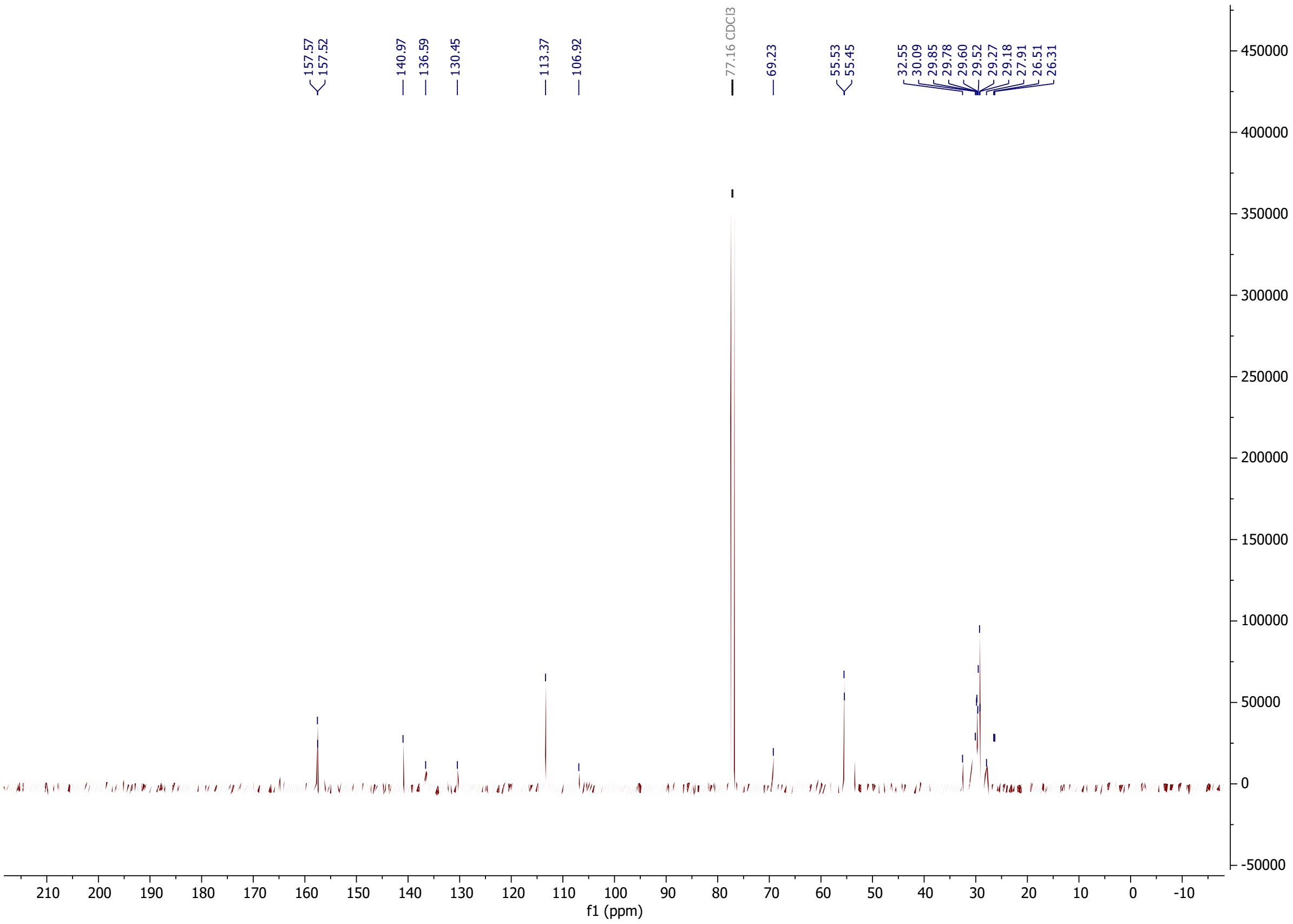

HRMS

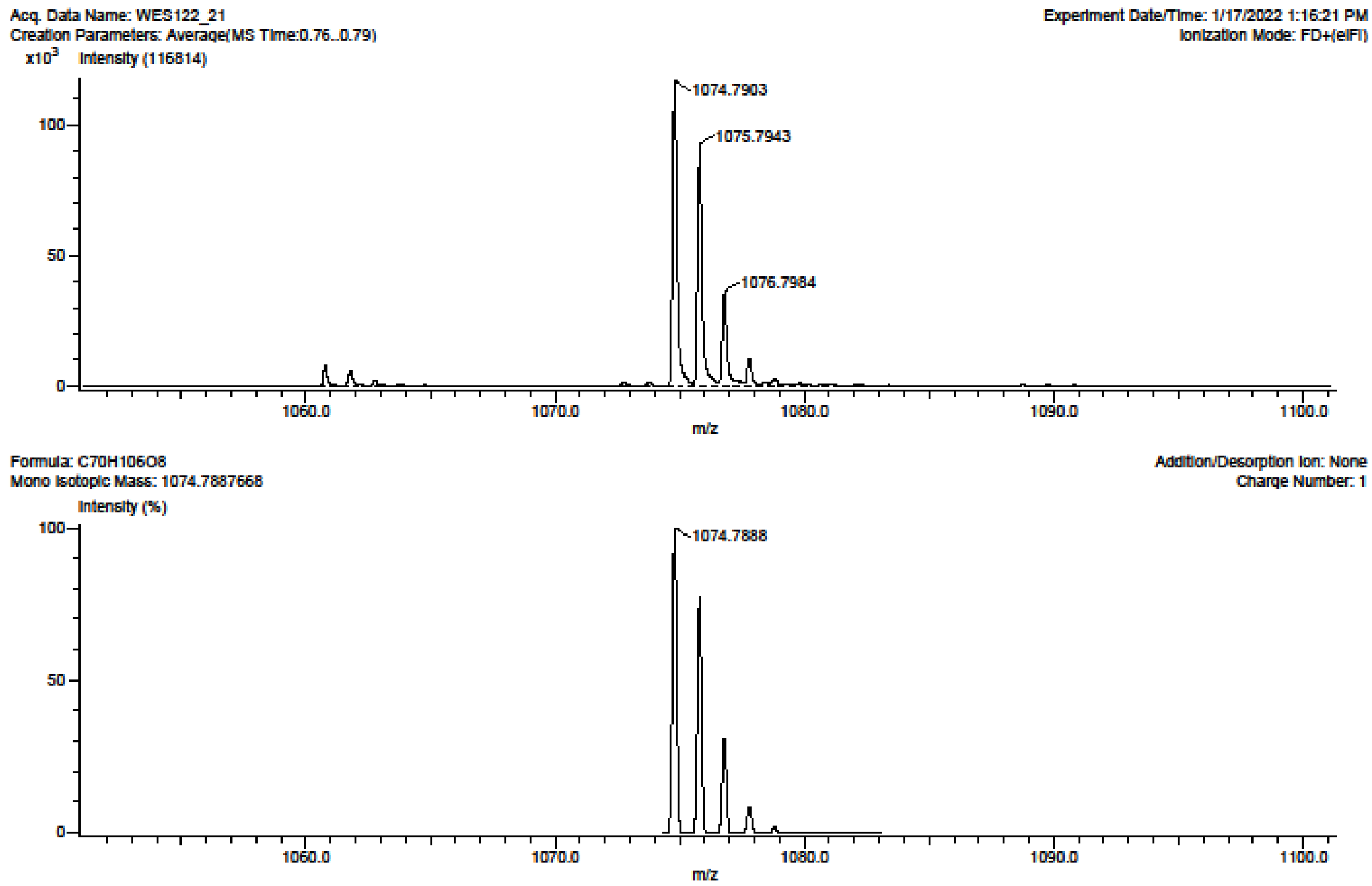

Precatenane 25b. ^{1}H-NMR (400 MHz, $CDCl_3$)

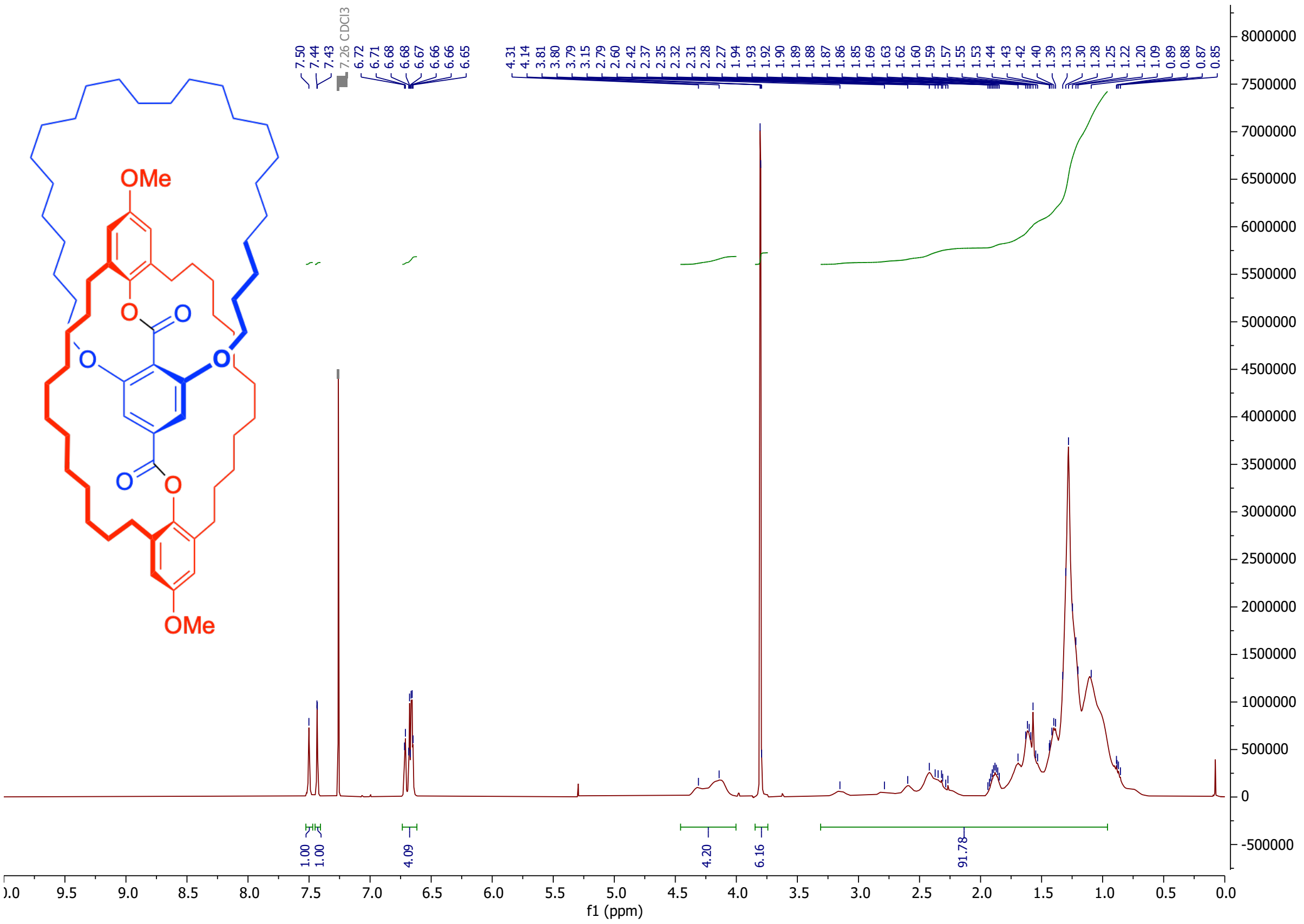

^{13}C{^{1}H}-NMR (101 MHz, $CDCl_3$)

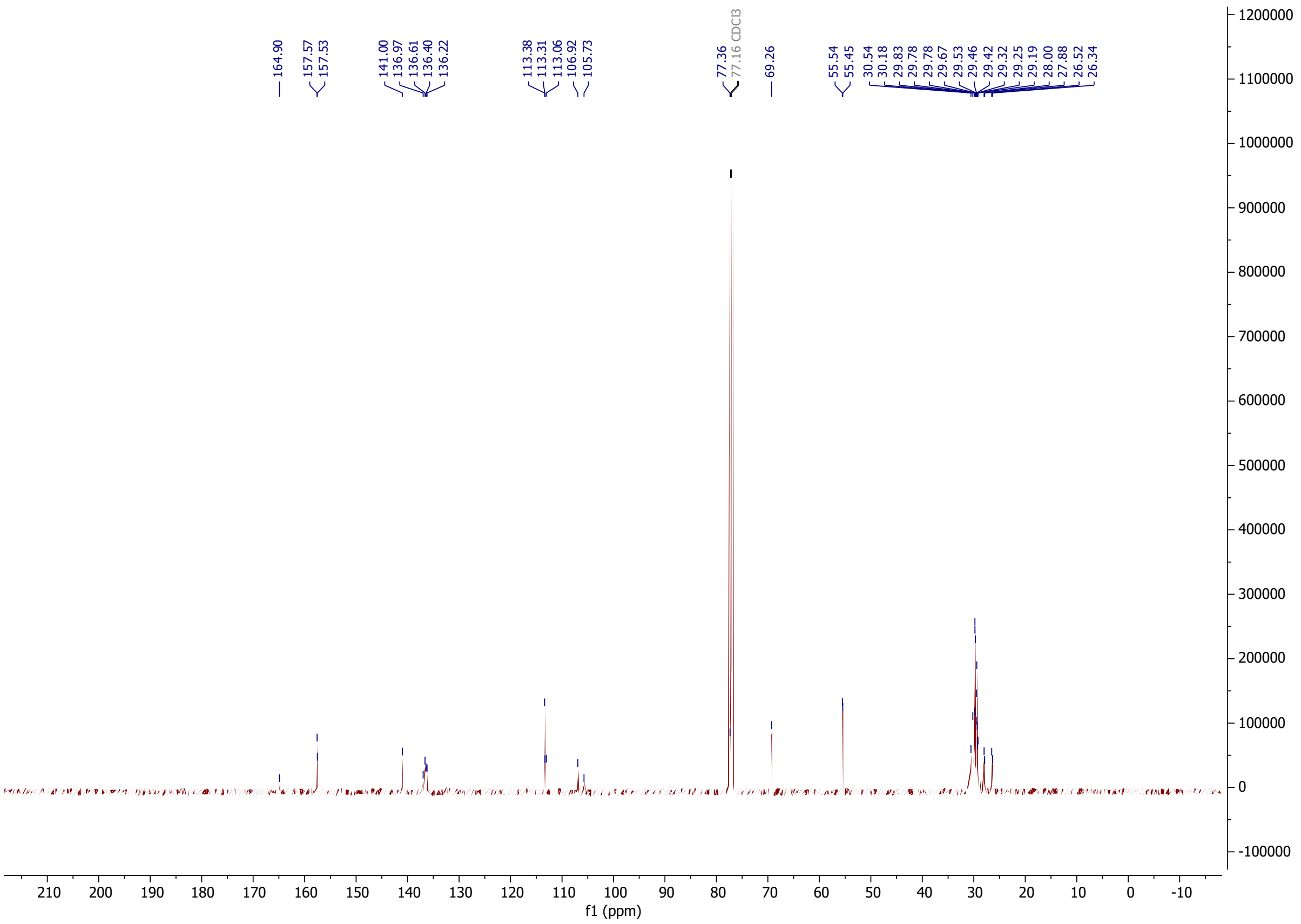

HRMS

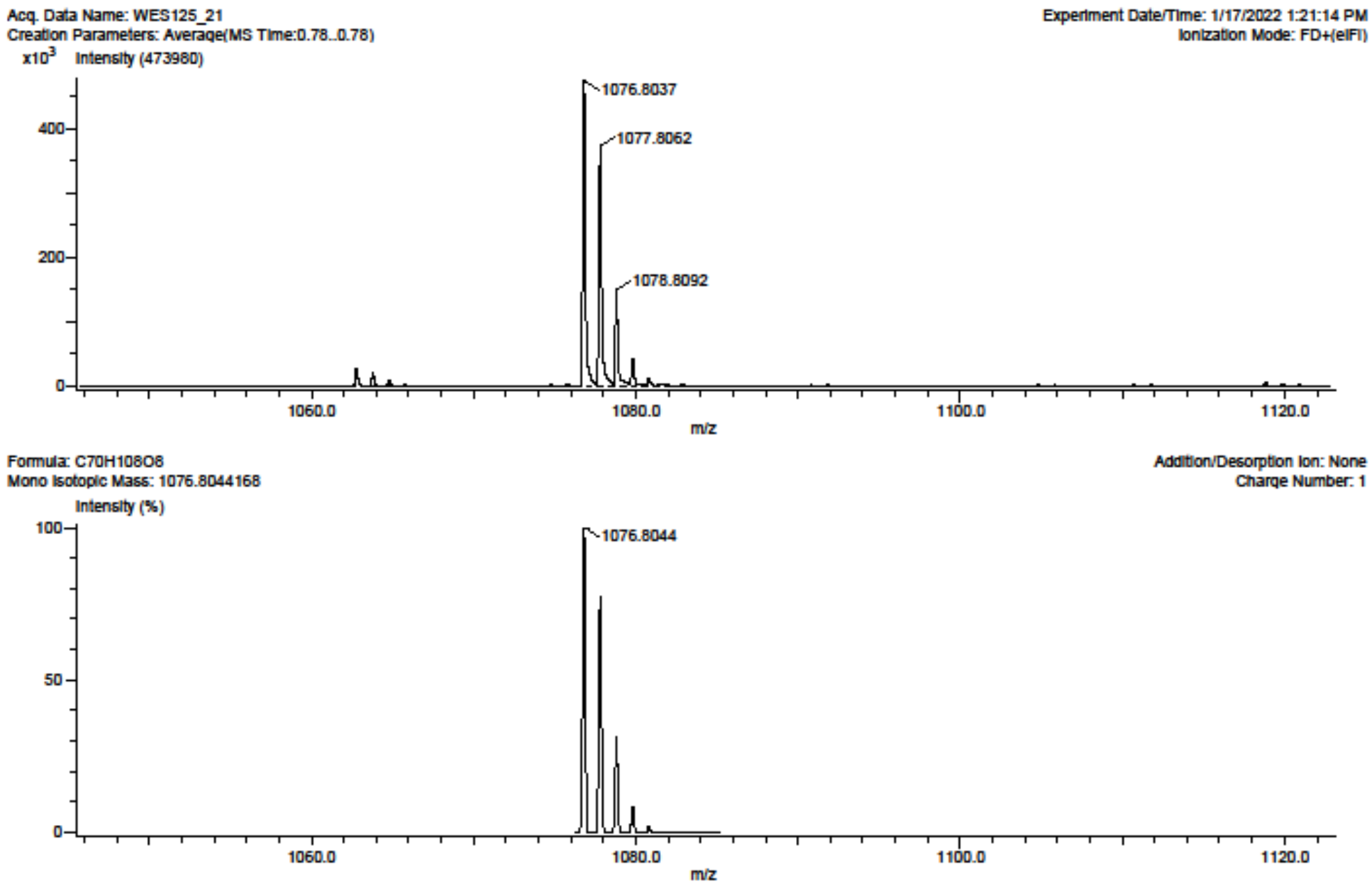

2,6-Di(hept-6-en-1-yl)-4-methoxyphenyl acetate (27). ^{1}H-NMR (400 MHz, $CDCl_3$)

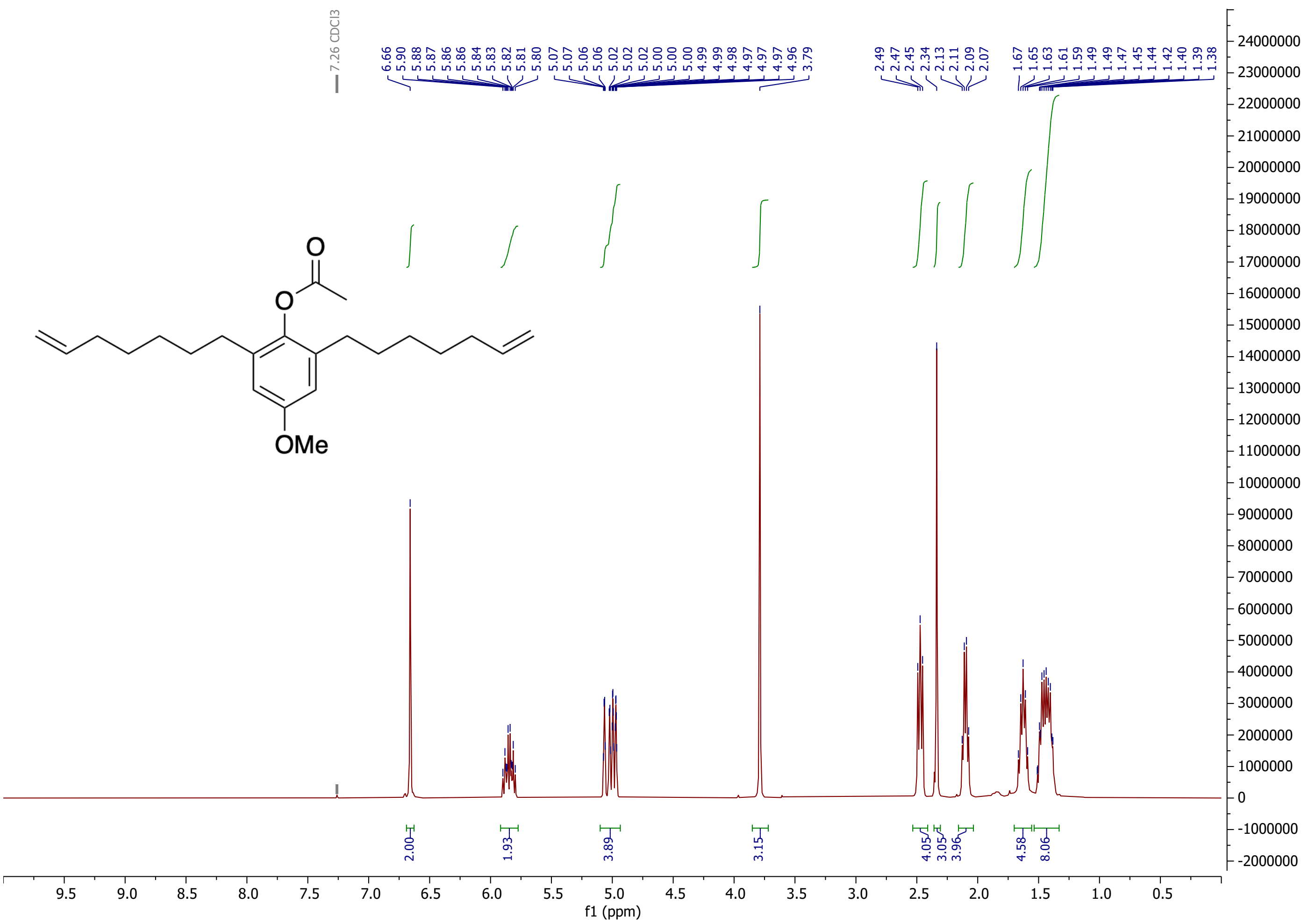

$^{13}C\{^1H\}$-NMR (101 MHz, $CDCl_3$)

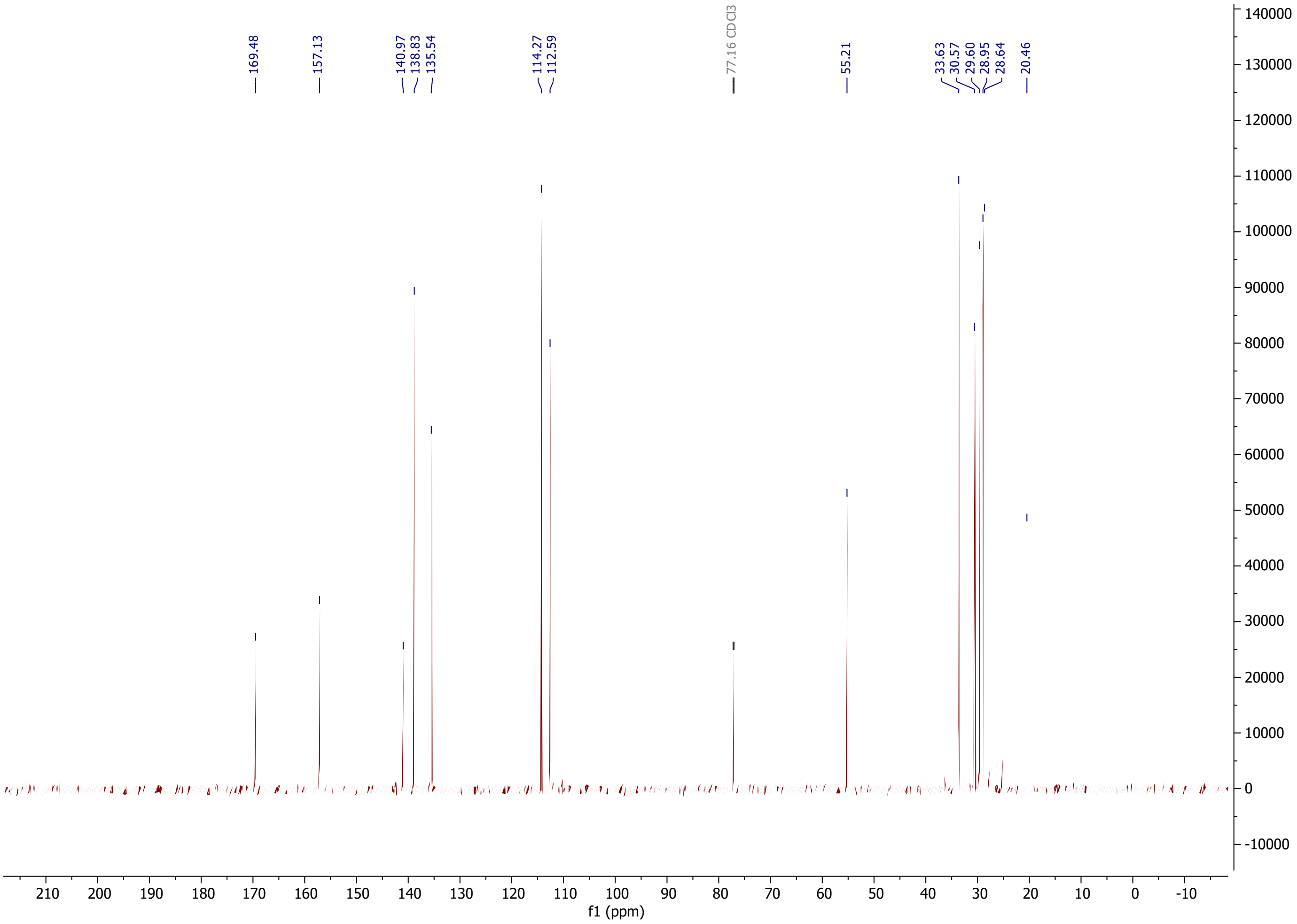

HRMS

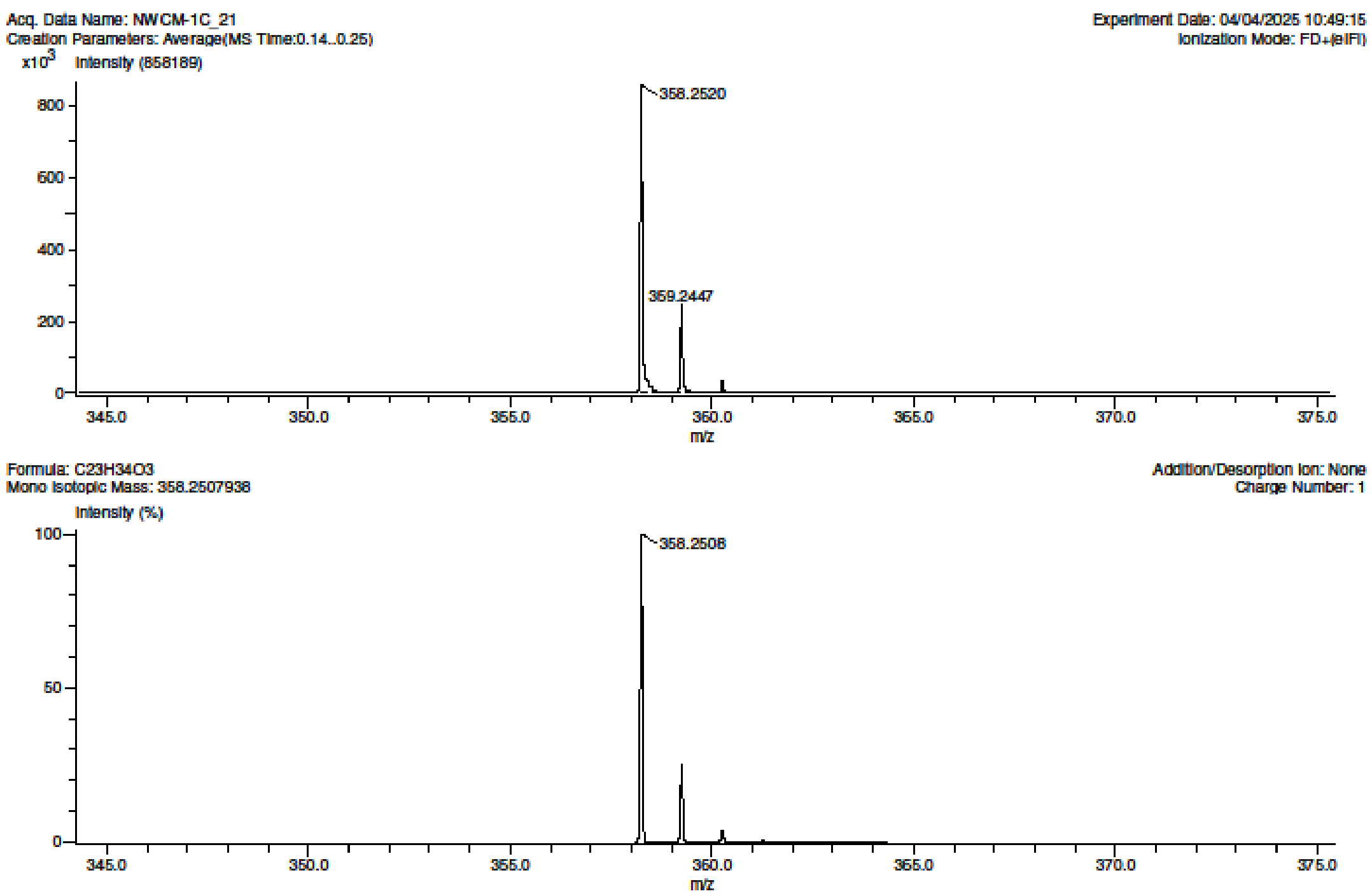

2,6-Di(hept-6-en-1-yl)-4-methoxyphenyl benzoate (28) ^{1}H-NMR (400 MHz, CDCl$_3$)

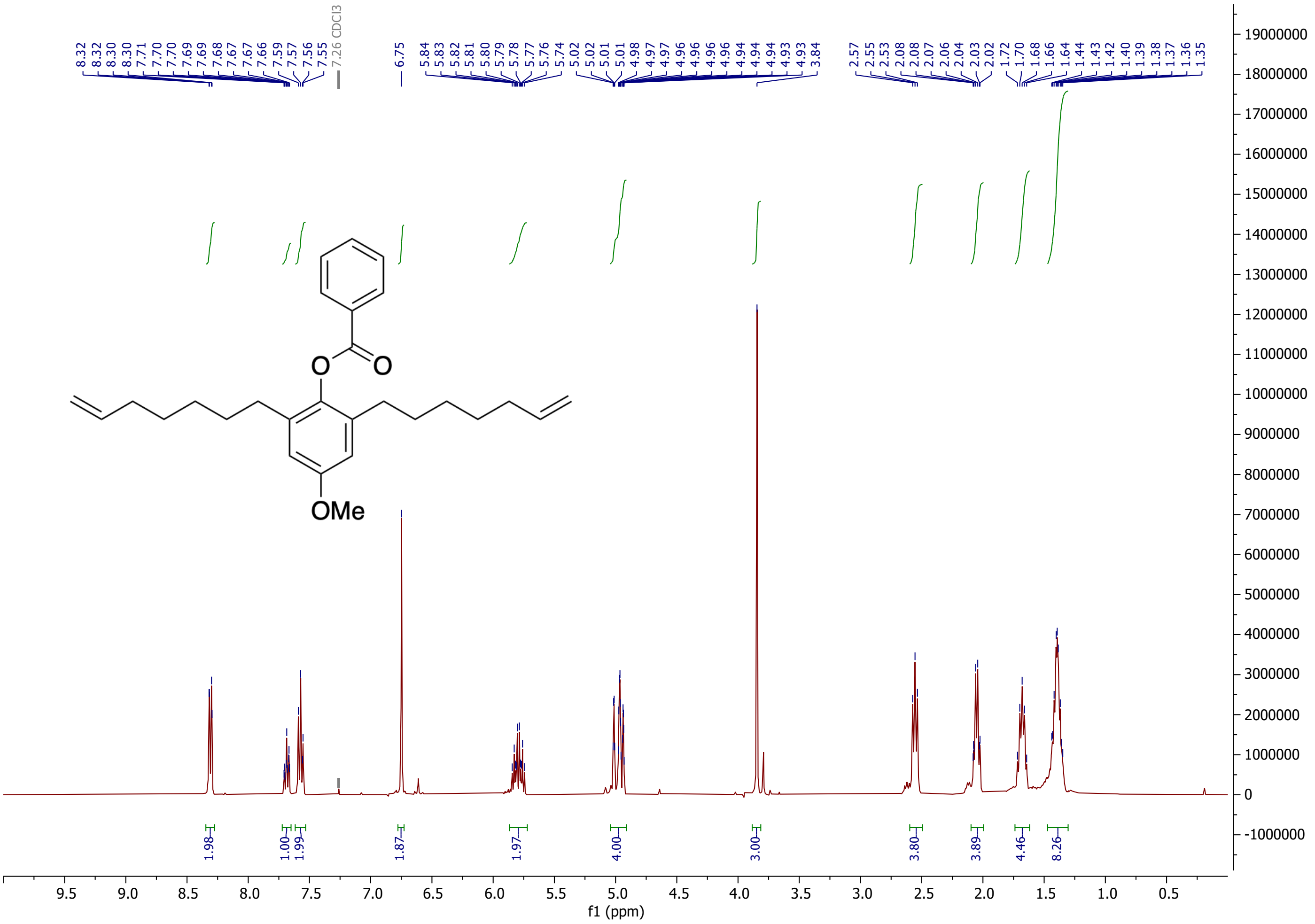

^{13}C{^{1}H}-NMR (101 MHz, CDCl$_3$)

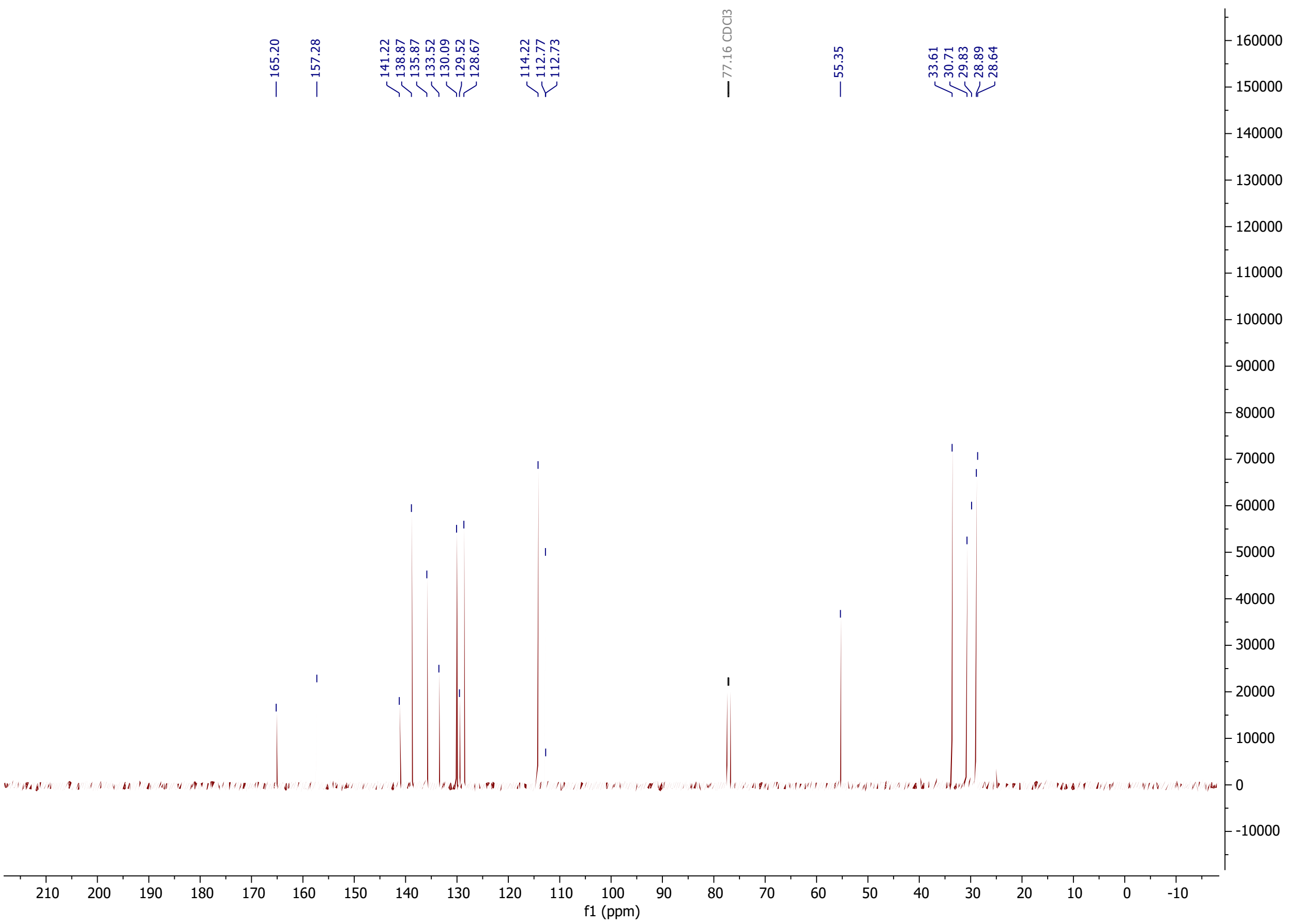

HRMS

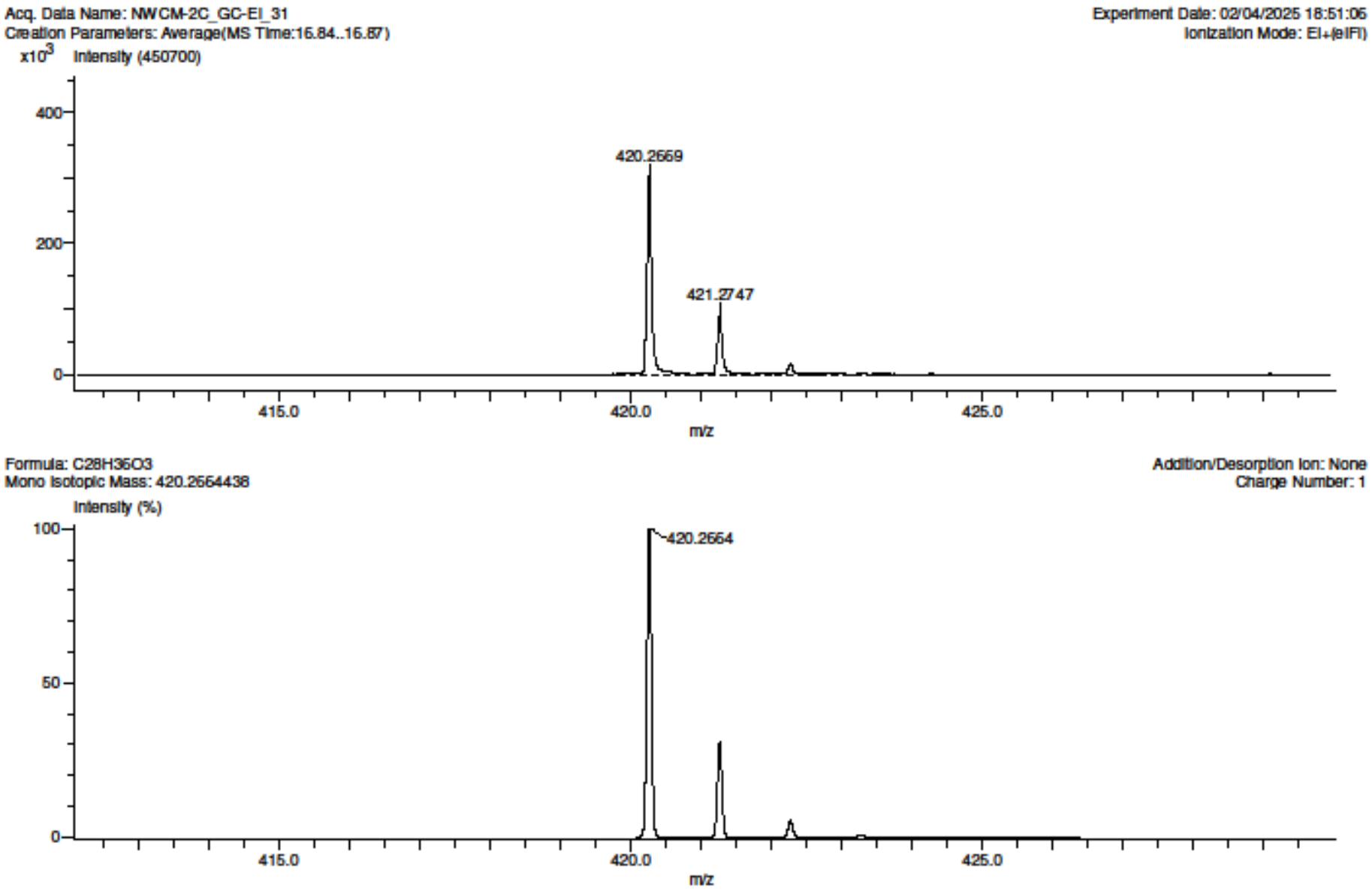

2,6-Di(hept-6-en-1-yl)-4-methoxyphenyl 2,6-dimethoxybenzoate (29). ^{1}**H-NMR (400 MHz, CDCl$_3$)**

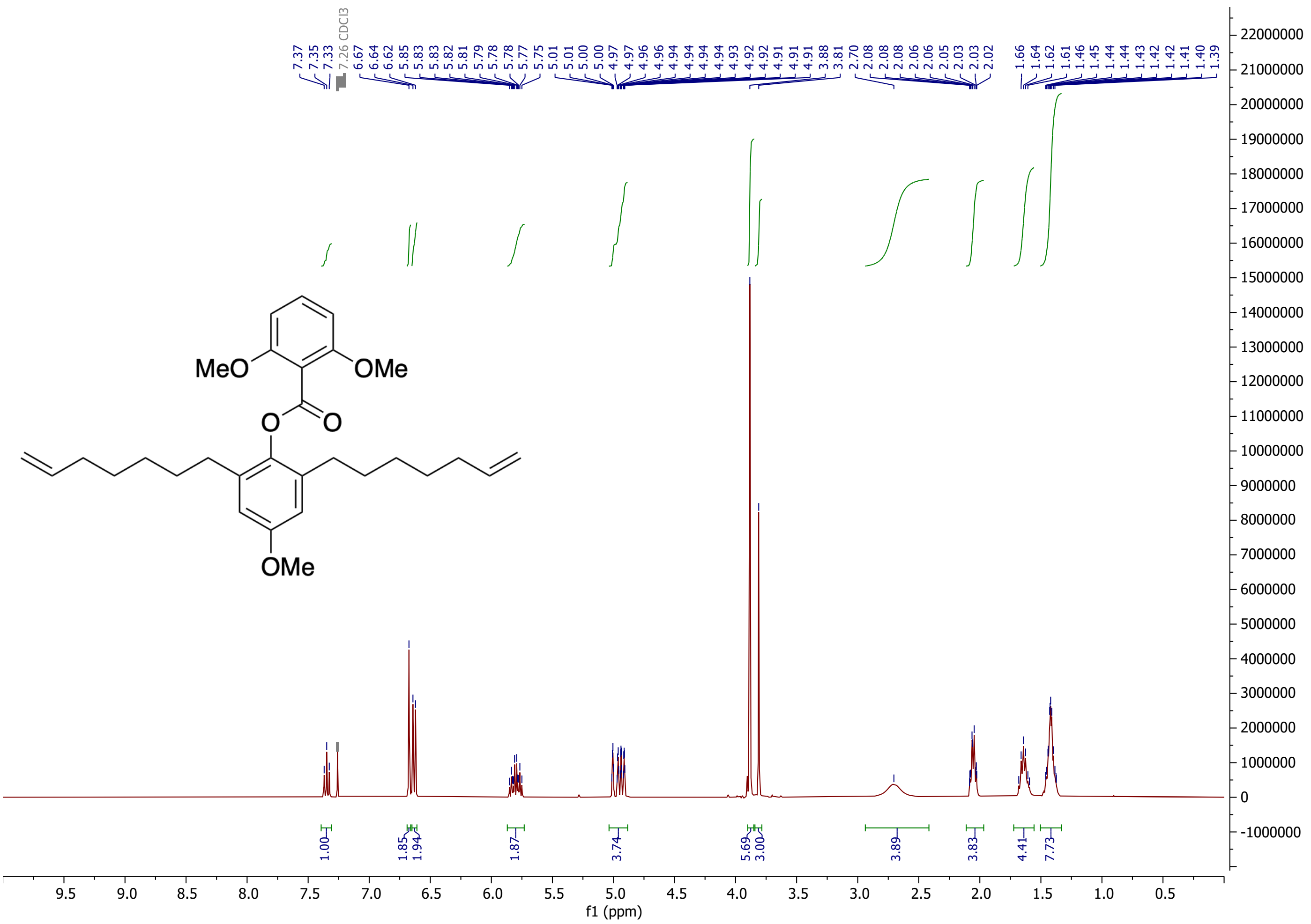

^{13}C{^{1}H}-NMR (101 MHz, CDCl$_3$)

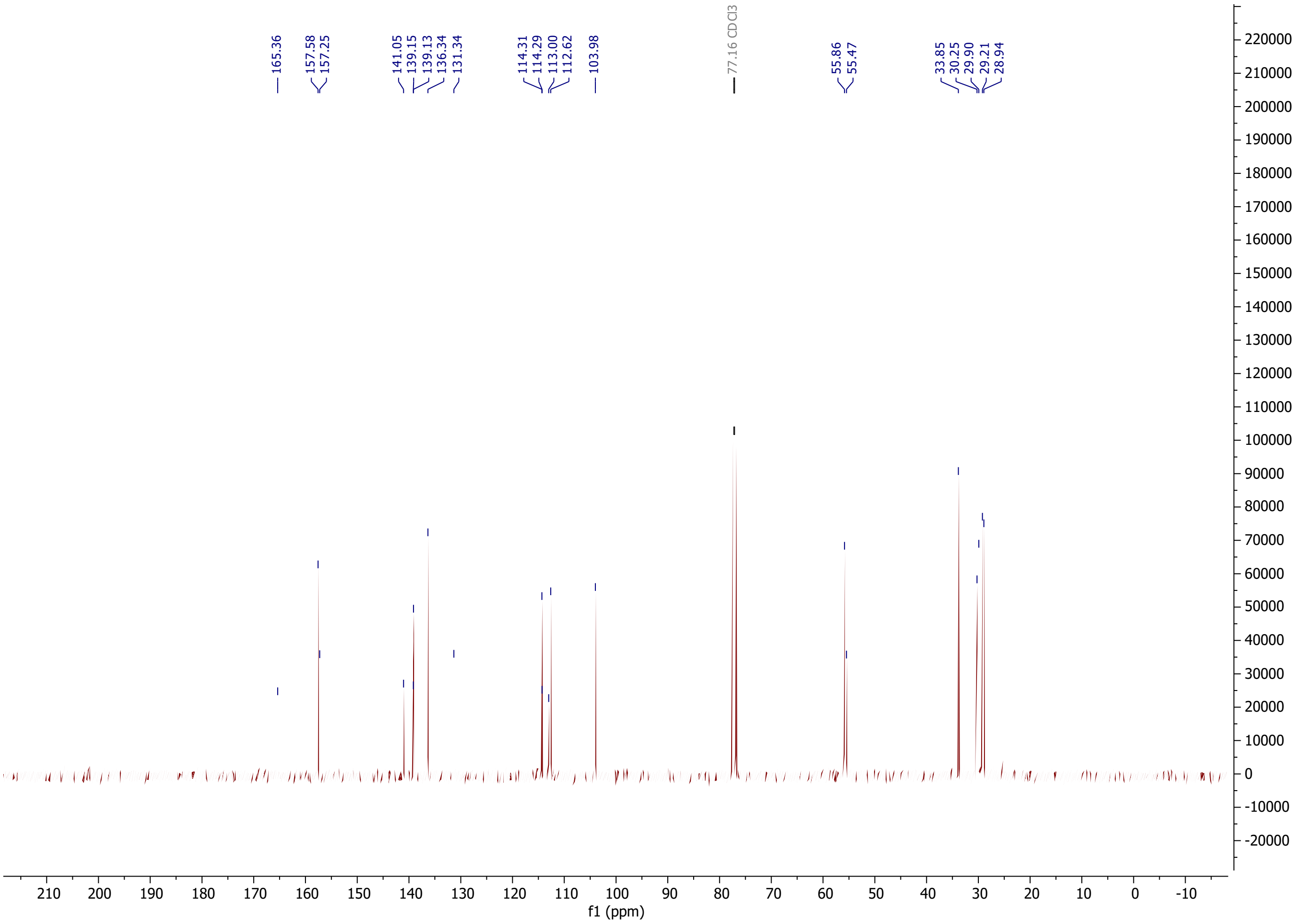
165.36
157.58
157.25
141.05
139.15
139.13
136.34
131.34
114.31
114.29
113.00
112.62
103.98
77.16 CDCl3
55.86
55.47
33.85
30.25
29.90
29.21
28.94
f1 (ppm)

HRMS

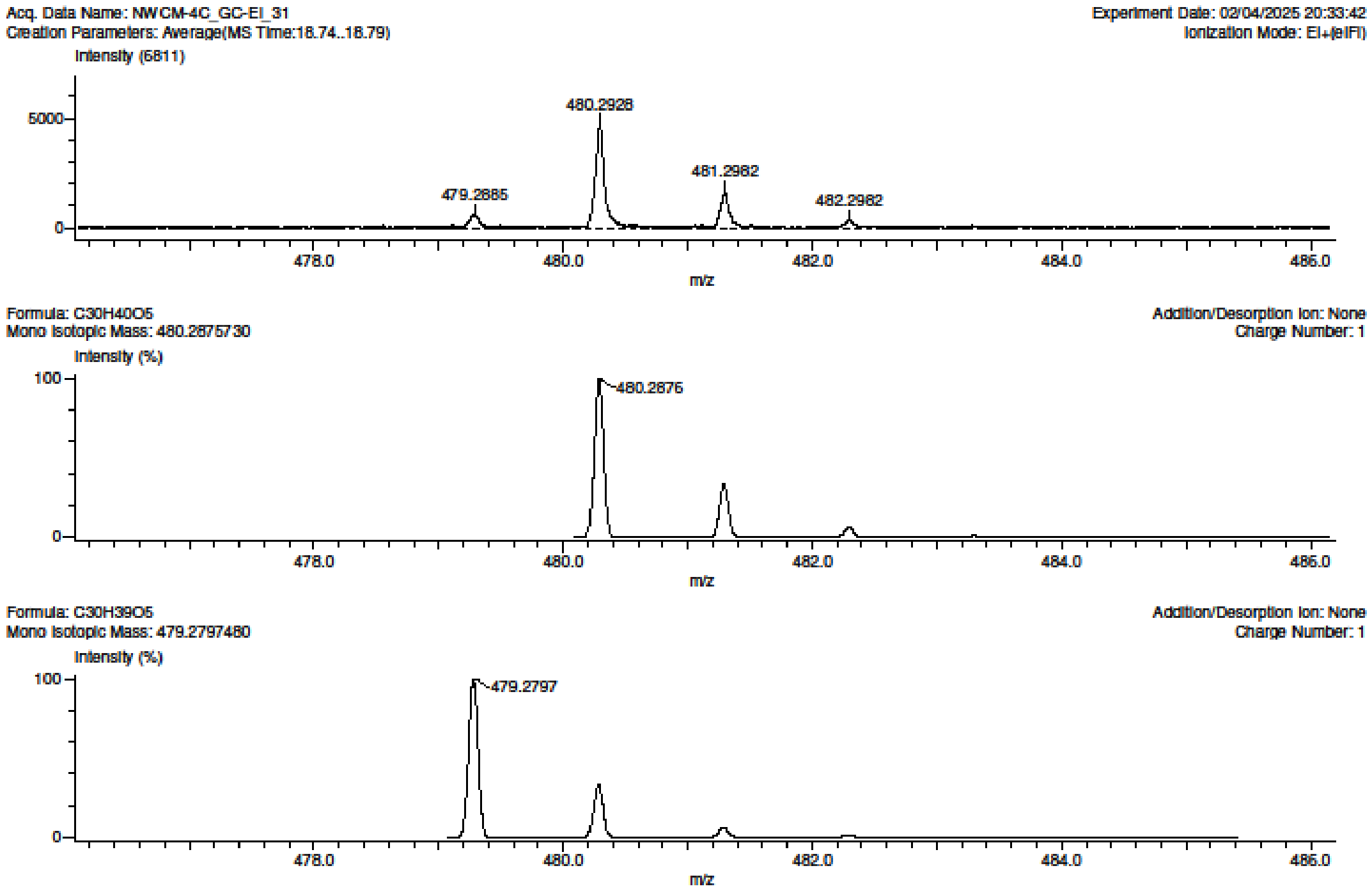
Acq. Data Name: NWCM-4C_GC-EI_31
Creation Parameters: Average(MS Time:18.74..18.79)
Experiment Date: 02/04/2025 20:33:42
Ionization Mode: EI+(eIFI)
Intensity (6811)
480.2928
481.2982
482.2982
479.2885
m/z
Formula: C30H40O5
Mono Isotopic Mass: 480.2875730
Addition/Desorption Ion: None
Charge Number: 1
Intensity (%)
480.2876
m/z
Formula: C30H39O5
Mono Isotopic Mass: 479.2797480
Addition/Desorption Ion: None
Charge Number: 1
Intensity (%)
479.2797
m/z

Bis(2,6-di(hept-6-en-1-yl)-4-methoxyphenyl) 2,6-dimethoxyterephthalate 31. ^{1}H-NMR (400 MHz, CDCl$_3$)

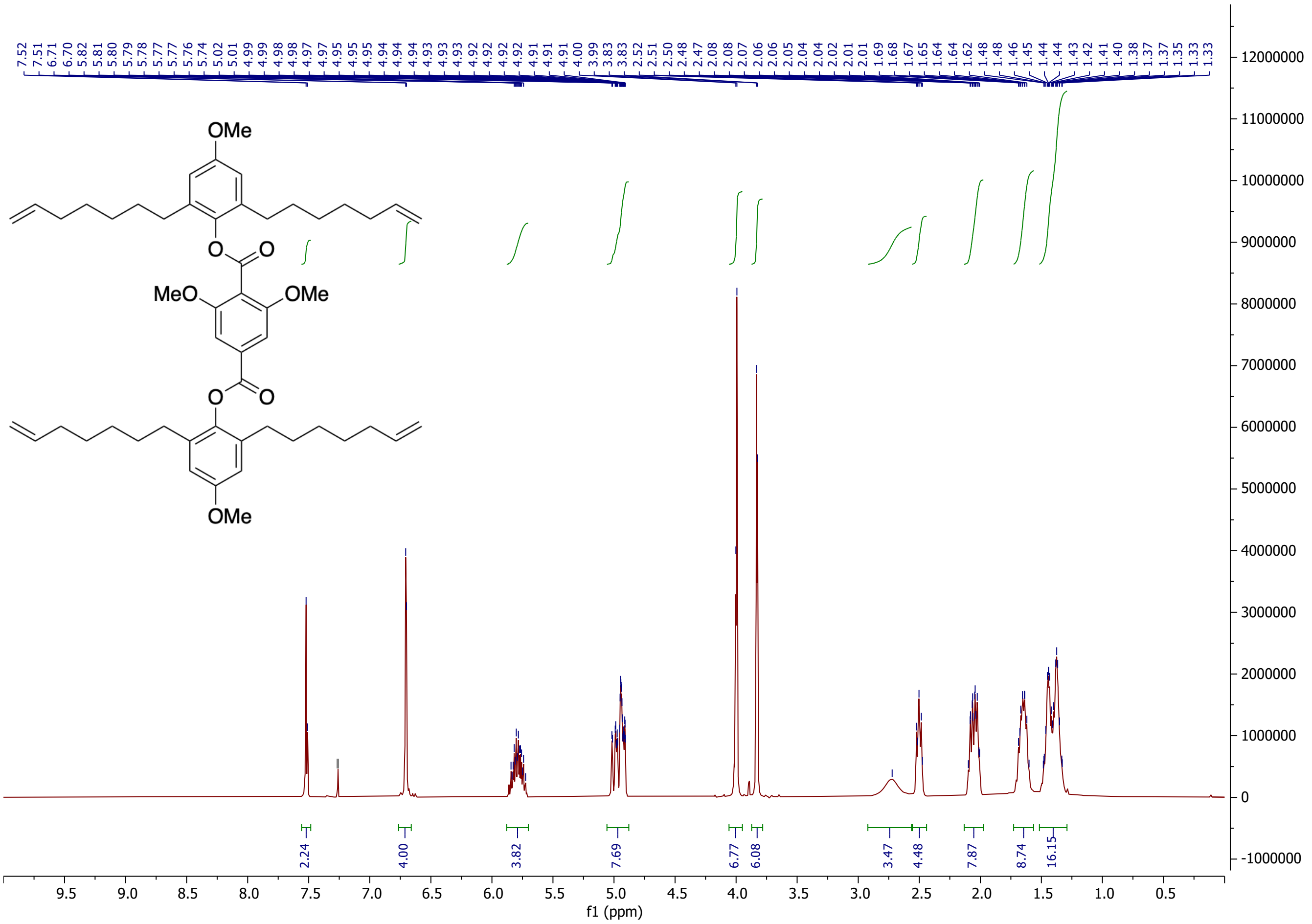

,

^{13}C{^{1}H}-NMR (101 MHz, CDCl$_3$)

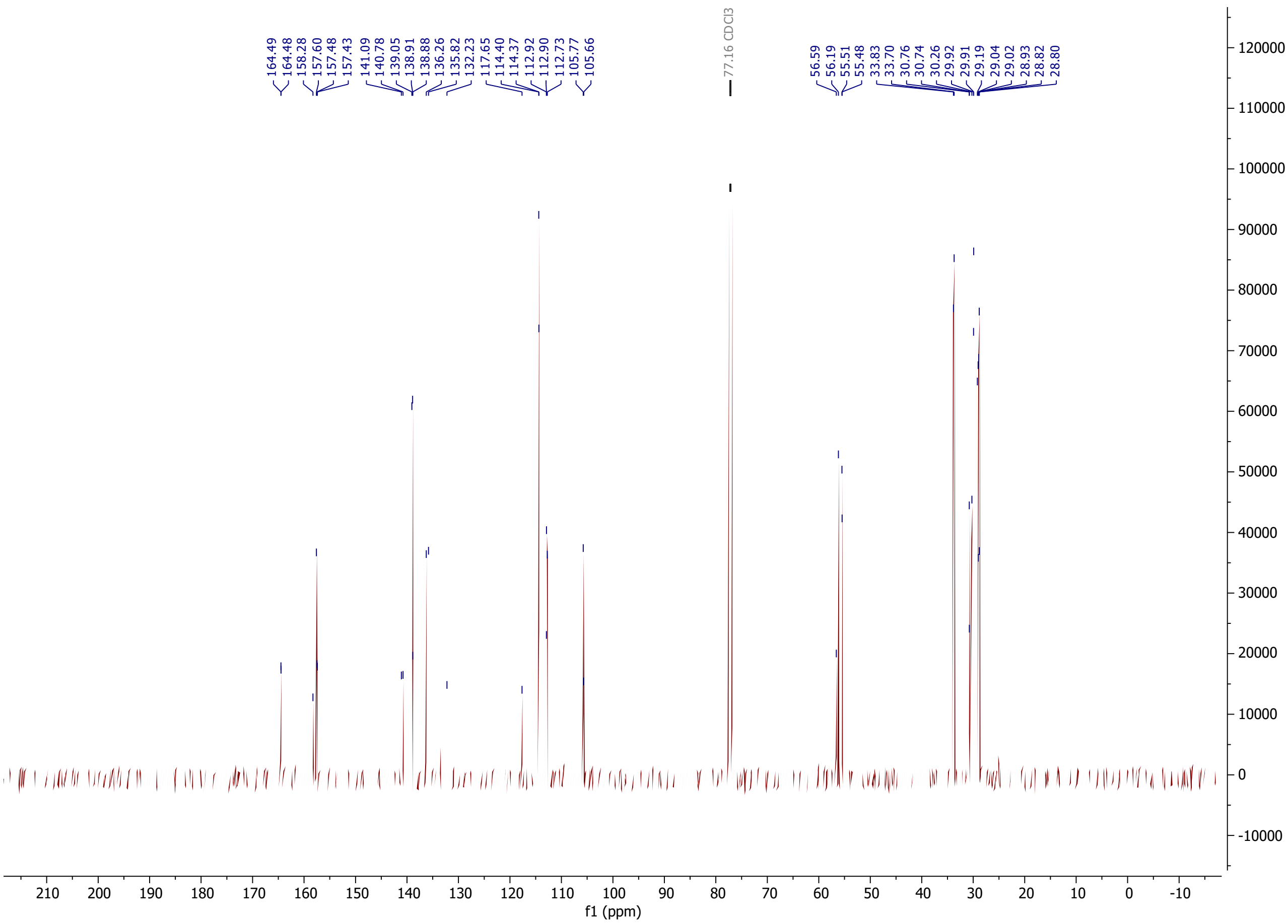

HRMS

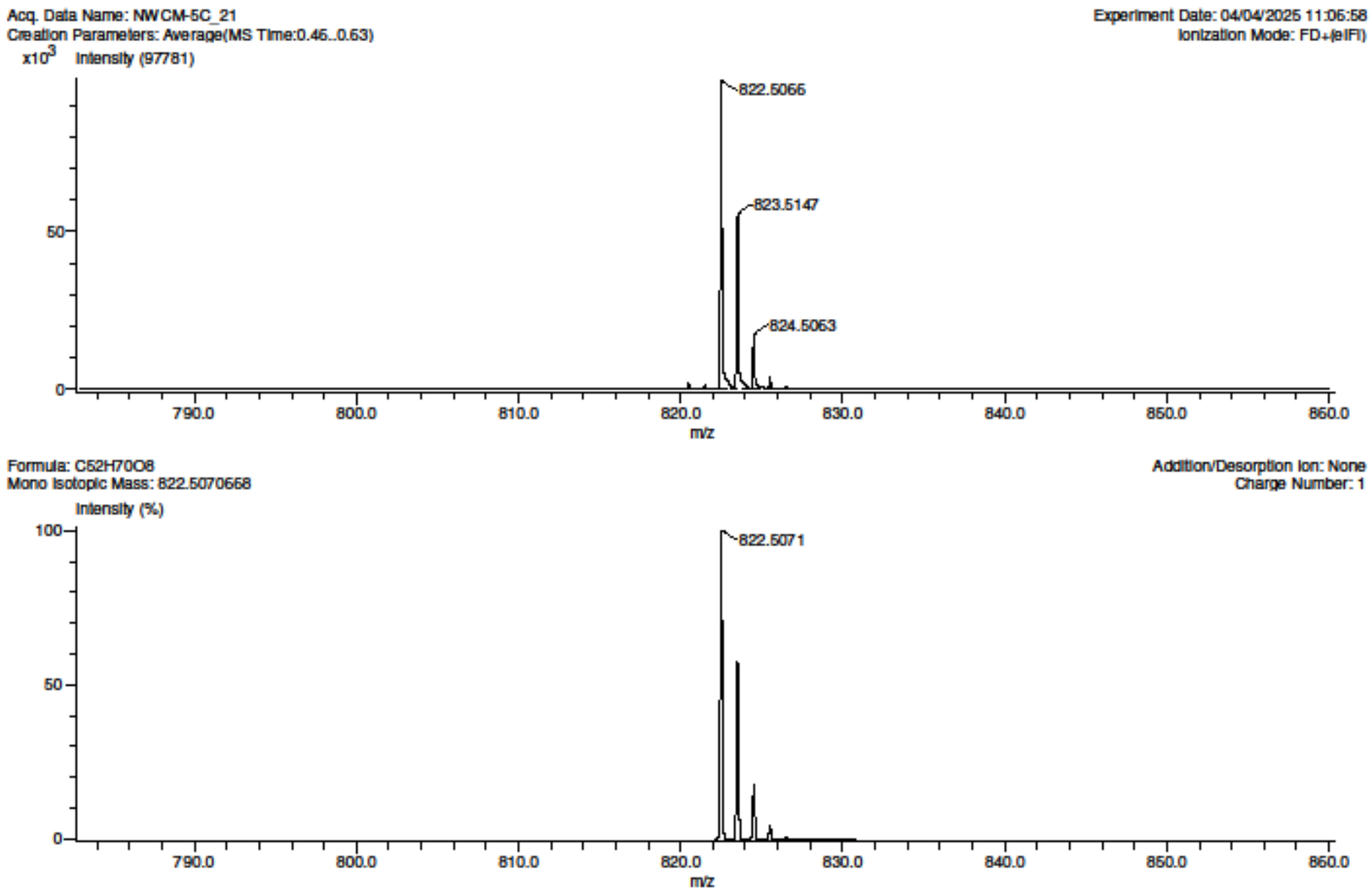

Bis(2,6- di(hept-6-en-1-yl)-4-methoxyphenyl) terephthalate (32). ^{1}**H-NMR (400 MHz, CDCl$_3$)**

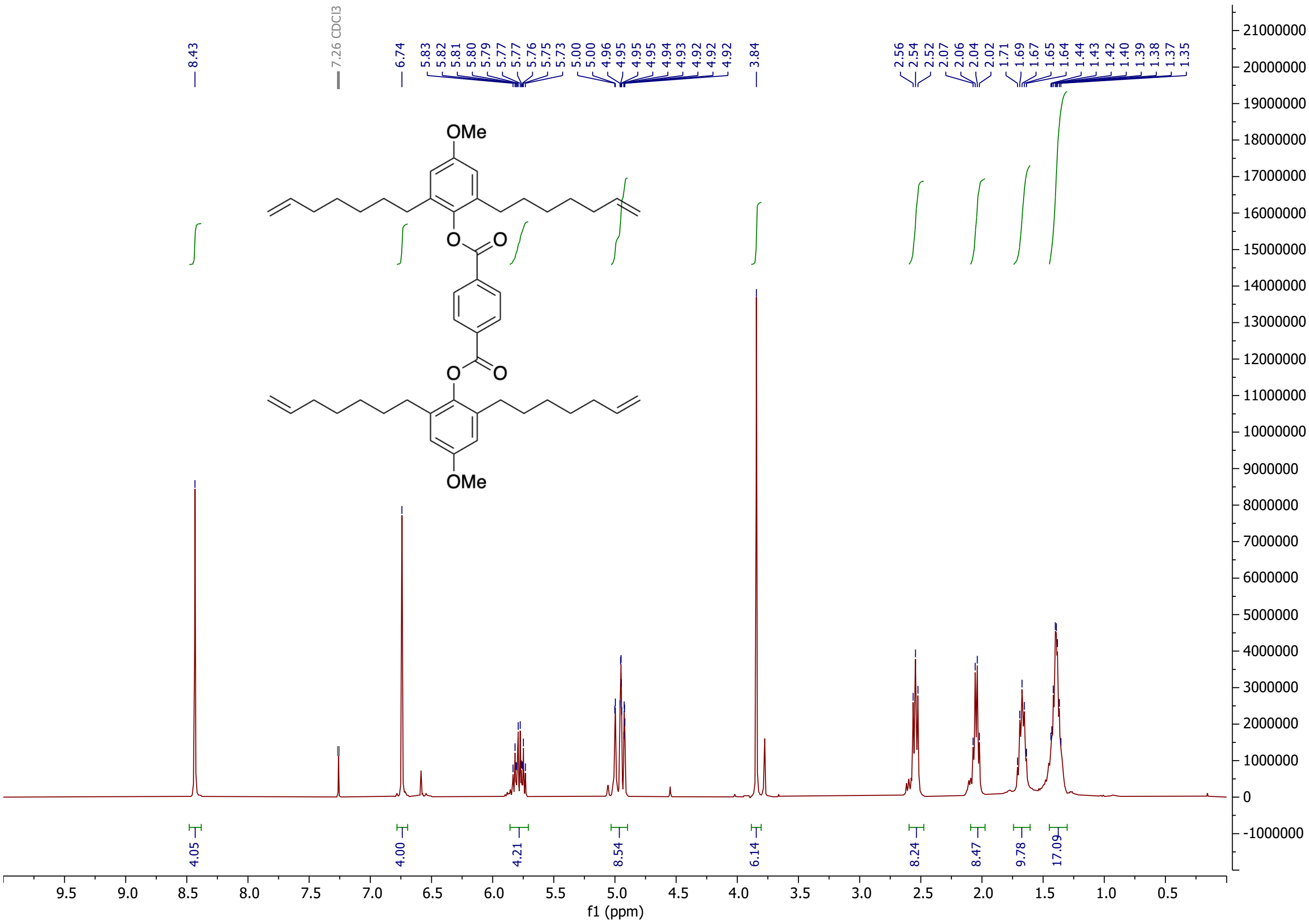

^{13}**C{^{1}H}-NMR (101 MHz, CDCl$_3$)**

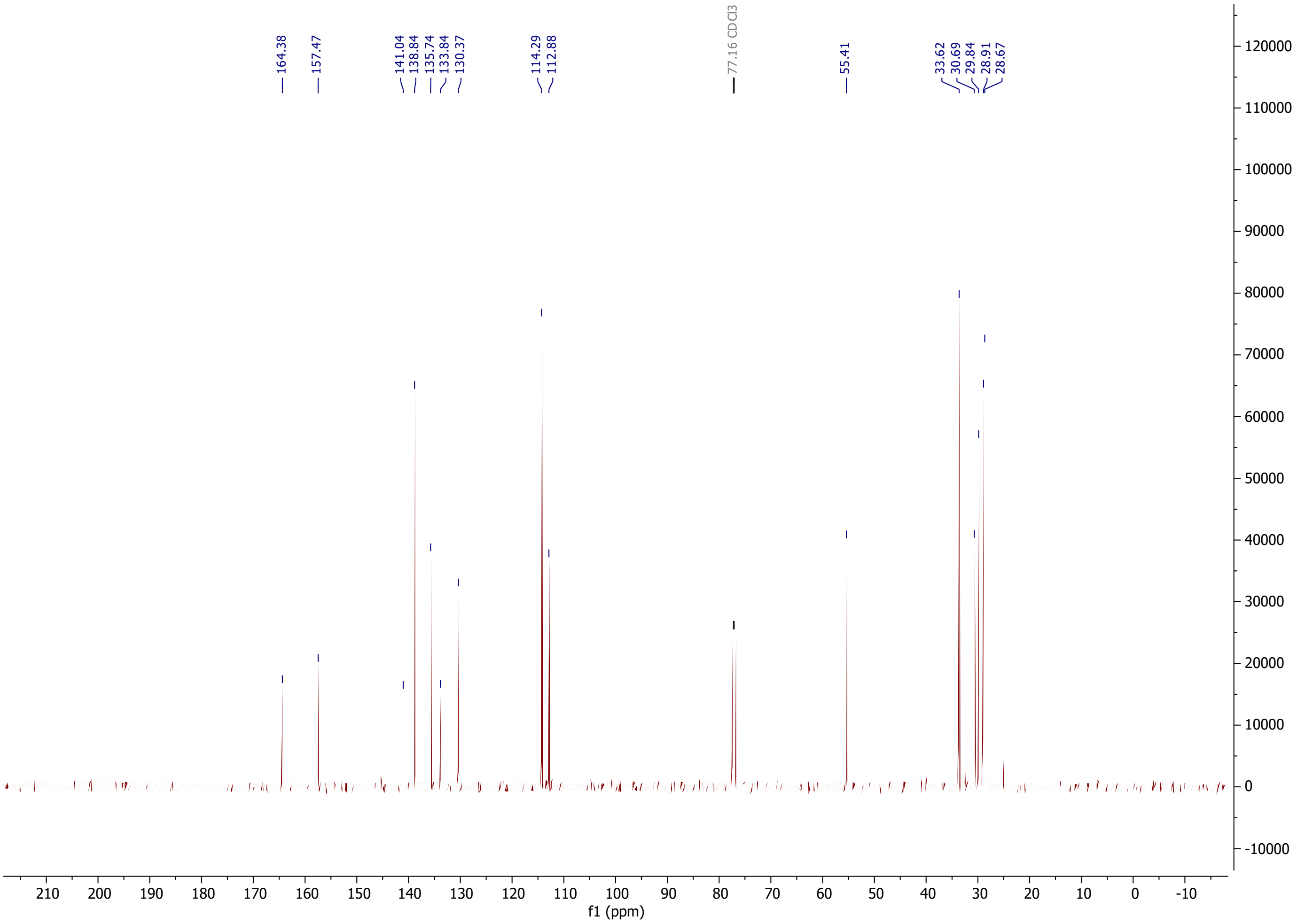

HRMS

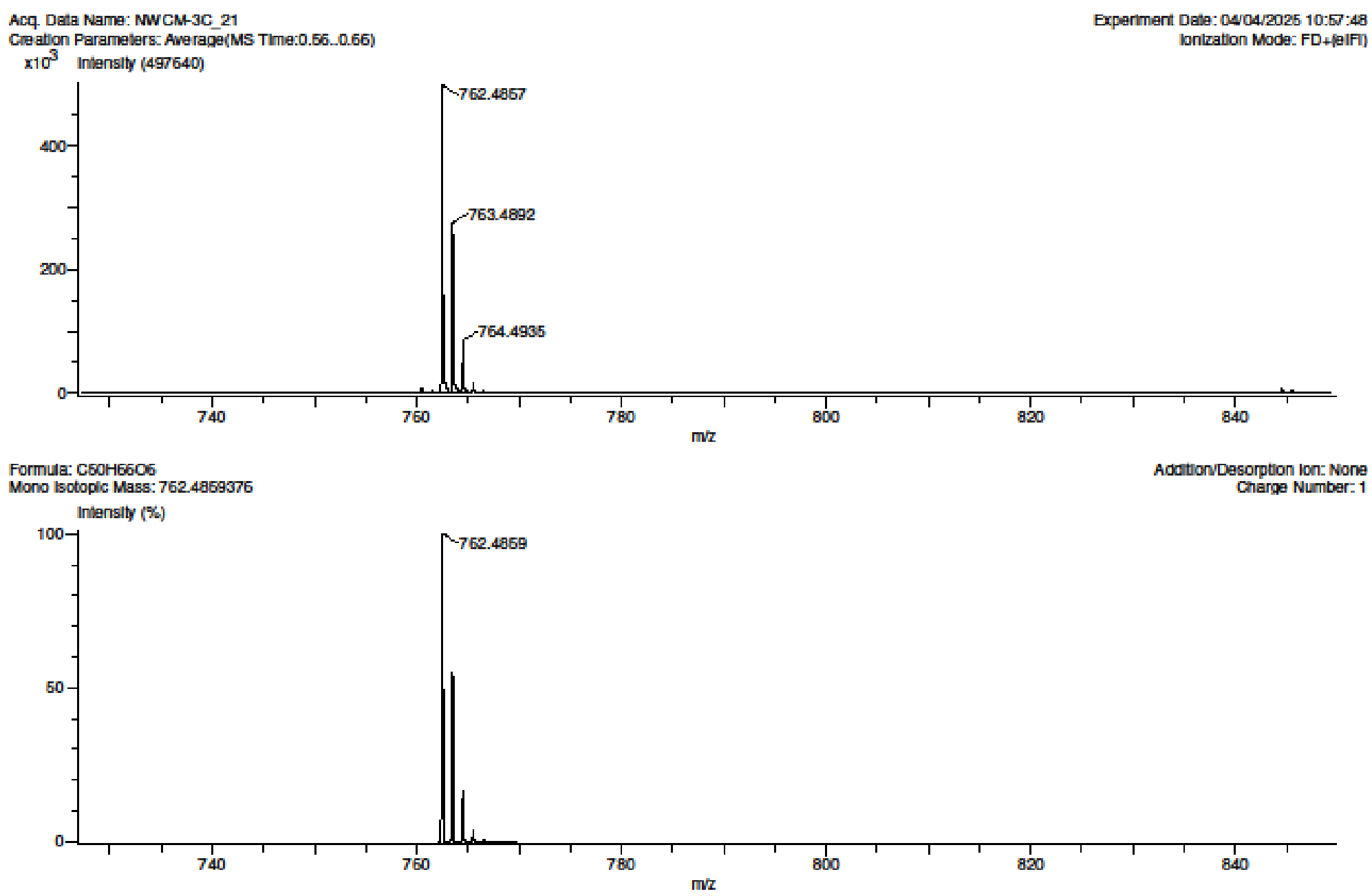

Bis(2,6-di(hept-6-en-1-yl)-4-methoxyphenyl) cyclohexane-1,4-dicarboxylate (33). ^{1}**H-NMR (400 MHz, $CDCl_3$)**

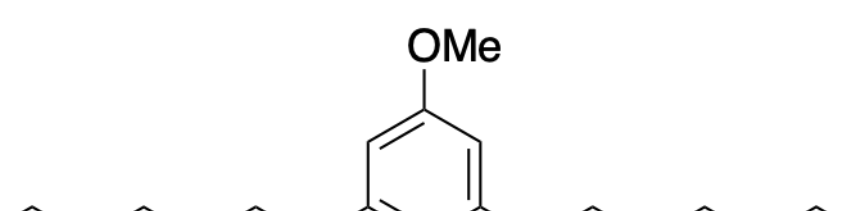

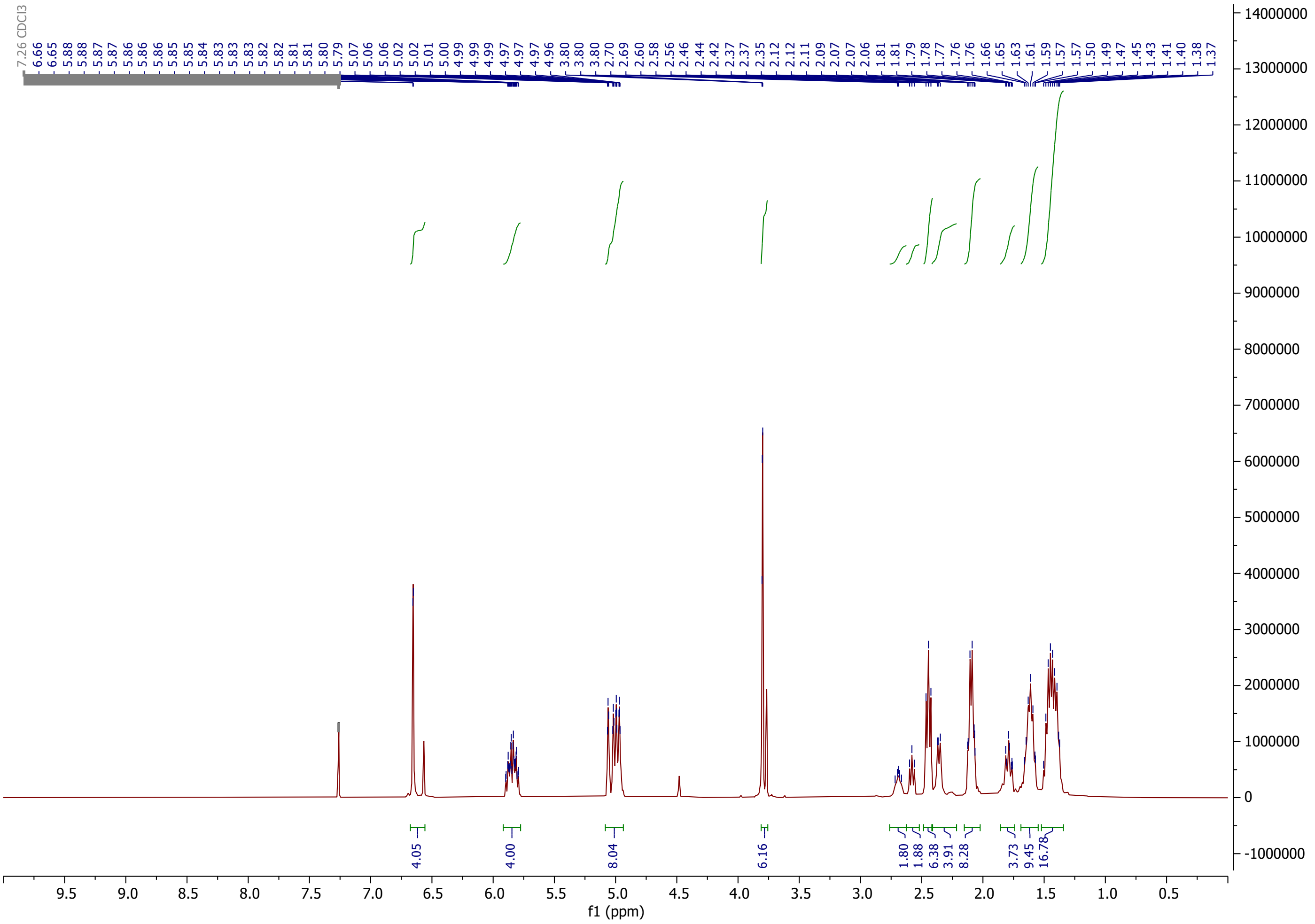

$^{13}C\{^1H\}$-NMR (101 MHz, $CDCl_3$)

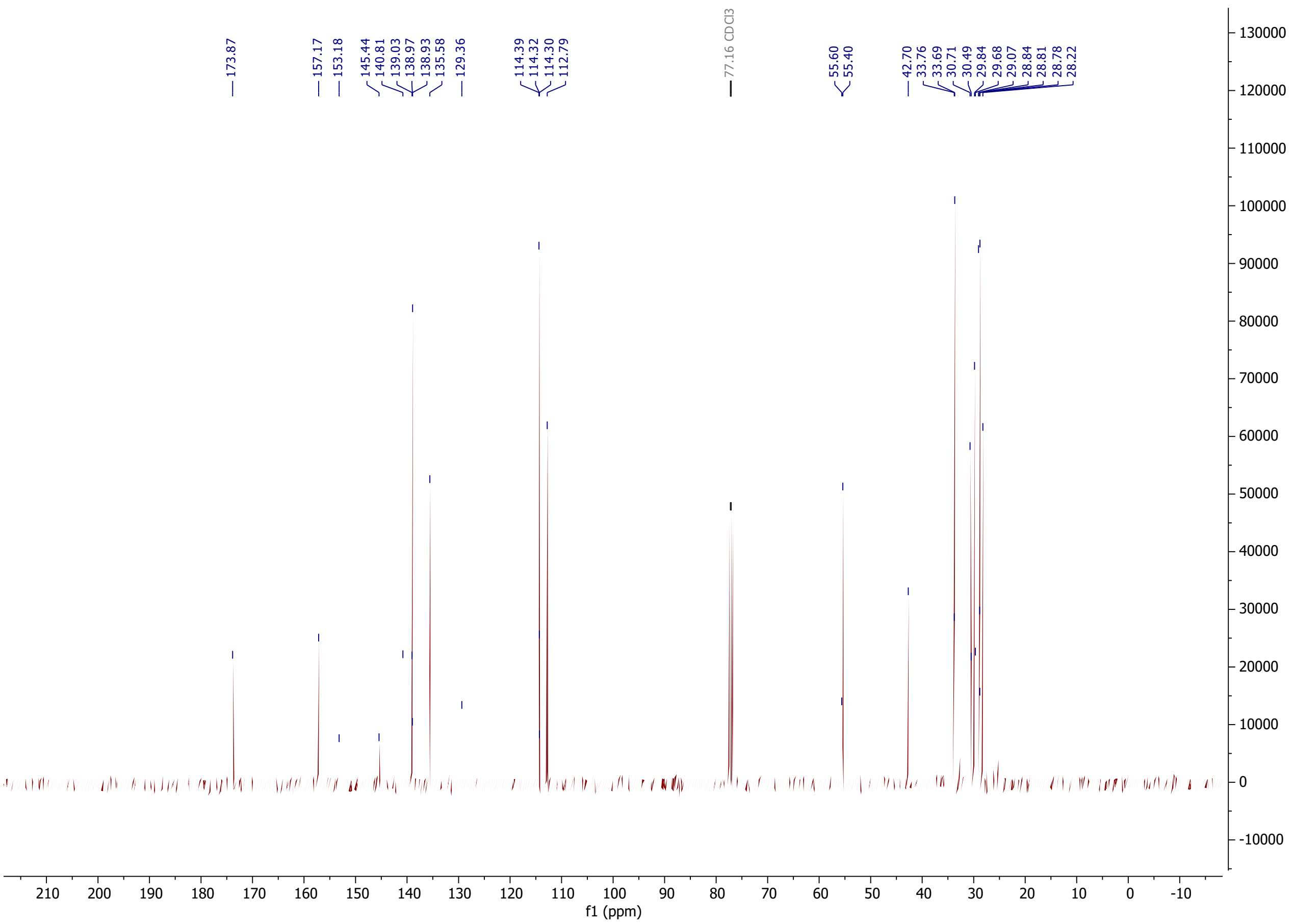

HRMS

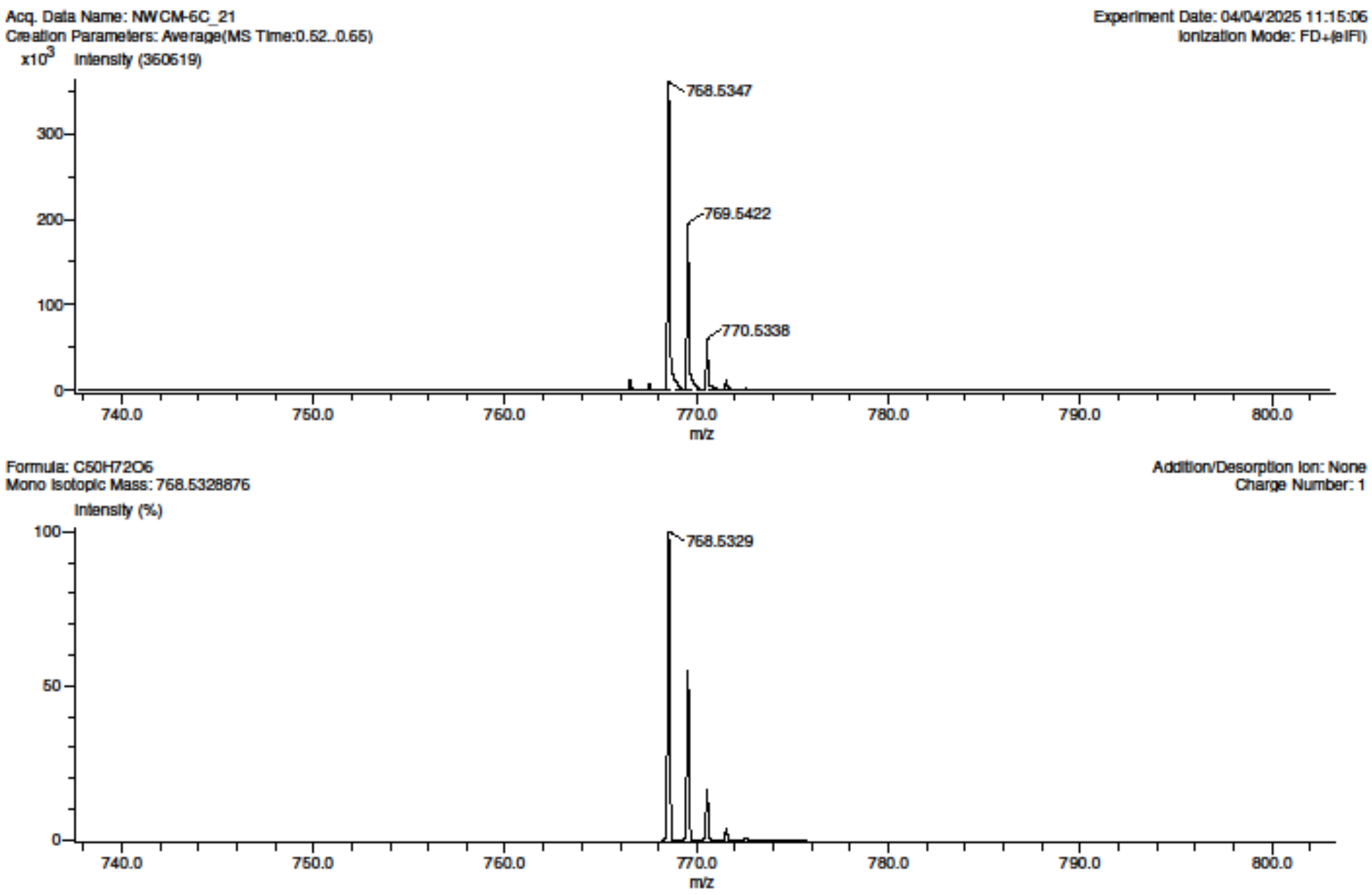

2,6-Di(hept-6-en-1-yl)cyclohexa-2,5-diene-1,4-dione (30). ^{1}H-NMR (400 MHz, $CDCl_3$)

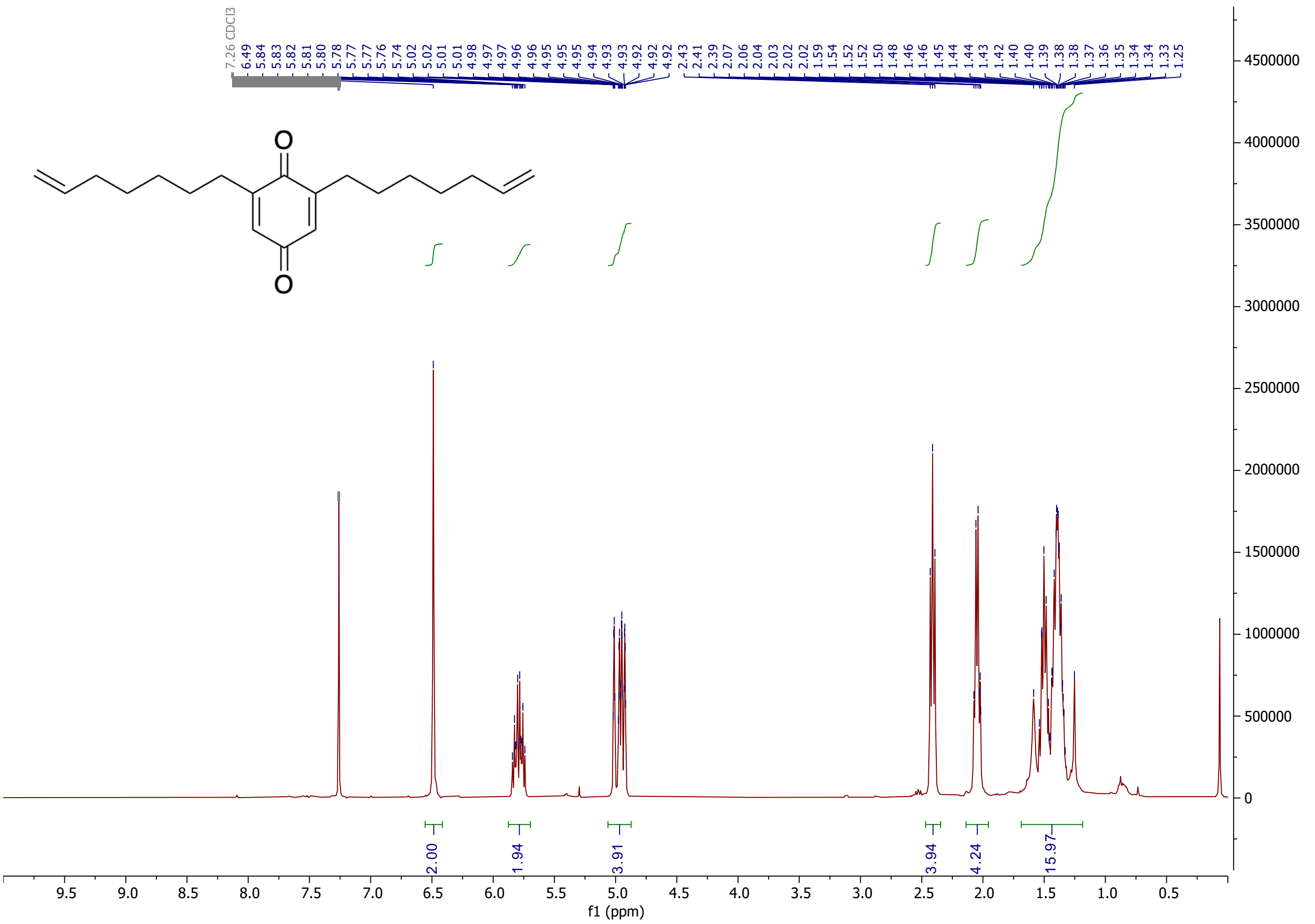

$^{13}C\{^1H\}$-NMR (101 MHz, $CDCl_3$)

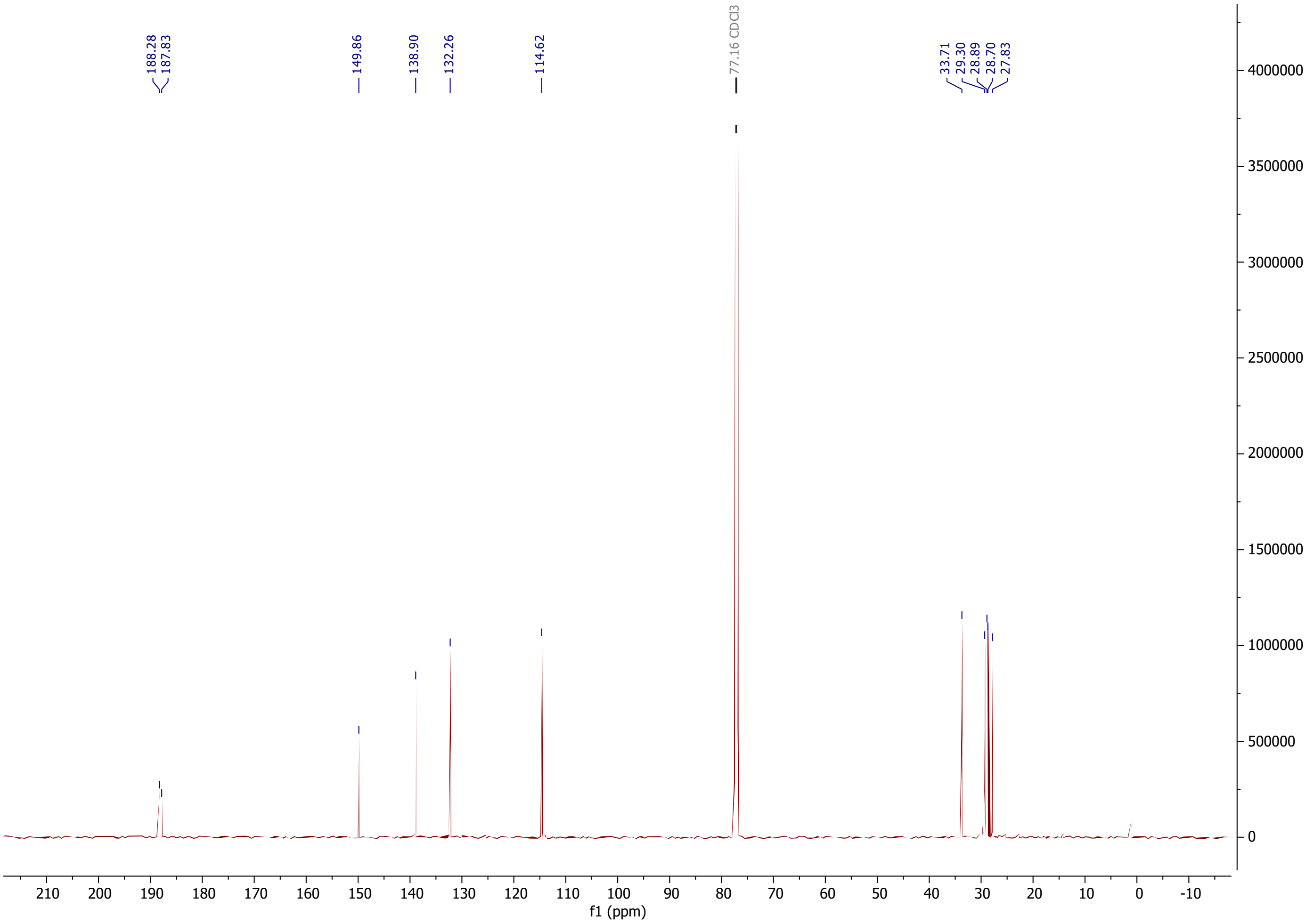

HRMS

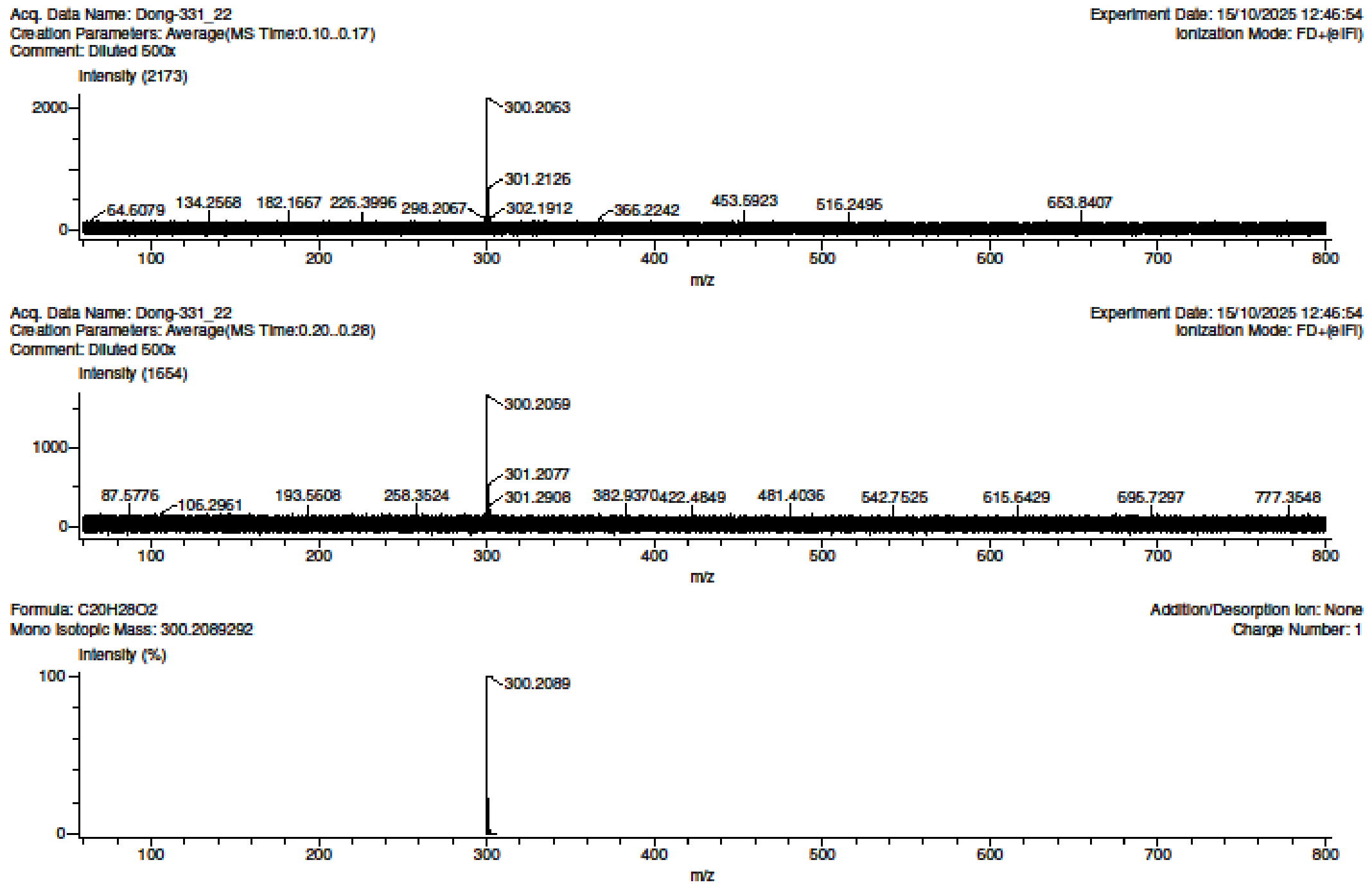

Catenane 34. ¹H-NMR (400 MHz, $CDCl_3$)

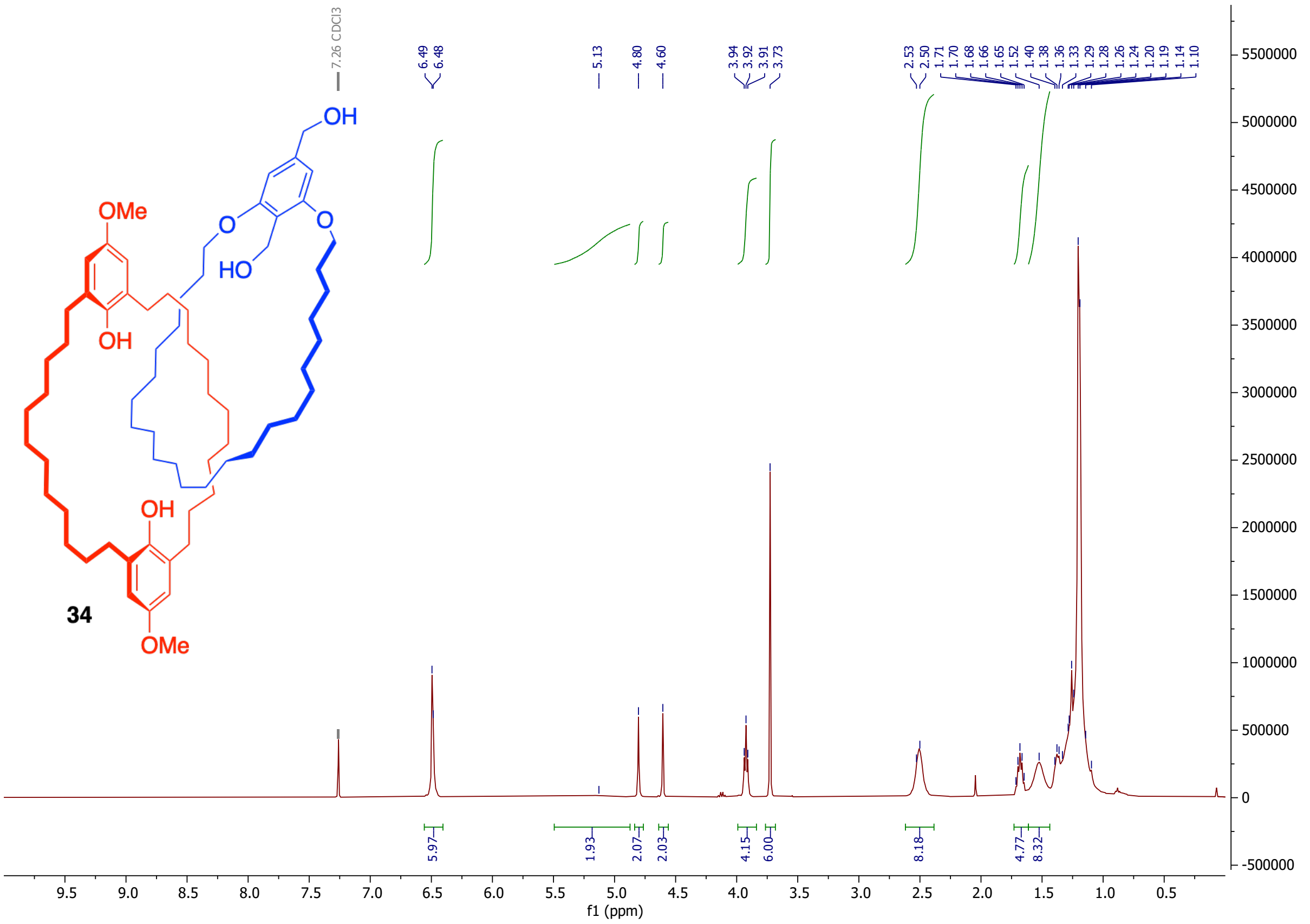

¹³C-NMR (101 MHz, $CDCl_3$)

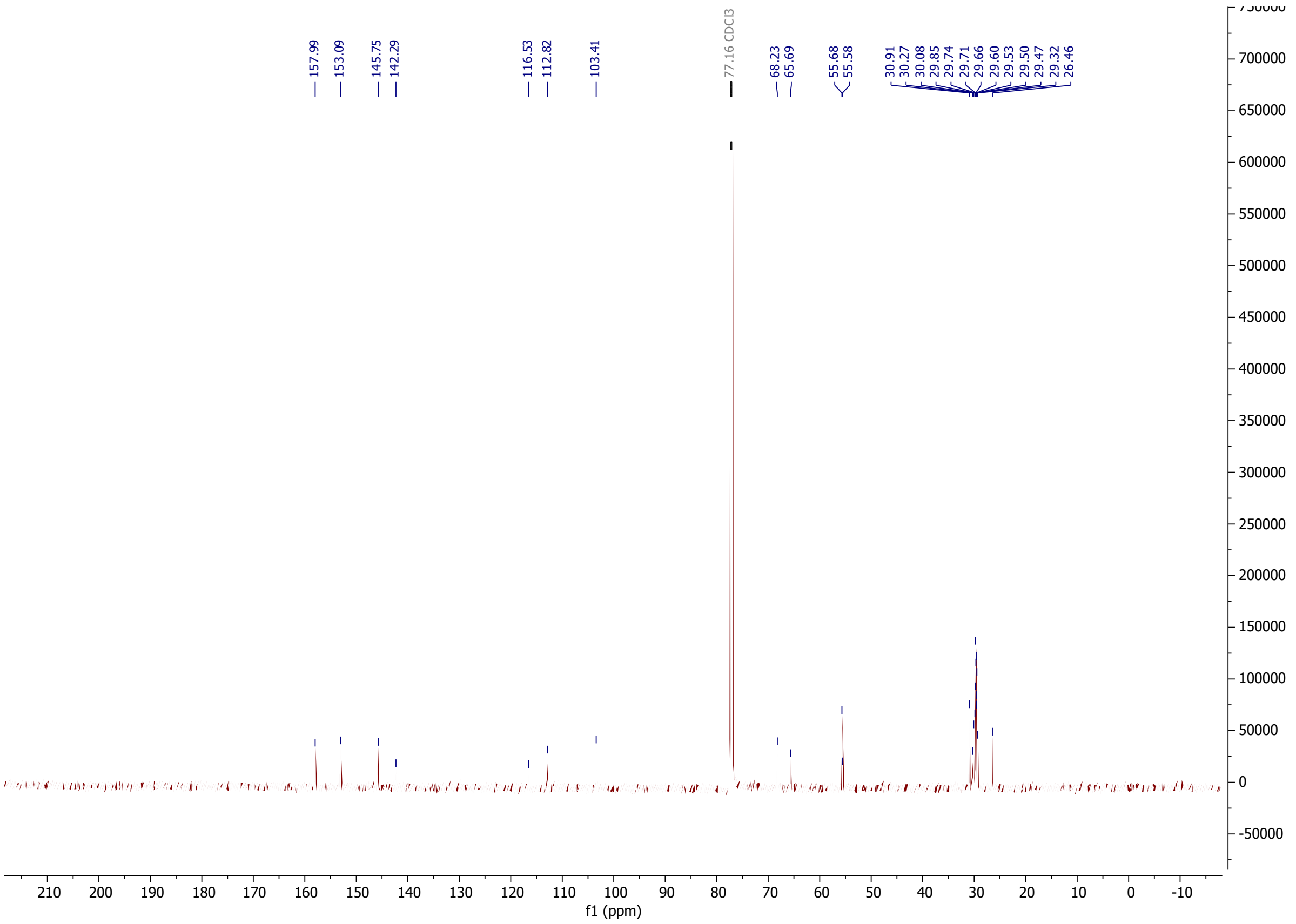

HRMS

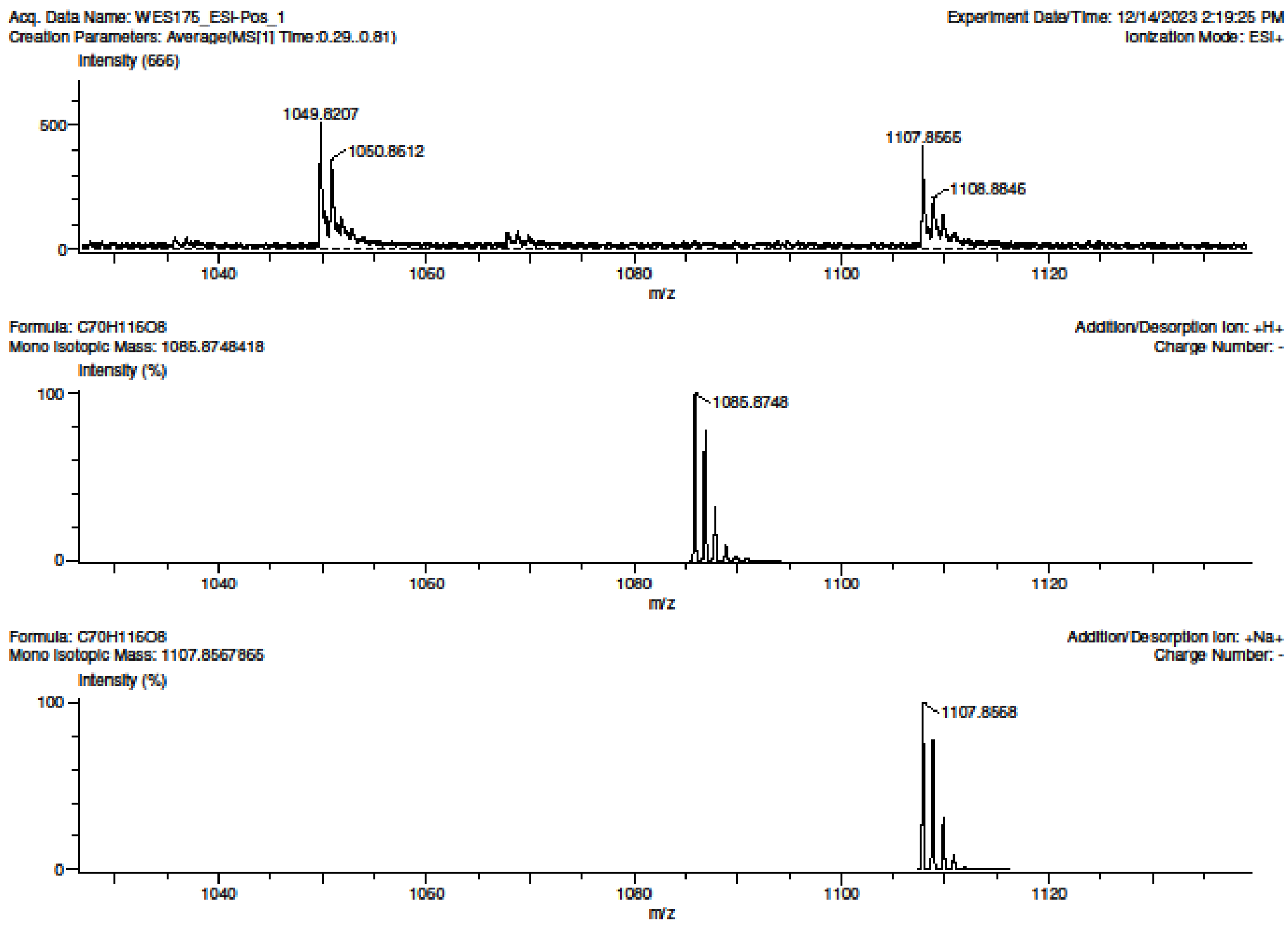
Acq. Data Name: WES175_ESI-Pos_1
Creation Parameters: Average(MS[1] Time:0.29..0.81)
Experiment Date/Time: 12/14/2023 2:19:25 PM
Ionization Mode: ESI+
Intensity (556)
1049.8207
1050.8612
1107.8555
1108.8845
m/z
Formula: C70H116O8
Mono Isotopic Mass: 1085.8748418
Addition/Desorption Ion: +H+
Charge Number: -
Intensity (%)
1085.8748
m/z
Formula: C70H116O8
Mono Isotopic Mass: 1107.8567865
Addition/Desorption Ion: +Na+
Charge Number: -
Intensity (%)
1107.8568
m/z
